# Identification, expression, alternative splicing and functional analysis of pepper WRKY gene family in response to biotic and abiotic stresses

**DOI:** 10.1371/journal.pone.0219775

**Published:** 2019-07-22

**Authors:** Jingyuan Zheng, Feng Liu, Chunhui Zhu, Xuefeng Li, Xiongze Dai, Bozhi Yang, Xuexiao Zou, Yanqing Ma

**Affiliations:** 1 Institute of Vegetable Research, Hunan Academy of Agricultural Sciences, Changsha, China; 2 Institute of Plant Protection, Hunan Academy of Agricultural Sciences, Changsha, China; National Taiwan University, TAIWAN

## Abstract

WRKY proteins are a large group of plant transcription factors that are involved in various biological processes, including biotic and abiotic stress responses, hormone response, plant development, and metabolism. WRKY proteins have been identified in several plants, but only a few have been identified in *Capsicum annuum*. Here, we identified a total of 62 *WRKY* genes in the latest pepper genome. These genes were classified into three groups (Groups 1–3) based on the structural features of their proteins. The structures of the encoded proteins, evolution, and expression under normal growth conditions were analyzed and 35 putative miRNA target sites were predicted in 20 *CaWRKY* genes. Moreover, the response to cold or CMV treatments of selected *WRKY* genes were examined to validate the roles under stresses. And alternative splicing (AS) events of some *CaWRKYs* were also identified under CMV infection. Promoter analysis confirmed that *CaWRKY* genes are involved in growth, development, and biotic or abiotic stress responses in hot pepper. The comprehensive analysis provides fundamental information for better understanding of the signaling pathways involved in the WRKY-mediated regulation of developmental processes, as well as biotic and abiotic stress responses.

## Introduction

WRKY transcription factors constitute one of the largest families of regulatory proteins in plants. Proteins in this family contain at least one or two highly conserved domains of ~60 amino acids, which are characterized by the hallmark heptapeptide WRKYGQK, followed by a C_2_H_2_ or C_2_HC zinc-finger motif [1, 2]. The WRKY domain facilitates binding to the W-box in the promoter of a target gene to modulate transcription [3]. As deduced from nuclear magnetic resonance (NMR) analysis of a WRKY domain of AtWRKY4, the conserved WRKYGQK sequence is directly involved in DNA binding [4]. WRKY proteins can be divided into three distinct groups based on the number of WRKY domains and certain features of the zinc-finger-like motif [1]. Group I proteins contain two WRKY domains, including a C2H2 motif. Both Groups II and III have a single WRKY domain, but Group II has a C2H2 motif, and the zinc-finger motif of Group III is C2HC [1]. Group II can be further divided into five subgroups (i.e., IIa–e) depending on the phylogeny of the WRKY domain [1]. Although further efforts are required to uncover the origin and evolution of the WRKY family, this family has greatly expanded in plants during evolution. A recent study showed that gene duplication events have played a critical role in the expansion of *WRKY* genes, which contain two WRKY domains that appear to be the ancestor of the single-WRKY domain encoding genes [5]. Domain gain and loss is a divergent force for the expansion of the WRKY family; most members of Groups I and II appear to predate the divergence of dicots and monocots [6].

Since the first cDNA encoding a WRKY protein, SPF1, was cloned from potato [7], a large number of WRKY proteins have been identified from different plant species [8–10]. Previous studies have identified 72 *WRKY* genes in *Arabidopsis* [1], 109 in rice [11], 55 in cucumber [12], 104 in poplar [13], 83 in tomato [14] and 79 in potato [15]. Several studies have implicated WRKY proteins in plant defense against various biotic stresses, such as fungal [16, 17], bacterial [18], and viral pathogens [19–23], and abiotic stresses, including salinity, drought, cold, and heat [10, 24–26]. WRKY proteins also play important regulatory roles in developmental processes and signal transduction processes mediated by plant hormones, such as salicylic acid (SA) [27], jasmonic acid [27] and so on. Numerous WRKY proteins are key factors that control plant response to pathogen infections that can trigger SA-dependent defense signaling [28,29].

Pepper is an economically important cultivated vegetable in China and other temperate regions. Two drafts of the hot pepper (Mexican landrace of *Capsicum annuum* L. cv. CM334 and Zunla-1 landrace of *C*. *annuum*) genome sequences have been reported [17, 30]. In the present study, we searched the Zunla-1 genome sequence to identify *WRKY* genes of hot pepper (*CaWRKY*). Subsequently, we compared the structure of the encoded proteins and the expression profiles of *CaWRKY* genes with those of their putative homologs in *Arabidopsis thaliana* WRKY (*AtWRKY*), and two other Solanaceae plants: tomato and potato. We also analyzed the expression of the identified *CaWRKY* genes under normal growth conditions and cold or CMV treatments. These studies provide information on the relationship between the evolution and functional divergence of the WRKY family in hot pepper and are useful for understanding the role of *CaWRKY* genes in plant response to biotic and abiotic stresses.

## Materials and methods

### Sequence database searches

The pepper annotated genes and proteins were obtained from the hot pepper genome database (https://www.ncbi.nlm.nih.gov/assembly/GCA_000710875.1#/def_asm_Primary_Assembly). [30]; the *Arabidopsis* proteins sequences were obtained from TAIR. We searched for WRKY proteins from 34,476 annotated pepper proteins. *Arabidopsis* WRKY proteins sequences were downloaded from TAIR (http://www.arabidopsis.org/), and 72 *Arabidopsis* WRKY proteins were used as query sequences in BLASTP searches against the predicted pepper WRKY proteins. The HMM search program was used to predict the WRKY domains (PF03106) of all selected candidate proteins, with the E-value set to -10 according to the HMMR User’s Guide. The new WRKY-like sequences, as confirmed by HMM search, in the pepper genome were reiteratively used to search the predicted pepper proteins until no new sequences were found. The transcriptome and EST sequences of pepper were downloaded from the pepper genome database and NCBI database (SRP100904 and SRX1959970).

### Multiple sequence alignment, gene structure construction, and phylogenetic analysis

The 62 amino acids that span the WRKY core domain of all CaWRKY proteins and 72 AtWRKY proteins were used to create multiple protein sequence alignments by CLUSTALW methods. Gene structure was obtained from the pepper gene annotation GFF3 file downloaded from the hot pepper genome database. The phylogenetic tree was constructed based on the amino acid sequences of WRKY domains with the use of the neighbor-joining method. The exon–intron organizations of pepper *WRKY* genes were identified by comparing their CDS sequences to the corresponding genomic sequences with PECE.

### Promoter sequence analysis for potential *cis*-acting elements

Upstream regions (~1500 bp) of the *CaWRKY* genes were downloaded from hot pepper genome database (https://www.ncbi.nlm.nih.gov/assembly/GCA_000710875.1#/def_asm_Primary_Assembly). [30] and were searched for regulatory elements using the PlantCARE (Available online: http://bioinformatics.psb.ugent.be/webtools/plantcare/html/). These elements included ABRE (*cis*-acting element involved in the abscisic acid responsiveness), W-box (binding site for the WRKY transcription factor in the defense response), CGTCA (*cis*-acting regulatory element involved in the MeJA-responsiveness), TCA (*cis*-acting element involved in salicylic acid responsiveness), TC-rich repeats (*cis*-acting element involved in the defense and stress responsiveness), MBS (MYB binding site involved in drought induction), WUN (wound-responsive element), GARE (gibberellins-responsive element), and LTR (low temperature-responsive element).

### Identification of alternative splicing events in *CaWRKY* genes

The alternative splicing (AS) events were classified into six basic types, including exon skipping (skippedExon), intron retention (IR), alternative 5′ splice site (Alt5′site), alternative 3′ splice site (Alt3′site), alternative first exon (altFirstExon) and alternative last exon (altLastExon). Using the previous transcriptome data from Illumina and PacBio SMRT RNA-seq for CMV infection in hot pepper [31], the AS events in *CaWRKY* genes were detected.

### Plant materials, growth conditions and treatments

The hot pepper (*C*. *annuum*) cultivar Zunla-1 was used throughout the study. Seeds were germinated in pots that contained vermiculite and grass charcoal soil, the plants were grown in a greenhouse with a 16 h-light and 8 h-dark photoperiod at 25 ± 2°C, and 7–8 week-old seedlings were used in the following treatments. For the cold treatments, seedlings were incubated at 8°C. The above-ground organizations were collected at 0, 1, 3, 6, and 12 h after treatment for RNA extraction. For CMV experiment, CMV-Fny was mock inoculated as described [31]. Three leaves of the pepper with CMV-Fny inoculation from the individual plants at each time (0 h, 6 h, 12 h, 24 h, 48 h and 72 h) were collected for RNA extraction.

### RNA isolation, RT-PCR and quantitative RT-PCR analysis

Total RNA was extracted according to the TRIzol method (Invitrogen). The integrity of RNA was assessed on agarose gels. Total RNA (2 μg) was reverse transcribed with SuperScript III first-Strand Synthesis System (Invitrogen) with the use of an oligo(dT) primer according to the manufacturer’s instructions. The cDNA synthesis reaction was served as the template for PCR. For quantitative RT-PCR, the specific primers were designed according to the WRKY gene sequences by DNAman 7 software. The pepper *β-actin* gene was used as a control (S1 Table).

The SYBR Green real-time PCR assay was carried out in a total volume of 20 μL, containing 0.4 μL of each primer, 10 μL of the 2× SYBR Premix Ex *Taq* II Mix (Takara, Japan), and 100 ng of the template cDNA. Reactions were amplified as follows: 95°C for 3 min, then 40 cycles of 95°C for 10 s, 57°C for 30 s, and 72°C for 10 s. The absence of genomic DNA and non-specific by products of the PCR amplification was confirmed through an analysis of dissociation curves. The melting curves were obtained by slow heating at 0.5°C/s, from 65°C to 95°C while continuously monitoring the fluorescence signal. Amplifications were performed in a CFX96 Real-time PCR Detection System (Bio-Rad, USA). All samples were run in quadruple replicates, with two biological replicates for each gene. Data analysis for relative quantification was performed by using the CFX Manager software to determine the relative quantification of the target gene compared with the reference gene (*β-actin*) via the relative quantification (^ΔΔ^CT) method.

## Results

### Chromosomal location analysis of the WRKY superfamily in hot pepper

**Fig 1:**
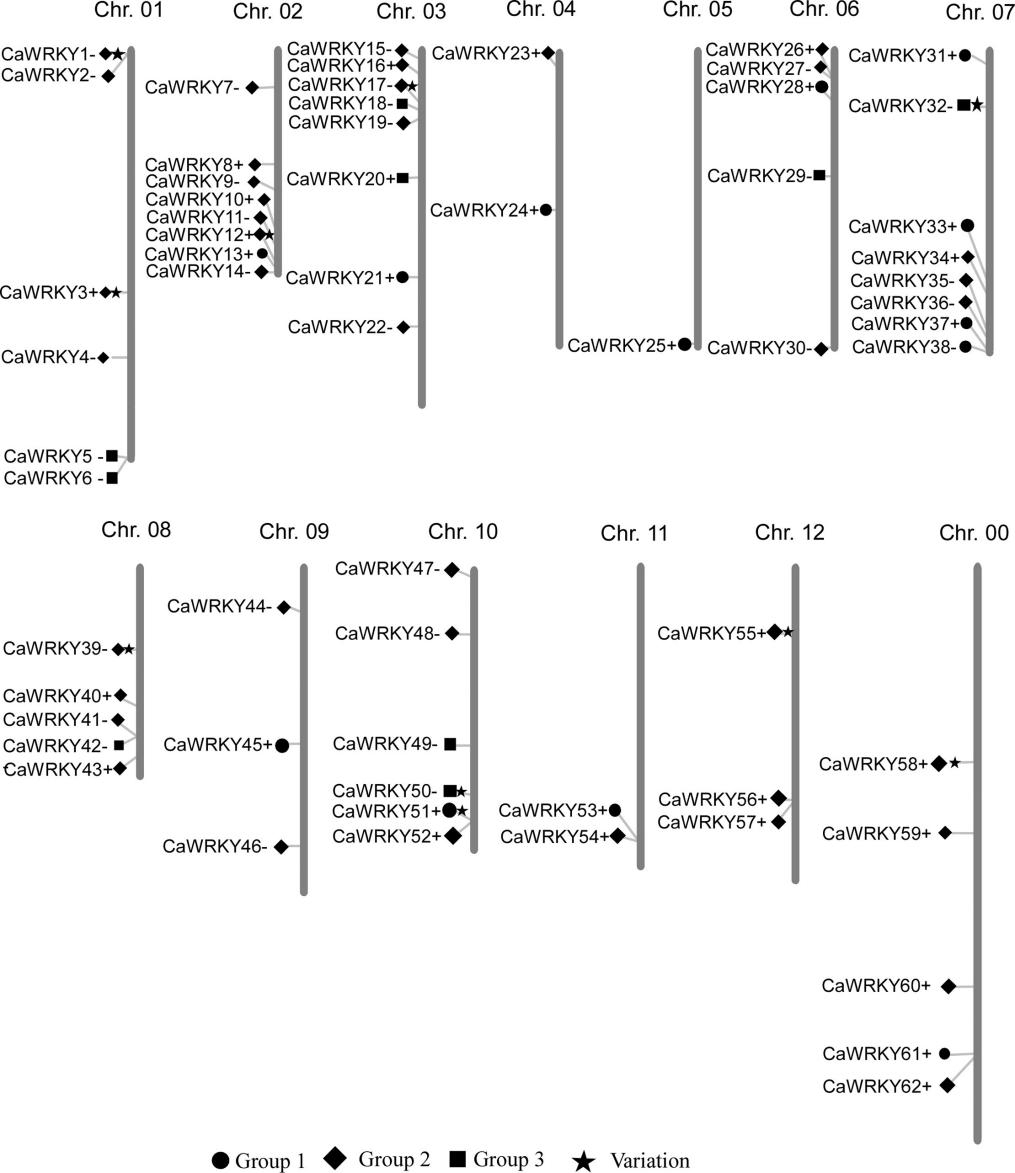
Mapping of the WRKY gene family on *Capsicum annuum* L. The size of a chromosome is indicated by its relative length. The 62 putative WRKY genes were renamed from *CaWRKY1* to *CaWRK62* according to their chromosomal locations. 5 *WRKY* genes could not be mapped to any chromosome were named *CaWRKY58*–*62*, respectively, and mapped to the putative chromosome 0 according to their raw scores in a search of hot pepper WRKY proteins with the MapInspect program.

To identify WRKY proteins encoded in the hot pepper genome, publicly available genome sequences of ZUNLA-1 were searched with the BLASTP, based on HMM. A total of 62 putative *WRKY* genes were identified in the hot pepper genome by HMMER, with *Arabidopsis WRKY* genes as a model. The sequences encode 62 putative *WRKY* genes. All these genes were consecutively named *CaWRKY1* to *CaWRKY62* according to their chromosomal locations in *C*. *annuum* (Fig 1). Each candidate gene was validated by checking for the presence of the WRKY motif. Only 11 different accession numbers for the same amino acid sequences were detected in GenBank (S2 Table). A total of 57 *CaWRKY* genes were distributed over the 12 pepper chromosomes with ORF sequences ranging from 414 bp to 2610 bp in length (S2 Table and S3 Table). Only 5 *WRKY* genes (*Capana00g000429*, *Capana00g001033*, *Capana00g003083*, *Capana00g004057*, and *Capana00g004112*) could not be mapped to any chromosome were named *CaWRKY58–62*, respectively, and mapped to the putative chromosome 0. An average of 4.8 *WRKY* genes were present per chromosome, with the highest number of genes (8) located on chromosomes 2, 3, and 7, respectively. Notably, only one *WRKY* gene is on chromosome 5, *CaWRKY25*, which belongs to Group 1 (Fig 1). We further compared the number of *WRKY* genes in different subgroups among the pepper, *Arabidopsis*, rice, cucumber, tomato, potato, cotton, poplar, and cabbage (Table 1). As shown in Table 1, the common feature is that the group 2a *WRKY* genes were much fewer than those of the other subgroups. Moreover, compared with two other Solanaceae plants (tomato and potato), pepper has less number for group 2e and group 3 (Table 1). Most of the *CaWRKYs* (59) have homologue genes in tomato, except for *CaWRKY7*, *CaWRKY19* and *CaWRKY51* (S4 Table).

**Table 1:** The number of WRKY family members in diverse species.

Group	pepper	*Arabidopsis*	Rice	Tomato	Potato	Cucumber	Cotton	Poplar
1	13	13	34	15	13	10	20	50
2a	4	4	4	5	5	4	7	5
2b	6	7	8	8	6	4	16	9
2c	16	18	7	16	16	16	26	13
2d	6	7	11	6	7	8	16	13
2e	8	9	-	17	16	7	13	4
3	9	14	36	11	14	6	14	10
SUM	62	72	100	78	77	55	112	104

Note: *Arabidopsis* (Eulgem et al. 2000), Rice (Wu et al. 2005), Tomato (Huang et al. 2012), Potato (Zhang et al. 2017), Cucumber (Ling et al. 2011), Cotton (Dou et al. 2014) and Poplar (He et al. 2012).

### Classification and phylogenetic analysis of *CaWRKY* genes

**Fig 2:**
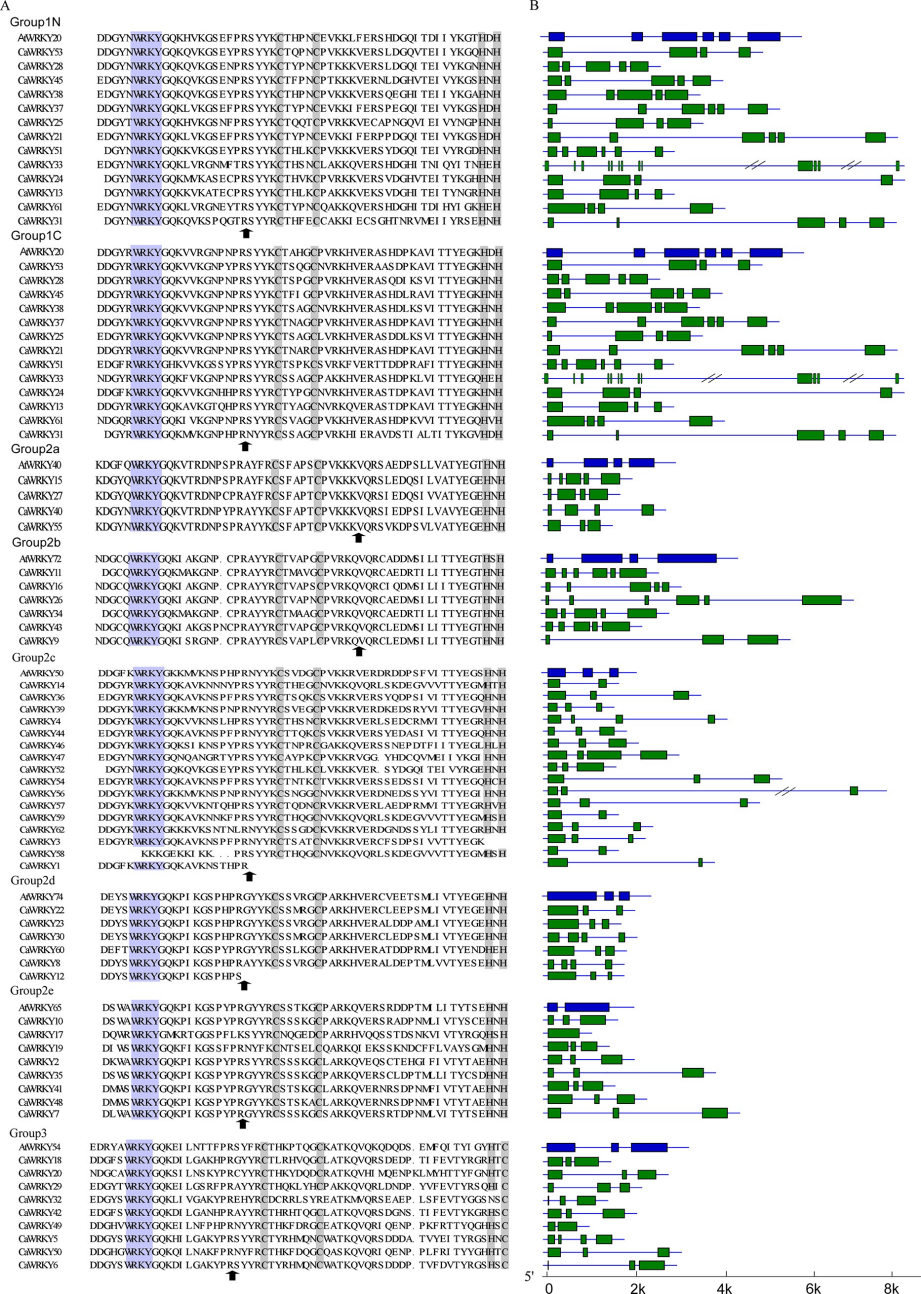
Sequences and gene structures of *CaWRKYs* and selected *AtWRKYs*. **(A) Alignment of *CaWRKYs* and selected *AtWRKYs* domain amino acid sequences.** The N-terminal WRKY domain or the C-terminal WRKY domain of group I WRKY domain are shown by the suffix ‘N’ or ‘C’, respectively. The conserved WRKY amino acid signature and the zinc-finger motif are highlighted in purple and gaps are shown by dot. The position of a conserved intron is indicated by arrowhead. Alignments was performed by Clustal W. **(B) Length of exon/intron of *CaWRKY* genes.** Exons of *CaWRKY* genes are indicated by the green boxes and introns are shown as solid line.

The phylogenetic relationship of the 62 CaWRKY proteins was examined with MEGA6.0 program. The CaWRKY proteins were categorized into seven groups and subgroups (1, 2a, 2b, 2c, 2d, 2e, and 3) according to the features specified for the WRKY superfamily in *Arabidopsis* [32]. A comparison with the domains of seven selected AtWRKY proteins resulted in improved separation of the different groups and subgroups (Fig 2).

The sequences in the WRKY domain were highly conserved. Among these proteins, 13 contained two complete WRKY domains each and belonged to Group 1 with the C_2_H_2_ type zinc-finger structure. A total of 40 CaWRKY proteins belonged to Group 2, with a C_2_H_2_ type zinc-finger structure, and 9 CaWRKY members were identified in Group 3, with a C_2_HC type zinc-finger structure rather than C_2_H_2_. The multiple sequence alignment and phylogenic tree of the CaWRKY domain showed that Group 2 proteins can be clearly divided into five distinct subgroups (2a–2e). A total of 4, 6, 16, 6, and 8 proteins were clustered in Subgroups 2a, 2b, 2c, 2d, and 2e, respectively. Notably, subgroup 2c contained 16 members, and 13 of them contained a complete WRKY domain. However, CaWRKY1 contained the conserved heptapeptide WRKYGQK sequences but lacked the amino acids that form the zinc-finger motif. CaWRKY58 lacked the conserved WRKY amino acid signature but had the zinc-finger motif. CaWRKY3, with the specific zinc-finger structure of C-X_4_-C-X_23_, replaced the structure of C-X_4_-C-X_23_-H-X_1_-H and also clustered in Subgroup 2c. Interestingly, CaWRKY12 did not contain the complete zinc-finger motif, but clustered in Subgroup 2d in the phylogenetic analysis. The main mechanisms of gene evolution are sequence divergence, duplication, and recombination. Recombination leads to rearrangement of domains; thus, domain gain and loss is a divergent force for expansion of the WRKY transcription factor family [6, 33].

As shown in Fig 2, although the WRKYGQK signature was highly conserved in the pepper WRKYs, the protein variants were distributed among all groups. Except for *CaWRKY58*, which lacked the conserved WRKY core sequence, each of the remaining 61 *CaWRKY* genes contained one of four WRKY domain amino acid sequence variants, WRKYGQK was the most common variant and was shared by 55 genes. Only 4 (*CaWRKY 39*, *CaWRKY50*, *CaWRKY*56, and *CaWRKY*62) contained WRKYGKK, whereas *CaWRKY17* contained WRKYGMK and *CaWRKY51* contained WRKYGHK, respectively (Fig 2).

**Fig 3:**
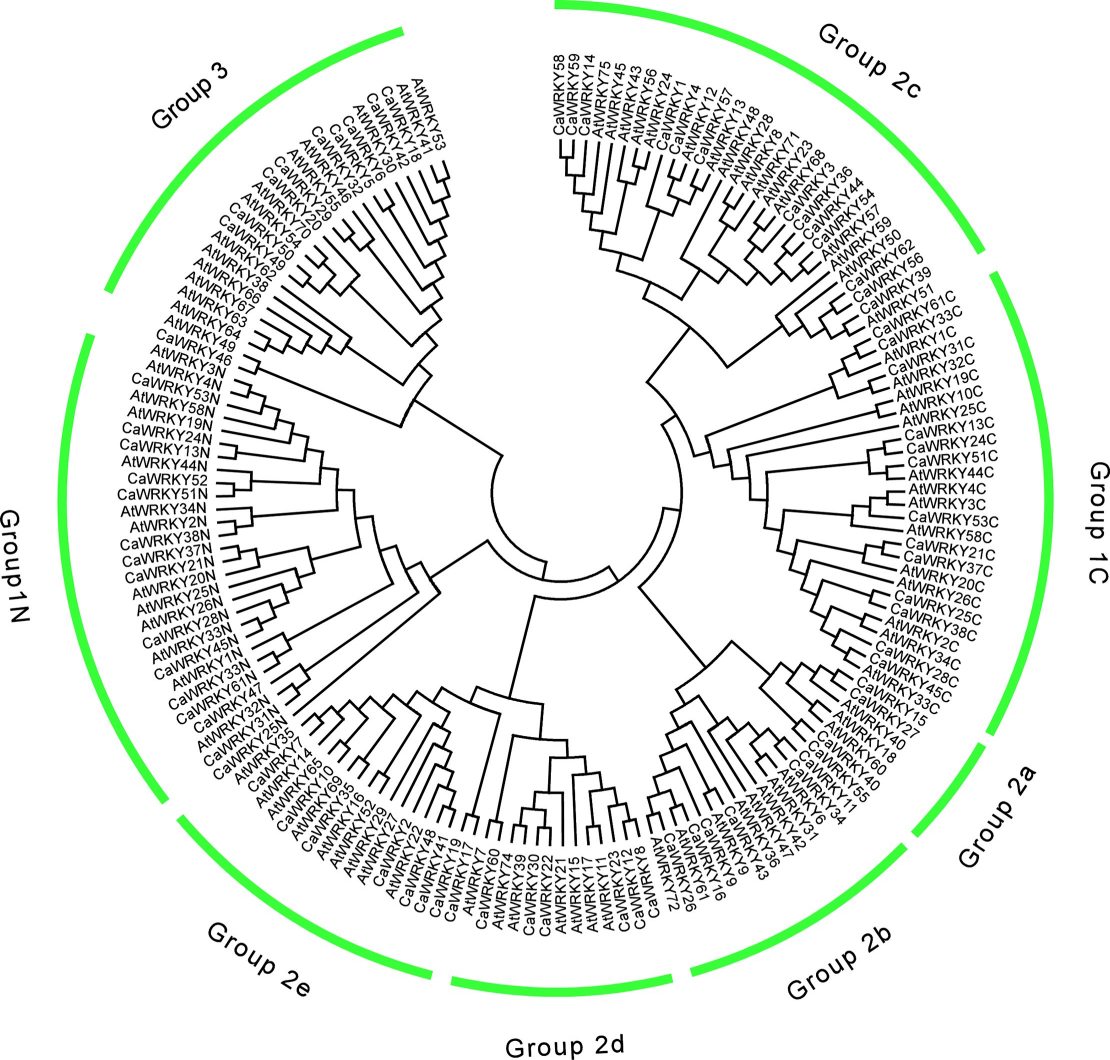
Unrooted Phylogenetic tree representing relationships among WRKY domains of hot pepper and *Arabidopsis*. The phylogenetic tree was constructed using neighbor-joining method in MEGA6.0 based on amino acid. Bootstrap test (≥500) based on 1000 replications are exhibited beside the nodes.

To better understand the phylogenetic relationships between the *CaWRKY*s and *AtWRKY*s, an evolutionary framework in an unrooted phylogentic tree was constructed according to the conserved WRKY domains (including the N-terminal and C-terminal domains) by using the neighbor-joining method. As shown in Fig 3, eight subgroups formed a typical topological tree (groups 1N, 1C, 2a–e, and 3), and the N-terminal and C-terminal domains were separately located at the phylogenetic tree. groups 2a and 2b were two subgroups from the same branch, and this condition is similar to groups 2d and 2e, while groups 2c and 1C were close in the genetic tree. *CaWRKY46* is a member of group 2c but is more similar to group 3 from the same branch based on the phylogenetic analysis.

### Identification of structure and conserved motifs of the CaWRKY family

An analysis of the structures of *CaWRKY* genes revealed that all *WRKY* genes had at least one intron inserted. Two major types of introns exist in the conserved WRKY domains of *CaWRKY* genes (Fig 2), which are similar to those conserved in *Arabidopsis*. One of the introns is spliced at the codon of R and named PR intron; the other is the VQR intron. We noticed that the distribution of introns is coincident with their alignment cluster in the *WRKY* gene subgroups. The VQR introns arise in the C_2_H_2_ motif of the zinc-finger region and are distributed in subgroups 2a and 2b, whereas the PR intron is observed in the WRKY domains of genes in groups 1 and 3 and Subgroups 2c–e.

**Fig 4:**
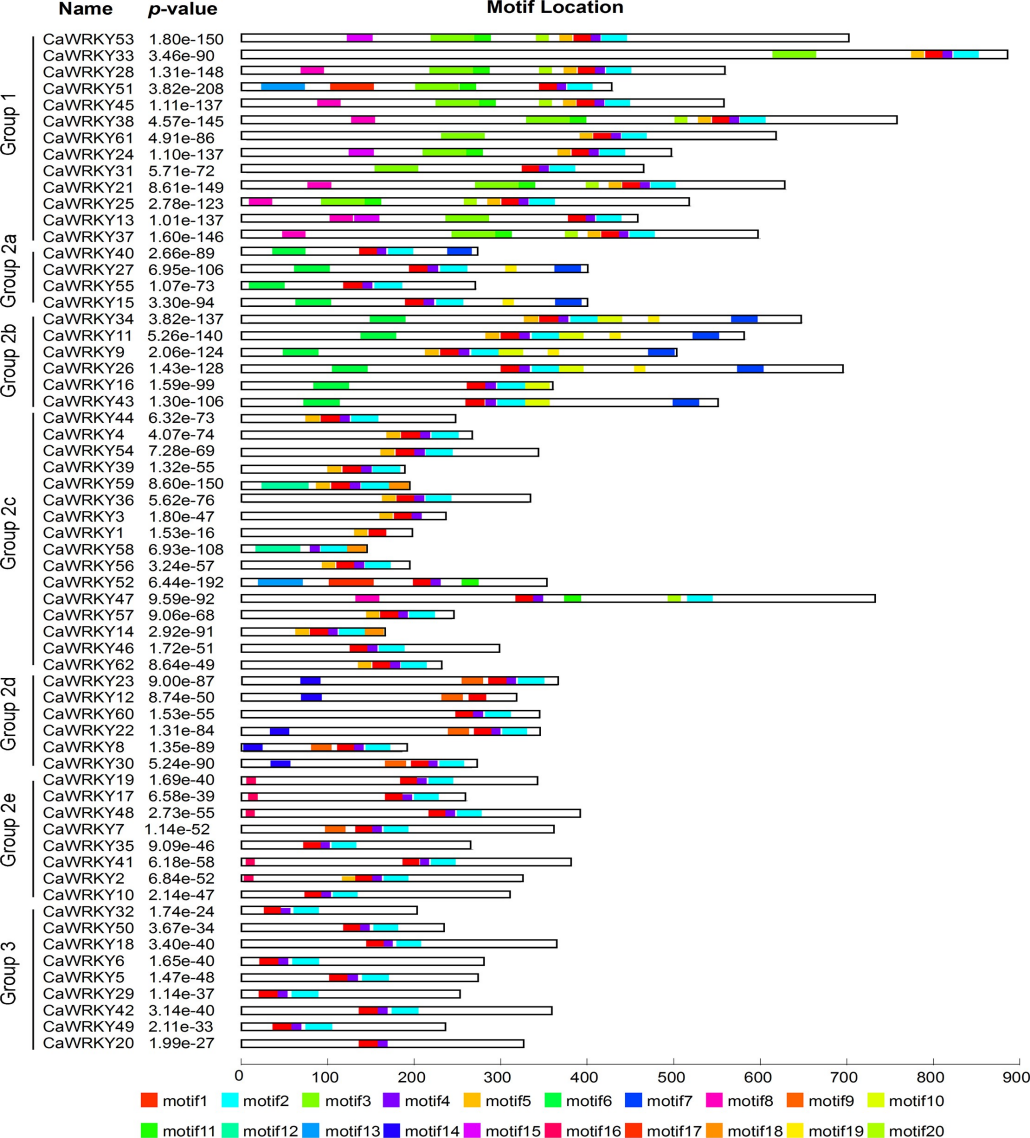
Conserved motifs of amino acid sequences of CaWRKY proteins. Motif analysis was performed using MEME4 software. The black rectangle represents the corresponding WRKY protein and its length. The different-colored boxes representation of the related motifs and their position in each WRKY amino acid sequence.

The conserved motifs of the WRKY family proteins in hot pepper were analyzed by using the MEME online software, and 20 motifs were identified (Table 2 and S2 Fig). These 20 motifs were distributed across different subgroups in the phylogenetic tree (Fig 4). Except for *CaWRKY58*, one or more conservative motifs around the WRKY domain motif can be detected in a WRKY protein. Similar motif compositions were generally shared by *WRKY* genes from the same group or subgroup, suggesting their functional similarity (Fig 4). Motifs 1 and 4 were detected in all the WRKY members, except for *CaWRKY1* and *CaWRKY12*. Interestingly, these motifs appear as a pair in most cases, indicating their functional relationship in the *WRKY* genes. Motif 16 has uniquely dispersed partial members of group 2e. In group 2d, Motif 7 is the unique motif except in *CaWRKY60*.

**Table 2:** The sequences of different motifs for WRKY gene family in hot pepper.

Motif	Width	Best Possible Match
1	20	DILDDGYRWRKYGQKVIKGN
2	29	GCPVRKHVERCSEDPKMVITTYEGEHNHP
3	50	DGYNWRKYGQKQVKGSEYPRSYYKCTHPNCPVKKKVERSHDGHITEIVYK
4	11	PYPRAYYRCTH
5	15	RKIREPRFAFQTRSE
6	38	LLEELNRMMEENKRLRMMLTQVCENYNALQMHFMEIMQ
7	28	LIEQMASAITADPNFTAALAAAISGIIG
8	27	LPICRSPYLTIPPGLSPSVLLDSPVLL
9	24	GSSGRCHCSKKRKHRVKRVIRVPA
10	26	LPLAATAMASTTSAAANMLLSGSMTS
11	19	GHHNHPKPQPNRRFAMGGH
12	50	HEFSLMNKKRSNTHAKEVLLFQGKNNGFLGLMASMETPSGVTNSFEDDVM
13	50	RPRCPVYKSFSELLTGAVDISSTNVHSEMAITAIRPKTIRLKPATNHALV
14	23	MEIEIVADAAVNKFKKVISLLDR
15	29	PLQGPFGMSHQQALAHVTAQAACSQSYMQ
16	11	DWDLHAVVRGC
17	50	VLYKPIGKLAQKKTIPLLENKGSSVSDQQRVIADAEAHIQSANEVKQQHD
18	21	IDKSTDNFEQILHQMQVYASF
19	12	PPFPTITLDLTA
20	15	GDDMDEDEFEAKRRK

### *CaWRKY* genes as the target genes of miRNAs in hot pepper

Previous studies have shown that miRNAs can bind to their targets with perfect/near-perfect complementarity, allowing an effective prediction of the target genes through computational analysis [34]. In this study, we searched the miRNA target sites in all *CaWRKY* genes of hot pepper using psRNATarget [35]. The results showed that 35 putative miRNA target sites were predicted in 20 *CaWRKY* genes (S3 Table). These miRNAs included can-miR167a-5p, can-miR171a-5p, can-miR172a-5p, can-miR482a-5p, and can-miR5303a. Among these putative target sites, interestingly, three *CaWRKY* genes (*CaWRKY17*, *CaWRKY38* and *CaWRKY51*) were predicted to be targeted by at least two miRNAs in hot pepper (S5 Table).

### Expression profile of *CaWRKY* genes in various developmental stages

**Fig 5:**
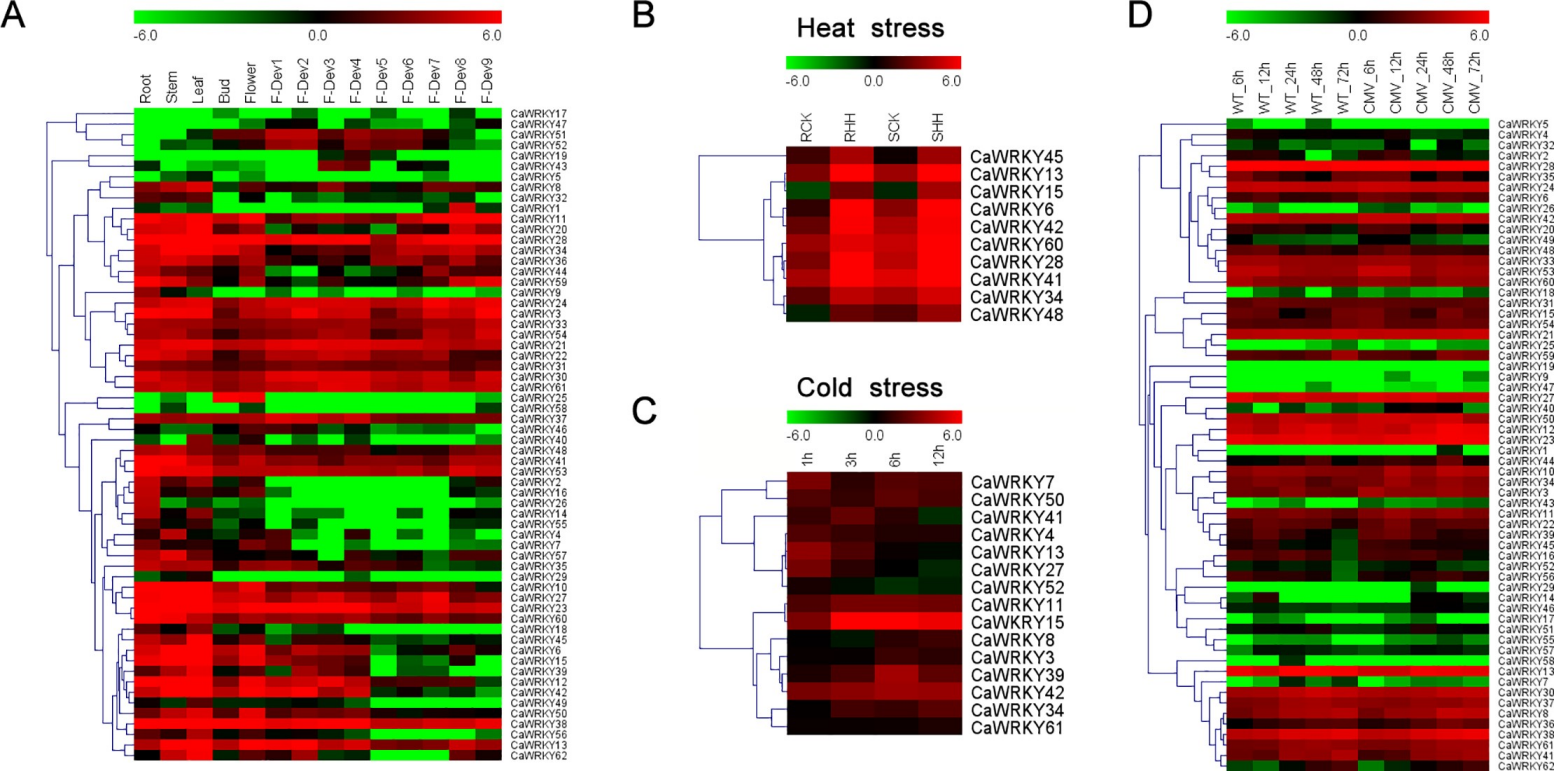
Heatmaps of expression profiles and hierarchical clustering of *CaWRKY* genes expression levels in normal tissues and different treatments. All the heatmaps were generated using MeV4.9 software with log2 transformed FPKM values. **(A)** The transcriptome database included two flower stages (closed flower and open flower), five pre-breaker stages (0–1 cm, 1–3 cm, 3–4 cm, 4–5 cm and mature green fruit), one breaker stage (when the fruit was turning red) and three post-breaker stages (3, 5, and 7 days after breaker), which were referred to as Root, Stem, Leaf, Bud, Flower, F-Dev-1, F-Dev-2, F-Dev-3, F-Dev-4, F-Dev-5, F-Dev-6, F-Dev-7, F-Dev-8, and F-Dev-9, respectively. **(B)** Heatmaps of expression profiles of *CaWRKYs* under heat stress. RCK, control of tolerant cultivar ‘R597’; RHH, heat treatment of ‘R597; SCK, control of susceptible ‘S590’; SHH, heat treatment of ‘S590’. **(C)** Heatmap of expression profiles of *CaWRKYs* under cold treatments. **(D)** Heatmaps of expression profiles of *CaWRKYs* under CMV infection (6 h, 12 h, 24 h, 48 h and 72 h).

Gene expression studies can provide essential indications with regard to the functions of a gene. To analyze the role and obtain the expression profile of WRKY genes in hot pepper, 14 transcriptome databases in different developmental stages under normal growth conditions were downloaded from the pepper genome database (https://www.ncbi.nlm.nih.gov/assembly/GCA_000710875.1#/def_asm_Primary_Assembly). The heat map illustration of expression profiles of *CaWRKY* genes is shown in Fig 5A. As shown in the Fig 5A, 62 genes clustered together in several different expression groups. However, not all the predicted *CaWRKY* genes were expressed in plants under normal growth conditions. Among the 62 predicted genes, 60 were expressed in at least one of the five tissues, whereas 30 of these genes were detected in all the tissues, while 2 genes were detected in only 1 tissue each: *CaWRKY25* in flower and *CaWRKY19* in fruit. The other 2 genes, *CaWRKY58* and *CaWRKY59*, did not show any detectable expression in the abovementioned tissues.

### Expression profile of *CaWRKY* genes under abiotic and biotic stress conditions

**Fig 6:**
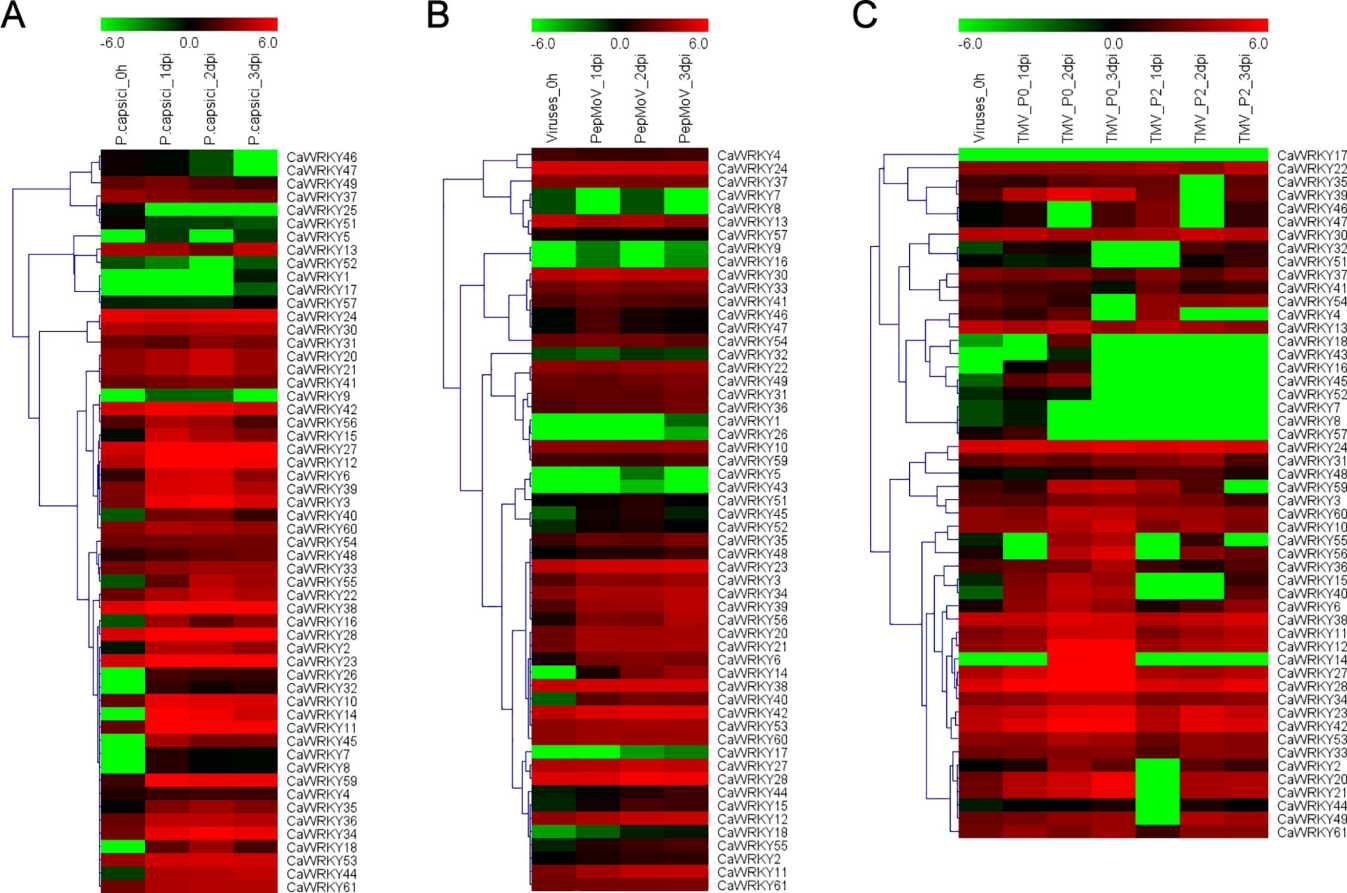
Heatmaps of expression profiles of *CaWRKY* genes in responses to *P*. *capsici*, PepMoV, TMV pathogen at 0, 1, 2, and 3 days post inoculation. All the heatmaps were generated using MeV4.9 software with log2 transformed FPKM values. **(A)** The expression patterns of *CaWRKYs* under *P*. *capsici* infection. **(B)** The expression patterns of *CaWRKYs* under PepMoV infection. **(C)** The expression patterns of *CaWRKYs* under TMV infection.

**Fig 7:**
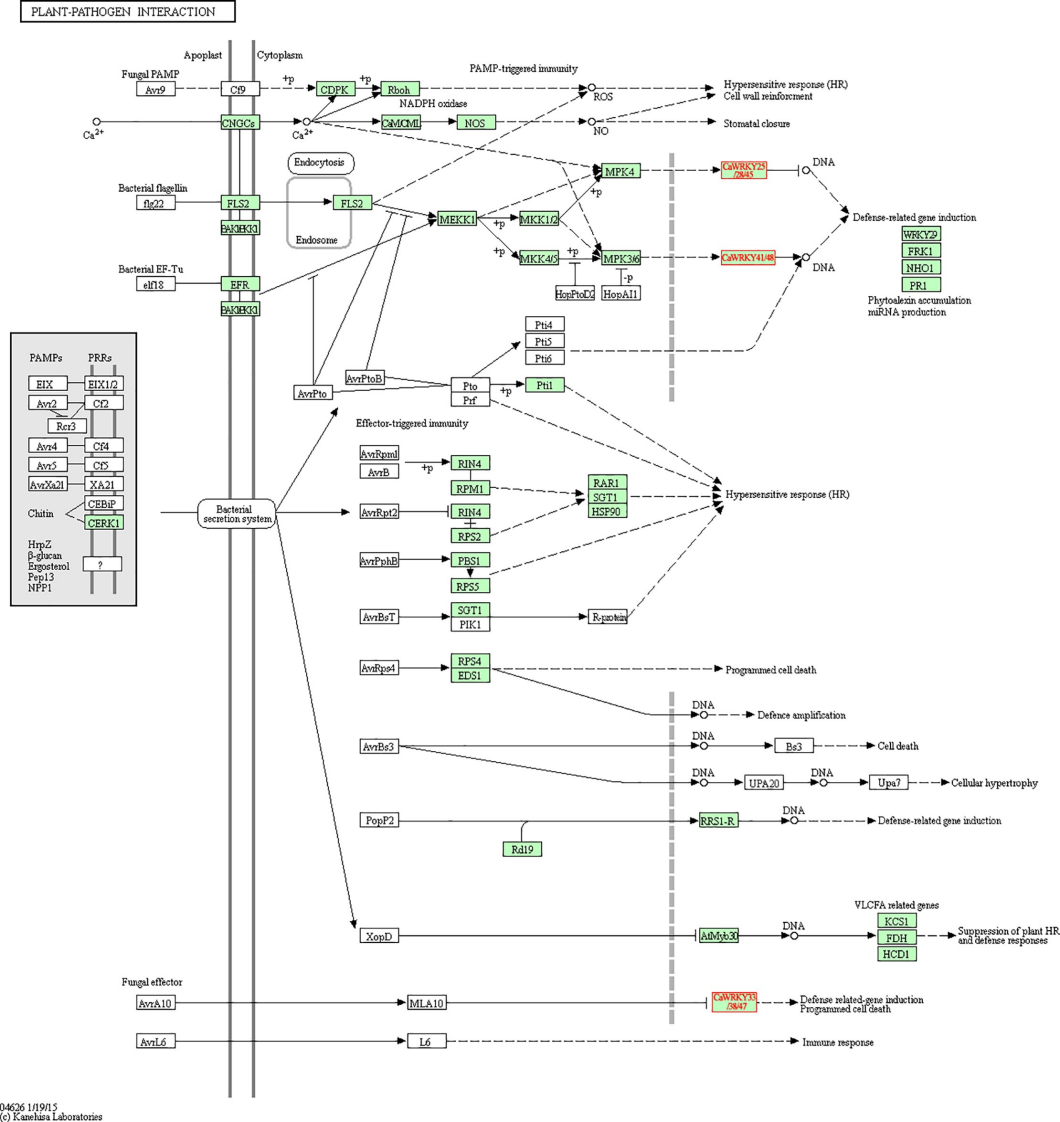
Schematic of the pathway category of ‘Plant-pathogen interaction’. Eight *CaWRKY* genes (*CaWRKY25/28/45*, *CaWRKY41/48 and CaWRKY33/38*/47) are included. The pathway map was download from the KEGG database (https://www.kegg.jp/).

To determine the response of *CaWRKY* genes to abiotic and biotic stresses, the RNA-seq data of hot pepper subjected to different treatments were analyzed in the study [36–38]. There are 10 *CaWRKY*s were induced under heat stress in heat-tolerant cultivar R597 (Fig 5B and S6 Table) [37]. Furthermore, 15 *CaWRKYs* were shown to differentially expressed under cold treatment (Fig 5C). And *CaWRKY13*, *CaWRKY15*, *CaWRKY34* and *CaWRKY42* were differentially expressed in both heat and cold stresses (Fig 5B and 5C). Cucumber mosaic virus (CMV) is a virus that can cause leaf distortion and fruit lesions, affecting pepper production. In our previous study, several genes involved in plant-pathogen interaction, including *CaWRKY45* were identified using illumina- and SMRT-based RNA-seq [31]. Expression patterns of the 62 *CaWRKYs* also showed that most of them were differentially expressed under CMV inoculation (Fig 5D and S6 Table). To further determine the potential functions of WRKY in response to other biotic stresses, the expression profiles of these 62 *CaWRKYs* under three pathogen (*Tobacco mosaic virus* (TMV), *Pepper mottle virus* (PepMoV), and *Phytophthora capsici*) infection were analyzed using the RNA-seq data [38]. Most of these *CaWRKYs* were induced under the three different virus inoculation (Fig 6 and S6 Table). And eight *CaWRKY* genes were up-regulated in the resistant TMV-P0, comparing to TMV-P2 which is susceptible by ‘CM334’ (Fig 6C and S6 Table) [38]. In addition, some *CaWRKYs* including *CaWRKY25*, -*28*, -*33*, -*38*, -*41*, -*45*, -*47*, -*48* were involved in different plants and pathogen interaction pathway (bacterial or fugal) (Fig 7).

### Validation of the expression profiles of *CaWRKY* genes under cold and CMV infection condition

**Fig 8:**
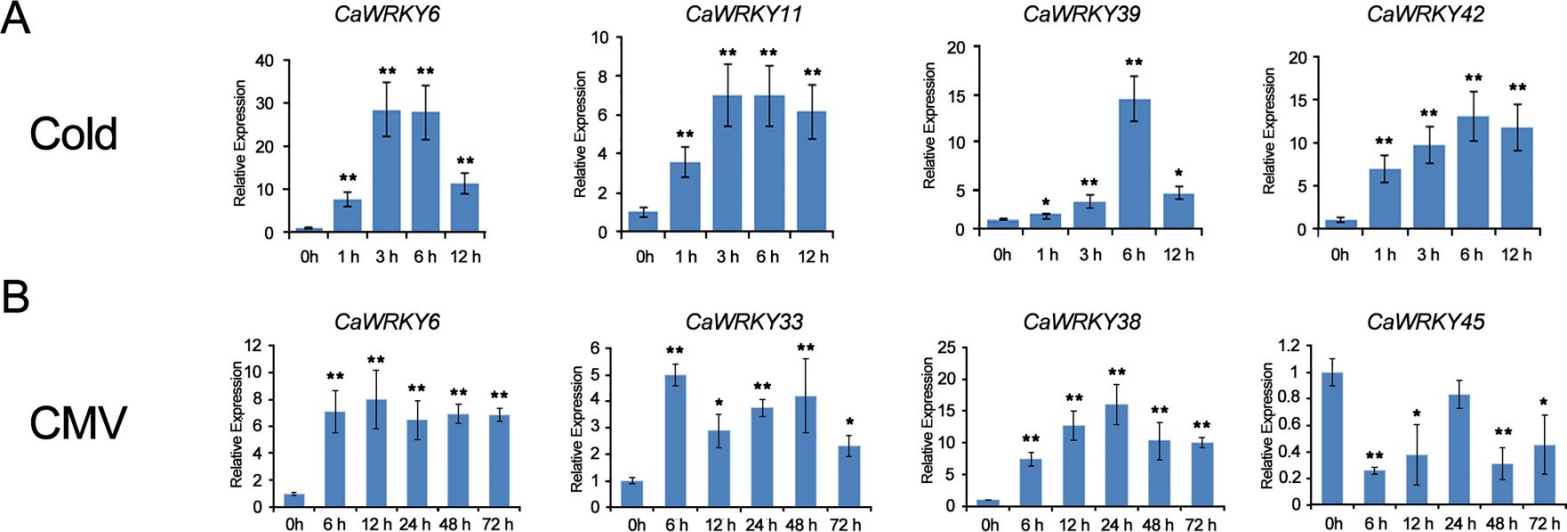
Quantitative RT-PCR validation of seven *CaWRKY* genes expression patterns under cold treatment or CMV infection. Error bars were obtained from three technical replicates. Asterisks reveal the gene significantly up-regulated or down-regulated under abiotic stresses by *t* test (*P < 0.05, **P < 0.01). Significant differences of the expression level among the different stages were determined by Student’s *t*-rest (*P < 0.05 or **P < 0.01).

To analyze the expression patterns of *CaWRKY* genes during plant response to the abiotic and biotic stresses, we selected 16 *WRKY* genes and used quantitative RT-PCR to examine their expression in response to the cold stresses. All the 16 selected WRKY genes exhibited differential expression under cold stress conditions. As shown in the figure, most of the selected *WRKY* genes were up-regulated in response to cold stress. And *CaWRKY27* (Capana06g001110) was highly expressed at the 3 h after cold stress, which has been reported to be in response to chilling in pepper (S2 Fig). The *WRKY* genes *CaWRKY6* and *CaWRKY42*, which were classified into group 3, were dramatically induced by the cold stress and peaked at 6 and 3 h after treatment, respectively. Furthermore, we also random selected 16 *CaWRKY* genes from CMV infection in hot pepper for validation by qRT-PCR (S3 Fig). Most of these CaWRKYs were up-regulated under CMV inoculation except for *CaWRKY20 and CaWRKY45*. Some of the *CaWRKYs* are involved in both abiotic and biotic responses, such as *CaWRKY6* in our study (Fig 8). In pepper, WRKY6 has been reported to regulate *R*. *solanacearum* resistance and provide tolerance to high-temperature and humidity (Table 3) [39]. The results indicated that many *CaWRKY* genes were induced under cold stress or CMV infection.

**Table 3:** The function of some *CaWRKY* genes reported in hot pepper.

Gene ID^a^	Reported gene^b^	NCBI ID	Functions	Reference
*CaWRKY45*	*WRKY-a*	AY391747.1	TMV and *Xanthomonas campestris*	Park et al., 2006^[^^42^^]^
*CaWRKY59*	*WRKY-b*	AY743433.1	TMV infection	Lim et al., 2011^[^^23^^]^
*CaWRKY27*	*CaWRKY1*	AY789641.1	Drought tolerance, TMV and *Pseudomonas syringe* infection	Moon et al. 2014^[^^26^^]^ Oh et al., 2008^[^^22^^]^
*CaWRKY15*	*CaWRKYd*	DI201993.1	TMV infection	Huh et al. 2012^[^^47^^]^
*CaWRKY10*	*CaWRKY27*	DQ102364.1	*Ralstonia solanacearum* resistance	Dang et al., 2013^[^^50^^]^
*CaWRKY13*	*CaWRKY58*	AY740531.1	*Ralstonia solanacearum* resistance	Wang et al., 2014^[^^18^^]^
*CaWRKY6*	*CaWRKY30*	FJ360844.1	Various pathogens (TMV *etc*.)	Zheng et al., 2011^[^^46^^]^
*CaWRKY34*	*CaWRKY6*	KF736800.1	*Ralstonia solanacearum* resistance High temperature and humidity tolerance	Cai et al., 2015^[^^39^^]^
*CaWRKY28*	*CaWRKY2*	DQ402421.1	Incompatible plant pathogens	Oh et al., 2006^[^^21^^]^

a, The unify IDs is used in this study. TMV, Tobacco mosaic virus.

b, Inconsistent nomenclature for pepper *WRKY* genes exists in the literature.

### Alternative splicing (AS) and interaction among CaWRKY proteins

Alternative splicing (AS) is an important post-transcriptional regulatory mechanism and has been reported to be related to virus infections in many plants [40]. In this study, we identified that 17 *CaWRKYs* have different types of AS events under CMV inoculation, including intron retention and exon skipping (S4 Fig and S7 Table). Most (41.18%) of these AS events were the form of intron retention (S7 Table).

**Fig 9:**
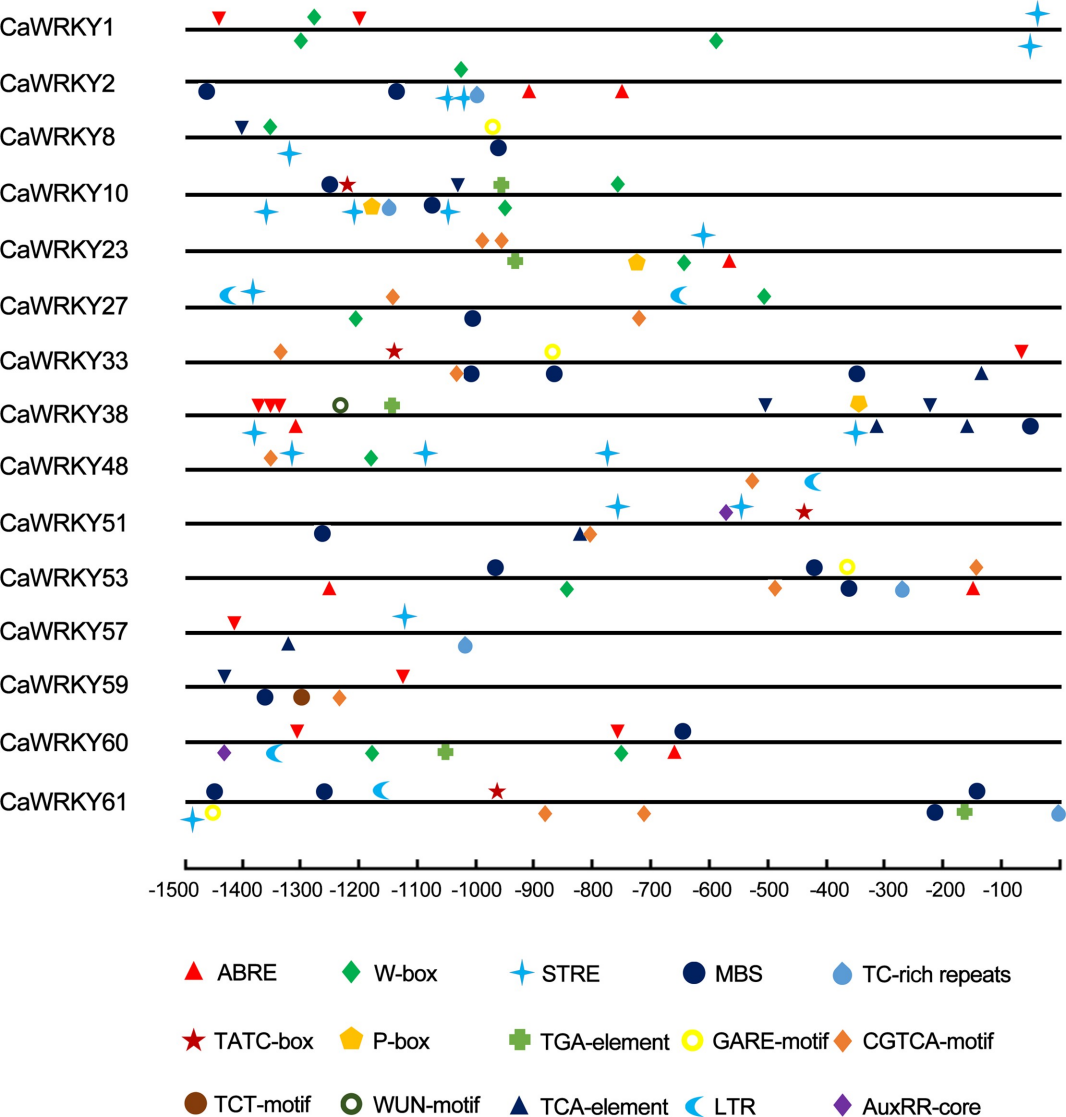
Predicted *cis*-elements in the promoter regions of *CaWRKY* genes. The promoter sequences (-1.5 kb) of 20 *CaWRKY* genes were analyzed. The *cis*-elements were indicated by different color symbols and placed in the relative position. Symbols above the line indicate the elements at the forward strand, while below the line indicate the reverse strand. ABRE (abscisic acid responsiveness), W-box defense response), CGTCA (MeJA-responsiveness), TCA (salicylic acid responsiveness), TC-rich repeats (defense and stress responsiveness), MBS (MYB binding site involved in drought induction), WUN (wound-responsive element), GARE (gibberellins-responsive element), and LTR (low temperature-responsive element).

*Cis*-element analysis of the promoter regions of the *CaWRKYs* also showed that many stress associated motifs were found in these *CaWRKY* genes, including ABRE, W-box, TC-rich repeats, TCA, LTR and so on (Fig 9 and S8 Table). The *CaWRKY38* has been predicted the most number of *cis*-elements (15), while *CaWRKY57* has only 4 *cis*-elements. Most promoter regions of these *CaWRKYs* contained ABRE, STRE, W box, and myb-binding site (MBS) motif, indicating their roles in abscisic acid, virus infection and drought responses [41].

**Fig 10:**
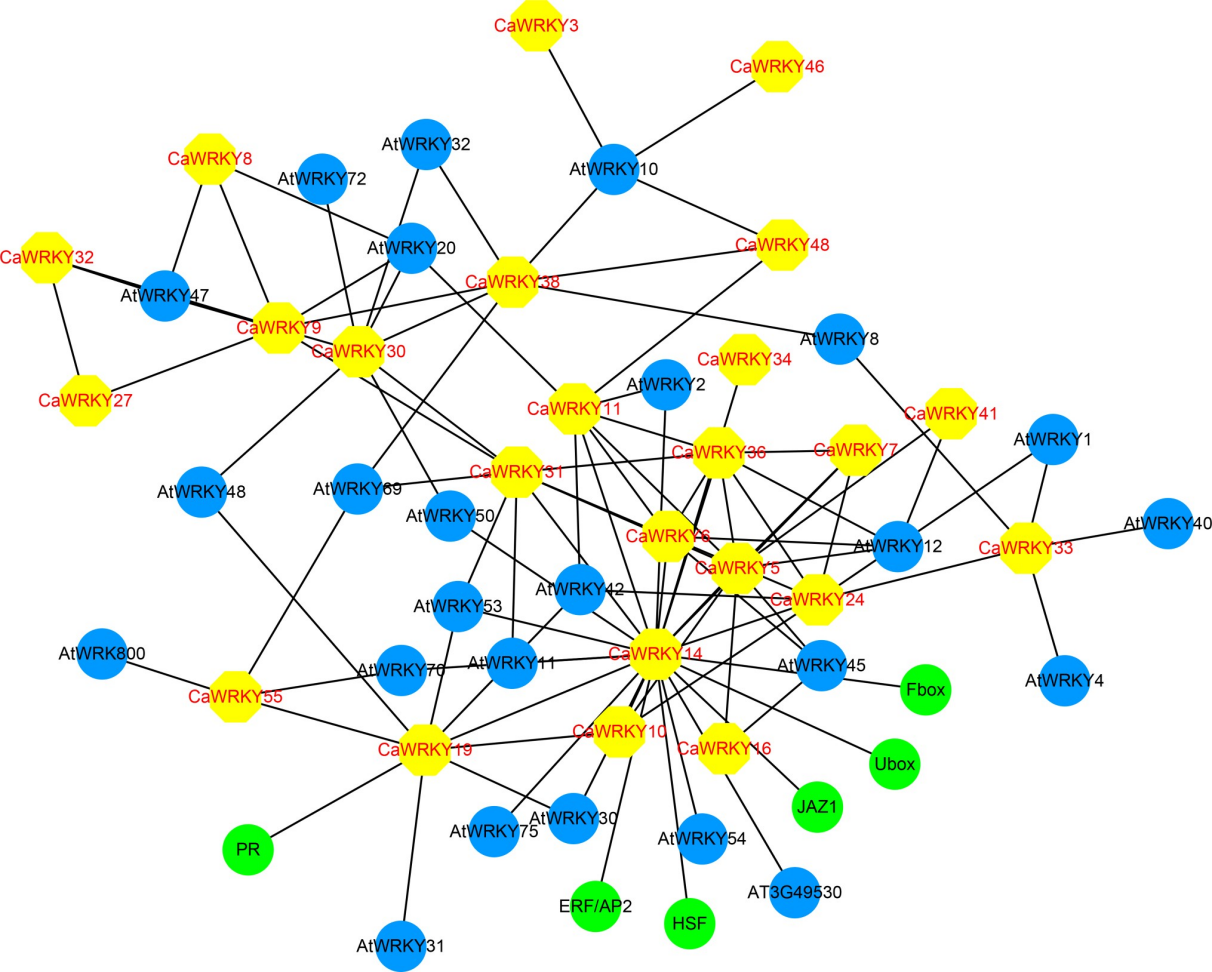
Interaction network of 24 *CaWRKY* genes identified in hot pepper and related genes in *Arabidopsis*.

Subsequently, the CaWRKY proteins potentially involved in plant stress responses were analyzed for protein-protein interaction using STRING database. Twenty-four CaWRKY proteins were formed interaction with other CaWRKYs or stress responsive genes, including PR, JAZ1, and HSF (Fig 10). Particularly, CaWRKY14 has been predicted to interact with 10 other CaWRKY proteins and 8 AtWRKYs from *Arabidopsis* (Fig 10).

## Discussion

### The *WRKY* genes in hot pepper

Whole-genome sequencing and assembly has greatly promoted the accurate annotation of the pepper gene families [17, 30]. In this study, we identified a total of 62 WRKY transcription factors from 34,476 pepper annotated genes in the hot pepper genome. Compared with the number genes of *Arabidopsis* (72; with genome size of 125 Mb) and rice (100; with genome size of 480 Mb), the size of the WRKY family in hot pepper (genome size of 3.48 Gb) is small, but closely related to species like cucumber (55; genome size of 367 Mb). A manual sequence check found three CaWRKY proteins (CaWRKY1, CaWRKY12, and CaWRKY58) that represent a deficient WRKY domain with the loss of the zinc-finger motif or the conserved heptapeptide WRKYGQK (Fig 4). In addition, 11 cDNA sequences that encoded *WRKY* genes were downloaded from the NCBI and used to search for new *WRKY* genes in pepper. Interestingly, all 11 genes were in our annotation for *CaWRKY* genes (S2 Table). However, inconsistent nomenclature for pepper *WRKY* genes exists in the literature, such as in *CaWRKYa* [42]. To unify the terminology, we recommend a novel nomenclature and renamed the *WRKY* genes on the basis of the previously described WRKY-encoding genes [11]. Three WRKY proteins (CaWRKY1, CaWRKY12, and CaWRKY58) that contain incomplete WRKY domains were named. All 62 genes were numbered according to the order of the chromosomes (S2 Table and Fig 1). The lengths of ORF sequences ranging from 414 bp to 2,610 bp imply a high degree of complexity among the *CaWRKY* genes.

The 62 *WRKY* genes in pepper can be divided into seven major groups 1, 2a–2e and 3, which are conserved in flowering plants [12, 27]. This conserved character suggests the wide distribution and biological functions for *WRKY* genes in flowering plants. Compared with the number *WRKY* genes in *Arabidopsis*, rice, and other plant species, the pepper *WRKY* genes did not significantly expand in history (Table 1). In the hot pepper variety CM334, nine major transcription factor families had fewer genes compared with other plant genomes, including the WRKY transcription factor families [17]. Previously, 73 *WRKY* genes were identified in the pepper variety CM334 [17]; we identified a relatively small number of *WRKY* genes in the pepper variety Zunla-1. One reason for this finding is the slight differences in the different versions of the pepper genome sequence. The other reason may be the varied number of identified WRKY pseudogenes because of the application of different gene prediction software parameters.

The heptapeptide WRKYGQK signature was highly conserved among pepper WRKY proteins, but six variations were identified as illustrated in Fig 2. Previous studies showed that CaWRKY1 (GenBank accession number EF468464) is a negative regulator of pathogen defense genes and contains the variant WRKYGKK rather than the heptapeptide WRKYGQK [20]. In other flowering plants, including *Arabidopsis*, rice and cucumber [6, 12, 27]. Variations in this region have also been reported, thereby suggesting that the variations in this region may be a common characteristic in WRKY proteins of the plant kingdom.

Compared with other plants, including *Arabidopsis*, rice, cotton, poplar, and cabbage, group 2a *WRKY* genes were much fewer than those of the other subgroups (Table 1). In flowering plants, group 2a genes are the subgroup with the smallest number of members and appear to play many important roles in response to biotic and abiotic stress [2]. In a recent report, Rinerson et al. (2015) suggested that group 2a genes are the last to evolve and appear to have arisen from group 2b genes on the basis of phylogenetic and comparative genomic studies [33].

To examine the evolutionary relationship among the CaWRKY proteins, an unrooted phylogenetic tree was constructed by using the neighbor-joining program. Our data showed that group 2 proteins are not monophyletic but form three distinct clades: 2a+2b, 2d+2e, and 2c (Fig 3). Moreover, groups 2a+2b, 2d+2e, and 2c are closely related to the group 1C domain, implying that the WRKY domain groups 2a+2b, 2d+2e, and 2c are evolutionarily close to the WRKY group 1C domain. Group 2 genes seem to have evolved from the group 1C domain. A hypothesis on the evolution of *WRKY* genes in plants have reported that proto-WRKY gene with a single WRKY domain underwent domain duplication to produce group 1 *WRKY* genes. Subsequent to the loss of the N-terminal WRKY domain formation in group 2 genes, group 3 genes are the last to evolve [2]. During domain analysis, we found that every *WRKY* gene in group 1 contained two WRKY domains, whereas in other plant species, a large number of *WRKY* genes with one WRKY domain in group 1 were identified, such as cucumber. Only 4 of the 12 group 1 *WRKY* genes contained only 1 WRKY domain. Domain acquisition and domain loss events appear to have shaped the WRKY family in rice [11]. The presence of 2 WRKY domains in group 1 implied that all these *CaWRKY* genes did not experience WRKY domain loss during the evolution process. Previous studies has proposed that genome duplication events resulted in the expansion of the *WRKY* genes in *Arabidopsis* and rice [2]. As compared with group 3 proteins in *Arabidopsis* (14 members) and rice (36 members), only 9 members are in group 3 for pepper. Therefore, the duplication events did not occur in the pepper WRKY family. On the basis of our phylogenetic analyses, hot pepper has four major WRKY transcription factor lineages: Groups 1+2c, 2a+2b, 2d+2e, and 3; Group 2a may be the youngest subgroup.

### WRKY protein roles in various biological processes

In plants, the WRKY proteins appear to be involved in the regulation of various processes [20] and play broad-spectrum regulatory roles as positive or negative regulators of plant defense regulation, abiotic stimuli, plant growth, and development, such as response to pathogen attack [43], plant hormone signaling [44], abiotic stresses [16], and plant development [45]. In the present study, many *CaWRKY* genes were differentially expressed under abiotic and biotic stresses (Figs 5–8). Our comparative transcriptome results and qRT-PCRs showed that *CaWRKY6* is expressed in all 14 organs at relatively high levels. The transcripts were dramatically induced after cold treatment and CMV infection (Fig 5C and Fig 8). The reported *CaWRKY30* (GenBank Number: FJ360844) was renamed to *CaWRKY6* in this study. The expression of *CaWRKY30* was induced by the application of various pathogens, phytohormones, and salicylic acid (Table 3) [46]. Therefore, the results indicated that *CaWRKY6* may play a role in the response to pathogen attack, the development of leaves and fruits, abiotic and biotic stress (Fig 8). In the plant signaling network, abscisic acid (ABA) represents a key signal to regulate plant growth and development [2]. Increasing evidence supports the important control of *WRKY* genes by ABA signaling. In our study, *cis*-elements analysis of the promoters showed that many *CaWRKY* genes have the ABRE motif (Fig 9 and S6 Table). Moreover, W-box motif was included in several *CaWRKYs* and some *CaWRKYs* have been reported to be directly bound to the W-box motifs of PR genes upon the pathogen infection [47]. In *Arabidopsis*, interaction of WRKY18 with WRKY40 and/or WRKY60 can affect the W-box binding activity, indicating that the *CaWRKY* genes containing W-box may be involved in the defense response [15].

Previous studies indicated that WRKY proteins are involved in the response to various abiotic stimuli. In *Arabidopsis*, the expression of 6 cold-inducible *WRKY* genes was enhanced in *icel*, a mutant defective in an upstream transcription factor required for chilling and freezing tolerance [48]. In our qRT-PCR experiments, 16 *WRKYs* (at least two genes in each group or subgroup) were selected and demonstrated that 15 *WRKYs* were up-regulated in response to cold stress, indicating that *CaWRKY* genes involved in response to cold exposure in hot pepper.

In the present study, the *CaWRKY* genes were also identified to be response to biotic stress, especially the virus infection (Figs 6–8). Previous studies have been reported 9 *CaWRKY* genes involved in many pathogen infection, such as TMV, *Xanthomonas campestris*, *Pseudomonas syringe*, *Ralstonia solanacearum* and so on (Table 3)[20, 42, 49]. Quantitative RT-PCRs validated most of the *CaWRKY* genes to be induced under CMV infection (Fig 8 and S3 Fig). *CaWRKY10*, *CaWRKY13* and *CaWRKY15* were up-regulated at all the stages under CMV inoculation (S3 Fig). The three *WRKY* genes have been reported to play roles in *Ralstonia solanacearum* resistance or TMV infection (Table 3) [8, 48, 50]. Overexpression of *CaWRKY10* (previous denoted as *CaWRKY27*) positively regulates the resistance response to *R*. *solanacearum*, suggesting they might also be involved in CMV defense in hot pepper [51]. Alternative splicing (AS) is an effective mean for plant defenses [52]. In this study, we identified several *CaWRKY* genes having AS events using the SMRT and Illumina RNA-seq data (S7 Table) [35]. There are 8 *CaWRKY* genes with intron retention type AS event, including *CaWRKY45* (Capana09g001251) which has been reported to be involved in response to CMV infection [35]. The mRNAs with intron retention are inaccessible to ribosomes, serving as an effective way to regulate protein synthesis, which can meet the demand for infection damage repair.

## Conclusions

In this study, we identified a total of 62 hot pepper WRKY transcription factors and analyzed their phylogenetic relationships and conserved motifs. The expression profiles of *CaWRKY* genes in different tissues under normal growth conditions were analyzed based on 14 transcriptome databases. A total of 35 putative miRNA target sites were predicted in 20 *CaWRKYs*. Furthermore, the expression levels of 16 selected WRKY transcripts were identified after cold or CMV treatments. Alternative splicing events of some *CaWRKYs* were also identified under CMV infection. In addition, promoter analysis confirmed that *CaWRKY* genes are involved in response to biotic or abiotic stresses in hot pepper. The construction and expression of novel *CaWRKY* genes provide fundamental information for better understanding of the signaling pathways involved in the WRKY-mediated regulation of developmental processes, as well as biotic and abiotic stress responses.

## Supporting information

S1 Fig The details of 20 motifs in the protein sequences of CaWRKYs. (PDF)

S2 Fig Expression patterns of selected 16 WRKY genes under cold stress. For quantitative RT-PCR, the relative expression level was calculated by the description methods. The *β*-actin gene in pepper plants was used as an internal reference to normalize the data. The error bars were calculated based on quadruple replicates.
(PDF)

S3 Fig Expression patterns of selected 16 *WRKY* genes after CMV virus inoculation. For quantitative RT-PCR, the relative expression level was calculated by the description methods. The *β*-actin gene in pepper plants was used as an internal reference to normalize the data.
(PDF)

S4 Fig Structures and isoforms of the 10 *CaWRKY* genes with alternative splicing (AS) events. (PDF)

S1 Table Primers used for quantitative RT-PCR in the study. (XLSX)

S2 Table The *WRKY* genes identified in hot pepper. (PDF)

S3 Table The nucleotide and protein sequences of 62 *CaWRKY* gene family. (PDF)

S4 Table The expression patterns of *CaWRKY* genes under abiotic and biotic stress in pepper leaves. (XLSX)

S5 Table The homologue genes of *CaWRKY* in tomato (*Solanum lycopersicum*). (XLSX)

S6 Table Predicted *CaWRKY* genes as the targets of some miRNAs in pepper. (XLSX)

S7 Table Details information of the alternative splicing (AS) events of *CaWRKYs* under CMV infection. (XLSX)

S8 Table Predicted *cis*-elements in the promoter regions of 15 *CaWRKY* genes of pepper. (XLSX)
