# Supplementary material for: Identification, expression, alternative splicing and functional analysis of pepper WRKY gene family in response to biotic and abiotic stresses
Source: PLoS One. 2019 Jul 22;14(7):e0219775. doi: 10.1371/journal.pone.0219775 (PMC6645504; doi:10.1371/journal.pone.0219775)

S1 Fig. The details of 20 motifs in the protein sequences of CaWRKYs

Motif1

Width= 27, sites=73, llr=3861, E-value=3.9e-1207

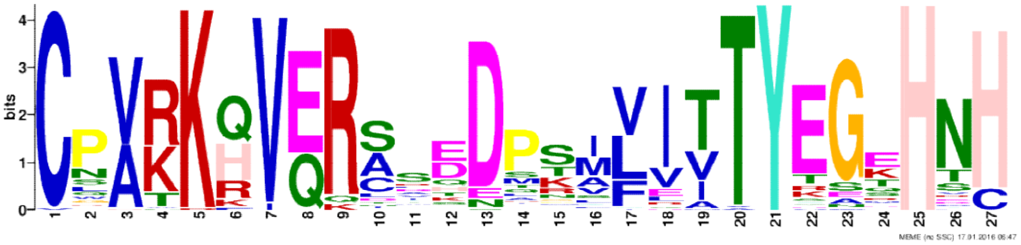

Motif2

Width= 20, sites=76, llr=3285, E-value=1.0e-1-27

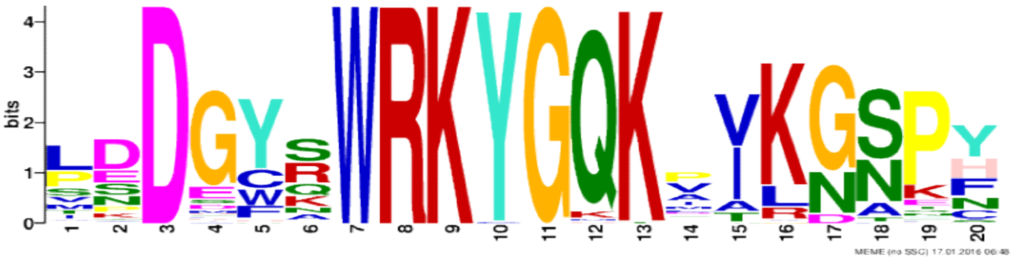

Motif3

Width=11, sites=74, llr=1707, E-value=7.8e-439

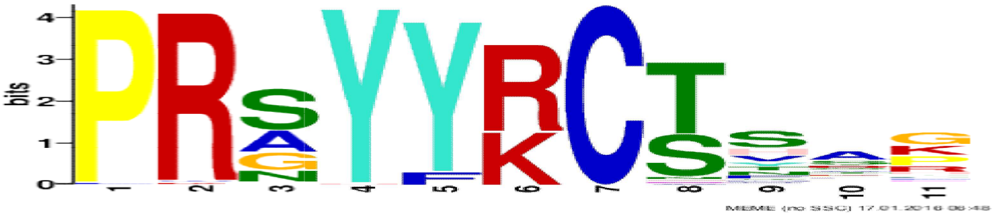

Motif4

Width= 32, sites=11, llr=917, E-value=4.4e-209

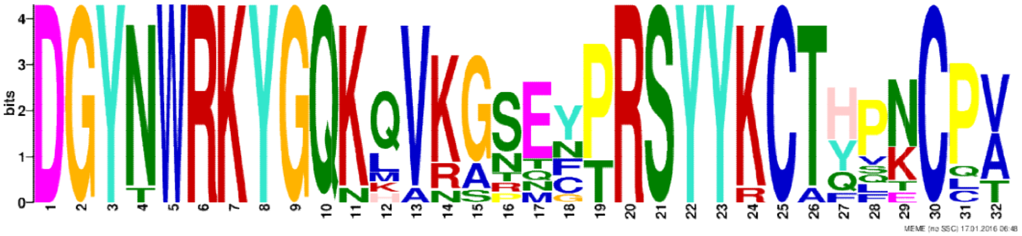

Motif5

Width=21, sites=29, llr=1022, E-value=2.3e-287

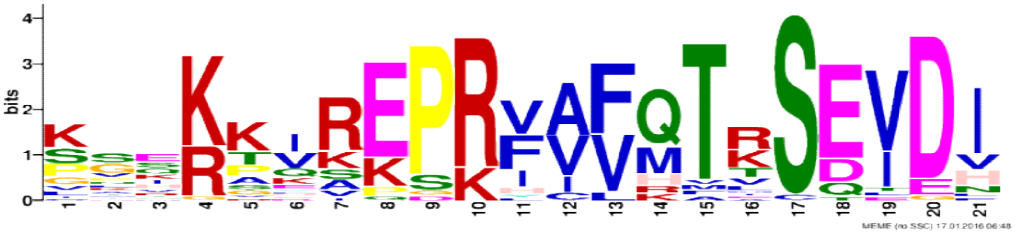

Motif6

Width= 28, sites=16, llr=969, E-value=1.8e-201

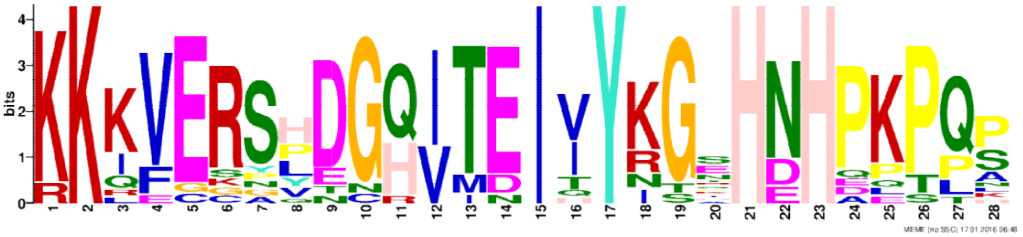

Motif7

Width=39, sites=12, llr=847, E-value=3.3e-109

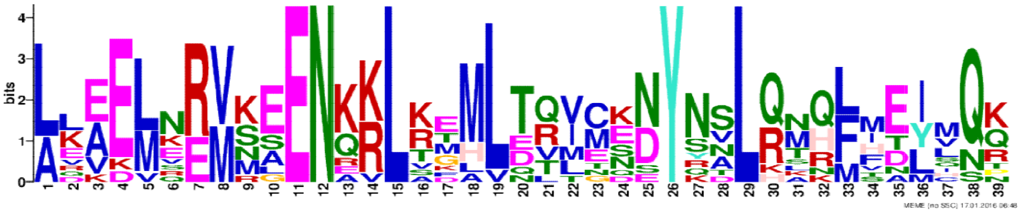

Motif8

Width=15, sites=23, llr=580, E-value=1.2e-062

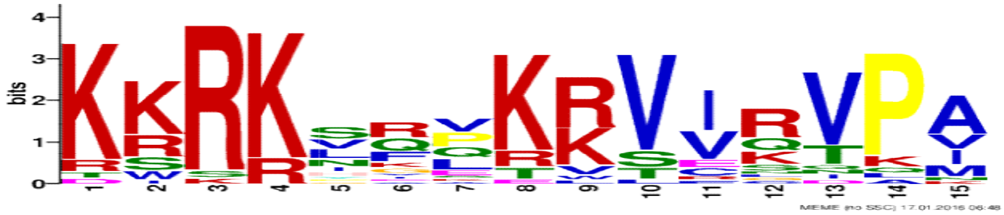

Motif9

Width=31, sites=11, llr=634, E-value=1.4e-070

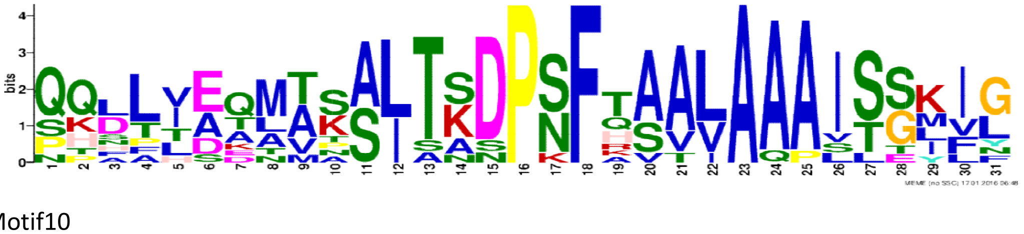

Motif10

Width=27, sites=8, llr=495, E-value=6.8e-069

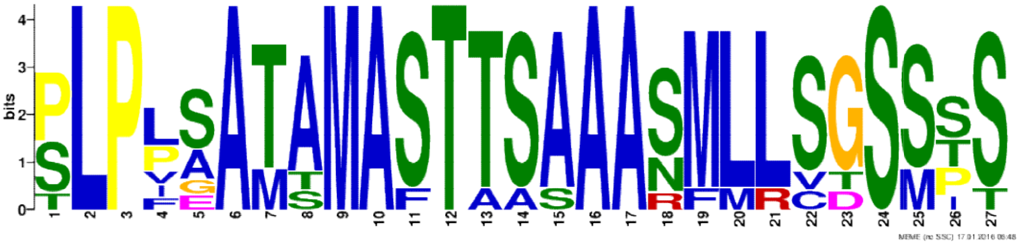

Motif11

Width=40, sites=6, llr=535, E-value=1.1e-055

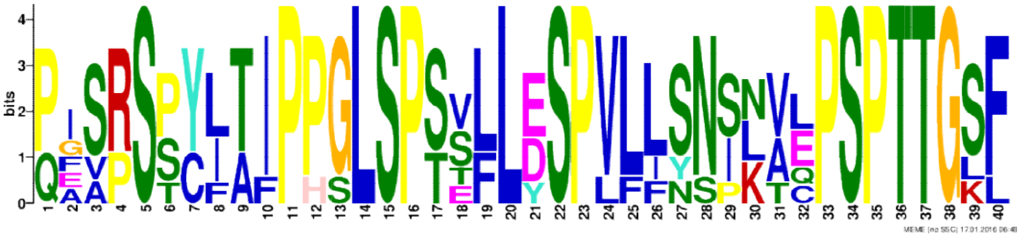

Motif12

Width=35, sites=8, llr=552, E-value=7.8e-052

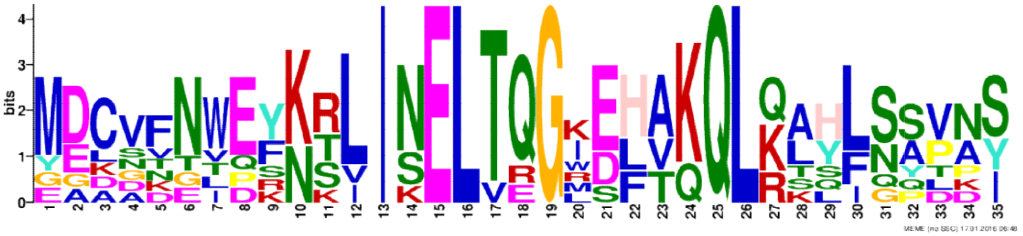

Motif13

Width=15, sites=7, llr=273, E-value=6.8e-034

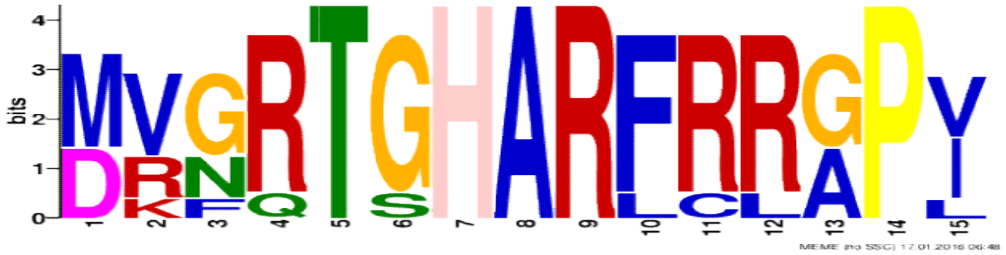

Motif14

Width=50, sites=4, llr=481, E-value=1.0e-037

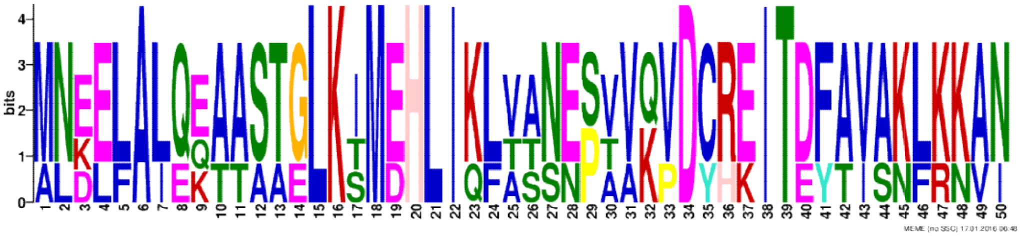

Motif15

Width=50, sites=4, llr=482, E-value=1.3e-037

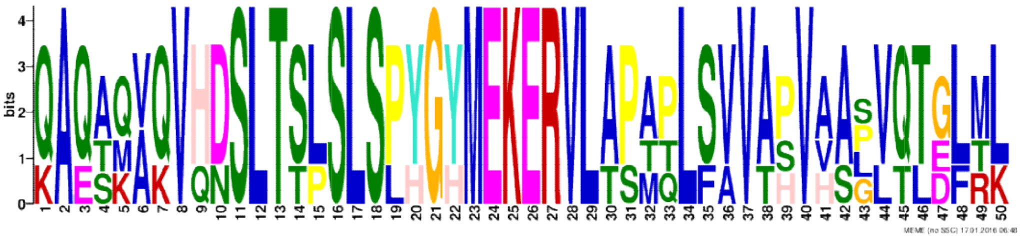

Motif16

Width=20, sites=11, llr=404, E-value=8.4e-029

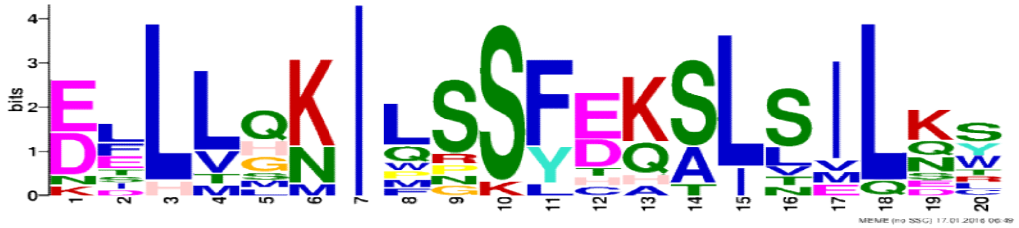

Motif17

Width=15, sites=8, llr=272, E-value=2.5e-022

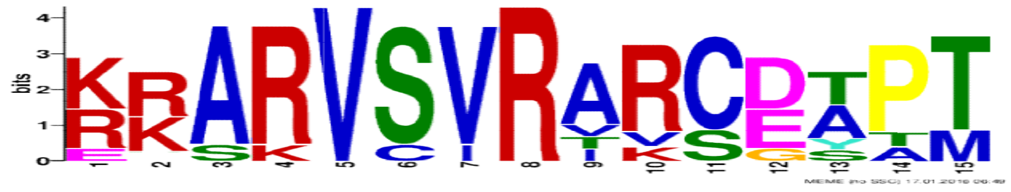

Motif18

Width=15, sites=5, llr=206, E-value=1.6e-021

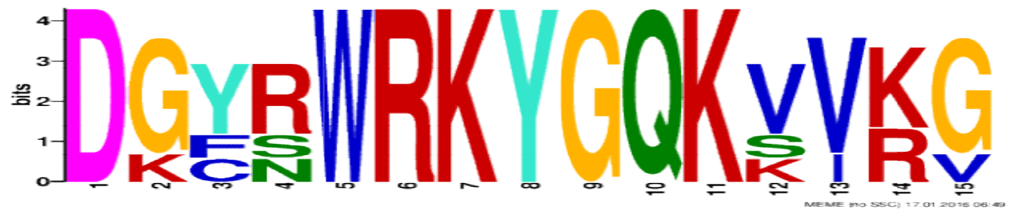

Motif19

Width=29, sites=4, llr=291, E-value=1.7e-021

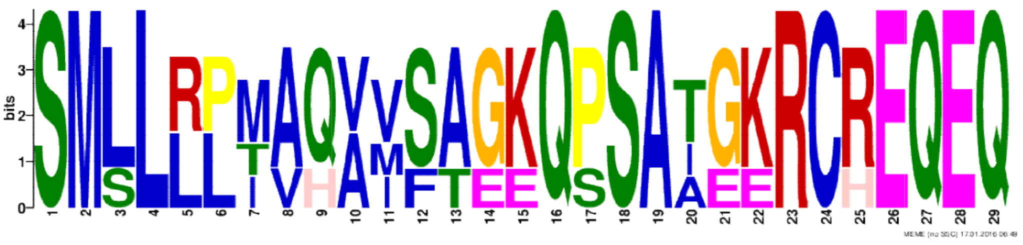

Motif20

Width=50, sites=2, llr=299, E-value=2.4e-021

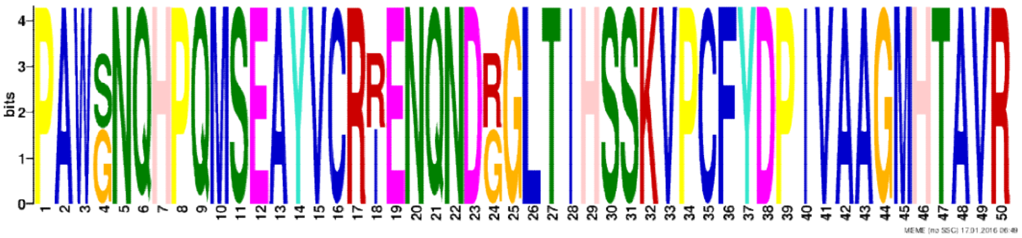

Supplement: S1 Fig — (PDF) [file pone.0219775.s001.pdf]
