# Supplementary material for: Identification, expression, alternative splicing and functional analysis of pepper WRKY gene family in response to biotic and abiotic stresses
Source: PLoS One. 2019 Jul 22;14(7):e0219775. doi: 10.1371/journal.pone.0219775 (PMC6645504; doi:10.1371/journal.pone.0219775)
Supplement: S2 Fig — For quantitative RT-PCR, the relative expression level was calculated by the description methods. The β-actin gene in pepper plants was used as an internal reference to normalize the data. The error bars were calculated based on quadruple replicates. (PDF) [file pone.0219775.s002.pdf]

S2 Fig. Expression patterns of selected 16 WRKY genes under cold stress. For quantitative RT-PCR, the relative expression level was calculated by the description methods. The *β*-actin gene in pepper plants was used as an internal reference to normalize the data. The error bars were calculated based on quadruple replicates.

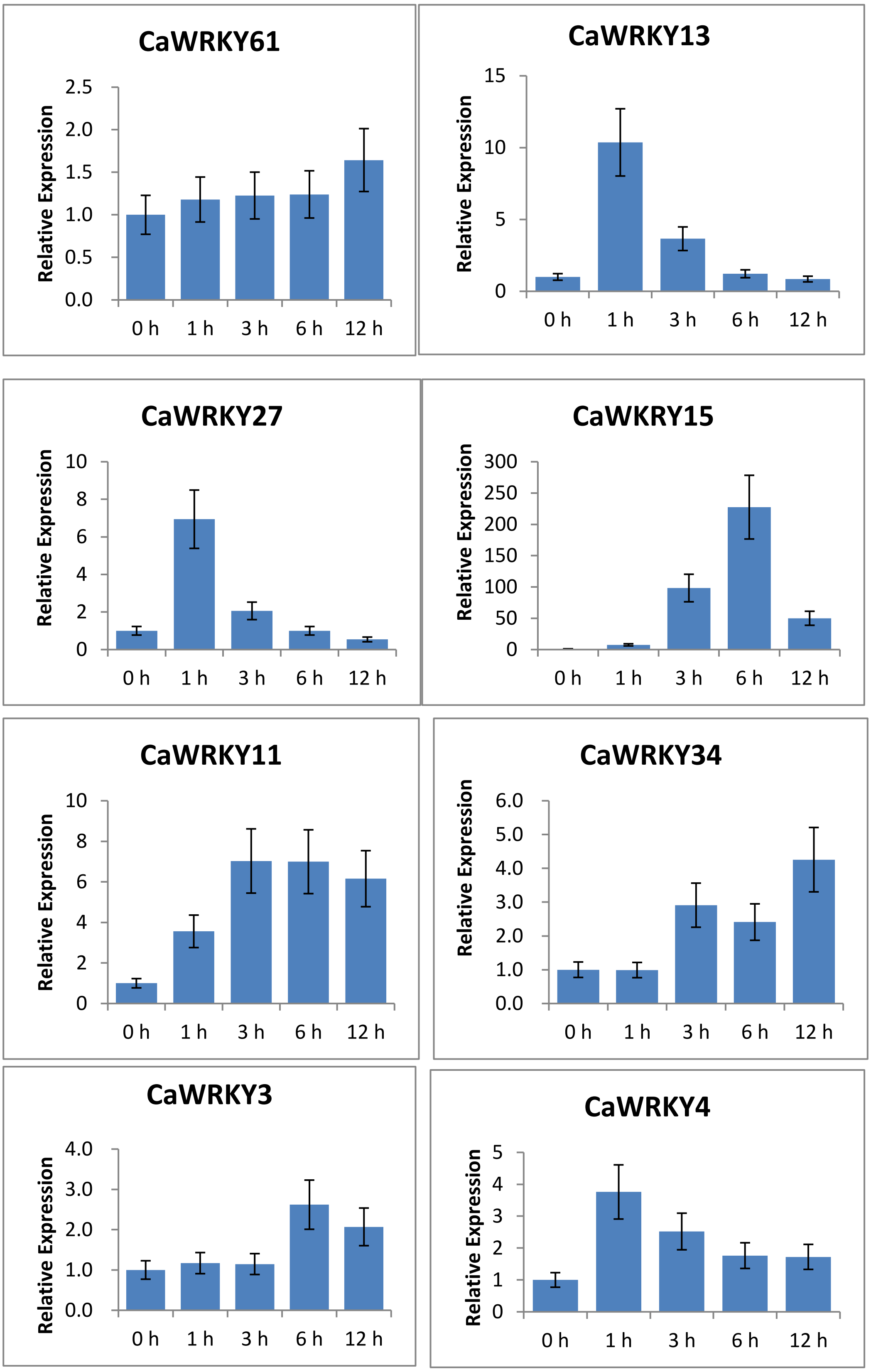

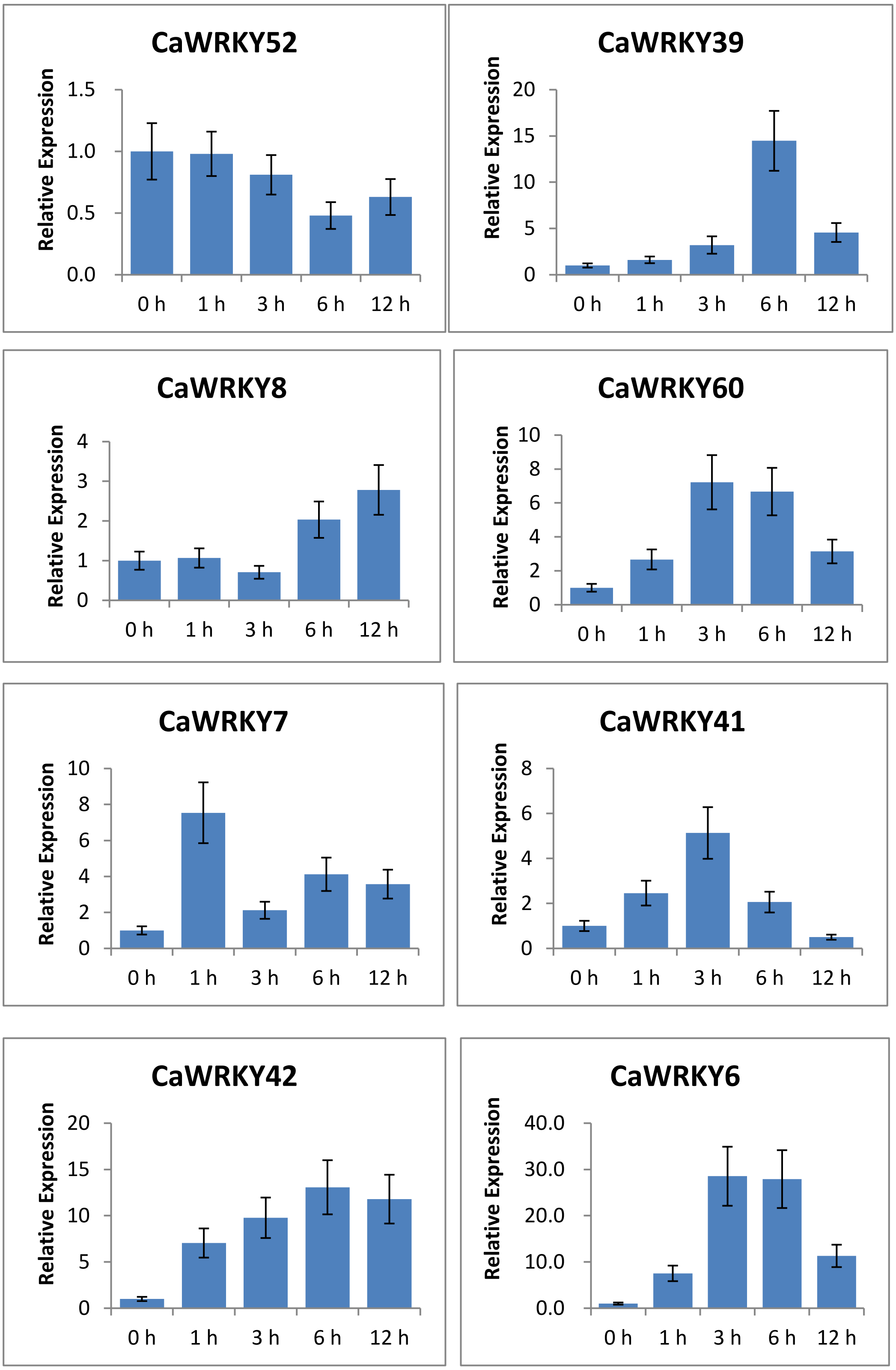
CaWRKY52
Relative Expression
0.0
0.5
1.0
1.5
0 h
1 h
3 h
6 h
12 h
CaWRKY39
Relative Expression
0
5
10
15
20
0 h
1 h
3 h
6 h
12 h
CaWRKY8
Relative Expression
0
1
2
3
4
0 h
1 h
3 h
6 h
12 h
CaWRKY60
Relative Expression
0
2
4
6
8
10
0 h
1 h
3 h
6 h
12 h
CaWRKY7
Relative Expression
0
2
4
6
8
10
0 h
1 h
3 h
6 h
12 h
CaWRKY41
Relative Expression
0
2
4
6
8
0 h
1 h
3 h
6 h
12 h
CaWRKY42
Relative Expression
0
5
10
15
20
0 h
1 h
3 h
6 h
12 h
CaWRKY6
Relative Expression
0.0
10.0
20.0
30.0
40.0
0 h
1 h
3 h
6 h
12 h
