# Supplementary material for: Identification, expression, alternative splicing and functional analysis of pepper WRKY gene family in response to biotic and abiotic stresses
Source: PLoS One. 2019 Jul 22;14(7):e0219775. doi: 10.1371/journal.pone.0219775 (PMC6645504; doi:10.1371/journal.pone.0219775)
Supplement: S3 Fig — For quantitative RT-PCR, the relative expression level was calculated by the description methods. The β-actin gene in pepper plants was used as an internal reference to normalize the data. (PDF) [file pone.0219775.s003.pdf]

S3 Fig. Expression patterns of selected 16 WRKY genes after CMV virus inoculation. For quantitative RT-PCR, the relative expression level was calculated by the description methods. The β-actin gene in pepper plants was used as an internal reference to normalize the data.

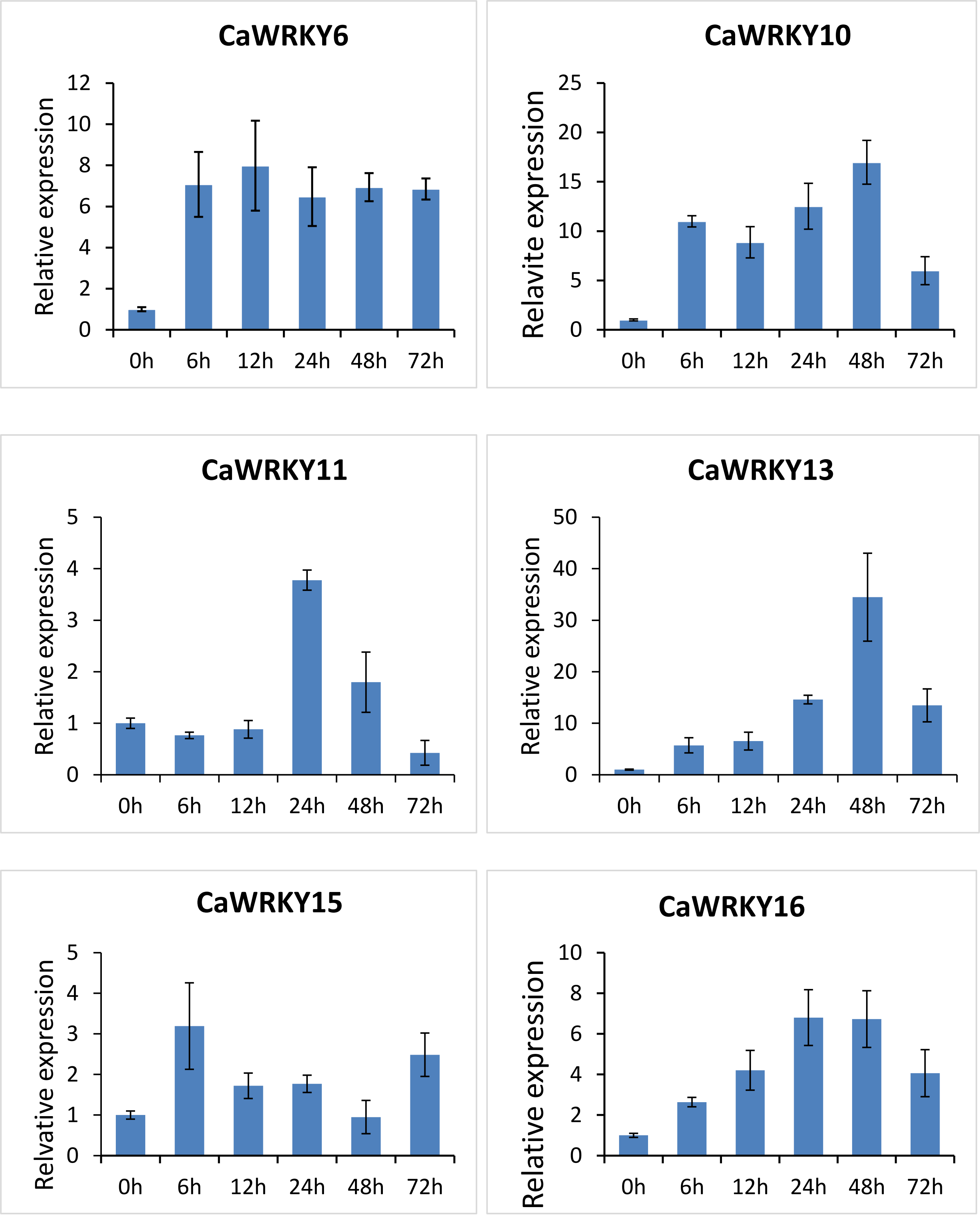

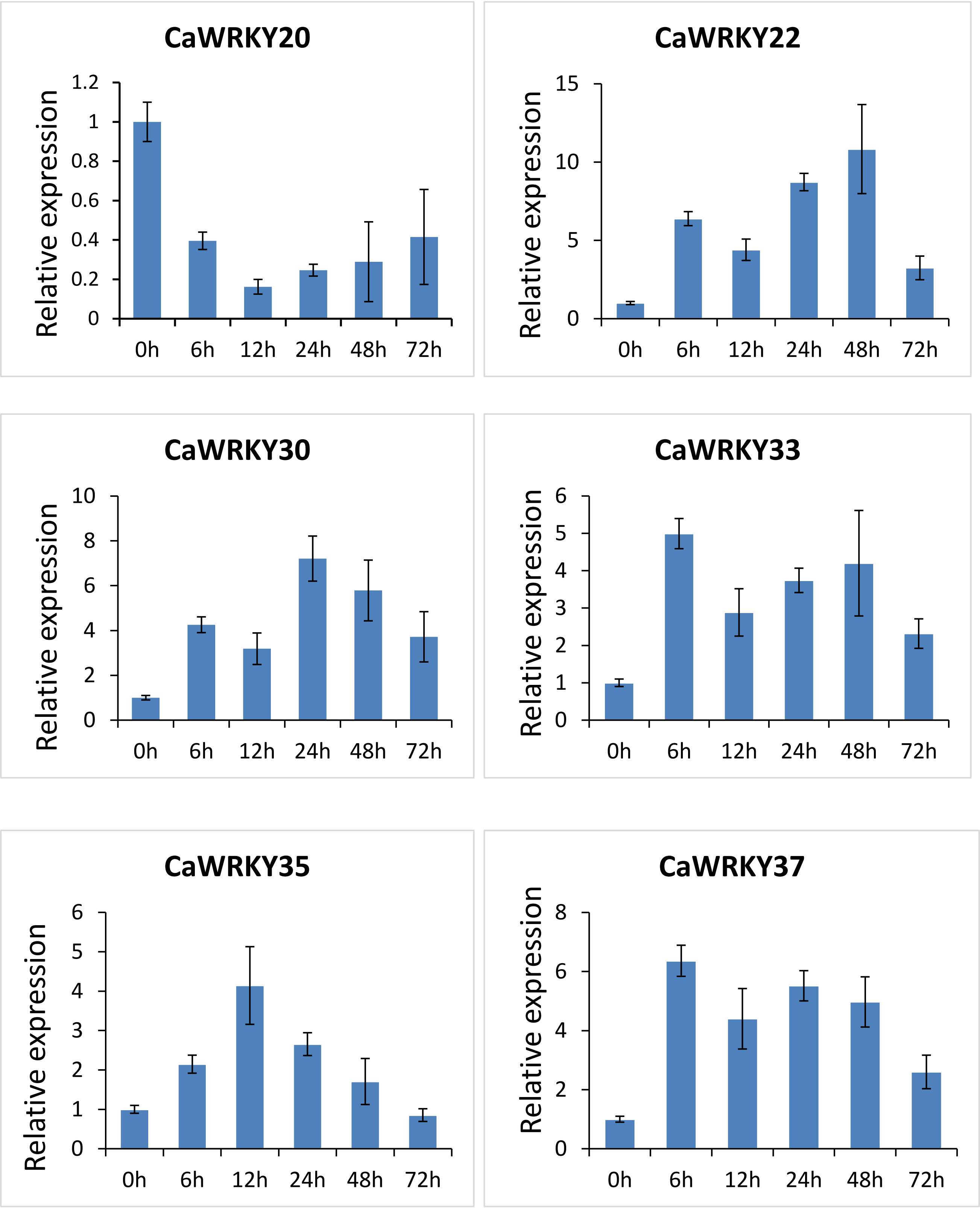

CaWRKY20
Relative expression
1.2
1
0.8
0.6
0.4
0.2
0
0h 6h 12h 24h 48h 72h
CaWRKY22
Relative expression
15
10
5
0
0h 6h 12h 24h 48h 72h
CaWRKY30
Relative expression
10
8
6
4
2
0
0h 6h 12h 24h 48h 72h
CaWRKY33
Relative expression
6
5
4
3
2
1
0
0h 6h 12h 24h 48h 72h
CaWRKY35
Relative expression
6
5
4
3
2
1
0
0h 6h 12h 24h 48h 72h
CaWRKY37
Relative expression
8
6
4
2
0
0h 6h 12h 24h 48h 72h

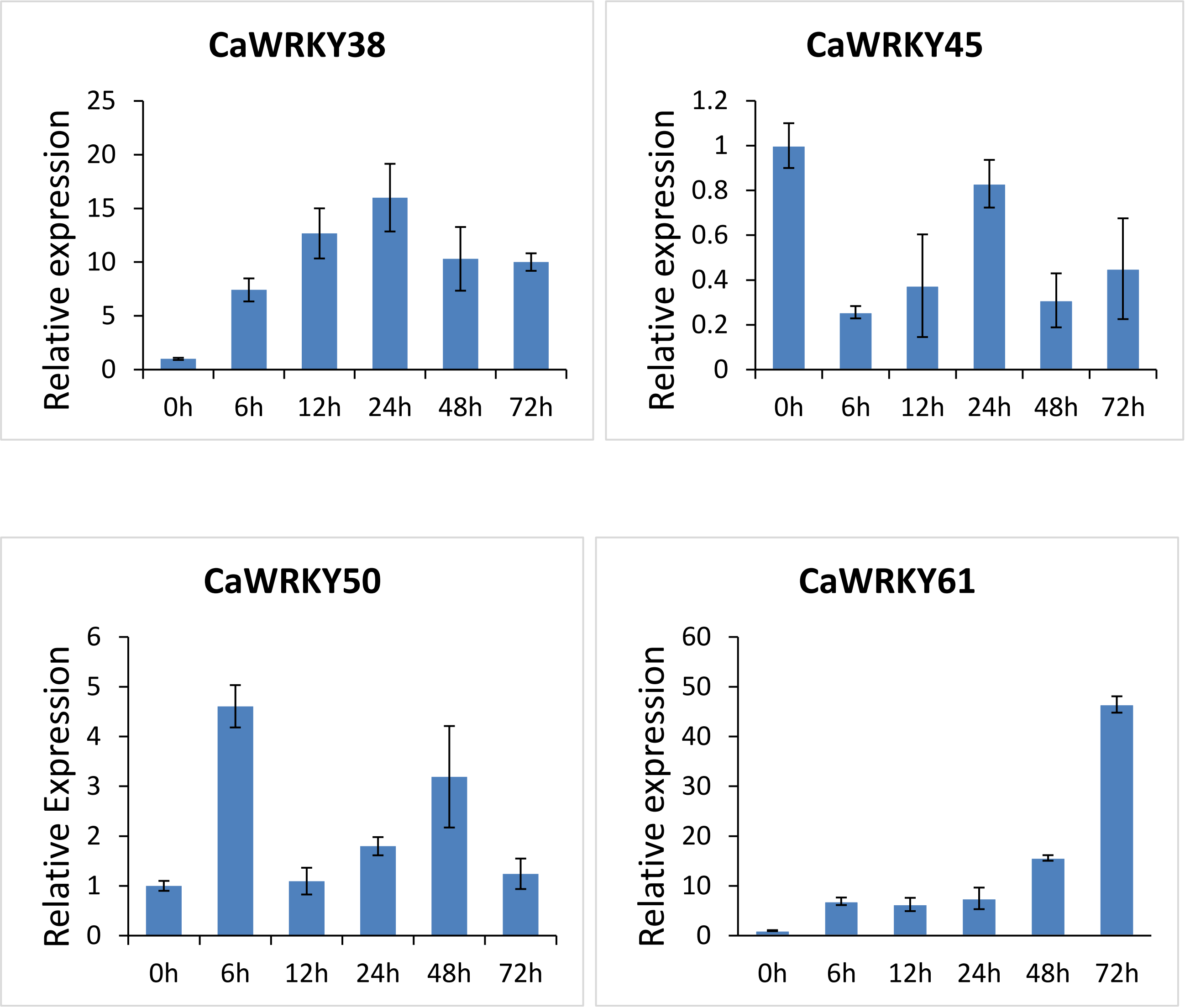
CaWRKY38
Relative expression
25
20
15
10
5
0
0h
6h
12h
24h
48h
72h
CaWRKY45
Relative expression
1.2
1
0.8
0.6
0.4
0.2
0
0h
6h
12h
24h
48h
72h
CaWRKY50
Relative Expression
6
5
4
3
2
1
0
0h
6h
12h
24h
48h
72h
CaWRKY61
Relative expression
60
50
40
30
20
10
0
0h
6h
12h
24h
48h
72h
