# Supplementary material for: Identification, expression, alternative splicing and functional analysis of pepper WRKY gene family in response to biotic and abiotic stresses
Source: PLoS One. 2019 Jul 22;14(7):e0219775. doi: 10.1371/journal.pone.0219775 (PMC6645504; doi:10.1371/journal.pone.0219775)
Supplement: S4 Fig — (PDF) [file pone.0219775.s004.pdf]

S4 Fig. Structures and isoforms of the 10 CaWRKY genes with alternative splicing (AS) events.

CaWRKY6

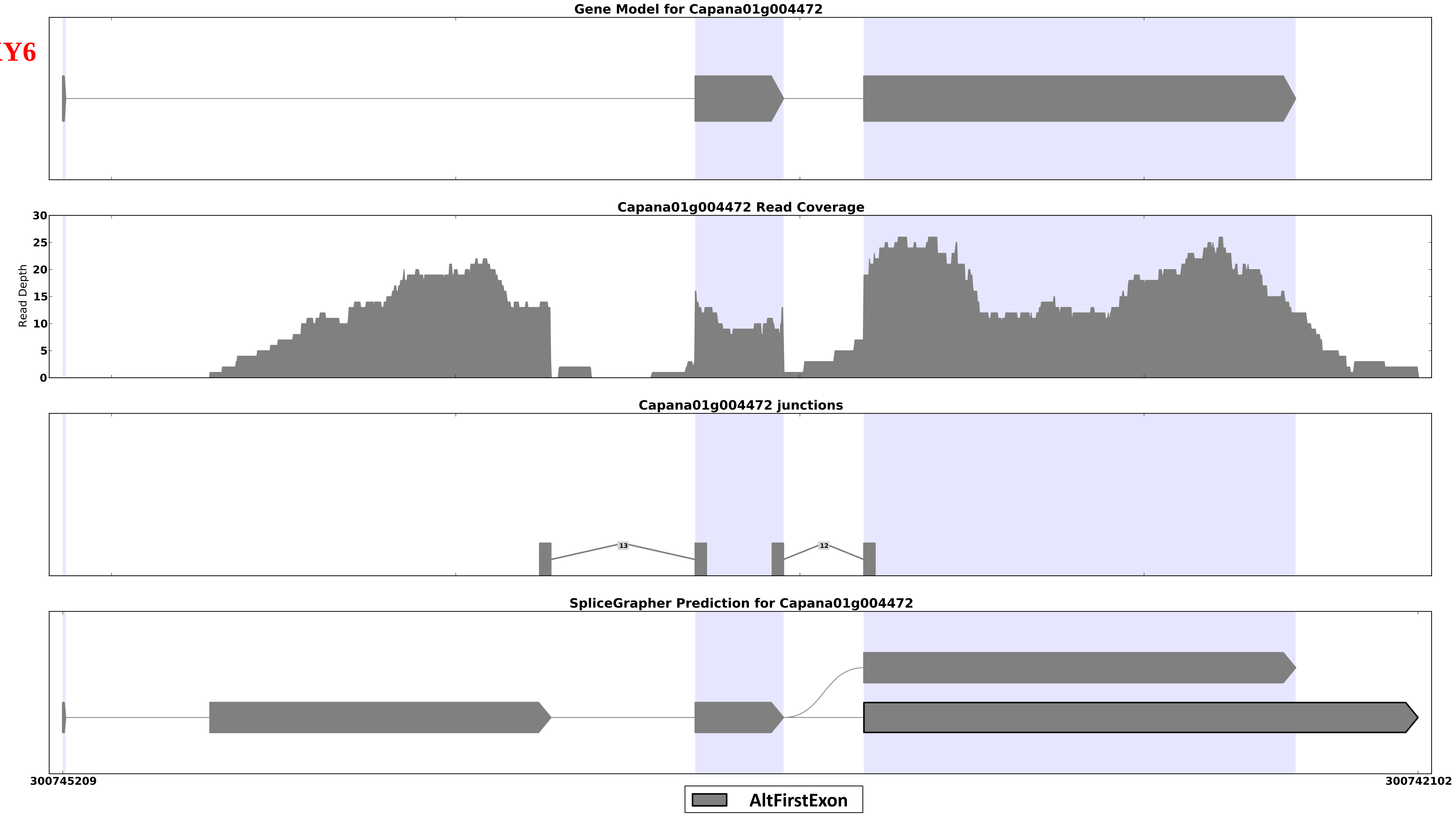

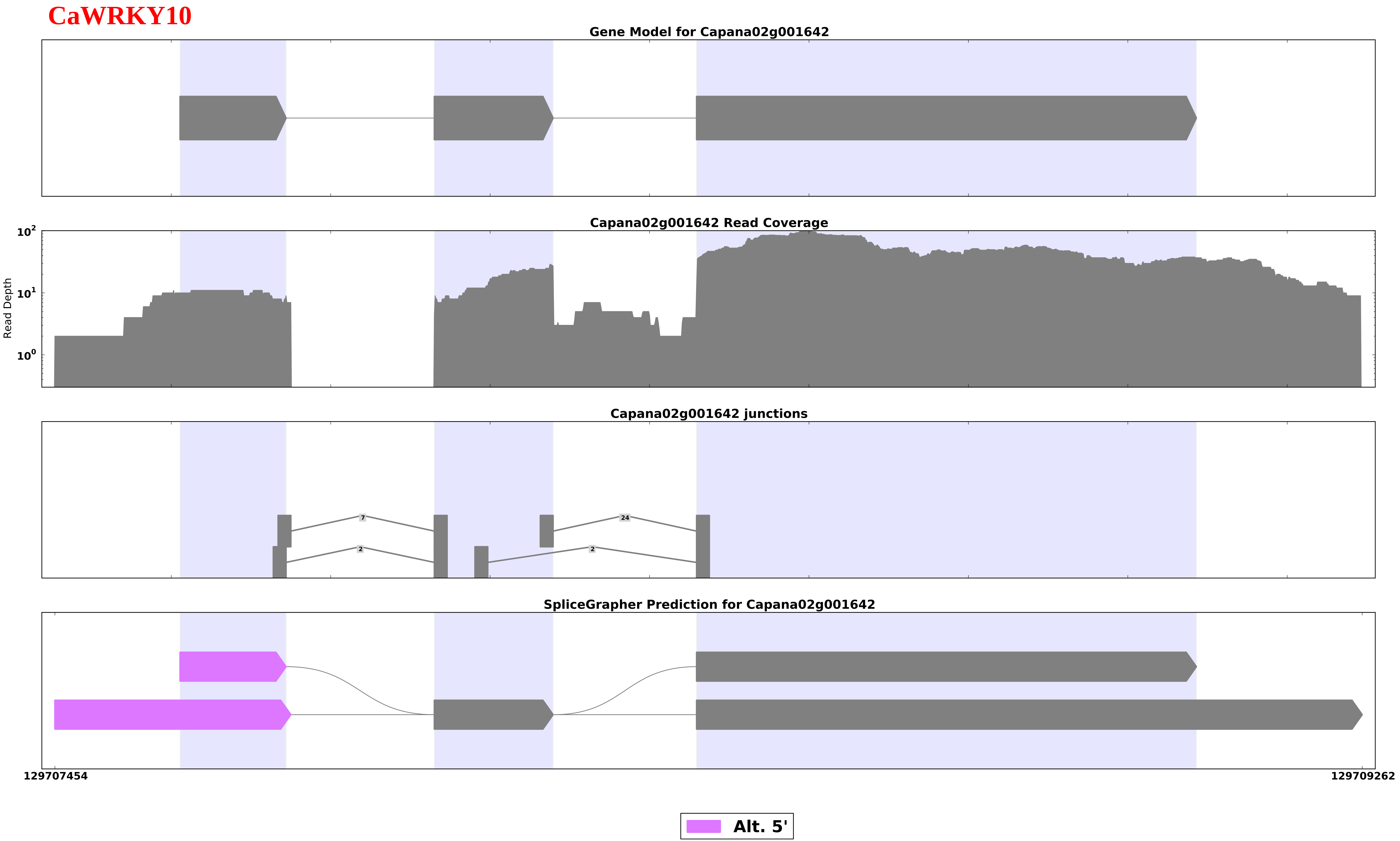
CaWRKY10
Gene Model for Capana02g001642
Capana02g001642 Read Coverage
Read Depth
10^2
10^1
10^0
Capana02g001642 junctions
7
24
2
2
SpliceGrapher Prediction for Capana02g001642
129707454
129709262
Alt. 5'

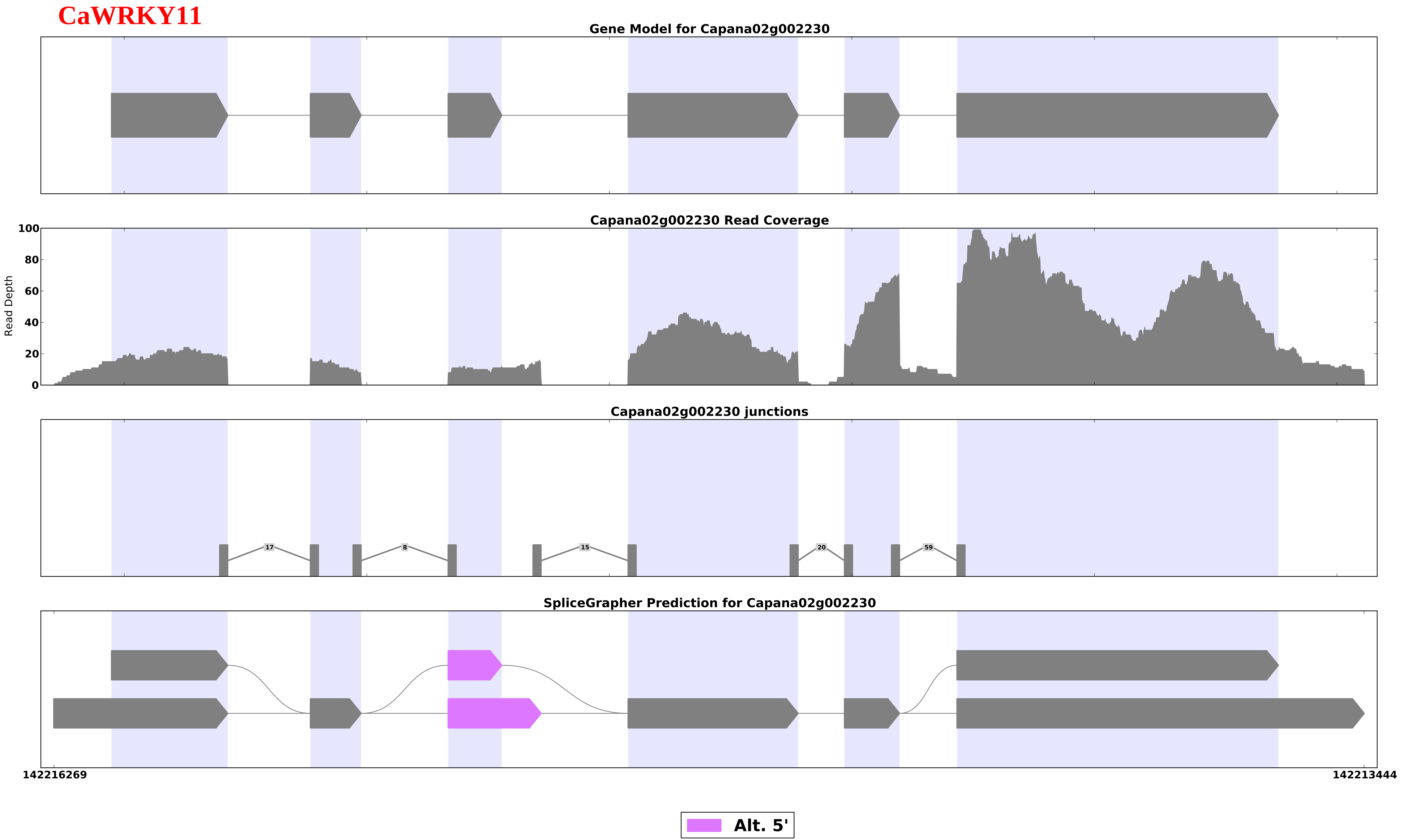
CaWRKY11
Gene Model for Capana02g002230
Capana02g002230 Read Coverage
Read Depth
100
80
60
40
20
0
Capana02g002230 junctions
17
8
15
20
59
SpliceGrapher Prediction for Capana02g002230
142216269
142213444
Alt. 5'

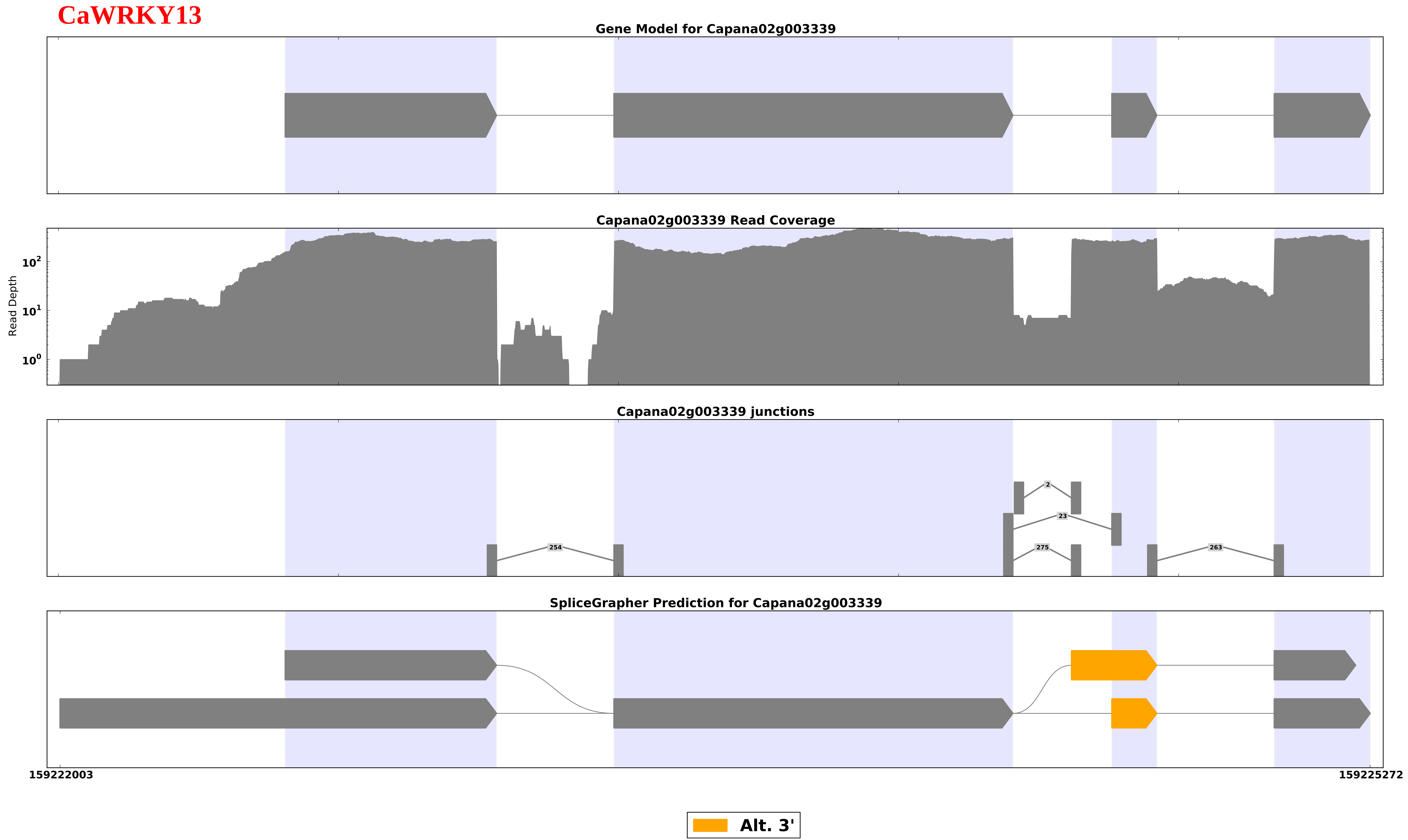

CaWRKY13
Gene Model for Capana02g003339
Capana02g003339 Read Coverage
Read Depth
10^2
10^1
10^0
Capana02g003339 junctions
2
23
254
275
263
SpliceGrapher Prediction for Capana02g003339
159222003
159225272
Alt. 3'

CaWRKY21

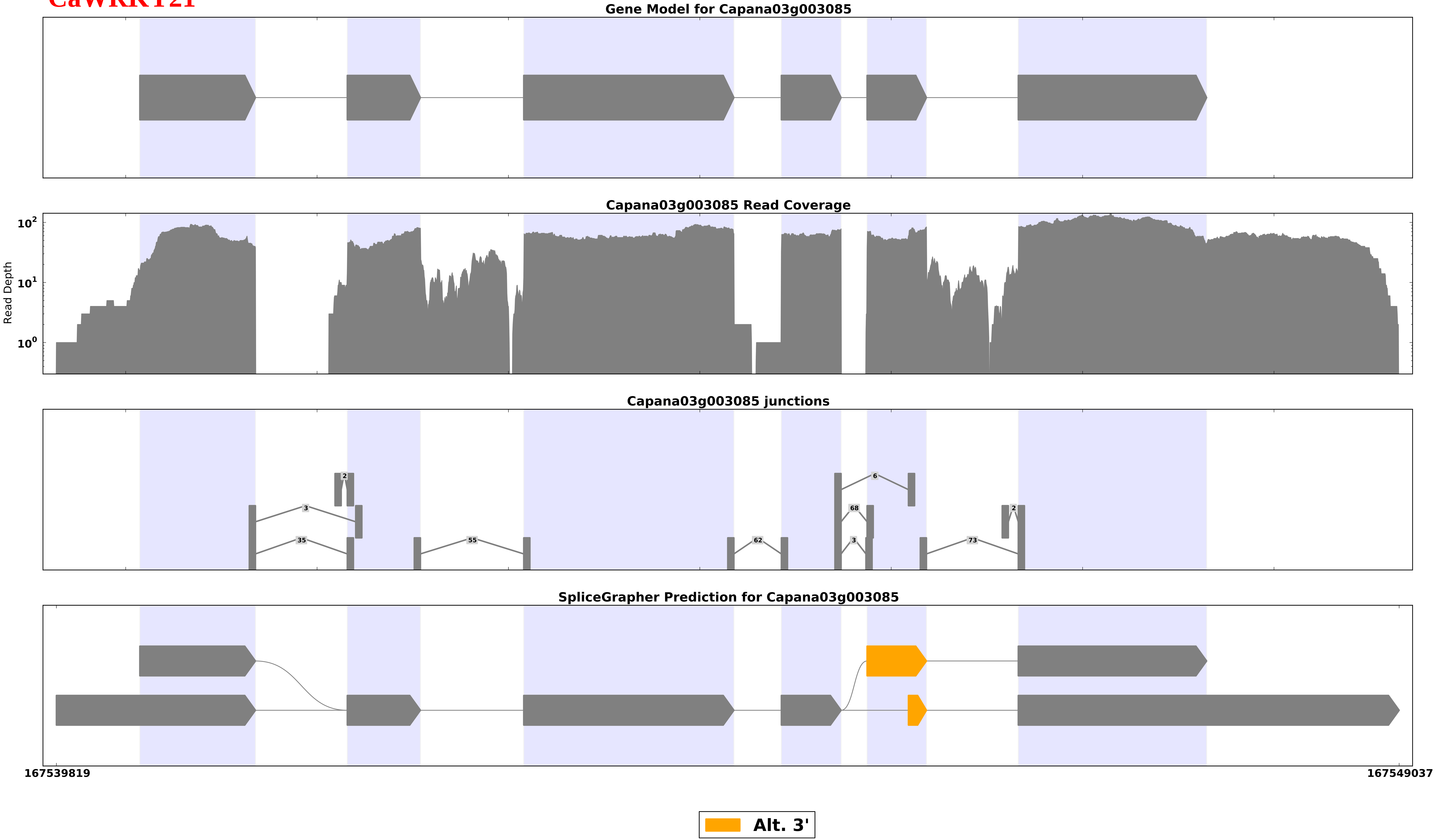
Gene Model for Capana03g003085
Capana03g003085 Read Coverage
Read Depth
10^2
10^1
10^0
Capana03g003085 junctions
2
3
35
55
62
6
68
3
2
73
SpliceGrapher Prediction for Capana03g003085
167539819
167549037
Alt. 3'

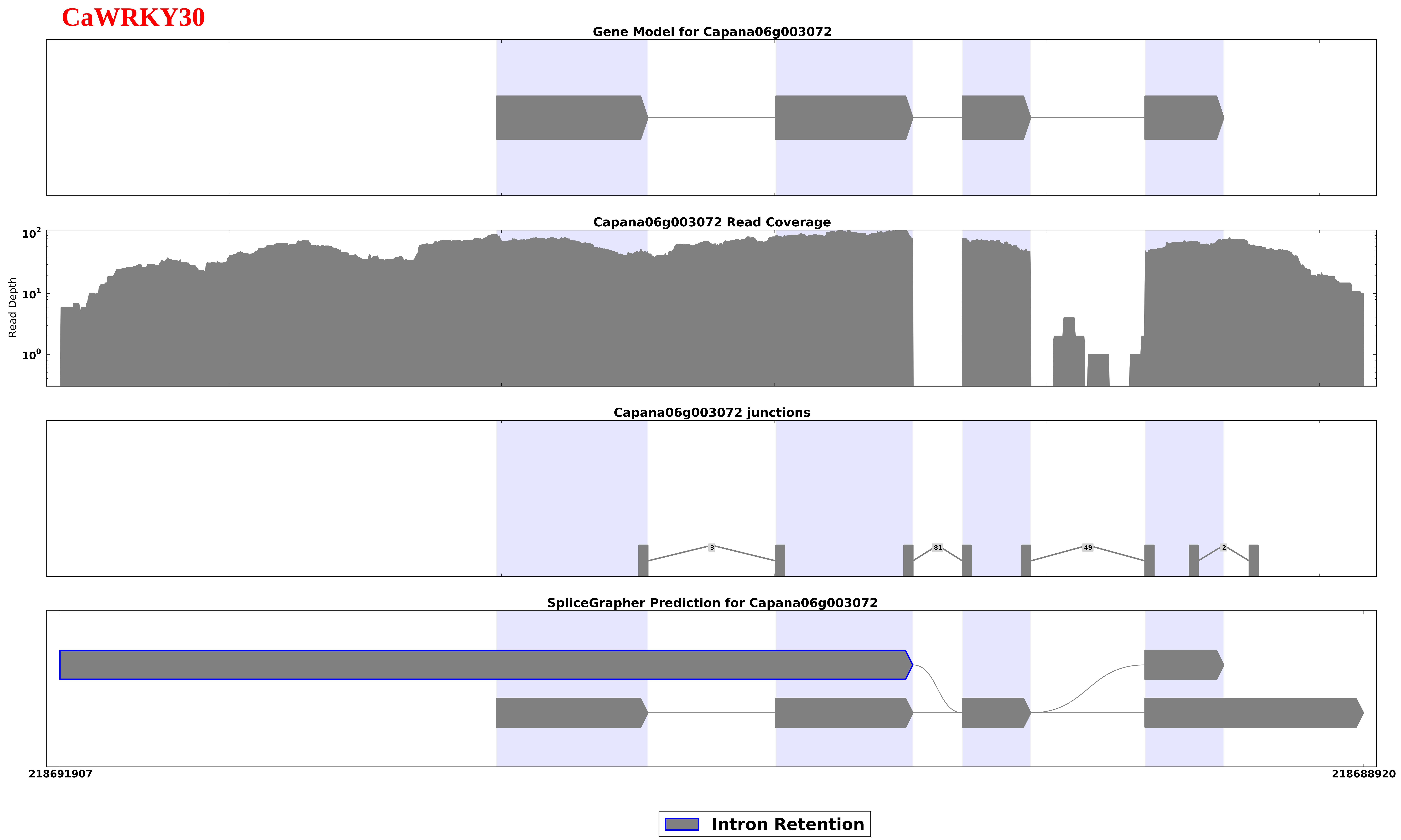
CaWRKY30
Gene Model for Capana06g003072
Capana06g003072 Read Coverage
Read Depth
10^2
10^1
10^0
Capana06g003072 junctions
3
81
49
2
SpliceGrapher Prediction for Capana06g003072
218691907
218688920
Intron Retention

CaWRKY31

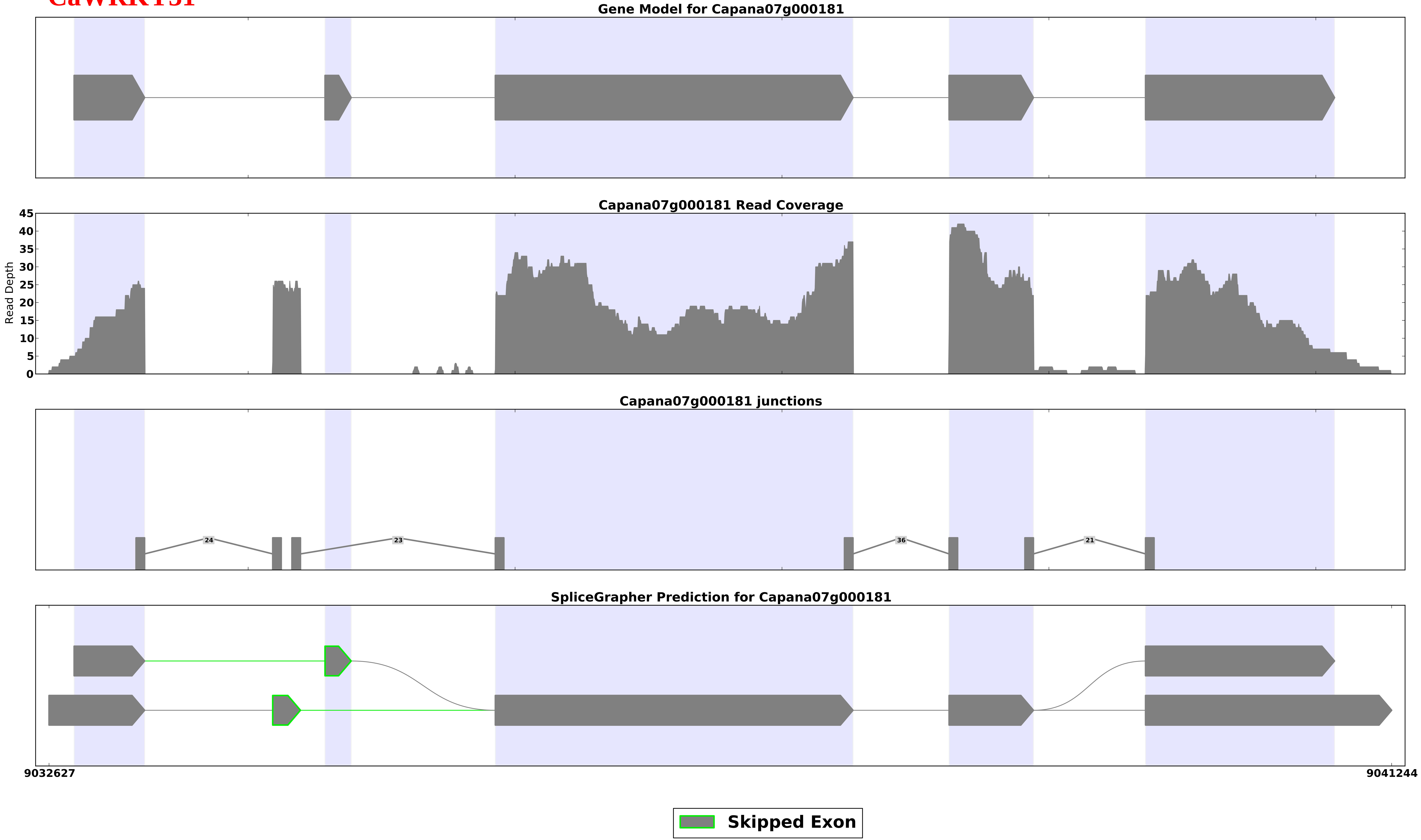
Gene Model for Capana07g000181
Capana07g000181 Read Coverage
Read Depth
45
40
35
30
25
20
15
10
5
0
Capana07g000181 junctions
24
23
36
21
SpliceGrapher Prediction for Capana07g000181
9032627
9041244
Skipped Exon

CaWRKY33

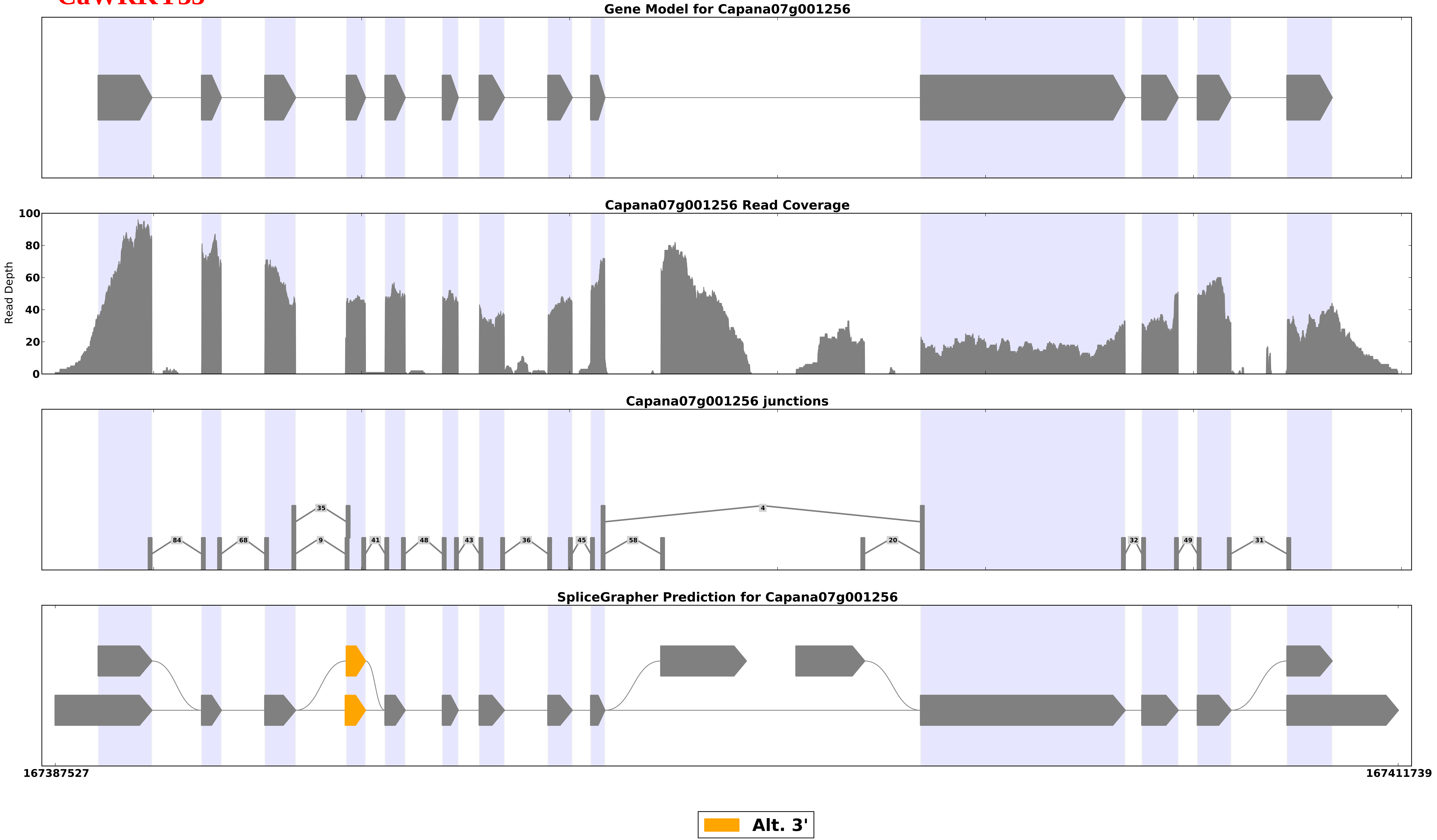
Gene Model for Capana07g001256
Capana07g001256 Read Coverage
Read Depth
100
80
60
40
20
0
Capana07g001256 junctions
84
68
35
9
41
48
43
36
45
58
4
20
32
49
31
SpliceGrapher Prediction for Capana07g001256
167387527
167411739
Alt. 3'

CaWRKY38

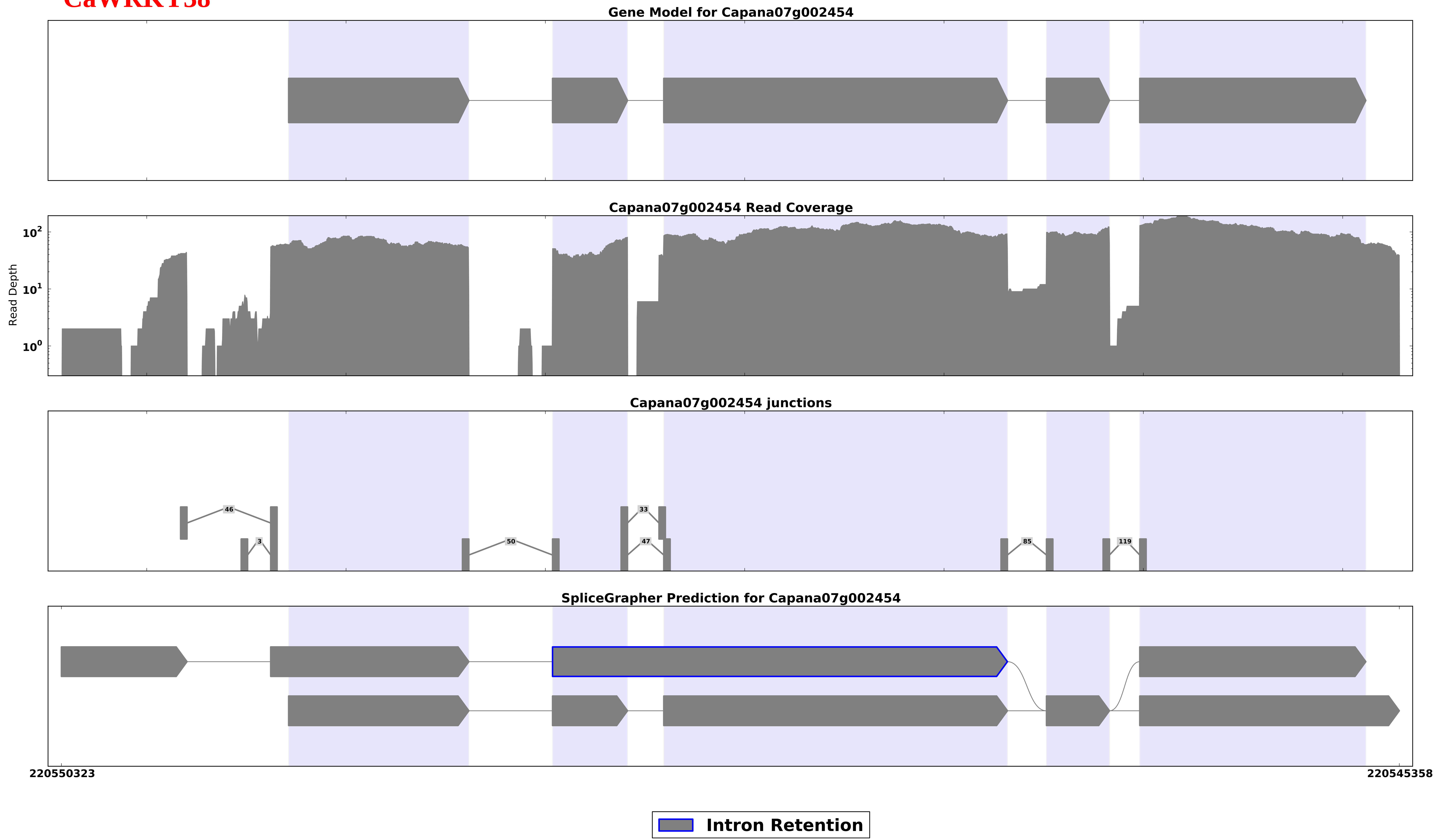

Gene Model for Capana07g002454
Capana07g002454 Read Coverage
Read Depth
10^2
10^1
10^0
Capana07g002454 junctions
46
3
50
33
47
85
119
SpliceGrapher Prediction for Capana07g002454
220550323
220545358
Intron Retention

CaWRKY51

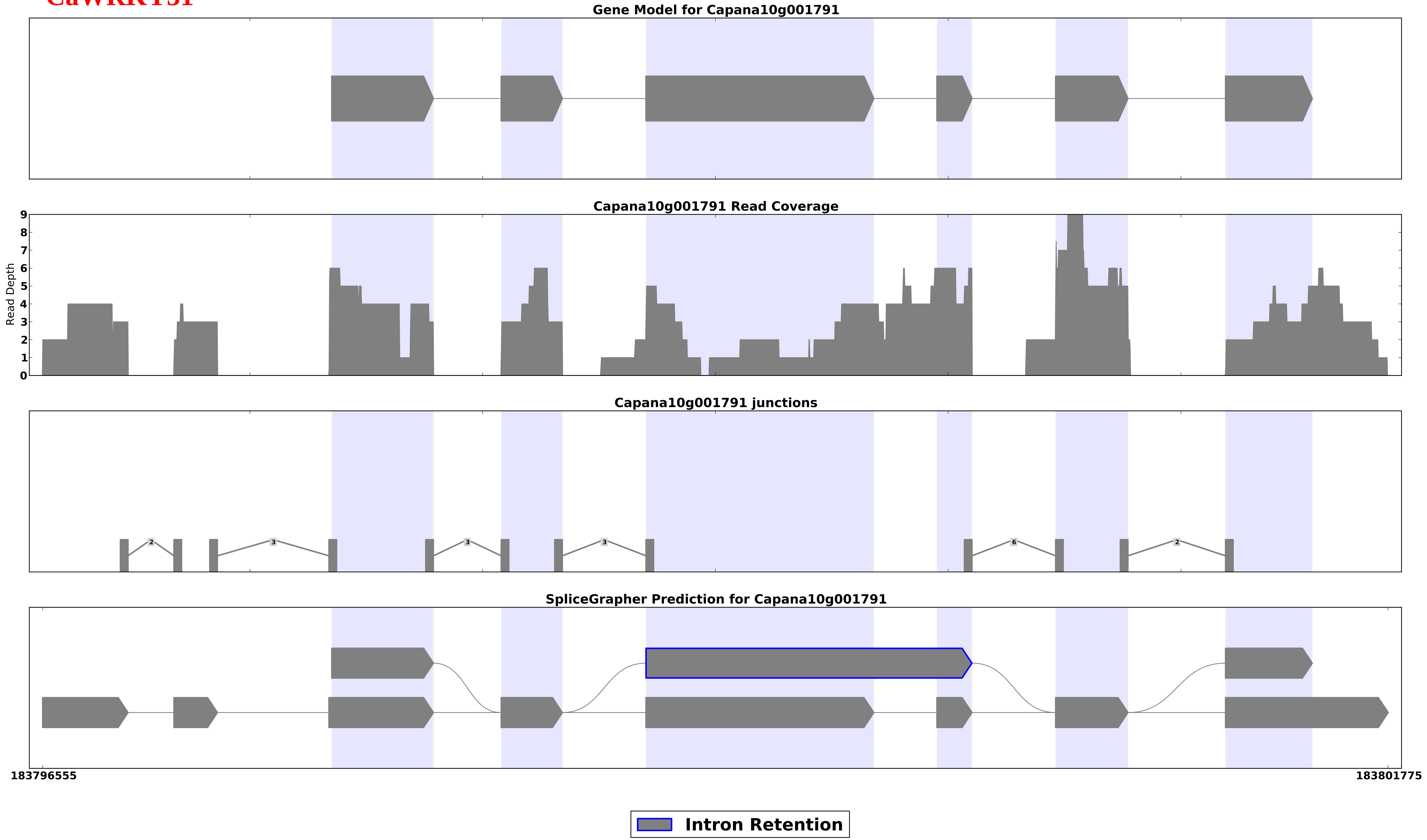

Gene Model for Capana10g001791
Capana10g001791 Read Coverage
Read Depth
0
1
2
3
4
5
6
7
8
9
Capana10g001791 junctions
SpliceGrapher Prediction for Capana10g001791
183796555
183801775
Intron Retention
