# Supplementary material for: Identification, expression, alternative splicing and functional analysis of pepper WRKY gene family in response to biotic and abiotic stresses
Source: PLoS One. 2019 Jul 22;14(7):e0219775. doi: 10.1371/journal.pone.0219775 (PMC6645504; doi:10.1371/journal.pone.0219775)
Supplement: S2 Table — (PDF) [file pone.0219775.s006.pdf]

S2 Table The WRKY genes identified in hot pepper.

Gene[a]	Annotation ID[b]	GeneBank accession	WRKY domain number	Predicted ORF length	Predicted Gene length[c]
CaWRKY1	Capana01g000165		1	573	3882
CaWRKY2	Capana01g000167		1	972	1861
CaWRKY3	Capana01g002803		1	684	2136
CaWRKY4	Capana01g003441		1	720	4199
CaWRKY5	Capana01g004471		1	744	1607
CaWRKY6	Capana01g004472	FJ360844	1	1095	2929
CaWRKY7	Capana02g000212		1	1089	4519
CaWRKY8	Capana02g000680		1	558	1604
CaWRKY9	Capana02g000918		1	1368	5845
CaWRKY10	Capana02g001642	DQ102364	1	912	1443
CaWRKY11	Capana02g002230	GQ253367	1	1662	2529
CaWRKY12	Capana02g003053		1	912	1600
CaWRKY13	Capana02g003339	AY740531	2	1344	2867
CaWRKY14	Capana02g003661		1	483	1467
CaWRKY15	Capana03g000473	GQ249255	1	1083	1807
CaWRKY16	Capana03g001099		1	975	3097
CaWRKY17	Capana03g001962		1	786	785
CaWRKY18	Capana03g002072		1	1098	1270
CaWRKY19	Capana03g002134		1	1011	1232
CaWRKY20	Capana03g002635	KF484401	1	900	2715
CaWRKY21	Capana03g003085		2	1851	8497
CaWRKY22	Capana03g003279		1	1020	1874
CaWRKY23	Capana04g000568		1	1053	1526
CaWRKY24	Capana04g001820		2	1464	8674
CaWRKY25	Capana05g002502		2	1524	3593
CaWRKY26	Capana06g001008		1	1896	7440
CaWRKY27	Capana06g001110		1	1086	1499
CaWRKY28	Capana06g001506	DQ402241	2	1647	2518
CaWRKY29	Capana06g002128		1	684	2051
CaWRKY30	Capana06g003072		1	801	1930
CaWRKY31	Capana07g000181		2	1368	8463
CaWRKY32	Capana07g000528		1	594	1192
CaWRKY33	Capana07g001256		2	2610	23687
CaWRKY34	Capana07g001387	KF736800	1	1911	2784
CaWRKY35	Capana07g001809		1	804	3913
CaWRKY36	Capana07g001968		1	957	3540
CaWRKY37	Capana07g002350		2	1764	5524
CaWRKY38	Capana07g002454		2	2232	3519
CaWRKY39	Capana08g000429		1	501	1352
CaWRKY40	Capana08g000683		1	795	2653
CaWRKY41	Capana08g001012		1	1122	1378
CaWRKY42	Capana08g001044		1	990	1924

CaWRKY43	Capana08g001961		1	1497	2105
CaWRKY44	Capana09g000676		1	663	1665
CaWRKY45	Capana09g001251	AY391747	2	1641	4091
CaWRKY46	Capana09g001790		1	861	1968
CaWRKY47	Capana10g000205		1	2112	2990
CaWRKY48	Capana10g000754		1	1149	2176
CaWRKY49	Capana10g001220		1	648	724
CaWRKY50	Capana10g001548		1	699	3050
CaWRKY51	Capana10g001791		2	1260	2886
CaWRKY52	Capana10g001805		1	1017	1397
CaWRKY53	Capana11g001882	DQ180348	2	1473	5095
CaWRKY54	Capana11g001905		1	924	5579
CaWRKY55	Capana12g001134		1	729	1310
CaWRKY56	Capana12g001826		1	564	13634
CaWRKY57	Capana12g001851		1	702	5017
CaWRKY58	Capana00g000429		1	414	1460
CaWRKY59	Capana00g001033	AY743433	1	513	1460
CaWRKY60	Capana00g003083		1	993	1664
CaWRKY61	Capana00g004057		2	1821	4145
CaWRKY62	Capana00g004112		1	672	2326

[a] The names *CaWRKY1-62* are given according to the chromosome order.

[b] Reannotated genes with original sequences in ZUNLA-1 genome database.

[c] Include intron length
