# Supplementary material for: Identification, expression, alternative splicing and functional analysis of pepper WRKY gene family in response to biotic and abiotic stresses
Source: PLoS One. 2019 Jul 22;14(7):e0219775. doi: 10.1371/journal.pone.0219775 (PMC6645504; doi:10.1371/journal.pone.0219775)
Supplement: S3 Table — (PDF) [file pone.0219775.s007.pdf]

S3 Table. The nucleotide and protein sequences of 62 CaWRKY gene family

Part I The nucleotide sequences of CaWRKY gene family in pepper genome database

CaWRKY1

>Capana01g000165 [mRNA] locus=Chr01:2380410-2384292
ATGCAACATTACAACTCAACAATAATGGACTCCGGCAAAGAGCCGCCGCCACCACCACCACCACCGTTGCTA
TCGACCGATCTATCTCCGGCCAACTTCTTCATGCAACAACCTAATGATCAAATGCCCATGCAAAATAATTATC
ACTCACACGTTGGACTTGATTGTGATAATATTGATTGGGCCGGGCTTTTATCGGCGGGCCCTTCAATTAATAA
TGAGTCGATTGCAACTACGTCAAATGTATCGATAAATCATAGGAATATGAATACAAATATGATGGAAGGTGG
TGAACAACATCAACAACAATTATTACCACAACAACAACAACATGAAGTATGTAGAAGAGATAAAGGGAGAA
TAATAAAGAAGAGAAAATATGTACCACCTAGGATTGCATTTCATACAAGGAGTACTGAGGATATTCTTGATG
ATGGATTTAAGTGGAGAAAATATGGTCAAAAAGCTGTCAAAAATAGTACACATCCAAGAGATGTTGGTGAAA
CATCAATGCAAGAACTAGGCTATGCAGGGCTCAAGTTAACAACTGATGTTAACTACCTCTCCCGTTGA
>CA01g01900
ATGCAAAATAATTATCACTCACACGTTGGACTTGATTGTGATAATATTGATTGGGCCGGGCTTTTATCGGCGG
GCCCTTCAATTAATAATGAGTCGATTGCAACTACGTCAAATGTATCGATAAATCATAGGAATATGAATACAAA
TATGATGGAAGGTGGTGAACAACATCAACAACAATTATTACCACAACAACAACAACATGAAGTATGTAGAAG
AGATAAAGGGAGAATAATAAAGAAGAGAAAATATGTACCACCTAGGATTGCATTTCATACAAGGAGTACTG
AGGATATTCTTGATGATGGATTTAAGTGGAGAAAATATGGTCAAAAAGCTGTCAAAAATAGTACACATCCAA
GGAGTTACTATCGATGTACACATCACACATGCAATGTGAAAAAACAAATTCAAAGACATTCAAAGGATACAA
GTATTGTGGTGACAACTTATGAAGGCATTCACAATCACCCTTGTGAGAAACTAATGGAGACCTTAAGTCCAAT
TCTAAAGCAACTTCAGTTTCTCTCTAGGTTCTAA

CaWRKY2

>Capana01g000167 [mRNA] locus=Chr01:2417686-2419547
ATGGATTGGGGTCTTCAAGCTGTTGTTATTGGATCATCAAGTACTTCATGTAGTGATATTCATGGATCAAATTA
TGTTGATTTTTCACCTAATTTGGATTTTCAAGAAAGTGAATATTTATCTTTTTCTCATCATCATCATCAACATCA
TGAGATGAAAAAAGAGGTTTATAGTGATGAATTAGAACAACTTTACAAGCCATTTTATCATGTTGGTGGACA
AAATATGTTGATGGGATCTTCAATTTCATTAGTTCCCAAAGAAGTTATTAAAGAAGAGAAAAGAGAAGAAGA
GCAACAACAAGTGGTGGCTGCTAGCAATACATATGTACCCAAGTACAAAAAAAAGGAAAAATGAACAAAAGA
GAGTGGTGCTTCAGTTGAAAGCAGATGATCTTTCTTCTGATAAGTGGGCATGGAGAAAATATGGTCAAAAAC
CTATTAAAGGCTCCCCTTATCCAAGGAGTTATTATAGGTGCAGTAGCTCAAAGGGGTGTTTAGCAAGAAAAC
AAGTAGAACAAAGTTGTACTGAACATGGAATTTTTATTGTGACATACACAGCTGAACACAACCATAGTCAAC
CAACACGTAGAAATTCACTAGCTGGTACTACCAAAAGCAAATTTCCAAATTCAAAAAACTCCATTAATAGTCC
CAAGAAGATTGTGAAAGAAGAAAAAATTACAAGTCCACATTGTTCAACATCGAATTTAGGGTTATCTCCAGA
AGCAGCACGTATGATCGACGAATTTCCAGAAATAAATCAAGAGAATAATATTATTATAGGTGGTGACAATAA
TTATTATGATGAAGATGGGATGAGAAATATTTTTGAAGGAAGTGATGAAAATGAATGTGTTTCAATTGAAGA
AATGTTTGATGGAGATTTCTTTGCTGGTCTTGAAGATATTCATGATGGATTTAATTCATCTTTTGGATGTAATA
ATTCAGCATTTCCCTTTTCTAGTACTTGA
>CA01g01920
ATGATAATGGATTGGGGTCTTCAAGCTGTTGTTATTGGATCATCAAGTACTTCATGTAGTGATATTCATGGATC
AAATTATGTTGATTTTTCACCTAATTTGGATTTTCAAGAAAGTGAATATTTATCTTTTTCTCATCATCATCATCA
ACATCATGAGATGAAAAAAGAGGTTTATAGTGATGAATTAGAACAACTTTACAAGCCATTTTATCATGTTGGT
GGACAAAATATGTTGATGGGATCTTCAATTTCATTAGTTCCCAAAGAAGTTATTAAAGAAGAGAAAAGAGAA
GAAGAGCAACAACAAGTGGTGGCTGCTAGCAATACATATGTACCCAAGTACAAAAAAAAGGAAAAATGAACA
AAAGAGAGTGGTGCTTCAGTTGAAAGCAGATGATCTTTCTTCTGATAAGTGGGCATGGAGAAAATATGGTCA
AAAACCTATTAAAGGCTCCCCTTATCCAAGGAGTTATTATAGGTGCAGTAGCTCAAAGGGGTGTTTAGCAAG
AAAACAAGTAGAACAAAGTTGTACTGAACATGGAATTTTTATTGTGACATACACAGCTGAACACAACCATA

GTCAACCAACACGTAGAAATTCACTAGCTGGTACTACCAAAAGCAAATTTCCAAATTCAAAAAACTCCATTA
ATAGTCCCAAGAAGATTGTGAAAGAAGAAAAAATTACAAGTCCACATTGTTCAACATCGAATTTAGGGTTAT
CTCCAGAAGCAGCACGTATGATCGACGAATTTCCAGAAATAAATCAAGAGAATAATATTATTATAGGTGGTG
ACAATAATTATTATGATGAAGATGGGATGAGAAATATTTTTGAAGGAAGTGATGAAAATGAATGTGTTTCAA
TTGAAGAAATGTTTGATGGAGATTTCTTTGCTGGTCTTGAAGATATTCATGATGGATTTAATTCATCTTTTGGA
TGTAATAATTCAGCATTTCCCTTTTCTAGTACTTGA

CaWRKY3

>Capana01g002803 [mRNA] locus=Chr01:178651461-178653597
ATGTATTACCTTGATAATAATTTTGCAGTCACTAATAGCCATTCATTTACAGGCCTAATCTCAGATTATTATGG
TGTTGAAGGTAGGAATATTATGAATACATCGTCTTCTTTGGGGTTCATGGAGTTATTGGGTTTTCAAGATTTGA
TGTGTTCATCATCAGCTTCATTCTTTGAGTTACCAAAAGAAGAAAACTCCTGTCCTGCAGTTTGTGTATCTGAA
GAAGTGAAGCCAACTGCAGGTGAAAGCCAAAATAAGCTTATAAGTACTGTAGCAGCAGCTAACGTATTCAAT
ACGCCATCTACCCCAAACTGTTCCTCTATTTCATCCGAGACAAATGAGGGCCACACTAATACCACTCATGAGG
ATGCAGAGGCCGGGGAAGTACTAGATCATCATGACCAACAACACACCAACACTAAACAACAGTTGAAAGCG
AAGAAAACAGTTAGTCAGAAGAAGCAGAGAGAGCCGAGATTTGCATTCATGACAAAGAGTGAGGTTGATTTT
CTGGAAGATGGTTACAGATGGAGAAAATATGGTCAAAAAGCTGTCAAAAACAGCCCATTTCCCAGGAACTAT
TATCGCTGCACAAGCGCAACTTGCAATGTAAAGAAGAGAGTAGAGCGATGCTTCAGTGACCCAAGCATAGTG
GTTACTACCTACGAAGGAAAAGTCCCATGA
>CA01g22410
ATGTATTACCTTGATAATAATTTTGCAGTCACTAATAGCCATTCATTTACAGGCCTAATCTCAGATTATTATGG
TGTTGAAGGTAGGAATATTATGAATACATCGTCTTCTTTGGGGTTCATGGAGTTATTGGGTTTTCAAGATTTGA
TGTGTTCATCATCAGCTTCATTCTTTGAGTTACCAAAAGAAGAAAACTCCTGTCCTGCAGTTTGTGTATCTGAA
GAAGTGAAGCCAACTGCAGGTGAAAGCCAAAATAAGCTTATAAGTACTGTAGCAGCAGCTAACGTATTCAAT
ACGCCATCTACCCCAAACTGTTCCTCTATTTCATCCGAGACAAATGAGGGCCACACTAATACCACTCATGAGG

ATGCAGAGGCCGGGGAAGTACTAGATCATCAGGACCAACAACACACCAACACGAAACAACAGTTGAAAGCG
AAGAAAACAGTTAGTCAGAAGAAGCAGAGAGAGCCGAGATTTGCATTCATGACAAAGAGTGAGGTTGATTTT
CTGGAAGATGGTTACAGATGGAGAAAATATGGTCAAAAAGCTGTCAAAAACAGCCCATTTCCCAGGAACTAT
TATCGCTGCACAAGCGCAACTTGCAATGTAAAGAAGAGAGTAGAGCGATGCTTCAGTGACCCAAGCATAGTG
GTTACTACCTACGAAGGAAAACATACCCATCTAAGTCCCATGAATACGATCATGCCCCGCCCTAGCTGCTATC
CAATTACTCCAGTACCCGCTTCACCTGGTGCCTTCCCTTTGCCGATGCAGTTCAATATTAATCAGTCCTTCAAC
AACTTGACAAGTTCTTTAGCCATGAATAATCAGCTTGATCATGCTGCTTTTGTTGCTCAAGGAAGGCGCTTTTG
CACTTCCGAAATGCTGGGAGACGAGGGGCTTCTTCAGGATCTTATGCCCTCCACGTTGATTAAAGAAAATTAC
AGATGA

CaWRKY4

>Capana01g003441 [mRNA] locus=Chr01:226400966-226405165
ATGGAAAGGGGTGGTGCAGAAAGGGATCATCAGTTAAACAACTACAATTTACAAGTTTCTTTCTCATCATCAT
CAGTAGCAGCTAATAATATCCATGAATTGGGATTTGTACACTTTGCAGATCATAACTTGAGTTTCTTAGCTCCT
TCATCACAATCTTCTCAAATATCTCAGCCACTTCAAGCCGCCAGCGTCAGCGTTACGCCACCTACCACTATCA
ACACAAACGTTGCAGCTGGCGGCTCGAATAACGTTACTGGTGGTCTAGGGTTTAGCCACAATGAACTTGTCAT
CAATAGATCTTCTTGGAACAGTGACCAGGTGGAAACACTAGATCCCAAGGCTGTTAATGACGAAAATTGCGG
CGGTAATGCCAACGAGGGTAACAATTCATGGTGGAAGACTTCATCTTCAGACAAAGGAAAGGTGAAGATAAG
GAGAAAGCTAAGAGAACCAAGGTTTTGTTTCCAAACAAGAAGTGACATTGATGTTCTTGATGATGGATATAA
ATGGAGAAAATATGGTCAGAAAGTTGTCAAGAACAGTCTTCATCCCAGGAGTTACTATCGGTGTACACATAG
TAATTGTAGAGTGAAGAAAAGAGTTGAAAGACTATCAGAAGATTGCCGTATGGTAATAACTACCTATGAAGG
TAGACATAACCATTCTCCTTGTGATGATTCTAACTCTTCTGATCATGATTGTTTCACCTCTTTCTAG
>CA01g28510ATGGAAAGGGGTGGTGCAGAAAGGGATCATCAGTTAAACAACTACAATTTACAAGTTTCTTTCT
CATCATCATCAGTAGCAGCTAATAATATCCATGAATTGGGATTTGTACACTTTGCAGATCATAACTTGAGTTT
CTTAGCTCCTTCATCACAATCTTCTCAAATATCTCAGCCACTTCAAGCCGCCAGCGTCAGCGTTACGCCACCTA

CCACTATCAACACAAACGTTGCAGCTGGCGGCTCGAATAACGTTACTGGTGGTCTAGGGTTTAGCCACAATGA
ACTTGTCATCAATAGATCTTCTTGGAACAGTGACCAGGTGGAAACACTAGATCCCAAGGCTGTTAATGACGA
AAATTGCGGCGGTAATGCCAACGAGGGTAACAATTCATGGTGGAAGACTTCATCTTCAGACAAAGGAAAGGT
GAAGATAAGGAGAAAGCTAAGAGAACCAAGGTTTTGTTTCCAAACAAGAAGTGACATTGATGTTCTTGATGA
TGGATATAAATGGAGAAAATATGGTCAGAAAGTTGTCAAGAACAGTCTTCATCCCAGGAGTTACTATCGGTG
TACACATAGTAATTGTAGAGTGAAGAAAAGAGTTGAAAGACTATCAGAAGATTGCCGTATGGTAATAACTAC
CTATGAAGGTAGACATAACCATTCTCCTTGTGATGATTCTAACTCTTCTGATCATGATTGTTTCACCTCTTTCT
AG

CaWRKY5

>Capana01g004471 [mRNA] locus=Chr01:300738347-300739954
ATGGATTGTTCTTTCAACTGGCAATACAAGACACTCATCAATGAGCTAACTCGAGGAATTGAACACGCTAAGC
AGCTTAAAGCTTACTTGAGCTCTGTAGCTTCCACTTCTGAAAATCAAGAATTGCTTCTGCAGAAGATACTTTCT
TCTTACGAGCAATCTCTGAGTGATGACAAGAAAAGGTGTTTCAAAGAACATCAGGAGCTCATTGATATTTCGA
AGAAGAGAAAATCACAGCTCACACGGACGGAACAAGTCAAAGTCAGTGCGGAGAGTGGATTTGAAGGTCCT
ACCGATGATGGATATAGCTGGAGAAAGTATGGACAGAAACACATTCTTGGAGCTAAATATCCTAGAAGCTAC
TACAGATGCACATATCGTCACATGCAAAATTGTTGGGCAACAAAACAAGTGCAAAGGTCAGATGATGATGCT
ACTGTATATGAGATCACATATAGGGGTTCCCATAATTGTCGTCAAGCAACAAATCGAGCATCACTAGAGAAG
CAAGAACTAAAGAAACAAGCGGTTTATCAAACAGGACAGCAATATTCAAATCAAGCGTTGATGAACTCGAGA
GCAAACCTGAAAGTCGATACTGATGACTTGGAAAAGAATGAAACAGCGTGTCCTTTCTCCTTTCCTCCGACAT
TCTCTGGTTTGACAGATGAAAATCAACATTTCCAGATTTCCGATGTTGATGACAATCGAACTACTTCTCAGTCT
CAAGCAGCTGCCAGATGA
>CA01g34460
ATGGATTGTTCTTTCAACTGGCAATACAAGACACTCATCAATGAGCTAACTCGAGGAATTGAACACGCTAAGC
AGCTTAAAGCTTACTTGAGCTCTGTAGCTTCCACTTCTGAAAATCAAGAATTGCTTCTGCAGAAGATACTTTCT

TCTTACGAGCAATCTCTGGTAATTCTCAAACGGACTGGCTCAACAGTCCACTCCTCGAAGCCTCTGCCGCCAA
TGTGTGGTGCAATTGAATCTTCGGTATCTGTTGACGGAAGTCCTAAGAGTGATGACAAGAAAAGGTGTTTCAA
AGAACATCAGGAGCTCCTTGATATTTCGAAGAAGAGAAAATCACAGCTCACACGGACGGAACAAGTCAAAGT
CAGTGCGGAGAGTGGATTTGAAGGTCCTACCGATGATGGATATAGCTGGAGAAAGTATGGACAGAAACACAT
TCTTGGAGCTAAATATCCTAGAAGCTACTACAGATGCACATATCGTCACATGCAAAATTGTTGGGCAACAAA
ACAAGTGCAAAGGTCAGATGATGATGCTACTGTATATGAGATCACATATAGGGGTTCCCATAATTGTCGTCAA
GCAACAAATCGAGCATCACTAGAGAAGCAAGAACTAAAGAAACAAGCGGTTTATCAAACAGGACAGCAATA
TTCAAATCAAGCGTTGATGAACTCGAGAGCAAACCTGAAAGTCGATACTGATGACTTGGAAAAGAATGAAAC
AGCGTGTCCTTTCTCCTTTCCTCCGACATTCTCTGGTTTGACAGATGAAAATCAACATTTCCAGATTTCCGATG
TTGATGACAATCGAACTACTTCTCAGTCTCAAGCAGCTGCCAGATGA

CaWRKY6

>Capana01g004472 [mRNA] locus=Chr01:300742280-300745209
ATGAGAAAATTGCAGCCCACGTGGACCGAACAAGTCAAAGTCAGCCCAAAGAGCGGATTTGAAGGTCCTACC
GATGACGGATATAGCTGGAGAAAGTACGGCCAGAAGGATATTCTTGGAGCTAAATATCCGAGAAGCTACTAC
AGATGCACGTATCGTCACATGCAAAATTGTTGGGCAACGAAACAAGTGCAGAGGTCAGACGATGATCCGACT
GTATTCGATGTCACATACAGAGGCTCTCATAGCTGTCATCACGCTACTTATTACGTACAACAATCAACACCGC
CAGAGAAACGAGAATTCAAGAAAGAGGCCGTTTATCAAAACAGGCAGAATTATTCAACTCAAGCGTTGATGA
GTTTGAGAGCAAACCTGAGAGTCGATACGAACGACTTGGACAAGAATGAGCAAGCAGCATGTCATTTCTCCT
TTCCTCCAACATTTTCTTCTGGTTTGACAGACGAAAATCATCGACGTTTCCAGATTTCCCATGTCGATGAAAAT
CTGATAGGTAGCGGCTATTCAGCGTCTTTTGTCTCTCCTACAACTCCTGAATCGAACTACTTCTCAGTGTCGAG
CAGCAGCCAGATGAATGGTTACGGAATGATTCATAACTTGAACCATTCGGAATCAGACCTCACCGATATATTC
TCAGCCAACACTTCCACAACGAGTTCTCCAATTGTTGGCGATTTTTCACTCGACAATTTGGAGCTAGATACAA
ACTTTCCATTCAACAATCCAAATTTTTTCTCATGA
>CA01g34470

ATGGATTGTGCAGTTAACTGGGAATATAAGACACTTATAAATGAGTTAACTCAAGGTATTGAACACACAAAA
CAACTTAGAGCTCATTTCAGCTCTGTTGATTCGACTATCCAAAATCAAGAGCTGCTACTTCAGAAGATACTTT
CATCTTATGAGCAATCTTTGTTGATTCTCAAATGTAGCGTTGGTGGCTCGATGGTTCAATCATCGTCGGCTATG
ATGCCGACGTGTGGTGTCATTGAATCATCAGTGGTGTCTGTCTATGGAAGTCCAAAGAGCGATGACAAGAAA
CGGAGTTTCCAAGATCATCATGAGGTTATCGATATTTCAAAGAAGAGAAAATTGCAGCCCACGTGGACCGAA
CAAGTCAAAGTCAGCCCAAAGAGCGGATTTGAAGGTCCTACCGATGACGGATATAGCTGGAGAAAGTACGGC
CAGAAGGATATTCTTGGAGCTAAATATCCGAGAAGCTACTACAGATGCACGTATCGTCACATGCAAAATTGTT
GGGCAACGAAACAAGTGCAGAGGTCAGACGATGATCCGACTGTATTCGATGTCACATACAGAGGCTCTCATA
GCTGTCATCACGCTACTTATTACGTACAACAATCAACATCGCCAGAGAAACGAGAATTCAAGAAAGAGGCCG
TTTATCAAAACAGGCAGAATTATTCAACTCAAGCGTTGATGAGTTTGAGAGCAAACCTGAGAGTCGATACGA
ACGACTTGGACAAGAATGAGCAAGCAGCATGTCATTTCTCCTTTCCTCCAACATTTTCTTCTGGTTTGACAGAC
GAAAATCATCGACGTTTCCAGATTTCCCATGTCGATGAAAATCTGATAGGTAGCGGCTATTCAGCGTCTTTTG
TCTCTCCTACAACTCCTGAATCGAACTACTTCTCAGTGTCGAGCAGCAGCCAGATGAATGGTTACGGAATGAT
TCATAACTTGAACCATTCGGAATCAGACCTCACCGATATATTCTCAGCCAACACTTCCACAACGAGTTCTCCA
ATTGTTGGCGATTTTTCACTCGACAATTTGGAGCTAGATACAAACTTTCCATTCAACAATCCAAATTTTTCTC
ATGA

CaWRKY7

>Capana02g000212 [mRNA] locus=Chr02:26415840-26420359
ATGATTCCACAGGCCAGTTCAAGCTTATTCAACCCGACCCAAGAAAATAATAATCATTTTGGACCAAACATTG
ATTCTCCATCAATGAAAATTAGACGACCTAACATCTTTTCAACGATGCTTCAGATCTCTCCTACCACCAAGTTG
GCCATGTCACCATGTGATATAGCCTGCTCCTCCCCCAACTCTAATGTTAATGTTATTGGTGCATTAGTTCCTAC
TCAAGATGCTATTATCTCTCCTAATTCCTCCAAAACATGCTTAGTGGAAAACTCTGGCTTGCAGATCTCATCTC
CGCGAAATACGGGTATCAAAAGAAGAAAGAGTCAGGCAAAAAAGGTGGTGTGTATACCAGCACCAGCACCT
GCAAATAGCCGGCAAGGGGGAGAAGTTGTTCCATCTGATTTATGGGCTTGGAGAAAGTACGGTCAGAAGCCA

ATCAAAGGCTCCCCTTACCCAAGAGGTTACTATAGGTGCAGTAGTTCCAAGGGATGTTCAGCAAGGAAGCAA
GTGGAAAGAAGCAGGACAGACCCAAACATGTTAGTCATCACCTACACTTCAGAACATAACCATCCATGGCCA
ACTCAAAGAAATGCCCTTGCTGGATCAACCAGGTCTCAGCCTAATAATTCCAAACACACTACAACCTCAAAG
AACAACACTAATATTATGCCAAATAATTCTCAATACCAAGGCGATACAAGTTTCAATGAAGATGAACAAAAT
GAGAGAAACATTAATCATGACAATAATGTTGCACAAGCGAACATTTCAACGTACCCAAAAGTGAAGGAAGA
GGTGGCCGAAGAAGATCTTCAACAGCAACTAGGGGAAATGCGTAATGTTGAATTTTCTAAAGGGAGTTATCA
GCCAATATTACCAGACTCGTCGAATCAGTGTCATGAGGATTTCTTTGCAGATTTGGTGGAACTTGAAGCTGAC
CCTCTAAACTTTTTGTTTGCCAATACTCTCTCGGGAGATATCAATGAAGTGGGACAGAAGAAGGCCATCGACG
CATTCAACTTGTACAATTGGAGCAAAGACCGTAACACCAACATAAACAACAAGGGTACTCAAGCCGACACAT
AA
>CA02g03480
ATGTGCAGCCCGCAAAAAGACATGACGAATAATTATCAAGGTGATTTAGCTGATATATTCAGAGGTGGAAAT
AGTACCACTTCCGGTGATCAGTCATCAACTAGTGTTGTTCCTGTTCCTGACGGATGGCAGTTTCCTAGCATAA
ACTATTCGGCCTCTGTGATTGAAGAACCGACGGCGTCGTTGGTTCAAGATTTTGGAGATCCATTTTGTAACTT
GAGAGATCCACTACTCTTCCATGATCTTGATATGATTCCACAGGCCAGTTCAAGCTTATTCAACCCGACCCAA
GAAAATAATAATCATTTTGGACCAAACATTGATTCTCCATCAATGAAAATTAGACGACCTAACATCTTTTCAA
CGATGCTTCAGATCTCTCCTACCACCAAGTTGGCCATGTCACCATGTGATATAGCCTGCTCCTCCCCCAACTCT
AATGTTAATGTTATTGGTGCATTAGTTCCTACTCAAGATGCTATTATCTCTCCTAATTCCTCCAAAACATGCTT
AGTGGAAAACTCTGGCTTGCAGATCTCATCTCCGCGAAATACGGGTATCAAAAGAAGAAAGAGTCAGGCAAA
AAAGGTGGTGTGTATACCAGCACCAGCACCTGCAAATAGCCGGCAAGGGGGAGAAGTTGTTCCATCTGATTT
ATGGGCTTGGAGAAAGTACGGTCAGAAGCCAATCAAAGGCTCCCCTTACCCAAGAGGTTACTATAGGTGCAG
TAGTTCCAAGGGATGTTCAGCAAGGAAGCAAGTGGAAAGAAGCAGGACAGACCCAAACATGTTAGTCATCA
CCTACACTTCAGAACATAACCATCCATGGCCAACTCAAAGAAATGCCCTTGCTGGATCAACCAGGTCTCAGCC
TAATAATTCCAAACACACTACAACCTCAAAGAACAACACTAATATTATGCCAAATAATTCTCAATACCAAGG
CGATACAAGTTTCAATGAAGATGAACAAAATGAGAGAAACATTAATCATGACAATAATGTTGCACAAGCGAA

CATTTCAACGTACCCAAAAGTGAAGGAAGAGGTGGCCGAAGAAGATCTTCAACAGCAACTAGGGGAAATGC
GTAATGTTGAATTTTCTAAAGGGAGTTATCAGCCAATATTACCAGACTCGTCGAATCAGTGTCATGAGGATTT
CTTTGCAGATTTGGTGGAACTTGAAGCTGACCCTCTAAACTTTTTGTTTGCCAATACTCTCTCGGGAGATATCA
ATGAAGTGGGACAGAAGAAGGCCATCGACGCATTCAACTTGTACAATTGGAGCAAAGACCGTAACACCAAC
ATAAACAACAAGGGTACTCAAGCCGACACATAA

CaWRKY8

>Capana02g000680 [mRNA] locus=Chr02:83748217-83749821
ATGGAGATTGAAATTGCAGCTGATGCTGCTATCACGAAGTTTAAGAAGGTAAATTCTCTTCTGGATCGATTCA
GAACTGGTCATGCTCGGTTCAGAAGAGCACCTATTGATAATCTCAAAAAAGATTATGTTGACCCTGAGGTTTC
TTCTGCTGTAAAGCCCCCTTTGTCGTCTTCGTCTTCTTTCAAAATTAGAAAGTGCACTTCTTCGGAGAACGGAT
TTTCTGGAAAGTGTAGTGGCTCATCCGGTCGATGTCATTGCTCAAAGAGAAGGAAATTAAGACCTAAAAGGG
TAGTTAGAGTTCCAGCAATAAGCATGAAGTTGTCAGACATCCCACCTGATGATTATTCATGGAGGAAATATGG
ACAAAAGCCAATTAAAGGATCTCCACATCCAAGGGCATATTACAAATGTAGTAGTGTGAGAGGTTGTCCAGC
ACGTAAACATGTAGAAAGAGCTTTGGATGAGCCAACGATGCTGGTTGTCACGTACGAAAGCGAGCATAACCA
TTCCCTCTCTGTTGCTGAAACAAGTAGTCTCATTTTAGAGTCTTCCTAA
>CA02g01800
ATGGAGATTGAAATTGCAGCTGATGCTGCTATCACGAAGTTTAAGAAGGTAAATTCTCTTCTGGATCGATTCA
GAACTGGTCATGCTCGGTTCAGAAGAGCACCTATTGATAATCTCAAAAAAGATTATGTTGACCCTGAGGTTTA
TTGTCTTACTCCAATTCAACAACTCCCTCCAAGTGCTTACGACCTTAATAATAACCAAATCTTCCAAAATCCAA
AACAAGAATTGGTCACAAAATCAATTAATTTTTCGCATGCCCCTGAAATTTGGTGCGCAAATTCGTTTAATAT
GTCAACGTTAACAGGGGAAACAGAGAGTGAGCAAATTTCAAATCTTTCTCAGGTTTCTTCTGCTGTAAAGCCC
CCTTTGTCGTCTTCGTCTTCTTTCAAAATTAGAAAGTGCACTTCTTCGGAGAACGGATTTTCTGGAAAGTGTAG
TGGCTCATCCGGTCGATGTCATTGCTCAAAGAGAAGGAAATTAAGACCTAAAAGGGTAGTTAGAGTTCCAGC
AATAAGCATGAAGTTGTCAGACATCCCACCTGATGATTATTCATGGAGGAAATATGGACAAAAGCCAATTAA

AGGATCTCCACATCCAAGGGCATATTACAAATGTAGTAGTGTGAGAGGTTGTCCAGCACGTAAACATGTAGA
AAGAGCTTTGGATGAGCCAACGATGCTGGTTGTCACGTACGAAAGCGAGCATAACCATTCCCTCTCTGTTGCT
GAAACAAGTAGTCTCATTTTAGAGTCTTCCTAA

CaWRKY9

>Capana02g000918 [mRNA] locus=Chr02:102411100-102416945
ATGGAGAAAAATTTAGATAATTCCCTCTCAGGAGCTAGTGCAGTTAAAGAAATCCATAATCATGAAGAAGAC
GATATCAGAAAAGCTTTAAAGGATGATGAATTTAAGTCAGCCTTGGCAGAAGTGAATGAGGTCAAACTAGAG
AACGCAAGATTAAAAATGTTGCTTCAACAAATAGAGAATGATTACAATTCTCTCCAAACTCGATTCTTCAATA
TTTGTCAACCAGATTTGAAAAAGAGTGTCAACCCAACATGCACTTCTGAAAAAATTGCAGATGAAGAAGAAT
GTGAACTTGTCTCGCTTCGACTCGGGCGAAGTCCGAGTCCAAGCGAGTCCAAAAAAGTTGATAAGAAAAGAA
CCAGAGAGGACTATCATTGTGTTCAGTCCAATGATGGGCTTAAACTTGGGCTGGACTACAGCAGTGGGGTATC
TGAATCAGATCTTCTAAAACCCAACAACGATCCAAGCCCAGGCCCAACTAGTGAAGTTGTCAAGACAATGAG
AAAAGGTGATGATGAAAGTGTCAAAAAAAGAGCTGGTGATGATGAAGTTTCTCAGCCTAATGTCAAGAGAGC
TAGAGTTTCAGTTCGGACAAAATGCGACTACCCAACAATAAATGATGGTTGTCAGTGGAGGAAATATGGGCA
GAAGATATCAAGAGGTAATCCGTGCCCACGAGCATATTATCGTTGCTCAGTCGCGCCATTATGCCCTGTGAGA
AAACAAGTCCAACGATGTCTCGAAGACATGTCTATATTGATCACAACTTATGAAGGAACACATAATCACTCA
CTTCCCATTGAGGCCACAGCTATGGCCTCAACTACTGCTGCTGCAGCTTCCATGCTTCTTTCTGGCTCATCGAC
AAGTTCTCAATCACCTAAAAATTTCACAAATTTGGCCAATTATTCGAAAACAACACCTCTCTATTTATCCAATT
CTTCTTCAAATCCTTTTCCCACCATCACTTTAGACTTCACCGCGTTTCCAACTACTTCATCATTTACTAGTTTCA
ACTTCCCTTCCAATTTCCAACCCGGCTCGGGACTTCTTTCTAACAGCTTAAGCTTTTCCTCGCCTGAGTCGTCC
ACAATACCCAAAATCTTGGGTAGTGGATGCCTAAATTATGATTCTACAAGTACATTGCCATATCACAAGAACC
TCATAAACATTGGCTCATCCCAAAAACAATTTGACCAACCATTTATTGGAAAGAACAACACTAGTACTAGTGA
TAAATTGAAGGAGGATTCTTCTCAGCAAGCACTAACTGAAACATTGACAAAGGCAATTACATCAGATCCTAG
CTTTCAATCAGTGCTAGCTGCTGCTATTTCATCAATGGTTGGTGCTACCAAAACTTGA

>CA02g08530ATGAGAAAAGGTGATGATGAAAGTGTCAAAAAGAGAGCTGGTGATGATGAAGTTTCTCAGCCT
AATGTCAAGAGAGCTAGAGTTTCAGTTCGGACAAAATGCGACTACCCAACAATAAATGATGGTTGTCAGTGG
AGGAAATATGGGCAGAAGATATCAAGAGGTAATCCGTGCCCACGATCATATTATCGTTGCTCAGTCGCGCCA
TTATGCCCTGTGAGAAAACAAGTCCAACGATGTCTCGAAGACATGTCTATATTGATCACAACTTATGAAGGAA
CACATAATCACTCACTTCCCATTGAGGCCACAGCTATGGCCTCAACTACTGCTGCTGCAGCTTCCATGCTTCTT
TCTGGCTCATCGACAAGTTCTCAATCACCTAAAAATTTCACAAATTTGGCCAATTATTCGAAAACAACACCTC
TCTATTTATCCAATTCTTCTTCAAATCCTTTTCCCACCATCACTTTAGACTTCACCGCGTTTCCAACTACTTCAT
CATTTACTAGTTTCAACTTCCCTTCCAATTTCCAACCCGGCTCGGGACTTCTTTCTAACAGCTTAAGCTTTTCCT
CGCCTGAGTCGTCCACAATACCCAAAATCTTGGGTAGTGGATGCCTAAATTATGATTCTACAAGTACATTGCC
ATATCACAAGAACCTCATAAACATTGGATCATCCCAAAAACAATTTGACCAACCATTTATTGGAAAGAACAA
CACTAGTACTAGTGATAAATTGAAGGAGGATTCTTCTCAGCAAGCACTAACTGAAACATTGACAAAGGCAAT
TACATCAGATCCTAGCTTTCAATCAGTGCTAGCTGCTGCTATTTCATCAATGGTTGGTGCTACCAAAACTTGA

CaWRKY10

>Capana02g001642 [mRNA] locus=Chr02:129707611-129709054
ATGGAAGGAAGATTCAACAATTTTTTTGTTTCTGAGCAAGATGATTCCGAGAATTCACCGGAAAACAGCTCCG
ACTCGCCGCGTTCCGCCATGTTCAATGATAACAAGATGATCACTTCAACTTCATCCCCTAACAGAAGATCCAT
AGAAAAGAGAGTGGTGTCAGTGCCAATTAAAGAAGTTGAAGGATCAAAAATGAAAGGTGAGATAAGCATGC
CACCATCTGATTCTTGGGCATGGAGAAAATATGGACAAAAGCCCATCAAAGGCTCTCCCTATCCCAGGGGAT
ATTATAGATGCAGTAGTTCAAAAGGATGTCCAGCAAGAAAACAAGTAGAAAGGAGCCGTGCGGACCCAAAC
ATGTTGATAGTGACATATTCTTGTGAACATAACCATCCTTGGCCGGCTTCTAGAAGTAATCAACACAACCATC
GTACAATTACCCCTACATCATGCACCAATAATAACACAAAGACAAAGACGAAAACAATAGCATCACTAACCG
CATCACCAGCAACAACAACAATAATAACGACTTCTGATATTCCGGTTTTACACTTTGAGCAAGAAAAGGCCA
CGACCGATTTCGCAGTACGCCCATCGGAGCCCAATTCGGACGAAAAATTTGTCAATCTTGGTGAATCATCACT
TATTAATGCTGAATTTGGATGGTTTTCTGATTTAGTTGAGTGTAACTCTACTACCATACTAGAAAGTCCAATTT

TGACCCAAGTCGAAGTTAATGATTTTGACATGTCATCAACATTGACAATGCAAGAGGAAGATGTGTCACTTTT
CGCCGATCTCGGAGAGTTACCGGAATGTTCAAGGGTATTTGGCCGTGGAATGATGGAGAGGGACGAGGAGCG
CGACCGACATAGCTTAACACCGTGGTGTGGGACCACAGGCTAA
>CA02g13500
ATGGAAGGAAGATTCAACAATTTTTTTGTTTCTGAGCAAGATGATTCCGAGAATTCACCGGAAAACAGCTCCG
ACTCGCCGCGTTCCGCCATGTTCAATGATAACAAGATGATCACTTCAACTTCATCCCCTAAAAGAAGCAGAAG
ATCCATAGAAAAGAGAGTGGTGTCAGTGCCAATTAAAGAAGTTGAAGGATCAAAAATGAAAGGTGAGATAA
GCATGCCCCCATCTGATTCTTGGGCATGGAGAAAATATGGACAAAAGCCCATCAAAGGCTCTCCCTATCCCAG
GGGATATTATAGATGCAGTAGTTCAAAAGGATGTCCAGCAAGAAAACAAGTAGAAAGGAGCCGTGCGGACC
CAAACATGTTGATAGTGACATATTCTTGTGAACATAACCATCCTTGGCCGGCTTCTAGAAGTAATCAACACAA
CCATCGTACAATTACCCCTACATCATGCACCAATAATAACACAAAGACAAAGACGAAAACAATAGCATCACT
AACCGCATCACCAGCAACAACAACAATAATAACGACTTCTGATATTCCGATTTTACACTTTGAGCAACAAAA
GGCCACGACCGATTTCGCAGTACGCCCATCGGAGCCCAATTCGGACGAAAAATTTGTCAATCTTGGTGAATCA
TCACTTATTAATGCTGAATTTGGATGGTTTTCTGATTTAGTTGAGTGTAACTCTACTACCATACTAGAAAGTCC
AATTTTGACCCAAGTCGAAGTTAATGATTTTGACATGTCATCAACATTGACAATGCAAGAGGAAGATGTGTCA
CTTTTCGCCGATCTCGGAGAGTTACCGGAATGTTCAAGGGTATTTGGCCGTGGAATGATGGAGAGGGACGAG
GAGCGCGACCGACATAGCTTAACACCGTGGTGTGGGACCACAGGCTAAACAATTGGAGCAAAGACCGTAAC
ACCAACATAAACAACAAGGGTACTCAAGCCGACACATAA

CaWRKY11

>Capana02g002230 [mRNA] locus=Chr02:142213621-142216150
ATGGACAAAGGATGGGGTCTTACCCTTGAAAGTTCTTCTTCTGATAAAGTTGGTTTCTTCATGAACAAGC
CTGTTTTTGGTTTTAATTTAAGTCCAAGATTGAACCCTGCTGAAATGTTTCCCAGCTCCGATGATAAACGTGCC
ATCGTTAATGAAGTTGACTTCTTCTCTGAGAAAAAGCCTATTGTAAAGAAGGAGAATTCTCAGGGTGACAGG
ACTGATCAATGTGTTGTAAATACTGGATTACAACTTGTGATTGCAAACGCTGGAAGTGATCAATCAACAGTAG

ATGATGGGATTTCATCGGAATTAGTACTTGAAGATAAACGAGCTAAAATTCAGCAGTTGGCGCAATTGCAAG
TTGAGCTTCAGAGGATGAATTCCGAAAATCAGCGTTTAAAAGGGATGCTTACTCAAGTTAATAACAGTTATTC
TGCACTTCAGATGCATCTTGTTGTCGAAGCCAAGTTTAATGACGAAAAGAAGCAAGAGAAAGAAGGGACCAT
AGTTCCAAGACAATTCATGGAGCTGGGCCCAAGTGGTTCCAAAGCTGATCCACTGGATGAGCCATCTAATTCC
CATACTTCATCAGAAGAAAGAACGCTTTCTGGATCACCTCGCAACAATATGGAATTATTGTCAAGGGATAAG
GCAATTGGCCGCGAAGAGAGTCCAGAATCTGAAAGTTGGGCTCCAAATAAGGTCCCTAAATTAATGAATTCA
TCAAAACCTGTTGAGCAACCAACCGAAGCAACTATGAGGAAAGCTCGTGTCTCCGTCCGTGCCCGATCAGAA
GCTCCTATGATTAGTGATGGCTGCCAGTGGAGAAAATATGGTCAAAAGATGGCTAAAGGCAATCCGTGTCCA
CGTGCTTATTATCGTTGCACGATGGCTGTTGGTTGTCCAGTGCGCAAACAGGTGCAAAGGTGTGCGGAGGACA
GGACAATCTTAATAACAACATACGAAGGTACACATAACCACCCCCTACCACCAGCAGCCATGGCAATGGCAT
CCACTACATCAGCAGCAGCAAACATGTTGCTCTCTGGTTCCATGCCAAGTGCTGATGGACTTATGAACACAAA
TTTCCTAGCCAGGGCCATGCTTCCATGTTCATCAAACATGGCAACTATTTCAGCATCTGCTCCATTTCCAACGG
TTACATTGGACCTCACAGCCCAAAATTCAAATGCTGCACTGCCTAATTATCACCAAAGAGTTAACCACGCTAA
TAATGCTCAATTCCAATTCCCTTTACCAGCTGGACTTAATCACCCGAATTTCATCGCTTCAATGTCAGCTCCAC
AAATGCCTCAGGTTTTAGGCCAGGCTATGTATAACCAGTCAAAATTTTCGGGTTTACAAGTTTCTCAGGACAA
TATTCACCACCCATCAATTTCTCATGACACACTCTCCGCTGCCACGGCTGCGATCACTGCTGACCCTAACTTCA
CTGCCGCCCTTGCCGCGGCCATCTCCTCCATCATAGGTTGTGGTTCTCATCCCAACAACAACGGCAACAGTAC
CATGTCTGGTCCATCAAGCAATAACAACAACACTAGCAGTTTCCCAGGGAACTAA
>CA02g18540
ATGGACAAAGGATGGGGTCTTACCCTTGAAAGTTCTTCTTCTTCTGATAAAGTTGGTTTCTTCATGAACAAGC
CTGTTTTTGGTTTTAATTTAAGTCCAAGATTGAACCCTGCTGAAATGTTTCCCAGCTCCGATGATAAACGTGCC
ATCGTTAATGAAGTTGACTTCTTCTCTGAGAAAAAGCCTATTGTAAAGAAGGAGAATTCTCAGGGTGACAGG
ACTGATCAATGTGTTGTAAATACTGGATTACAACTTGTGATTGCAAACGCTGGAAGTGATCAATCAACAGTAG
ATGATGGGATTTCATCGGAATTAGTACTTGAAGATAAACGAGCTAAAATTCAGTTGGCGCAATTGCAAGTTG
AGCTTCAGAGGATGAATTCCGAAAATCAGCGTTTAAAAGGGATGCTTACTCAAGTTAATAACAGTTATTCTGC

ACTTCAGATGCATCTTGTTACACTCATGCAACAGCAGCAGCAACAACAACAGCAGCAACAAATGATTTCAAG
AACTGAAAGTACACATGCCCATGAGGTTGTCGAAGCCAAGTTTAATGACGAAAAGAAGCAAGAGAAAGAAG
GGACCATAGTTCCAAGACAATTCATGGAGCTGGGCCCAAGTGGTTCCAAAGCTGATCCACTGGATGAGCCAT
CTAATTCCCATACTTCATCAGAAGAAAGAACGCTTTCTGGATCACCTCGCAACAATATGGAATTATTGTCAAG
GGATAAGGCAATTGGCCGCGAAGAGAGTCCAGAATCTGAAAGTTGGGCTCCAAATAAGGTCCCTAAATTAAT
GAATTCATCAAAACCTGTTGAGCAACCAACCGAAGCAACTATGAGGAAAGCTCGTGTCTCCGTCCGTGCCCG
ATCAGAAGCTCCTATGATTAGTGATGGCTGCCAGTGGAGAAAATATGGTCAAAAGATGGCTAAAGGCAATCC
GTGTCCACGTGCTTATTATCGTTGCACGATGGCTGTTGGTTGTCCAGTGCGCAAACAGGTGCAAAGGTGTGCG
GAGGACAGGACAATCTTAATAACAACATACGAAGGTACACATAACCACCCCCTACCACCAGCAGCCATGGCA
ATGGCATCCACTACATCAGCAGCAGCAAACATGTTGCTCTCTGGTTCCATGCCAAGTGCTGATGGACTTATGA
ACACAAATTTCCTAGCCAGGGCCATGCTTCCATGTTCATCAAACATGGCAACTATTTCAGCATCTGCTCCATTT
CCAACGGTTACATTGGACCTCACAGCCCAAAATTCAAATGCTGCACTGCCTAATTATCACCAAAGAGTTAACC
ACGCTAATAATGCTCAATTCCAATTCCCTTTACCAGCTGGACTTAATCACCCGAATTTCATCGCTTCAATGTCA
GCTCCACAAATGCCTCAGGTTTTAGGCCAGGCTATGTATAACCAGTCAAAATTTTCGGGTTTACAAGTTTCTC
AGGACAATATTCACCACCCATCAATTTCTCATGACACACTCTCCGCTGCCACGGCTGCGATCACTGCTGACCC
TAACTTCACTGCCGCCCTTGCCGCGGCCATCTCCTCCATCATAGGTTGTGGTTCTCATCCCAACAACAACGGC
AACAGTACCATGTCTGGTCCATCAAGCAATAACAACAACACTAGCAGTTTCCCAGGGAACTAA

CaWRKY12

>Capana02g003053 [mRNA] locus=Chr02:154730960-154732560
ATGGCTGTGGACTTAATGATGGATTATAGAAACACTAGCAATAGTAGCAGCAATAATTGTATCAACTTCGTAA
CCAAATTGGAAGAAAAAGCAGTTGTTCAAGAAGCCGCTTCTGGTCTAGAGAGTGTTGAGAAACTCATCAGAA
TGTTGTCTCGTAATAAATCTCCACAAATTCAGCAGCAAAATAAATCCCCCATGGAGATCGAACTAGTGGCTGA
TGCAGCAGTAACAAAGTTCAAGAAGGTAATTTCACTTCTAGATCGAAACAGAACTGGTCATGCTAGATTCAG
AAGAGCCCCTTTAGCTGCTACCACTTCCCCTTCACCAACAAATTGTAACAAAGATATCGTCGACACTAAAGTT

TATTCTCCAACTCCAATCCAACAAGTCCCTTTAGTATCCTATGAACATTATAACCCTCTCGTTCCACCAAAGAC
GATTAGTTTCTCATATTCACCTGAGATGTCTCGTACAAACTCGTTCAATATATCATCGTTGACAGGGGATACG
GAGAGTAAACAACATTCTTCATCTTCAGCAGCTTTCCAGATTACCAATCTTTCCTCTCAAGTCACTAATTCTGC
TGGAAAGCCTCCGTTGTCTTCTTCTTCACTGAAAAGAAAGTGTAGTTTATCAGAAAATGCTGTATCTGGCAAG
TGCAGTGGACCCTCTGGTAGATGCCATTGTTCCAAGAGAAGGAAGTTAAGGCTGAAGAGGGTAATTAGAGTA
CCGGCAATAAGCATGAAACTGGCAGATATTCCACCTGATGATTACTCATGGAGAAAATATGGACAGAAGCCA
ATCAAAGGATCTCCACATCCAAGCTTTGTTTGCCCTCATGAAAAGAAGAAGGGTTTTCCGATCAATGTAAACT
GGCTGTTAATTAGACTAGTTAATCAGTCTGTTGAGTAA
>CA02g14640
ATGGCTGTGGACTTAATGATGGATTATAGAAACACTAGCAATAGTAGCAGCAATAATTGTATCAACTTCGTAA
CCAAATTGGAAGAAAAAGCAGTTGTTCAAGAAGCCGCTTCTGGTCTAGAGAGTGTTGAGAAACTCATCAGAA
TGTTGTCTCGTAATAAATCTCCACAAATTCAGCAGCAAAATAAATCCCCCATGGAGATCGAACTAGTGGCTGA
TGCAGCAGTAACAAAGTTCAAGAAGGTAATTTCACTTCTAGATCGAAACAGAACTGGTCATGCTAGATTCAG
AAGAGCCCCTTTAGCTGCTACCACTTCCCCTTCACCAACAAATTGTAACAAAGATATCGTCGACACTAAAGTT
TATTCTCCAACTCCAATCCAACAAGTCCCTTTAGTATCCTATGAACATTATAACCCTCTCGTTCCACCAAAGAC
GATTAGTTTCTCATATTCACCTGAGATGTCTCGTACAAACTCGTTCAATATATCATCGTTGACAGGGGATACG
GAGAGTAAACAACATTCTTCATCTTCAGCAGCTTTCCAGATTACCAATCTTTCCTCTCAAGTCACTAATTCTGC
TGGAAAGCCTCCGTTGTCTTCTTCTTCACTGAAAAGAAAGTGTAGTTTATCAGAAAATGCTGTATCTGGCAAG
TGCAGTGGACCCTCTGGTAGATGCCATTGTTCCAAGAGAAGGAAGTTAAGGCTGAAGAGGGTAATTAGAGTA
CCGGCAATAAGCATGAAACTGGCAGATATTCCACCTGATGATTACTCATGGAGAAAATATGGACAGAAGCCA
ATCAAAGGATCTCCACATCCAAGGGGATACTACAAGTGTAGCAGTGTAAGAGGGTGTCCAGCACGCAAACAT
GTTGAAAGAGCATCGGATGATCCAACTATGCTCATTGTTACATATGAAGGAGAACATAATCATTCACTTTCTG
TTGCAGAAACAAGTAGTCTTATTTTAGAATCTTCTTAA

CaWRKY13

>Capana02g003339 [mRNA] locus=Chr02:159222405-159225272
ATGGGAGAAACCGGGGGAGAAGCTTCAGCAATATCATTTCCAGCTTTAACAATTCCACCACGGCCCTCGTAC
GAATCCTTTTTTAACATGTCCTCTTTCAGCCCAGGTCCAATGTCCTTAGTTTCCAGTTTCTTATCCGAACAGAG
CCCTGATTCTGCAGACCGTCCTTCTTTCTCCCAGCTTCTTGCTGGAGTCATTGCTTCACCGACACTTTTGCCCG
ATGATTCTGGAGATCCTTCAGGGTCTGAAAAAAGCACTGGGTATAGGAAGAATCGACCGATGAATTTGGCGT
TGGCTCAATCTCCTTTGTTCATGATTCCCTCTGGTTTCAGTCCTTCTGGGTTCCTTAATTCTCCTGGATTTCTTTC
CCCACTCCAGAGTCCCTTTGGAATGTCCCATCAGCAGGCTCTAGCACATGTTACTGCACAGGCTGAATGTTCT
AGTTCATACATGCAAATGCAAGCCGAAGATCAGTGTTCCGCTCAGGTGGCTTCAGCAGAAGCAGCATTAGGA
AATGAGTTGTTGACTGATCCAAAGGAATCTTCTTTGCAGATAAAGGAATGTTTGCAGCCTAGATTGGATAAGA
AACCATCAGACAAGCAGGGTAAGCAATTTGAACTGACGGAGGTTCCTCAATTTGAGAACAAGACATCCTTTG
GTGCTTTCGACAAGTCAGCTTGTGATGGTTATAACTGGAGAAAATATGGCCAGAAAAAGGTTAAGGCTACTG
AATGCCCTCGGAGCTACTATAAGTGCACGCATCTCAAATGTCCAGCGAAGAAGAAGGTTGAGAAATCCGTTG
ATGGTCACATAACTGAGATCACATACAATGGCCGGCACAACCATGCTCAACCAACCAAACAAAGAAAAGATG
GTTCTGCTTTGGATAGTACAGACGGCTCCGGAGTTCAACCAGACATTAGTACACATGATTGGACAGTAATGAA
TAGCTCGGATGGGTCTTCTCCTTCTCATTCTGAACAGGTTCCAAACCAAATGGCATCTGAACTTGTGAAAAAA
GAATGTGATGAAACCAAAAGCAATTTGATAGAAGTAGATGAGGGGCATGATGAACCAGATGCAAAGAGAAC
AAGTGAAGTTGATATCTTAGATGATGGGTACAGATGGCGAAAATATGGGCAGAAGGCGGTAAAGGGGACTC
AACATCCAAGGAGTTACTATCGTTGCACATATGCTGGATGCAATGTCCGCAAACAAGTCGAGAGGGCTTCAA
CTGATCCAAAAGCTGTCATAACAACATATGAAGGCAAGCATAATCATGATATTCCTACTGTTATTAGGAATAG
AGGAACGAGGAATACAGCCAAAGATACTTGGCGTTGA
>CA02g27910
ATGGGAGAAACCGGGGGAGAAGCTTCAGCAATATCATTTCCAGCTTTAACAATTCCACCACGGCCCTCGTAC
GAATCCTTTTTTAACATGTCCTCTTTCAGCCCAGGTCCAATGTCCTTAGTTTCCAGTTTCTTATCCGAACAGAG
CCCTGATTCTGCAGACCGTCCTTCTTTCTCCCAGCTTCTTGCTGGAGTCATCGCTTCACCGACACTTTTGCCCG
ATGATTCTGGAGATCCTTCAGGGTCTGAAAAGAGCACTGGGTATAGGAAGAATCGACCGATGAATTTGGCGT

TGGCTCAATCTCCTTTGTTCATGATTCCCTCTGGTTTCAGTCCTTCTGGGTTCCTTAATTCTCCTGGATTTCTTTC
CCCACTCCAGAGTCCCTTTGGAATGTCCCATCAGCAGGCTCTAGCACATGTTACTGCACAGGCTGAATGTTCT
AGTTCATACATGCAAATGCAAGCCGAAGATCAGTGTTCCGCTCAGGTGGCTTCAGCAGAAGCAGCATTAGGA
AATGAGTTGTTGACTGATCCAAAGGAATCTTCTTTGCAGATAAAGGAATGTTTGCAGCCTAGATTGGATAAGA
AACCATCAGACAAGCAGGGTAAGCAATTTGAACTGACGGAGGTTCCTCAATTTGAGAACAAGACATCCTTTG
GTGCTTTCGACAAGTCAGCTTGTGATGGTTATAACTGGAGAAAATATGGCCAGAAAAAGGTTAAGGCTACTG
AATGCCCTCGGAGCTACTATAAGTGCACGCATCTCAAATGTCCAGCGAAGAAGAAGGTTGAGAAATCCGTTG
ATGGTCACATAACTGAGATCACATACAATGGCCGGCACAACCATGCTCAACCAACCAAACAAAGAAAAGATG
GTTCTGCTTTGGATAGTACAGACGGCTCCGGAGTTCAACCAGACATTAGTACACATGATTGGACAGTAATGAA
TAGCTCGGATGGGTCTTCTCCTTCTCATTCTGAACAGGTTCCAAACCAAATGGCATCTGAACTTGTGAAAAAA
GAATGTGATGAAACCAAAAGCAATTTGATAGAAGTAGATGAGGGGCATGATGAACCAGATGCAAAGAGAAC
AATGTCCCATCCTTCTGTTGTAAAACAAGAAGAAAAATTGTACAGCAACAAAAGTATGTATCTAATTCAGTTA
CTATGTTGCAGGAAGATGGCAGTTGAGACTCTAGCTTCATCACATGGCACAGTAGCTGAATCCAAGATTATTC
TGCAGACAAGAAGTGAAGTTGATATCTTAGATGATGGGTACAGATGGCGAAAATATGGGCAGAAGGCGGTA
AAGGGGACTCAACATCCAAGGAGTTACTATCGTTGCACATATGCTGGATGCAATGTCCGCAAACAAGTCGAG
AGGGCTTCAACTGATCCAAAAGCTGTCATAACAACATATGAAGGCAAGCATAATCATGATATTCCTACTGTTA
TTAGGAATAGAGGAACGAGGAATACAGCCAAAGATACTTGGCGTTGA

CaWRKY14

>Capana02g003661 [mRNA] locus=Chr02:163357641-163359108
ATGGAGAATAACCAGCCCATGCTCCTTCTTGGTTCAGCAAGTAGTTATTACAACTCTATGAATGGAGGATTAA
AGACATCGTTCACCCAGATTAGTCGTGATCAGATGGAAGTGGATACATCAGAAAATCATAATAAATATATAT
CATCATTATCGGTTAAGAAGAAGGGAGATAATAAGAAAATTAAGAAGCCCAGATTTGCTTTCCAAACAAGAA
GTCAGGTTGATATACTTGATGATGGCTATCGTTGGAGAAAATATGGACAAAAAGCTGTCAAGAACAACAATT
ACCCAAGAAGCTACTACAGATGTACACATGAAGGATGCAATGTGAAGAAGCAAGTACAACGCCTTTCCAAAG

ATGAAGGAGTTGTAGTGACCACTTATGAAGGCATGCACACCCATCCTATTGACAAGCCCAATGATAATTTTGA
ACAAATCCTCCATCAGATGCAGATTTTCCCTAATCATCCCCTTAATTAA
>CA02g30960
ATGCTCCTTCTTGGTTCAGCAAGTAGTTATTACAACTCTATGAATGGAGGATTAAAGACATCGTTCACCCAGA
TTAGTCGTGATCAGATGGAAGTGGATACATCAGAAAATCATAATAAATATATATCATCATTATCGGTTAAGAA
GAAGGGAGATAATAAGAAAATTAAGAAGCCCAGATTTGCTTTCCAAACAAGAAGTCAGGTTGATATACTTGA
TGATGGCTATCGTTGGAGAAAATATGGACAAAAAGCTGTCAAGAACAACAATTACCCAAGAAGCTACTACAG
ATGTACACATGAAGGATGCAATGTGAAGAAGCAAGTACAACGCCTTTCCAAAGATGAAGGAGTTGTAGTGAC
CACTTATGAAGGCATGCACACCCATCCTATTGACAAGCCCAATGATAATTTTGAACAAATCCTCCATCAGATG
CAGATTTTCCCTAATCATCCCCTTAATTAA

CaWRKY15
>Capana03g000473 [mRNA] locus=Chr03:6501568-6503375
ATGGAATTCACCAGTTTAGTTGATACTTCTTTGGATCTCAGCTTCAGACCTCTTCGAGTTCCTGATGATATACC
GAAACAAGAAGTTGAGAGTAATTTCATTGGGCTTGGAAGAGATCTCATGCCTGACAAAGATGATCAGGCAGG
TGATTTGTTGGAGGAACTAAATAGAGTAAGTGCTGAAAACAAGAAACTGACTGAGATGTTAACAGTTATGTG
CCAGAATTACAATGCATTGAGAAACCAATTGACTGAATATTTGAACAAGCAAAACAGTACTACTAGTACTGC
AGCTGATAATAATCATGATCATCATAGCGATGGATCGAAAAAAAGAAAAGTCGAAAACAACAACAATGAAA
TTGTGAAATCAGTTCAAGGATTACACTCAGAGAGCAGCTCAAGTGATGAAGATTCATCTAACAAGAAACCAA
GAGAACAACACATTAAAACTAACACTTGTAGAGTTTATGTCAAAACCGAAGCATCTGATACTTCTCTTATTGT
GAAGGATGGATATCAGTGGAGGAAATATGGTCAGAAAGTAACAAGAGATAACCCATCTCCCAGAGCTTATTT
CAAATGCTCTTTTGCTCCTACCTGCCCAGTCAAAAAAAAGGTGCAAAGAAGCTTGGAAGACCAATCGATTCTA
GTAGCGACATATGAAGGAGAACACAACCATTCCAAAATGGATGGTTCAGGCCCCGTTACAACTTCCCCGTCT
AGCCGATTAAACCCGAAAAATACTCTTGTGGGTGCTAATACTACTACTGTCATGCCATGCTCCAGTACTAGTA
TCATCAACACACCATCAGGACCAACCTTAACATTGGATCTTACACAACCAAAAAAATTACAAAATGACGAAA

AGAAAGTGAACAGCAATACTAGTACTAGTAATGCAAGTGGTCAAAAAAGCAAATCACCAGGAGGACATGAT
CATCATCAGCAAAATAGACCAGAGTTTCAACAGTTGTTTATAGATCAAATGGCTTCTTCATTGACTAAAGATC
CAAGTTTTCAAGCAGCCTTAGCCGCTGCCATATCGGGAAAATTCTTACAAAATAATCATACAGACAAATAA
>CA03g32070
ATGGAATTCACCAGTTTAGTTGATACTTCTTTGGATCTCAGCTTCAGACCTCTTCGAGTTCCTGATGATATACC
GAAACAAGAAGTTGAGAGTAATTTCATTGGGCTTGGAAGAGATCTCATGCCTGACAAAGATGATCAGGCAGG
TGATTTGTTGGAGGAACTAAATAGAGTAAGTGCTGAAAACAAGAAACTGACTGAGATGTTAACAGTTATGTG
CCAGAATTACAATGCATTGAGAAACCAATTGACTGAATATTTGAACAAGCAAAACAGTACTACTAGTACTGC
AGCTGATAATAATCATGATCATCATAGCGATGGATCGAAAAAAAGAAAAGTCGAAAACAACAACAATGAAA
TTGTGAAATCAGTTCAAGGATTACACTCAGAGAGCAGCTCAAGTGATGAAGATTCATCTAACAAGAAACCAA
GAGAACAACACATTAAAACTAACACTTGTAGAGTTTATGTCAAAACCGAAGCATCTGATACTTCTCTTATTGT
GAAGGATGGATATCAGTGGAGGAAATATGGTCAGAAAGTAACAAGAGATAACCCATCTCCCAGAGCTTATTT
CAAATGCTCTTTTGCTCCTACCTGCCCAGTCAAAAAAAAGGTGCAAAGAAGTGTGGAAGACCAATCGATTCT
AGTAGCGACATATGAAGGAGAACACAACCATTCCAAAATGGATGGTTCAGGCCCCGTTACAACTTCCCCGTC
TAGCCGATTAAACCCGAAAAATACTCTTGTGGGTGCTAATACTACTACTGTCATGCCATGCTCCAGTACTAGT
ATCATCAACACACCATCAGGACCAACCTTAACATTGGATCTTACACAACCAAAAAAATTACAAAATGACCAA
AAGAAAGTGAACAGCAATACTAGTACTAGTAATGCAAGTGGTCAAAAAAGCAAATCACCAGGAGGACATGA
TCATCATCAGCAAAATAGACCAGAGTTTCAACAGTTGTTTATAGATCAAATGGCTTCTTCATTGACTAAAGAT
CCAAGTTTTCAAGCAGCCTTAGCCGCTGCCATATCGGGAAAATTCTTACAAAATAATCATACAGACAAATAA

CaWRKY16

>Capana03g001099 [mRNA] locus=Chr03:18684171-18687268
ATGGAGGCTTCTTTCAAAAAATCAAATATTCATGGACATGTATTCAAAGTGGAGAAGATCAACGCTGATGAT
AAAGGTTTTGTTGAAGATACTAAAGTTCTTAAGTTTAGCAAGAAAAGAGAACTCCATGAGGACCATAAGTCG
AAGTCATCTCAGCTTCAAAAGGATTATCTCACCAGTGACAAGGAGGATGATCAGCTCGAATCAGCCAAAGCC

GATATGGAAGAGGTAATGGAAGAAAATCAAAGGCTGAAGAAGCATTTAGATCGAGTTATGAAGGATTATCA
GAACCTTCAAATGCAATTCCACGAAATTTCTCAAAGAGGTGTCGAAAAATCCAACAACGTTAAACATGATGA
AGCTGAACTTGTGTCCCTTAGCCTAGGAAGAACTTCAAGCGACACAAAAATAGAGTTATCCAAAATCTTGAA
CAAAAAAGAGAATGTAGAGGAAGAAGATAACCTAACCCTAGGATTGGATTGCAAGTTTCAATCGTCTGCGAA
TGCGCCTACAAAATCTTCACCTTCAAATCTCAGCCCGGAGAATAGCTTAGGTGAAGTTAAGGATGAAAAAGG
AACCGAAACATGGCCAGCTCACAAAGGTCTCAAGACGATCAGGGATGAAGAAGATGATGTGGTGCAACAAA
ACCCTACTAAAAGAGCTAAGGTTTCTGTCAGAATTAGATGTGATACCCCAACGATGAACGATGGATGCCAAT
GGAGAAAATACGGACAAAAAATTGCAAAGGGGAACCCATGTCCTAGAGCTTACTATCGTTGCACAGTAGCAC
CATCTTGCCCAGTTAGAAAACAGGTTCAAAGATGCATTCAGGACATGTCAATCTTGATCATCACATATGAAGG
AACACATAACCATCCACTTCCTCTTTCAGCCACATCAATGGCTTTCACCACTTCAGCTGCCGCTTCCATGCTAT
TGTCCGGTTCATCCACCTCCGAATCAGGCTCTACTTAG

CaWRKY17

>Capana03g001962 [mRNA] locus=Chr03:39904102-39904887
ATGGCTGAAAACCAAAACGACTGGGATTTATGGGCAATTGTGAGAAGTTGCTGCAACATGAACAACTCTGTT
CATGATGATGTGATCAGTTTCGACAACGTTAATAGTACCTCTGTTCTTGTCGATCATGGTGTTCATGAGGATCC
TACTCATGGTAACTCCGCTAATAATGCGAGAACATCAACTTCTTTTCAAGAACAAAGTGGTTGCGCTGGTGAT
TTCAGTGACTCGTTCGCCACAGAAAACAAGCACTATTTTGGGTTAGATGAAGTTCTCGGACTTTCCAAGAACG
TTAATACCAACTCAAGAATTGAACCCCACAACCCAGAGAACCAAACTGAATCTATTACAGACACCCCACTTG
TAGAACACGAGAAGAAGAAGAACAAGAACAAGAAGACAAGATATTCATTGTCGAGTTCAAGTATTGAGGCA
GGCAAGGCGTTTCCTTACTGCAGGAAGAGCACTGAAAGATATGAAGTATTGGCTGAGAAATTGAGTGAGGCG
GATCAATGGAGATGGAGAAAGTATGGGATGAAGCGAACTGGCGGTTCACCTTTTCTCAAGAGCTATTATAGG
TGTAATCAGGGTGAAGATTGTCCGGCAAGGAGACATGTGCAGCAAAGCTCAACAGATTCAAACAAGGTGATT
GTAACTTATAGAGGCCAACACAGTCACCCTCCTCCTAACCAACACATTGCAACAGTACAGGGGAACCATAAT
GCTGCTGCACCAGTAGAAGACCCGCCATTTCCCTCTTCTCCCTCTACTTTGCTGTTCAACTAA

>CA03g20260
ATGGCTGAAAACCAAAACGACTGGGATTTATGGGCAATTGTGAGAAGTTGCTGCAACATGAACAACTCTGTT
CATGATGATGTGATCAGTTTCGACAACGTTAATAGTACCTCTGTTCTTGTCGATCATGGTGTTCATGAGGATCC
TACTCATGGTAACTCCGCTAATAATGCGAGAACATCAACTTCTTTTCAAGAACAAAGTGGTTGCGCTGGTGAT
TTCAGTGACTCGTTCGCCACAGAAAACAAGCACTATTTTGGGTTAGATGAAGTTCTCGGACTTTCCAAGAACG
TTAATACCAACTCAAGAATTGAACCCCACAACCCAGAGAACCAAACTGAATCTATTACAGACACCCCACTTG
TAGAACACGAGAAGAAGAAGAACAAGAACAAGAAGACAAGATATTCATTGTCGAGTTCAAGTATTGAGGCA
GGCAAGGCGTTTCCTTACTGCAGGAAGAGCACTGAAAGATATGAAGTATTGGCTGAGAAATTGAGTGAGGCG
GATCAATGGAGATGGAGAAAGTATGGGATGAAGCGAACTGGCGGTTCACCTTTTCTCAAGAGCTATTATAGG
TGTAATCAGGGTGAAGATTGTCCGGCAAGGAGACATGTGCAGCAAAGCTCAACAGATTCAAACAAGGTGATT
GTAACTTATAGAGGCCAACACAGTCACCCTCCTCCTAACCAACACATTGCAACAGTACAGGGGAACCATAAT
GCTGCTGCACCAGTAGAAGACCCGCCATTTCCCTCTTCTCCCTCTACTTTGCTGTTCAACTAA

CaWRKY18

>Capana03g002072 [mRNA] locus=Chr03:44184049-44185319
ATGGACAATTACGGAGCTGACAATACTAATGTTGAGTTTAATAGGATCATAAATGAATTAACACAAGGTCAG
GACCTTGTTCAACAACTCCAGCTCCATCTCAATGCTCCTAACTATAATTCCTCTGCTTCATTCGAAAACACCCG
GGAAATCTTGCTTCACAATATCCAGTCCAAATTTGACAAGGCTCTGTCCATACTCCAATACAATAGTACTACC
GGAGACAATTCTAATTCACCACTAACACATTCTACTTCCCCTGCCATTCCTGTTTTTGGAGTGTCTGATTCTCC
ACGATCGAGCCCTCCTCATAGTGAAGATTCTGATCGTGACCTCGAATCCAAAGATCCTCATGCTACCCGCAAG
AGAAAGAGCACCACTCCACGGTGGACAAAGCAAGTTCAAATTCATCCAGGAGCACCACTTGAAGGGACTCTT
GATGATGGTTTTAGTTGGAGAAAATATGGGCAGAAAGATATTCTTGGTGCCAAACATCCAAGAGGTTACTAC
AGATGCACGCTTCGACATGTCCAAGGTTGTTTGGCCACGAAACAAGTTCAAAGATCCGATGAAGATCCCACC
ATTTTCGAAGTCACTTATCGTGGAAGGCACACTTGCAGTCAAGGTGGTGGTGGTGCTAGTGCCAGTAATG
TGCATCCAGCACCACTACCTTTGGTTGTAACAATACCTCAAAATCAAGAACCAAACTTAGGAAATCATGAAC

AATATCAACTTATCCCCGCACCACATCAAAATTCACCTGAAATTCTCTTGGATTTTCAGAAAAACCTCAGTAT
CTCCAAAGATGACTTCAACTTCAATACTCACCATGATCATCCTAACAATGTGCCATATATTCCCCCGTATAGT
AATTTCCCTTCATCATCGTCGTCCCACGTTAACACTGATCATCAAGATTACACCTTTGTGGCTAATTCCTCTAC
AATTCCAATCAACAACTTTGTGGAGAATTTTCCTCCTTCCTCTAATAATATGTCTGCAGGAACTTCTCAAATGA
ACAATGCAGACTACCAATTTAACTCAATGGGTTTTGAATCAAACTTCCCGTATAATTATCAAGGATTCTCCTC
CTAA

>CA03g19220
ATGGACAATTACGGAGCTGACAATACTAATGTTGAGTTTAATAGGATCATAAATGAATTAACACAAGGTCGG
GACCTTGTTCAACAACTCCAGCTCCATCTCAATGCTCCTAACTATAATTCCTCTGCTTCATTCGAAAACACCCG
GGAAATCTTGCTTCACAATATCCAGTCCAAATTTGACAAGGCTCTGTCCATACTCCAATACAATAGTACTACC
GGAGACAATTCTAATTCACCACTAACACATTCTACTTCCCCTGCCATTCCTGTTTTTGGAGTGTCTGATTCTCC
ACGATCGAGCCCTCCTCATAGTGAAGATTCTGATCGTGACCTCGAATCCAAAGATCCTCATGCTACCCGCAAG
AGAAAGAGCACCACTCCACGGTGGACAAAGCAAGTTCAAATTCATCCAGGAGCACCACTTGAAGGGACTCTT
GATGATGGTTTTAGTTGGAGAAAATATGGGCAGAAAGATATTCTTGGTGCCAAACATCCAAGAGGTTACTAC
AGATGCACGCTTCGACATGTCCAAGGTTGTTTGGCCACGAAACAAGTTCAAAGATCCGATGAAGATCCCACC
ATTTTCGAAGTCACTTATCGTGGAAGGCACACTTGCAGTCAAGGTGGTGGTGGTGGTGCTAGTGCCAGTAATG
TGCATCCAGCACCACTACCTTTGGTTGTAACAATACCTCAAAATCAAGAACCAAACTTAGGAAATCATGAAC
AATATCAACTTATCCCCGCACCACATCAAAATTCACCTGAAATTCTCTTGGATTTTCAGAAAAACCTCAGTAT
CTCCAAAGATGACTTCAACTTCAATACTCACCATGATCATCCTAACAATGTGCCATATATTCCCCCGTATAGT
AATTTCCCTTCATCATCGTCGTCCCACGTTAACACTGATCATCAAGATTACACCTTTGTGGCTAATTTCTCTAC
AATTCCAATCAACAACTTTGTGGAGAATTTTCCTCCTTCCTCTAATAATATGTCTGCAGGAACTTCTCAAATGA
ACAATGCAGACTACCAATTTAACTCAATGGGTTTTGAATCAAACTTCCCGTATAATTATCAAGGATTCTCTTCC
TAA

CaWRKY19

>Capana03g002134 [mRNA] locus=Chr03:48509522-48510754
ATGGATGACAACAATTGGGATTTAGGCGCAGTTATAAGAAATTGTGGAATTAATAGACCTAGTAATGATATA
ACTCCTAACTTAGGCTCCGAATCATTAAATTTTGATGATGATGATTTGAATTTTCTTGACAGAATTTTTGGTGT
TGACAACAATAATTTTGATTACATAGCTCCTACAAATTTTTCAATTTCTCGTCAGGAAAAATCATATGATCAAT
TTGATGACATCATAAACCCCACAACTCCTGTTTCTATAATTGCTAATCCATTTAAAATCACTAACCAAAATGA
TAACCAACAAATATACTTACCACCCATTCAACCAGCTCAAGTTAGCCAACAAGTTTTTCTTTCTTCACCATCAG
GTGTTGAAGCACGAGAGTGTGTGCCAACAACAACAACTACTACAATATCACATGAATGTATAAATCTGCAAC
AACAACTCTTGATGTGGGATTCCACCTTGACCATGAGGAATCCTCCGATTCAAATTCGTAAAAGTAAAAATCA
GTCGATATGGACTACATATGAATTGTTTCAAGAGGAACTCACAGATGATATATGGTCATGGCGCAAGTATGGT
CAGAAGTTTATCAAAGGTTCTTCATTTCCAAGGAACTACTTTAAGTGTAATACATCAGAGCTCTGTCAAGCAA
GGAAACAAATTGAAAAAAGTTCAAAGAATGATTGTTTTTTCTTGGTAGCCTATTCCGGTATGCATAACCATGA
TCCACCCATAATTCGTAGATCTTTTTCTGATTGGAACCATAGTTCCAAATATAAGCTTCCAAAAGGCATAAAT
ATTATTCCCAAAGCATTAAAATTGAATGCATCACCTTTCTCATCAAAGAGTGGTAAACGTTATAGAGCTTCAT
CTACACTTGAAACTGAGAGCACATCTCGCAAAAAAAATAAAATGATTGTTGAAACCATGAAGAACAATGTTG
ATGATGAGGAGGAGGAGAATATTAATGAAGATGTTCCCAAGGGATTTGAAGAACTCAACTAA
>CA03g25570
ATGGATGACAACAATTGGGATTTAGGCGCAGTTATAAGAAATTGTGGAATTAATAGACCTAGTAATGATATA
ACTCCTAACTTAGGCTCCGAATCATTAAATTTTGATGATGATGATTTGAATTTTCTTGACAGAATTTTTGGTGT
TGACAACAATAATTTTGATTACATAGCTCCTACAAATTTTTCAATTTCTCGTCAGGAAAAATCATATGATCAAT
TTGATGACATCATAAACCCCACAACTCCTGTTTCTATAATTGCTAATCCATTTAAAATCACTAACCAAAATGA
TAACCAACAAATATACTTACCACCCATTCAACCAGCTCAAGTTAGCCAACAAGTTTTTCTTTCTTCACCATCAG
GTGTTGAAGCACGAGAGTGTGTGCCAACAACAACAACTACTACAATATCACATGAATGTATAAATCTGCAAC
AACAACTCTTGATGTGGGATTCCACCTTGACCATGAGGAATCCTCCGATTCAAATTCGTAAAAGTAAAAATCA
GTCGATATGGACTACATATGAATTGTTTCAAGAGGAACTCACAGATGATATATGGTCATGGCGCAAGTATGGT
CAGAAGTTTATCAAAGGTTCTCCATTTCCAAGGAACTACTTTAAGTGTAATACATCAGAGCTCTGTCAAGCAA

GGAAACAAATTGAAAAAAGTTCAAAGAATGATTGTTTTTTCTTGGTAGCCTATTCCGGTATGCATAACCATGA
TCCACCCATAATTCGTAGATCTTTTTCTGATTGGAACCATAGTTCCAAATATAAGCTTCCAAAAGGCATAAAT
ATTATTCCCAAAGCATTAAAATTGAATGCATCACCTTTCTCATCAAAGAGTGGTAAACGTTATAGAGCTTCAT
CTACACTTGAAACTGAGAGCACATCTCGCAAAAAAAATAAAATGATTGTTGAAACCATGAAGAACAATGTTG
ATGATAAGGAGGAGGAGAATATTAATGAAGATGTTCCCAAGGGATTTGAAGAACTCAACTAA

CaWRKY20

>Capana03g002635 [mRNA] locus=Chr03:93204626-93207341
ATGGAGGAGCTTGTTGATGAAACCCCCCGTAAAAGATTGATCAAAGAACTTGTTGAAGGAAAAAGCTTTGCA
AAGCAACTTCAAAGTCTACTTCAACAACCTAATATTGAACATTATGATGGATCAGTCTTAGCTGATGAACTTG
TTCTCAAAATCTGGAGATCTTTTACTCAGGCTATTACTGAGTTAAATACGTTGGTTGATTCCAACAGTATTTTA
GTCCAGACCCAGATGGAGGTGGAAAAGACCGAAGAGGTGGATCAAGCCGATACCGGCGACCGGTCTAATTCT
GAATTGAAGAAGAAGGGCAAACAAGGGGGAAAAGACCGGAGAGGTTGCTACAAGAGAAGAAACAACTCAG
GTTCATGGATGAGAGAATCCGAAACAATGAATGATGGTTGTGCATGGAGGAAATACGGGCAGAAGAGTATA
CTCAACTCAAAATATCCTAGGTGCTACTACAGGTGCACCCACAAGTACGATCAAGATTGCCGGGCCACAAAA
CAAGTTCATATAATGCAAGAAAATCCAAAACTAATGTACCACACCACATACTTTGGCAACCACACTTGCAATC
CAGCAAAGATTCGGAAGCATATAAATAATGCACAATTCAATCATTCTATGCTGGAATGTCCACCTTTTGAAGT
AAAACCAAAAATCCCTAGCAGTGTCACTCATGATTCAACAGAAGAAGAAGAAGAATCATTAAAGGGACAAA
GTGATAATGTATCATCAACTATGGATTCTTACTTATGGGAAGATTTTATGCCTTCTTCTCCCTCAGCTCATGAT
TCCACCTTGGCTTCTCATAATTCTTCTTATTTTCAAGGACTGATTAGTAGTGAGATGGGAGATCTTGTTAAATT
TAGTGACTTTGAGGCCATAGAGTTTTTTTGA
>CA03g12230
ATGGAGGAGCTTGTTGATGAAACCCCCCGTAAAAGATTGATCAAAGAACTTGTTGAAGGAAAAAGCTTTGCA
AAGCAACTTCAAAGTCTACTTCAACAACCTAATATTGAACATTATGATGGATCAGTCTTAGCTGATGAACTTG
TTCTCAAAATCTGGAGATCTTTTACTCAGGCTATTACTGAGTTAAATACGTTGGTTGATTCCAACAGTATTTTA

GTCCAGACCCAGATGGAGCCGATAACNNNNNNNNNTAATTCTGAATTGAAGAAGAAGGGCAAACAAGGGGG
AAAAGACCGGAGAGGTTGCTACAAGAGAAGAAACAACTCAGGTTCATGGATGAGAGAATCCGAAACAATGA
ATGATGGTTGTGCATGGAGGAAATACGGGCAGAAGAGTATACTCAACTCAAAATATCCTAGGTGCTACTACA
GGTGCACCCACAAGTACGATCAAGATTGCCGGGCCACAAAACAAGTTCATATAATGCAAGAAAATCCAAAAA
TAATGTACCACACCACATACTTTGGCAACCACACTTGCAATCCAGCAAAGATTCGGAAGCATATAAATAATG
CACAATTCAATCATTCTATGCTGGAATGTCCACCTTTTGAAGTAAAACCAAAAATCCCTAGCAGTGTCACTCA
TGATTCAACAGAAGAGGAAGAAGAATCATTAAAGGGACAAAGTGATAATGTATCATCAACTATGGATTCTTA
CTTATGGGAAGATTTTATGCCTCCTTCTCCCTCAGCTCATGATTCCACCTTGGCTTCTCATAATTCTTCTTATTT
TCAAGGACTGATTAGTAGTGAGATGGGAGATCTTGTTAAATTTAGTGACTTTGAGGCCATAGAGTTTTTTTGA

CaWRKY21

>Capana03g003085 [mRNA] locus=Chr03:167540037-167548534
ATGGAAGATTCTCACTCTCACTCTCACTACCCTCGCCCCTATAGCAACTCAGCTCCACTCTCTTCCATCAATGA
AACTTCTGAGCAAGTCAAATTTTCAAGCTCCGATGCTGCGCTCTTCTCCAGCTCTGATGCTGCGTTCGTCTACT
CCTCCGCCTTTGGATCGAACAGTAGTAGCAGTGCTAAGTACAAACTGATGTCACCTGCTAAGCTCCCGATCTC
TCGCTCCCCGTGTATAACTATCCCTCCTGGTCTCAGTCCTTCTTCCTTTCTCGAATCTCCCGTTCTTCTTTCTAA
CATCAAAGCTGAGCCTTCTCCGACCACAGGTTCCTTCTCCAAGTTTCAACTAATGCAAGGCTCTAGTGGGAGT
GCTGCTTTTTCATTGATGAGAAGCTGTTCCAGTGGAAATGCATATGGTGAAACGACCGGCGAATTTGAGTTTG
AATTTCCTATTGGATCTAGCTCTACATCAGGATCACTGGCAAAGGAAGCTGTGATTTGTGCAGGTTTCAACCA
ACAGCAAAGTGAACCACTGATTCAAGTTCAAAATCGATGTCCTTCTCAATCATTAGCACCTCCAGCTCTTGTT
AAAAGTGAGATGCCTAATTCAAAAGAGCTGAGTCTACCTACACCTGTTTGTTTAGATGCCTCGTTAATTAGTA
CTGCTGCTGCTGCAACTGATAATGAGGAGGTAAATCAAAGAGGTCAATCAAATCCAAGCTCGCACAGGTCAT
CTGCTGATAATAAAAATGTATCATCAGTAACAGCTGATAGATCGTCTGAAGATGGGTATAACTGGAGAAAGT
ATGGCCAGAAACTTGTCAAAGGAAGTGAATTCCCAAGGAGCTACTACAAATGTACATACCCAAACTGTGAAG
TTAAAAAGATATTTGAGCGCCCTCCTGATGGACAAATAACAGAGATTGTGTATAAAGGTTCCCATGATCATCC

TAAACCCCAACCCAACCATAGGTTTACTCCTGGTGCTCTTACGTCCGTCCAAGAAGACAGAGGTGAGAGAGA
AGCATGCCTCACTGGTCAAGAAGACAAATTCAACACTAATGCCCAGACTAGTAACACTGAGCCCAGTGGTAC
TCCACTATCACCTCAACAAGCAGATGATGATGGTCTTGAAGGTACAGTGTCTCAGTTGCACAGCTCTAATGAT
CAGATGGACGAAGATGATTCATTTGCAAAAAGAAGGAAAATGGATGGTGTCATGGATATTATACCAGTTGTT
AAGCCTATCCGTGAACCACGTGTTGTTGTTCAAACTGTGAGTGAAGTTGATATATTGGATGATGGATACAGGT
GGCGGAAGTACGGTCAAAAGGTGGTACGTGGTAATCCTAATCCCAGGAGTTATTACAAGTGCACCAATGCTA
GATGCCCTGTCAGGAAACATGTGGAGAGGGCATCCCATGATCCCAAAGCTGTAATTACTACATATGAGGGGA
AACACAATCATGATGTACCAACAGCCAGGACTAATAGCCATGAAATGGCAGGATCAGCGCCTGTAACTGGGA
GTTCTAGGGTCAGAGTGGAAGAGAATGGTGCAATTAGCTTGGATCTAGGTGTTGGTATAGGACATGGCATGG
AGAATAGACGCAATGGACAACTTCACACACTGCCTGCAGAAACTGTACGTAGCCAAGGTCAGGTTTCAAGTT
CTATTGTGATGGTAGTACAGCCAGCTGCAGTTGCGGCATGCTACAGCATTGTAAATGGTGGCATGAACCGTTT
TGGAACTATAGAAAATCGTGTTCAGGGCACTGGCTTTGAGACTCTGCCCTTGCAATCTTCTGCTCAATATCCTC
AAAACTATGGAATGATACTTTTGGGCCCATGA
>CA03g12030
ATGCCTAATTCAAAAGAGCTGAGTCTACCTACACCTGTTTGTTTAGATGCCTCGTTAATTAGTACTGCTGCTGC
TGCAACTGATAATGAGGAGGTAAATCAAAGAGGTCAATCAAATCCAAGCTCGCACAGGTCATCTGCTGATAA
TAAAAATGTATCATCAGTAACAGCTGATAGATCGTCTGAAGATGGGTATAACTGGAGAAAGTATGGCCAGAA
ACTTGTCAAAGGAAGTGAATTCCCAAGGAGCTACTACAAATGTACATACCCAAACTGTGAAGTTAAAAAGAT
ATTTGAGCGCCCTCCTGATGGACAAATAACAGAGATTGTGTATAAAGGTTCCCATGATCATCCTAAACCCCAA
CCCAACCATAGGTTTACTCCTGGTGCTCTTACGTCCGTCCAAGAAGACAGAGGTGAGAGAGAAGCATGCCTC
ACTGGTCAAGAAGTTATTCCTCTCTCTGGTTTATCTTTTCTTGAAGACAAATTCAACACTAATGCCCAGACTAG
TAACACTGAGCCCAGTGGTACTCCACTATCACCTCAACAAGCAGATGATGATGGTCTTGAAGGTACAGTGTCT
CAGTTGCACAGCTCTAATGATCAGATGGACGAAGATGATTCATTTGCAAAAAGAAGCAGGAAAATGGATGGT
GTCATGGATATTATACCAGTTGTTAAGCCTATCCGTGAACCACGTGTTGTTGTTCAAACTGTGAGTGAAGTTG
ATATATTGGATGATGGATACAGGTGGCGGAAGTACGGTCAAAAGGTGGTACGTGGTAATCCTAATCCCAGGA

GTTATTACAAGTGCACCAATGCTAGATGCCCTGTCAGGAAACATGTGGAGAGGGCATCCCATGATCCCAAAG
CTGTAATTACTACATATGAGGGGAAACACAATCATGATGTACCAACAGCCAGGACTAATAGCCATGAAATGG
CAGGATCAGCGCCTGTAACTGGGAGTTCTAGGGTCAGAGTGGAAGAGAATGGTGCAATTAGCTTGGATCTAG
GTGTTGGTATAGGACATGGCATGGAGAATAGACGCAATGGACAACTTCACACACTGCCTGCAGAAACTGTAC
GTAGCCAAGGTCAGGTTTCAAGTTCTATTGTGATGGTAGTACAGCCAGCTGCAGTTGCGGCATGCTACAGCAT
TGTAAATGGTGGCATGAACCGTTTTGGAACTATAGAAAATCGTGTTCAGGGCACTGGCTTTGAGACTCTGCCC
TTGCAATCTTCTGCTCAATATCCTCAAAACTATGGAATGATACTTTTGGGCCCATGA

CaWRKY22

>Capana03g003279 [mRNA] locus=Chr03:203453030-203454904
ATGGAGGAGATTGAGGAAGCTAACAGGGTGGCAGTTGAGAGTTGTCATAGAGTTATTACTATGTTATCACAA
CCCCATGATCAAAAACAGTTTGGAAATGTAGCAAGAGAGACTGGAGAGGCTGTACACAAGTTCAAGAAAGT
GGCGACACTTCTAAATTCCAATTTAGGTCATGCAAGAGTAAGAAAGGCCAAGAAAATTATAACCCCTTTACCT
CAAAACCTCTTGTTAGAGAGCCCAAGTTGCAAAACTTATGATCAGCCTAAATCCCTACAGTTACTGCCTATCA
CAGAAATTGGTTCTAATGTGAAAAGCACTCTTACTTTAGCCAACCCTTCACTAGAATTAAGCTCACATAGTAA
AAATCCTCTTCAGTTAGCTCAACAAACACCGTTGTCAAGCTATCACTTTCTCCAACAACAGCAACAGAGGAGG
TATCAACTTCAGCAGCAACAGTTAAAGCAGCAGACAGATATGATGTACCGGCGAAGCAATAGTGGGATTAGC
CTAAATTTTGATAGCTCTACTTGTACGCCAACTATGTCATCCACTAGATCGTTTATTTCCTCTTTGAGTATTGA
CGGAAGTGTTGCTAACTTGGATGGTAATGCAAATGCCTTCCATTTTATTGGGGCATCACGCTCTGCGGATCAG
AGCTCATTTCAACACAGGAAAAGGTGCTCAGGGAGGGGAGAGGAGGGAAGTGTGAAATGTGGAAGCAGTGG
TAGATGTCACTGTTCCAAGAAGAGGAAGCACAGGGTGAAGAGGTCAATCAAGGTGCCTGCTATAAGTAACAA
GCTAGCTGATATTCCCCCGGATGAGTATTCATGGAGAAAGTATGGACAGAAGCCAATCAAAGGTTCTCCGCA
CCCTAGGGGATACTACAAGTGTAGCAGCATGAGAGGCTGTCCTGCCAGGAAACACGTTGAGAGATGCTTGGA
AGAACCTTCGATGCTTATTGTCACTTACGAAGGAGAACATAACCATTCTAGGTTGCCAT
>CA06g07080

ATGGAGGAGATTGAGGAAGCTAACAGGGTGGCAGTTGAGAGTTGTCATAGAGTTATTACTATGTTATCACAA
CCCCATGATCAAAAACAGTTTGGAAATGTAGCAAGAGAGACTGGAGAGGCTGTACACAAGTTCAAGAAAGT
GGCGACACTTCTAAATTCCAATTTAGGTCATGCAAGAGTAAGAAAGGCCAAGAAAATTATAACCCCTTTACCT
CAAAACCTCTTGTTAGAGAGCCCAAGTTGCAAAACTTATGATCAGCCTAAATCCCTACAGTTACTGCCTATCA
CAGAAATTGGTTCTAATGTGAAAAGCACTCTTACTTTAGCCAACCCTTCACTAGAATTAAGCTCACATAGTAA
AAATCCTCTTCAGTTAGCTCAACAAACACCGTTGTCAAGCTATCACTTTCTCCAACAACAGCAACAGAGGAGG
TATCAACTTCAGCAGCAACAGTTAAAGCAGCAGACAGATATGATGTACCGGCGAAGCAATAGTGGGATTAGC
CTAAATTTTGATAGCTCTACTTGTACGCCAACTATGTCATCCACTAGATCGTTTATTTCCTCTTTGAGTATTGA
CGGAAGTGTTGCTAACTTGGATGGTAATGCAAATGCCTTCCATTTTATTGGGGCATCACGCTCTGCGGATCAG
AGCTCATTTCAACACAGGAAAAGGTGCTCAGGGAGGGGAGAGGAGGGAAGTGTGAAATGTGGAAGCAGTGG
TAGATGTCACTGTTCCAAGAAGAGGAAGCACAGGGTGAAGAGGTCAATCAAGGTGCCTGCTATAAGTAACAA
GCTAGCTGATATTCCCCCGGATGAGTATTCATGGAGAAAGTATGGACAGAAGCCAATCAAAGGTTCTCCGCA
CCCTAGGGGATACTACAAGTGTAGCAGCATGAGAGGCTGTCCTGCCAGGAAACACGTTGAGAGATGCTTGGA
AGAACCTTCGATGCTTATTGTCACTTACGAAGGAGAACATAACCATTCTAGGTTGCCATCTCAATCGGCAAAT
GCATGA

CaWRKY23

>Capana04g000568 [mRNA] locus=Chr04:9062869-9064395
ATGGCTGTAGAATTAATGTCCACTGGTTATAGACCCAATAATTTCTCATCAAAAATGGAAGAAAATTCCGTGC
AAGAAGCTGCTGCTGCCGGCCTTCAAAGCGTTGAGAAATTAATCCGGTTGCTTTCTCAATCCCAACAACAACA
ACAACAAAATCATCAGCAACAAACAAATTTTCAGAATTCATCGTCGAATGATTATCAGGTTGTTGCTGATGTT
GCTGTTAACAAATTCAAAAAGTTCATTTCGTTACTTGACAAAAATCGTACTGGACATGCCAGATTTCGTCGTG
GTCCAATTACTTCTCCTCCTCCACCCCCACCTCCGCCGCCAGTTCCAGCTAAACCCCAGCAGAACAAGCAACA
AATGGAGGAATCTGAAAAGCAACAATCTAGTGCTACAAAAATTTACTGTCCAACTCCCATACAAAGATTACC
ACCGTTACCACACAACCACCATCAACAAATACTAATCAAAAATGGATCGATCGAGAGAAAAGAAGCAGCGTC

ATCTACAACTATTAATTTTGCTTCTCCGTCACCGGCAACTTCCTTCATGTCGTCGTTAACAGGAGAAACGGAG
AGTTTACAGAACTCTTTGTCTTCCGGATTTCAAATTACGAATCTTTCTCAGGTCTCTTCCGCCGGCCGGCCGCC
GATTTCGACTTCTTCATTCAAGAGAAAGTGTAGTTCTATGGACGATATTGCCCTCAAGTGTAACAGCGCCGGT
GGATCCTCCGGCCGTTGCCACTGCCCTAAGAAAAGGAAATCAAGAGTCAAAAGGGTAGTGAGAGTTCCTGCA
ATTAGCATGAAAATGGCTGATATTCCACCTGATGATTATTCATGGAGAAAGTATGGTCAAAAACCCATCAAG
GGTTCTCCTCATCCCAGGGGATATTACAAATGTAGCAGTGTACGAGGATGTCCTGCACGTAAACATGTAGAA
AGAGCATTAGATGATCCAGCTATGTTGATTGTTACATATGAAGGAGAACACAATCATTCCCATTCCATTACAG
AAACACCAGCAACTCATGTTCTTGAATCATCTTAA
>CA04g18300
ATGTCCACTGGTTATAGACCCAATAATTTCTCATCAAAAATGGAAGAAAATTCCGTGCAAGAAGCTGCTGCTG
CCGGCCTTCAAAGCGTTGAGAAATTAATCCGGTTGCTTTCTCAATCCCAACAACAACAACAACAAAATCATCA
GCAACAAACAAATTTTCAGAATTCATCGTCGAATGATTATCAGGTTGTTGCTGATGTTGCTGTTAACAAATTC
AAAAAGTTCATTTCGTTACTTGACAAAAATCGTACTGGACATGCCAGATTTCGTCGTGCTAAACCCCAGCAGA
ACAAGCAACAAATGGAGGAATCTGAAAAGCAACAATCTAGTGCTACAAAAATTTACTGTCCAACTCCCATAC
AAAGATTACCACCGTTACCACACAACCACCATCAACAAATACTAATCAAAAATGGATCGATCGAGAGAAAAG
AAGCAGCGTCATCTACAACTATTAATTTTGCTTCTCCGTCACCGGCAACTTCCTTCATGTCGTCGTTAACAGGA
GAAACGGAGAGTTTACAGAACTCTTTGTCTTCCGGATTTCAAATTACGAATCTTTCTCAGAGAAAGTGTAGTT
CTATGGACGATATTGCCCTCAAGTGTAACAGCGCCGGTGGATCCTCCGGCCGTTGCCACTGCCCTAAGAAAAG
GAAATCAAGAGTCAAAAGGGTAGTGAGAGTTCCTGCAATTAGCATGAAAATGGCTGATATTCCACCTGATGA
TTATTCATGGAGAAAGTATGGTCAAAAACCCATCAAGGGTTCTCCTCATCCCAGGGGATATTACAAATGTAGC
AGTGTACGAGGATGTCCTGCACGTAAACATGTAGAAAGAGCATTAGATGATCCAGCTATGTTGATTGTTACAT
ATGAAGGAGA

CaWRKY24

>Capana04g001820 [mRNA] locus=Chr04:115696954-115705628

ATGGGGGAAAAACTGAAAGTTCCAGCAGTTTCAGCACTTCCTGCACTAACAATTCCACCTAGGGAAACATTTT
TTGGTGGTGGAAATATGTCTTATTTTAGCCCAGGTCCAATTACCCTTGTGTCCAGCTACTTCTCTGAATCTGAG
CACCCTTCTTTCTCTCAGCTCCTTGCTGGGGCCATGGCTTCTCCACTAGCAAAACCCCTTTTGACTAAAGAAGA
AGAAGCTAATTGTAAAGAGGGGAATTTAGGGTATAAGCAGAATCGACCGATGAGTTTAATGGTGGCTCACTC
TCCTTTCTTTACCCCTTTTAGTCCTTCTGGATTGCTTAATTCACCTGCCTTTCTTTCCCCACTTCAGAGTCCGTTT
GGGATGTCACACCAGCAAGCCCTAGCACATGTTACAGCGCAGGCAGCATTATCCCAGTCTTACCTACAGGGG
ACATCAGCACAAGTATTAGGGACATCTGACCTAGACGAATCTTCTTTACAACCTCAATTAGATACTATGCCGT
CAGACCAGCAGATTAAGAAGTTTGAACTGCCACAGATTTCTCAATCTGAGGAAAAGCCATACCTTAATTCAGT
CGATAAGCCAGCTTCTGATGGTTACAATTGGAGGAAGTATGGCCAGAAGATGGTTAAGGCAAGTGAATGCCC
TCGGAGCTACTATAAATGCACACATGTCAAATGTCCTGTCAGGAAGAAGGTTGAGCGATCCGTTGATGGTCA
CGTAACTGAGATAACATATAAAGGCCATCACAATCACGAGCTTCCTCAACCAAACAAACGCAGAAGAGATAG
TGGTGCTCAGGATGGTTCAGACTGCTCCAAAGCTAACCCTGAAATTGAAACACATACTGAGATAGAAACCAG
CGGCTTAAATGGGGCGCATCTTGCTCATTCTGAGCAGGTGTCTACTGAAAGGGCATCTGAACCTCCAGTTTTG
AAGGATTATGATGAAATTGTGGACACTGCAACAGCAACAGGCAAGGAACAGGATGATGAATCAAATGTGAA
GAGAATGAAGACAACAGTCGAGACTCCTATTCTTTTTTCATCACATAAAGCGGAATCAGAATCCAAGATTGTT
GTGCAGACAAGGAGTGAAGTAGACATTTTGGATGACGGGTTCAAGTGGCGTAAGTATGGGCAAAAAGTGGTG
AAGGGGAATCATCATCCGAGGAGTTATTACCGTTGCACATATCCTGGATGCAATGTTCGCAAACATGTTGAGA
GGGCTTCAACAGACCCAAAAGCTGTCATAACAACATATGAAGGCAAACACAATCATGAAAGTCCCATTGCTA
GAAACAGAAGCCATAGCGCAGCCCAAGATAGTACTTGTCAGTTGAACGAGCAAGAGATTGCAACTTGGAGGC
CTTCACTTCATGAAAAAGTTGCTCTTCATGCCAATGAAATACCAGTGTGTAGGCAGCTGAAAGATGAACATAT
GGCAGCATAA
>CA04g11710
AGTCCGTTTGGGATGTCACACCAGCAAGCCCTAGCACATGTTACAGCGCAGGCAGCATTATCCCAGTCTTACC
TACAGGGGACATCAGCACAAGTATTAGGGACATCTGACCTAGACGAATCTTCTTTACAACCTCAATTAGATAC
TATGCCGTCAGACCAGCAGATTAAGAAGTTTGAACTGCCACAGATTTCTCAATCTGAGGAAAAGCCATACCTT

AATTCAGTCGATAAGCCAGCTTCTGATGGTTACAATTGGAGGAAGTATGGCCAGAAGATGGTTAAGGCAAGT
GAATGCCCTCGGAGCTACTATAAATGCACACATGTCAAATGTCCTGTCAGGAAGAAGGTTGAGCGATCCGTT
GATGGTCACGTAACTGAGATAACATATAAAGGCCATCACAATCACGAGCTTCCTCAACCAAACAAACGCAGA
AGAGATAGTGGTGCTCAGGATGGTTCAGACTGCTCCAAAGCTAACCCTGAAATTGAAACACATACTGAGATA
GAAACCAGCGGCTTAAATGGGGCGCATCTTGCTCATTCTGAGCAGGTGTCTACTGAAAGGGCATCTGAACCTC
CAGTTTTGAAGGATTATGATGAAATTGTGGACACTGCAACAGCAACAGGCAAGGAACAGGATGATGAATCAA
ATGTGAAGAGAATGAAGACAACAGTCGAGACTCCTATTCTTTTTTCATCACATAAAGCGGAATCAGAATCCA
AGATTGTTGTGCAGACAAGGAGTGAAGTAGACATTTTGGATGACGGGTTCAAGTGGCGTAAGTATGGGCAAA
AAGTGGTGAAGGGGAATCATCATCCGAGGTAA

CaWRKY25

>Capana05g002502 [mRNA] locus=Chr05:216311579-216315172
ATGTCGTCGCTGCCACCTTGCCGGACGGATTTCCTGACGATACCGCCCGGCATTAGTCCGGCGGCATTGCTTG
ATTCTACCTTCATCCTCCCAAGTTCAATGACACCTACTGATAATGTTGCACAAATTTTGCATTGCATTGAGTCA
ACAAACAATCAAATCCCTCAGCAACAACAAGAGTTTACTAAGAAACAAAACCAATTTGAGCAAAGAGGCAT
ATTCAGTAGTAGCATTAGTCCTAACAATTCATCAGATGATGGATACACATGGAGAAAATATGGACAAAAGCA
TGTAAAAGGGAGTAATTTTCCCAGAAGTTACTACAAATGCACTCAACAAACATGTCCTGTGAGGAAAAAAGT
TGAGTGTGCACCAAATGGACAAGTCATAGAAATTGTCTACAATGGTCCTCATAATCACCCGAAAACTCAACA
TCTTCGACGAAAAGCTATGGATGCAGATTCCTATGTTGTTGGACAAGAAAATGGATCATCATCATCATCATTG
ATATGGAGAAACGATCAGCAACTTGAGTACAATAAGGATGTTAATAGTTGTTGTAATGAACTAGAAAGAAAA
CCTTCTGCATCTGTCTTGTCTGATGTCTCATCCGATCCAATGTTGTCGAACAATCTGAAATCGATGAATGTGTT
TGAATCAGATGCAACTCATGAGCTTTCTTCTACGCTAAATAGTTTTGATGATGAAGATGAAGATTTGGCCACT
CAAGAAGGTAATTTTCTTGGGGATGGTATCAATGAGTTTGAATTTGAGCCAAAAAGAAGGAAGAAAGAAAGT
TATTCAGTTGAACCAAGTTTACTGTCGAGAACAGTGAGAGAACCGAAAGTAGTCCTCCAGGTGGAGAGTGAG
ACCGATATTCTTGAAGATGGCTATCGCTGGAGAAAATATGGCCAGAAAGTTGTCAAAGGAAACCCTAATCCC

AGGAGCTACTACAAATGTACGAGCGCTGGATGCTTAGTGAGGAAACATGTCGAAAGGGCATCAGATGACTTA
AAATCAGTAATCACAACGTATGAGGGGAAACACAACCATGAAGTGCCATCGGCAAACAAGACGAATGGTGT
TGCAGGACATTTTCGATCAGCATCTATGTTGAACAACGGGCAGCAGCAGCCGGCTTGCACAGCATCTCGTAA
GAGTCTTAAAGATTCAAATATCAGAGTGCAATTTCAAGATCTACCTATTCCGTTCGAAAGAAAGTTTTTCATG
GGAAGTGAATATTTAAGGCCAAATTTTGGTGCAAGTTACCTCAGTGACTTGAGTTTCGGAGGTGGTTCTTTGC
AGCTCCCGGACTTTGCAATACCATTACCATTGCCATCGCGAATGAGTTTTCCTGCAAGACAGAACGAGCCTCG
TCTTGGTGACTTTCAGATGAATAATCATCTCTTGCTGCCAAATGGTGCATCGACATTTTTAGCAGCTGGGAAT
ACACGACATATTAACGATGACAACAATAACTCGAGACTACTTAAAGCCAAAGACGAGGTCCAGTACTGGACC
TAG
>CA05g20090
ATGGAAGCCTTGGAGCAACTGGTGAAGTTGCTCCCATGCGACCAGAGGGTCACTCACGGGTTCAAGCTGTGG
AAACAGCCTCTGCCAGAACTGCAAGATAAGACACCTACTGATAATGTTGCACAAATTTTGCATTGCATTGAGT
CAACAAACAATCAAATCCCTCAGCAACAACAAGAGTTTACTAAGAAACAAAACCAATTTGAGCAAAGAGGC
ATATTCAGTAGTAGCATTAGTCCTAACAATTCATCAGATGATGGATACACATGGAGAAAATATGGACAAAAG
CATGTAAAAGGGAGTAATTTTCCCAGAAGTTACTACAAATGCACTCAACAAACATGTCCTGTGAGGAAAAAA
GTTGAGTGTGCACCAAATGGACAAGTCATAGAAATTGTCTACAATGGTCCTCATAATCACCCGAAAACTCAA
CATCTTCGACGAAAAGCTATGGATGCAGATTCCTATGTTGTTGGACAAGAAAATGGATCATCATCATCATCAT
TGATATGGAGAAACGATCAGCAACTTGAGTACAATAAGGATGTTAATAGTTGTTGTAATGAACTAGAAAGAA
AACCTTCTGCATCTGTCTTGTCTGATGTCTCATCCGATCCAATGTTGTCGAACAATCTGAAATCGATGAATGTG
TTTGAATCAGATGCAACTCATGAGCTTTCTTCTACGCTAAATAGTTTTGATGATGAAGATGAAGATTTGGCCA
CTCAAGAAGGTAATTTTCTTGGGGATGGTATCAATGAGTTTGAATTTGAGCCAAAAAGAAGGAAGAAAGAAA
GTTATTCAGTTGAACCAAGTTTACTGTCGAGAACAGTGAGAGAACCGAAAGTAGTCCTCCAGGTGGAGAGTG
AGACCGATATTCTTGAAGATGGCTATCGCTGGAGAAAATATGGCCAGAAAGTTGTCAAAGGAAACCCTAATC
CCAGGAGCTACTACAAATGTACGAGCGCTGGATGCTTAGTGAGGAAACATGTCGAAAGGGCATCAGATGACT
TAAAATCAGTAATCACAACGTATGAGGGGAAACACAACCATGAAGTGCCATCGGCAAACAAGACGAATGGT

GTTGCAGGACATTTTCGATCAGCATCTATGTTGAACAACGGGCAGCAGCAGCCGGCTTGCACAGCATCTCGTA
AGAGTCTTAAAGATTCAAATATCAGAGTGCAATTTCAAGATCTACCTATTCCGTTCGAAAGAAAGTTTTTCAT
GGGAAGTGAATATTTAAGGCCAAATTTTGGTGCAAGTTACCTCAGTGACTTGAGTTTCGGAGGTGGTTCTTTG
CAGCTCCCGGACTTTGCAATACCATTACCATTGCCATCGCGAATGAGTTTTCCTGCAAGACAGAACGAGCCTC
GTCTTGGTGACTTTCAGATGAATAATCATCTCTTGCTGCCAAATGGTGCATCGACATTTTTAGCAGCTGGGAA
TACACGACATATTAACGATGACAACAATAACTCGAGACTACTTAAAGCCAAAGACGAGGTCCAGTACTGGAC
CTAG

CaWRKY26

>Capana06g001008 [mRNA] locus=Chr06:17571223-17578663
ATGGAGAACAAGAACAAAGCTGAATATTATAGTCCTGATGATGAAGATCAAGAAAATATTATCCATAAGTTT
GGAAAGGGAAGAAAAGAACGTGAGGATGATAAGTCAAAGCCATCCTCCCCTCACCACAAGGATTTCATGGC
AATTGACAATAATATTAAGGGAGTTGCTGTTAATGTTATGGTCAAGAGAGAAAGATCACCCCCAGAACTCAA
CTCAATGGCTTCTTCATCTGCTCACAAAGAAAAGGATGATCAGCTTGCATTAGCCAAAGTTGAAATGCGAGA
AGTAATGGAAGAAAATCAAAGGCTTCGATTTCACTTGGATCGAATCATGAAGGAATATAGAAATCTGCAGAA
TCAATTCCATGATATCGTTCAAAGAGAAGTTGATCAAAAATCAAGCAGTACAGTGAACACTACTCAACATGA
ATCTGATCACGAAACAAATGAACTTGTTTCCCTTAGCCTAGGAAGGGCAACGAGTGATATGAAAAAAGAAGA
ATTATCAAAAATCTTGAAGAAAGATAAAGGTCGTGATGATGAAGATGTTAATAAAAGTCTTGATTTAGGTTTG
GATTGCAAGTTTGAAGAATGTTCGCCTGTGAAGAATCGTAGTCCAGAGAATAGCTTAGATGATCATCAAGCT
AATAAGGACGAAAATGGAGAGACGTCGACGACCACGTGGCCTCCTAACAAAAATCTGAAGACGATGAGGAA
TGATGGAGACAATGGTGATGATGTTTCACAACAAAACCCTACTAAAAGAGCTAGGGTTTCTGTTAGAGTCAG
ATGTGATGCACCTACGATGAATGATGGATGCCAATGGAGAAAATATGGTCAAAAAATTGCAAAAGGAAATCC
ATGCCCTCGAGCTTATTATCGTTGCACTGTAGCACCAAATTGCCCAGTTAGAAAGCAGGTTCAAAGATGTGCT
GAGGACATGTCAATATTGATCACCACATATGAAGGAACACACAACCACACACTCCCTCTTTCCGCCACCGCA
ATGGCTTCCACCACCTCTGCTGCCGCTAACATGTTGTTATCCGGTTCGTCGAGTTCATCAGACCCAAGTCCACA

AATAACTGCCACCACTACCAACACCGCCACCGCTACTACTTCTGCCAATATCAACGGACTCAACTTCTATATC
TCCGATACCTCAAAACATAAGTCGCCTTTTTACTTTCCTAATTCATCCATCTCAGCATCCACACTTAATAATTC
GCACCCTACAATCACTCTCGATTTGACTTCTACGTCCTCCTCCTCATCCTCGTCCTTATCTCATCTTAACAGGAT
GAGTAACAATCTCCACCCTAGATATAATTACAATAACTCCTCCACAAACCTCAATTTTAGTTCAGTGCTAGAA
TCCAATTCCCTTCCCATTTCTTGGACAAATTATCAAAACCAAACCTGCAACAAGAACAACCAAAATTTTGGTT
CACTTAACTTCTCATCAAGACCAAATCAAGAAAATATTTTCCAGTCTTATTTACAAAAAAACAACAACATTAT
CCCTACACAATCTTCTTTTCCACCAGATACAATTGCAGCTGCAACGAAAGCAATAACGTCAGACCCTAATTTC
CACTCAGCATTAGCTGCTGCTCTCACCTCAATCATTGGAAATACTGGAATCGAAAATAAGCCTGGCCATAATT
TCAATGTTTCTGAGCCATTTCCAGTTTTGTCCAGTCTCCCATCAAGCTCAAATCCGAATAAATGCTCGTCAAGT
TTTTTAAATAAACCAACTTCTTCTTCCGCGAATAATAGTTCACAGCAGCCTGGAAATAATAACAACTTGGTGT
TCTTCGCGCAATCTTCTTCTTCTTCATTGCCATTTTCTACGTCCAATAAGGGTAAATCTACCTCTCCTAGTGATA
GCTAG
>CA06g19170
ATGGTCAAGAGAGAAAGATCACCCCCAGAACTCAACTCAATGGCTTCTTCATCTGCTCACAAAGAAAAGGAT
GATCAGCTTGCATTAGCCAAAGTTGAAATGCGAGAAGTAATGGAAGAAAATCAAAGGCTTCGATTTCACTTG
GATCGAATCATGAAGGAATATAGAAATCTGCAGAATCAATTCCATGATATCGTTCAAAGAGAAGTTGATCAA
AAATCAAGCAGTACAGTGAACACTACTCAACATGAATCTGATCACGAAACAAATGAACTTGTTTCCCTTAGCC
TAGGAAGGGCAACGAGTGATATGAAAAAAGAAGAATTATCAAAAATCTTGAAGAAAGATAAAGGTCGTGAT
GATGAAGATGTTAATAAAAGTCTTGATTTAGGTTTGGATTGCAAGTTTGAAGAATGTTCGCCTGTGAAGAATC
GTAGTCCAGAGAATAGCTTAGATGATCATCAAGCTAATAAGGACGAAAATGGAGAGACGTCGACGACCACGT
GGCCTCCTAACAAAAATCTGAAGACGATGAGGAATGATGGAGACAATGGTGATGATGTTTCACAACAAAACC
CTACTAAAAGAGCTAGGGTTTCTGTTAGAGTCAGATGTGATGCACCTACGATGAATGATGGATGCCAATGGA
GAAAATATGGTCAAAAAATTGCAAAAGGAAATCCATGCCCTCGAGCTTATTATCGTTGCACTGTAGCGCCAA
ATTGCCCAGTTAGAAAGCAGGTTCAAAGATGTGCTGAGGACATGTCAATATTGATCACCACATATGAAGGAA
CACACAACCACACACTCCCTCTTTCCGCCACCGCAATGGCTTCCACCACCTCTGCTGCCGCTAACATGTTGTTA

TCCGGTTCGTCGAGTTCATCAGACCCAAGTCCACAAATAACTGCCACCACTACCAACACCGCCACCGCTACTA
CTTCTGCCAATATCAACGGACTCAACTTCTATATCTCCGATACCTCAAAACATAAGTCGCCTTTTTACTTTCCT
AATTCATCCATCTCAGCATCCACACTTAATAATTCGCACCCTACAATCACTCTCGATTTGACTTCTACGTCCTC
CTCCTCATCCTCGTCCTTATCTCATCTTAACAGGATGAGTAACAATCTCCACCCTAGATATAATTACAATAACT
CCTCCACAAACCTCAATTTTAGTTCAGTGCTAGAATCCAATTCCCTTCCCATTTCTTGGACAAATTATCAAAAC
CAAACCTGCAACAAGAACAACCAAAATTTTGGTTCACTTAACTTCTCATCAAGACCAAATCAAGAAAATATTT
TCCAGTCTTATTTACAAAAAAACAACAACATTATCCCTACACAATCTTCTTTTCCACCAGATACAATTGCAGCT
GCAACGAAAGCAATAACGTCAGACCCTAATTTCCACTCAGCATTAGCTGCTGCTCTCACCTCAATCATTGGAA
ATACTGGAATCGAAAATAAGCCTGGCCATAATTTCAATGTTTCTGAGCCATTTCCAGTTTTGTCCAGTCTCCCA
TCAAGCTCAAATCCGAATAAATGCTCGTCAAGTTTTTTAAATAAACCAACTTCTTCTTCCGCGAATAATAGTTC
ACAGCAGCCTGGAAATAATAACAACTTGGTGTTCTTCGCGCAATCTTCTTCTTCTTCATTGCCATTTTCTACGT
CCAATAAGGGTAAATCTACCTCTCCTAGTGATAGCTAG

CaWRKY27

>Capana06g001110 [mRNA] locus=Chr06:20042156-20043655
ATGGAATTTACCAGTTTGGTTGATACTTCATTGGATTTGAGCTTTAGGCCTCGTCCAGTTCTTGATAAATTGCC
GAAACAAGAAGTTCAGAGTGATTTCACTGGATTGAGGGGAGACAATATGGGGGTGAAAAATGAGACAGTGG
ATTTGTTAGAGGAACTAAATAGAGTGAGCAGTGAAAACAAGAAGCTTACTGAGATGCTCACAGTGGTTTGTG
AAAATTACAATGTTTTAAGAAACCAAATGATGGAGTATATGAGCACACAAAATGGTGTGGCAGATGATAGTG
CAGGGTCAAGGAAGAGAAAAGCTGAAAGTATCTCCAATCCCAACAACAGCAACAGCAACGTCAACATCAAC
AACAACAACAACAACTTGGATGTTGTGCCTGGACGTTCATCAGAAAGTAGCTCAAGTGATGAAGAGTCTTCTT
GCAAGAAACTTAGAGAAGAGCACATAAAAGCCAAGGTTACAGTTGTTTCTATGAAGACTGATGCATCTGATA
CCTCTCTTATTGTAAAGGATGGTTATCAGTGGAGGAAGTATGGTCAGAAAGTAACAAGAGACAACCCTTGTC
CAAGAGCTTACTTTAGATGCTCATTTGCACCTACCTGTCCTGTCAAGAAGAAGGTTCAGAGAAGCATAGAAG
ATCAGTCTATTGTGGTGGCAACATATGAAGGAGAACATAACCATCCAATGACCTCAAAACCAGAAGCAGGAG

GTGCAAATACTACTAGTACTTCCACTGGCAGCCGGTTAAATGTGACGACTATCGCGGGTACTACTGCTTCAGT
ACCTTGCTCTACAACTCTCAATCCTTCAGGACCAACCATTACTCTCGATCTTACTGCACCGAAAACAGTAGAA
AAACGCGATATGAAGATGAATCAGAGTGCTAGTCCTACCGGTGGCAATAGCATTCATACATCAACAGGAGTT
GAATATCAAAATAGGCCAGAGTTCCAACAGTTCTTGATAGAGCAAATGGCTACTTCCTTGACCAAAGATCCA
AGCTTCAAAGCAGCACTTGCTGCCGCCATATCAGGAAAAATCCTCCAACATAATAATCAGACGGGCAGATGG
TAA
>CA00g87690
ATGGAATTTACCAGTTTGGTTGATACTTCATTGGATTTGAGCTTTAGGCCTCGTCCAGTTCTTGATAAATTGCC
GAAACAAGAAGTTCAGAGTGATTTCACTGGATTGAGGGGAGACAATATGGGGGTGAAAAATGAGACAGTGG
ATTTGTTAGAGGAACTAAATAGAGTGAGCAGTGAAAACAAGAAGCTTACTGAGATGCTCACAGTGGTTTGTG
AAAATTACAATGTTTTAAGAAACCAAATGATGGAGTATATGAGCACACAAAATGGTGTGGCAGATGATAGTG
CAGGGTCAAGGAAGAGAAAAGCTGAAAGTATCTCCAATCCCAACAACAGCAACAGCAACGTCAACATCAAC
AACAACAACAACAACTTGGATGTTGTGCCTGGACGTTCATCAGAAAGTAGCTCAAGTGATGAAGAGTCTTCTT
GCAAGAAACTTAGAGAAGAGCACATAAAAGCCAAGGTTACAGTTGTTTCTATGAAGACTGATGCATCTGATA
CCTCTCTTATTGTAAAGGATGGTTATCAGTGGAGGAAGTATGGTCAGAAAGTAACAAGAGACAACCCTTGTC
CAAGAGCTTACTTTAGATGCTCATTTGCACCTACCTGTCCTGTCAAGAAGAAGGTTCAGAGAAGCATAGAAG
ATCAGTCTATTGTGGTGGCAACATATGAAGGAGAACATAACCATCCAATGACCTCAAAACCAGAAGCAGGAG
GTGCAAATACTACTAGTACTTCCACTGGCAGCCGGTTAAATGTGACGACTATCGCGGGTACTACTGCTTCAGT
ACCTTGCTCTACAACTCTCAATCCTTCAGGACCAACCATTACTCTCGATCTTACTGCACCGAAAACAGTAGAA
AAACGCGATATGAAGATGAATCAGAGTGCTAGTCCTACCGGTGGCAATAGCATTCATACATCAACAGGAGTT
GAATATCAAAATAGGCCAGAGTTCCAACAGTTCTTGATAGAGCAAATGGCTACTTCCTTGACCAAAGATCCA
AGCTTCAAAGCAGCACTTGCTGCCGCCATATCAGGAAAAATCCTCCAACATAATAATCAGACGGGCAGATGG
TAA

CaWRKY28

>Capana06g001506 [mRNA] locus=Chr06:35621649-35624167
ATGGCTGCTTCAAGTTTCTCATTTCCCACTTCTTCTTCATTCATGACCACTTCTTTCACCGACCTTCTTGCTTCTT
CAGATGATTATCCTATTACCAAAGGACTTGGTGATAGAATTGCTGAGAGAACTGGTTCTGGAGTTCCTAAGTT
CAAGTCTCTACCACCTCCTTCACTTCCTTTATCTCCTCCTCCTTTTTCGCCTTCCTCTTACTTTGCTATTCCTCCT
GGTTTAAGTCCTACTGAACTCTTGGACTCACCTGTTCTCTTGTCATCTTCAAACGTTCTTCCGTCTCCCACAAC
AGGGAGTTTTCCAGCTCAGGCTTTTAATTGGAAGAGCAGCAGCAACAATCAGGATGTTAAACAGGAAGAAAA
AAACTGCTCTGATTTTTCCTTCCAGACACAAGTAGGGACAGCTGCATCAATATCTCAATCCCAAACTAGCCAT
GTCTCTTTGGGGCAGCAAGCATGGAATTATCAAGAGCCCACAAAGCAGGATGGTCTATCATCCGATCAAAAT
GCTAATGGAAGATCTGAATTCAACACTATGCAGAGTTTTATGCAGAATAATGATCATAGCAATAGCGGGAAC
GGATACAACCAGAGTATAAGGGAGCAGAAAAGATCAGATGATGGGTACAATTGGAGAAAATATGGGCAGAA
ACAAGTAAAAGGTAGTGAAAATCCAAGAAGTTACTACAAGTGTACATACCCAAATTGTCCCACCAAGAAGAA
GGTTGAAAGATCATTAGATGGCCAAATTACTGAGATTGTGTATAAGGGTAATCACAACCACCCAAAGCCTCA
GGCTACCAGAAGATCGTCGTCATCCACAGCTTCATCTGCAATCCAATCTTACAATACACAAACCAACGAAATC
CCAGATCATCAATCCTATGGTTCAAATGGTACAGGACAAATCGATTCAGTTGCAACACCTGAGAATTCTTCTA
TTTCATTTGGGGATGATGATCATGAGCACACTTCTCAAAAGAGCAGGTCAAGAGGAGATGATCTTGATGAAG
AGGAACCAGACTCGAAAAGATGGAAAAGAGAAAGCGAAAGTGAAGGTCTATCTGCACTTGGGAGTAGGACA
GTTAGAGAACCTAGAGTTGTAGTTCAAACTACAAGTGATATCGATATCCTAGATGATGGTTATAGATGGAGG
AAGTATGGTCAAAAAGTAGTGAAAGGAAATCCTAATCCCAGGAGCTACTACAAATGCACAAGTCCAGGATGT
CCAGTAAGAAAACATGTGGAAAGGGCATCACAAGATATAAAGTCAGTGATAACAACCTATGAAGGGAAGCA
CAACCATGACGTTCCGGCAGCTAGGGGCAGTGGAAACCACTCAATTAATCGACCTATTGCCCCGACCATTAC
GAACAACAATAGTGCCATGGCCATAAGACCCTCCGTGACATCTCATCAGTCCAACTATCAAGTTCCAATGCAA
AGTATAAGGCCACAACAGTTTGAAATGCGAGCACCCTTTACGCTAGAGATGTTGCAGAAGCCTAATAATTAT
GGTTTCTCAGGATATGCCAATTCAGAGGATTCATACGAAAACCAACTTCAGGACAATAATGGGTTTTCTAGAG
CTAAGAACGAACCTCGAGATGACATGTTTATGGAGTCATTGCTTTGCTGA
>CA06g13580

ATGGCTGCTTCAAGTTTCTCATTTCCCACTTCTTCTTCATTCATGACCACTTCTTTCACCGACCTTCTTGCTTCTT
CAGATGATTATCCTATTACCAAAGGACTTGGTGATAGAATTGCTGAGAGAACTGGTTCTGGAGTTCCTAAGTT
CAAGTCTCTACCACCTCCTTCACTTCCTTTATCTCCTCCTCCTTTTTCGCCTTCCTCTTACTTTGCTATTCCTCCT
GGTTTAAGTCCTACTGAACTCTTGGACTCACCTGTTCTCTTGTCATCTTCAAACGTTCTTCCGTCTCCCACAAC
AGGGAGTTTTCCAGCTCAGGCTTTTAATTGGAAGAGCAGCAGCAACAATCAGGATGTTAAACAGGAAGAAAA
AAACTGCTCTGATTTTTCCTTCCAGACACAAGTAGGGACAGCTGCATCAATATCTCAATCCCAAACTAGCCAT
GTCTCTTTGGTACTACTCCCTCCATCTCAAATTAGTTGTCGTTTTATCTTGCTTTACATGCCAGAGATATTAATT
AAGAGGGTTTCTTTGGTTACTTTAATCTTATGTCAAGCATGGAATTATCAAGAGCCCACAAAGCAGGATGGTC
TATCATCCGATCAAAATGCTAATGGAAGATCTGAATTCAACACTATGCAGAGTTTTATGCAGAATAATGATCA
TAGCAATAGCGGGAACGGATACAACCAGAGTATAAGGGAGCAGAAAAGATCAGATGATGGGTACAATTGGA
GAAAATATGGGCAGAAACAAGTAAAAGGTAGTGAAAATCCAAGAAGTTACTACAAGTGTACATACCCAAAT
TGTCCCACCAAGAAGAAGGTTGAAAGATCATTAGATGGCCAAATTACTGAGATTGTGTATAAGGGTAATCAC
AACCACCCAAAGCCTCAGGCTACCAGAAGATCGTCGTCATCCACAGCTTCATCTGCAATCCAATCTTACAATA
CACAAACCAACGAAATCCCAGATCATCAATCCTATGGTTCAAATGGTACAGGACAAATCGATTCAGTTGCAA
CACCTGAGAATTCTTCTATTTCATTTGGGGATGATGATCATGAGCACACTTCTCAAAAGAGCAGGTCAAGAGG
AGATGATCTTGATGAAGAGGAACCAGACTCGAAAAGATGGAAAAGAGAAAGCGAAAGTGAAGGTCTATCTG
CACTTGGGAGTAGGACAGTTAGAGAACCTAGAGTTGTAGTTCAAACTACAAGTGATATCGATATCCTAGATG
ATGGTTATAGATGGAGGAAGTATGGTCAAAAAGTAGTGAAAGGAAATCCTAATCCCAGGAGCTACTACAAAT
GCACAAGTCCAGGATGTCCAGTAAGAAAACATGTGGAAAGGGCATCACAAGATATAAAGTCAGTGATAACA
ACCTATGAAGGGAAGCACAACCATGACGTTCCGGCAGCTAGGGGCAGTGGAAACCACTCAATTAATCGACCT
ATTGCCCCGACCATTACGAACAACAATAGTGCCATGGCCATAAGACCCTCCGTGACATCTCATCAGTCCAACT
ATCAAGTTCCAATGCAAAGTATAAGGCCACAACAGTTTGAAATGCGAGCACCCTTTACGCTAGAGATGTTGC
AGAAGCCTAATAATTATGGTTTCTCAGGATATGCCAATTCAGAGGATTCATACGAAAACCAACTTCAGGACA
ATAATGGGTTTTCTAGAGCTAAGAACGAACCTCGAGATGACATGTTTATGGAGTCATTGCTTTGCTGA

CaWRKY29

> Capana06g002128 [mRNA] locus= Chr06:92548747..92550798

ATGGGCGATACAGACAGAAGAACAGTGAGAGTGGCTGCTCCAAGAATGGGAAATCTTGAGCTCCCACCTGAG
GACGGTTATACTTGGAGAAAATATGGTCAGAAAGAGATTCTTGGATCTAGGTTCCCTAGGTAATTCATCACAA
TTTCATTAACTAATACTCCCCCCTCACCCTTCCCCCTTCCCCTCTCCCAAATTGATTCTCACATTATGCTTCTCA
ATATTTGCTTTGATTAATAATTAAAGTTAAATTAAATTACATTAGGTTAACTCAATATTTTATAATTAAAATTT
AGATATAAAACTATTTGAAAAGTATAACTAGTTACAATTTTTTCTCATGTGAATTTGATGAAAAAAAATATAT
TTTAAAATATCAAAATTCATATTTGTAATTTGTTTTCAATGATTATTGATTAGTACTATCCACCAAAGTGGACA
AAAATCTAAAGACCAAGTGTGCTTCCTTATGACAGGGTCAAAATAAACCATATCTCCATCTTCGTAATTAATT
TAACTGATTACAAGACCTTTGAATTGAAGTCGAATTCAAGCTAGAAACAGTCAGTTTGAACAATTTGACTAAT
GGGATGCGAAGTGTCACCTTTATAAAGACATGAATGTATCCAATATTATAATTAAGAACTTTTCTTGTTTAGT
CACTCATAAAAAACGAGAAAACGCATAAAACATAAATGTATACGTATACTACTACACAATCAGCTTGCACTT
TTAGACCTTTCTCGCACTGACTTTTCCTATCTGTAGTAAACAAAATACTTATAATTGGCATATTTTGACCAATT
TGAATTCCACATACAACAACATTGTTGGAGTCAGTGGGACATTTTGACCCTTTAGATTTCTCACACATTCTAG
AAATATTGATATGCATCTAATAACTACCCTTTGATTTAATCTTCTTCTTGTTGTTTTCATATAAAAAATGGATC
CTCGTCTCTTTCCTTTGGTATGAACACGAGGAATTTTAAGATACAAAATTACTCCAGTATTTCATTTCATAAAG
ATTGTAAATTAATTATCAATTTAATTTTTGTGTACTAATAATGTAAACTTTCACACAATCATGTTACCTAGCTA
GAACAACGACACTGTATATATCTTTAATAATTAAGTCTAATTTGATAGCCAAAAAAATTAAGTACATGACATA
TTGCATACACCAACAATGTAATTTTTTATGGTAATTAAATCCATTAACTATTACTGATAGTTTTGATGATTTTA
GGGCTTATTATAGATGCACCCATCAAAAGCTATACCATTGTCCAGCCAAGAAGCAAGTTCAGCGTCTTGATAA
TGATCCTTACGTATTTGAAGTAACATATCGATCTCAACACATTTGCTATATGTCCGCCACAGCTCCCACCGTGC
CTCCACCATCTGTGGAAGAGATAACTCATCAAACCACCACAACTCCTCCACCGCTGCTGCCACTGCCGCCACC
AACTTCAGCCTCCTTAAGCGGGCATTGGCTCTCCATGGATATTAAGCCACAAGTAGAAGCTGGCACAAGCTAC
AGTACCGCTCAATTTGACATACAAAGGTACAAACATATTTCAGAATTAATTTAATGGGGTTCAACTTCAAGAT
ATTCATTTCTTGAAAATTTTAAAAAATAATCGGTCAAAAGTTTAATATTTGTACTACTTTATCCATGTTAAATA

TGGGTAAACCAGACATTTTATTTGTTTTTGTATAATCCATTTGTGATCCGTCAATAGCCGAATTGACCCTCCTA
TATGTCAATACTAAATTAACCTTACCGCTAATACTATAAATCCATGTTTGTACAGGGATTTTGGACATGCTAGT
GGTGGTTCACTAACTAGCATATGCAATGTTGTGACCGCCTGCGTGGACGGTGGTGCGGGCCCTTCCGGTAGTA
GATTTGGAAGAGAAGTTGACTACCAGCCAGTGGTGGATATGGCGGACGCCATGTTTAATTCTGGGAGTAGTA
GCAATACTAGCATGGACATCATCTTTTCTTCTATTGATGATAAATGGGACACGACACAAAAGAAAGAATAG

CaWRKY30

>Capana06g003072 [mRNA] locus=Chr06:218689176-218691106
ATGGAGGAAATAGAAGAAGCGAATAATGCTGCCGTTGAGAATTGTCATAGAGTGATGAGTCTTTTATCGTCT
GGGACACACGATCGGAATCAGTATATGAATTTAGTTAGAGAAACTGGAGAAGCAGTAAACAAGTTCAAGAA
AGTTGTCACTCTTTTGAATTCAACTTTAGGTCATGCAAGAGTGAGGAAATCGAACAAATTCAAGACCCCTTTA
CCTCATAACATCCTTGTAGAAAACCCGAATTGCAAAATCGATGATCAGGCGAAAGCTCTTAGGCGGAGCAGT
AGTGGCGTTAGTCTAAATTTTGATAGCTCGACATGTACTCCGACCATGTCCTCGACTAGGTCTTTTATCTCCTC
ATTGAGTGTGGACGGTAGCGTTGCTAATGGTAGTAACTTTCATTTAGTTGGGGCTTCACAATCTTTGGATCAG
AGCTCGTTCCAACACAAGAGAAGGTGCTCGGAAAGGGGAGACGAAGGAAGCGTGAAATGTGGAAGCAGTGG
GAAATGCCACTGTTCAAAGAAGAGGAAACACAGAGTGAAAAGATCAATCAAAGTACCTGCTGTAAGTAACA
AGCTAGCTGATATTCCTTCCGACGAGTATTCTTGGAGAAAGTATGGACAAAAGCCGATCAAAGGTTCTCCGCA
TCCTAGGGGATACTATAAATGTAGCAGCATGAGAGGCTGCCCTGCCCGGAAACATGTTGAGAGATGCTTGGA
AGACCCTTCAATGCTTATCGTGACTTACGAAGGTGAACATAATCATCCCAGAATGCCATCACAATCAGCTAAT
ACCTGA
>CA06g01330
ATGGAGGAAATAGAAGAAGCGAATAATGCTGCCGTTGAGAATTGTCATAGAGTGATGAGTCTTTTATCGTCT
GGGACACACGATCGGAATCAGTATATGAATTTAGTTAGAGAAACTGGAGAAGCTGTAAACAAGTTCAAGAAA
GTTGTCACTCTTTTGAATTCAACTTTAGGTCATGCAAGAGTGAGGAAATCGAACAAATTCAAGACCCCTTTAC

CTCATAACATCCTTGTAGAAAACCCGAATTGCAAAATCGATGATCAGGCGAAAGCTCTTAGGTCACGTCCTAT
CGATGAAAATCGAGTCCTAGAGATGGGGGGTACTAATGTGAAATGCAATCTTACTTTAGGAAGCCCTTCTTTG
GAATTAAGTTCAAATAGTAGAAATCCCCTTAATTTTGGCCAACAAACGCATTTGCCGAGCTACAACTATCTTC
AACAGCAGCAGCAGCAACAACGGCTGTTTCTACTTCAGCAGCAGTCTTTTATCTCCTCATTGAGTGTGGACGG
TAGCGTTGCTAATGGTAGTAACTTTCATTTAGTTGGGGCTTCACAATCTTTGGATCAGAGCTCGTTCCAACACA
AGAGAAGGTGCTCGGAAAGGGGAGACGAAGGAAGCGTGAAATGTGGAAGCAGTGGGAAATGCCACTGTTCA
AAGAAGAGGAAACACAGAGTGAAAAGATCAATCAAAGTACCTGCTGTAAGTAACAAGCTAGCTGATATTCCT
TCCGACGAGTATTCTTGGAGAAAGTATGGACAAAAGCCGATCAAAGGTTCTCCGCATCCTAGGGGATACTAT
AAATGTAGCAGCATGAGAGGCTGCCCTGCCCGGAAACATGTTGAGAGATGCTTGGAAGACCCTTCAATGCTT
ATCGTGACTTACGAAGGTGAACATAATCATCCCAGAATGGCATCACAATCAGCTAATACCTGA

CaWRKY31

>Capana07g000181 [mRNA] locus=Chr07:9032674-9041137
ATGGCCGGCGTTACTGAGAGCTCCAAATTCACCGGAACAAATAAAAGCAGTCACAAAATCGAAAACGGAGT
AGTCGATATCGATAACGGCGTTAACGGAGCTGAGAATTTACAATCGGAAAGTCAACTTTCTGTTTATTTAGCT
ATTTCGGGATGTGGGAGACGTTTGAGTGGATTTGCTGTGGATAGAATTAGTGTTACCAGGCCTGGAACTATGA
ATGCTCAAGCACGAACAAAACATCAACAACGTGTGCCAGATGAATCATCCACATTGGAGCTGTCCTCTACAT
CTGTTGCACAGTCCATTTCATCAGTGCCAAGTCCAACTCTAGCAGAAAGCCGGTTGTCAGCAGTAGTAAATTG
TGGTACAGGAGAAGTGGCTAAGCAGAGCTCCGATGCCAAGGTTCAACCTCTTGTACCAGTAAAGACATCAAA
CCGTGATGGGTACAACTGGCGGAAGTATGGTCAAAAGCAAGTTAAAAGTCCTCAGGGTACTCGAAGTTATTA
CCGATGCACTCATTTCGAGTGTTGTGCCAAGAAGATTGAGTGCTCTGGTCACACTAATCGTGTAATGGAGATT
ATTTATAGAAGTGAACACAATCATGATCCATCCCCGAGCGTAACTTGCTCAAGGGAAAGCAAGTCTGCAATA
TTGTCTGCATCTACCAATGGCAAAAGTTTAATAGATCATCCAAATAGAAATTCTAATGAGACTGTGGCATCCT
CTTTCAAAGAAAATTTACAAGAAAGTTTACCAATCGCCGAGACAGCAAATTTGGATTCCGGTGGATCCGACA

CTGACACTGAAATCGATATTAAAGAGGAGCATTGTGACGAACCTGAACAAAAGAAGAGGTCAAGAAAAAGT
GACGCGAGTTGTTACGAATCTGTTTCTAAACCTGGAAAGAAACCCAAACTTGTGGTGCATGCTGCTTGTGATG
TAGGAATCTCAAGTGATGGCTACAGGTGGCGAAAGTACGGACAAAAAATGGTGAAGGGAAATCCCCATCCA
AGGAACTATTATCGTTGTTCATCAGCTGGATGTCCTGTTCGAAAGCACATTGAGAGGGCTGTAGATAGCACAA
TCGCGCTAACAATAACCTACAAGGGAGTACACGATCATGACATGCCCGTACCAAAAAGGCGTCATGGTCCAC
CAAGTGCACCTCTTATTGCTGCTGCTGCCGCCCCAGCTTCCATAACCGATATGAAGAAACCTGAACCACTACA
ACATCAAAAATCGACCACACAATGGTCCGTTGATAAACAAGGTGAGTTGACAAGTGAGAAATTGGATCTTGG
AGGAGGTAAGGCAATGGGATCGGCTCGAACTCTGTTGAGTATTGGATTTGAAATAAAGCCTTGCTGA
>CA07g01910
ATGAATGCTCAAGCACGAACAAAACATCAACAACGTGTGCCAGATGAATCATCCACATTGGAGCTGTCCTCT
ACATCTGTTGCACAGTCCATTTCATCAGTGCCAAGTCCAACTCTAGCAGAAAGCCGGTTGTCAGCAGTAGTAA
ATTGTGGTACAGGAGAAGTGGCTAAGCAGAGCTCCGATGCCAAGGTTCAACCTCTTGTACCAGTAAAGACAT
CAAACCGTGATGGGTACAACTGGCGGAAGTATGGTCAAAAGCAAGTTAAAAGTCCTCAGGGTACTCGAAGTT
ATTACCGATGCACTCATTTCGAGTGTTGTGCCAAGAAGATTGAGTGCTCTGGTCACACTAATCGTGTAATGGA
GATTATTTATAGAAGTGAACACAATCATGATCCATCCCCGAGCGTAACTTGCTCAAGGGAAAGCAAGTCTGC
AATATTGTCTGCATCTACCAATGGCAAAAGTTTAATAGATCATCCAAATAGAAATTCTAATGAGACTGTGGCA
TCCTCTTTCAAAGAAAATTTACAAGAAAGTTTACCAATCGCCGAGACAGCAAATTTGGATTCCGGTGGATCCG
ACACTGACACTGAAATCAATATTAAAGAGGAGCATTGTGACGAACCTGAACAAAAGAAGAGGTCAAGAAAA
AGTGACGCGAGTTGTTACGAATCTGTTTCTAAACCTGGAAAGAAACCCAAACTTGTGGTGCATGCTGCTTGTG
ATGTAGGAATCTCAAGTGATGGCTACAGGTGGCGAAAGTACGGACAAAAAATGGTGAAGGGAAATCCCCAT
CCAAGGAACTATTATCGTTGTTCATCAGCTGGATGTCCTGTTCGAAAGCACATTGAGAGGGCTGTAGATAGCA
CAATCGCGCTAACAATAACCTACAAGGGAGTACACGATCATGACATGCCCGTACCAAAAAGGCGTCATGGTC
CACCAAGTGCACCTCTTATTGCTGCTGCTGCCGCCCCAGCTTCCATAACCGATATGAAGAAACCTGAACCACT
ACAACATCAAAAATCGACCACACAATGGTCCGTTGATAAACAAGGTGAGTTGACAAGTGAGAAATTGGATCT
TGGAGGAGGTAAGGCAATGGGATCGGCTCGAACTCTGTTGAGTATTGGATTTGAAATAAAGCCTTGCTGA

CaWRKY32

>Capana07g000528 [mRNA] locus=Chr07:40750931-40752123
ATGATTGAACCTTGGTCAAGAAGATGCTGGCACGAAAGACAAGAGTACATACAAGCTCGATCTGGAGAAGA
GGTTCCACCTGAAGATGGATACAGTTGGAGGAAATATGGACAAAAACTTATCGTAGGTGCAAAATATCCCAG
AGAACATTATCGATGTGATTGTCGTCGTTTATCTTATCGTGAGGCTACTAAGATGGTCCAACGAAGTGAGGCA
GAGCCATTATCTTTTGAAGTTACCTATGGAGGAAGTAACAGTTGTGGTCAAGAAAACAAAAACCAAAACGGA
GAACATGTTGTGCTAACAAAAGAAACACAGCGTGATGAGGTTGGAAGAGCAGCCGGGGAAACACCTGAATG
CTATACTCCGGAAATGGTTTCAACACCAAATACTTCATTCAACAATTCGTCCGCAGGGGTTGTGTTCTCAAAC
TCCAATCCGGTATTTAACATCACCAATTCCGATTTAATCCCCACACCGACTTCTTCACCTTATCCAGACACGGA
TATTTCACTTGAGGATGATAGTCTCACTGTCTTGTTTGATGATGTGCCTGAAAATGCTAGAAGCACCTATAAG
TGCAATGCAAACTAG
>CA05g11500
TGCTGGCACGAAAGACAAGAGTACATACAAGCTCGATCTGGAGAAGAGGTTCCACCTGAAGATGGATACAGT
TGGAGGAAATATGGACAAAAACTTATCGTAGGTGCAAAATATCCCAGAGAACATTATCGATGTGATTGTCGT
CGTTTATCTTATCGTGAGGCTACTAAGATGGTCCAACGAAGTGAGGCAGAGCCATTATCTTTTGAAGTTACCT
ATGGAGGAAGTAACAGTTGTGGTCAAGAAAACAAAAACCAAAACGGAGAACATGTTGTGCTAACAAAAGAA
ACACAGCGTGATGAGGTTGGAAGAGCAGCCGGGGAAACACCTGAATGCTATACTCCGGAAATGGTTTCAACA
CCAAATACTTCATTCAACAATTCGTCCGCAGGGGTTGTGTTCTCAAACTCCAATCCGGTATTTAACATCACCA
ATTCCGATTTAATCCCCACACCGACTTCTTCACCTTATCCAGACACGGATATTTCACTTGAGGATGATAGTCTC
ACTGTCTTGTTTGATGATGTGCCTGAAAATGCTAGAAGCACCTATAAGTGCAATGCAAACTAG

CaWRKY33

>Capana07g001256 [mRNA] locus=Chr07:167387734-167411421

ATGGCTGATTCCGGAAATTCGAAGAACTATGGCGTCCCTATTTACGGTGCCGGTTGGGTCCCACCGAGCGCCC
TCAGATCTGCAGTCGAACCGCCGCCGGCCACTGATGATGATAAGGACGACGGCGTCGAGAAATCATCGTCAT
CGGAAAGTTATGTTGTCCTTGCCGGAGGCGGTGGAGAAGGAAATAGCGGTATTCGTAACGCTCTCATTGTTGC
TCAATTCCATTTTGATTCTAATGTCCTCTCTGATGAACCCGTGGCTAGGTTAGGAACTGGCGGTGATCTTCCTT
ATAGGATGGCAGTACATCCAGGTGGAGACGGTCTTATATGTTCATTGCCAAAAAGCTGCAGATGGTTTGACTG
GGATATTCAGAGAGCTGAAAATCGTGCATTGGGTCTGAAATCATCTGAGAGAGTACTTGAGCCATTGGAGGA
TGTTGGACAACAATTAGCGCTAGCTTTTAACAATGACGGTTCTTTACTTGCTGTTGGCGGTGAGGAAGGCAAG
TTGAGGGTTTACAAGTGGCCTAGCATGGAAAATATTCTTGATCAGGCTAATGCTCATGCTTCTGTGAAGGATC
TAGACTTCAGCCCTGATGGAAAATTTCTTGCATCTGTTGGAAGCGGCCCTTGTCGGATTTGGGATGTCTCAAT
ATCAACATCTGTAGCTTCTTTGATGAAGGAAAATGATGAGATTTTTGGCTATTGTAGATTCTCACCAAGTAAC
GATGAGAATCAGGTTCTATACATCACCACAATGCAAGATCAAGGTGGAAGTATTTCAAAGTGGAATACTACT
ACATGGAAGAGGATAAAGTCAAAGCGTGTTGTTCGTGATCCTATTTGTGCCTTCAATCTTTCACCCAATGGAA
AGCTTCTGGCAATAGGAACAATTGAGGGAGATGTTCTGATAGTGTCTTCCAACAATTTACAAGTGCAAAATGT
GGTAAAAAAGGCTCATCTTGGTCTGGTAACAACATTGAAGTTCTCAGAAGATTCGAGGGCCTTGCTTTCTGCC
TCCATGGATTCAAGAGTGAGGGTGACAGTTATAAAGGAAGAGAAGAAAAGTGGCCGAATGGGCATATCTGA
GGATGATATGACGGATGAAATTTCCTCCAGAAAATTAAAGCAGAAACAGGACCCTGACACTGTCATCAACGG
ATCAGAGTCGAAAGAGAAAGGAACATGTGAATCAATGTCAGCTGAAGTTGTTTCTAATGAACTACATAAAAG
ACCGAACCCTGATGCTTTGGCAAAGGTATCACAAAGTAATCGAGATGAAAGTGCACATCCTACGACATGTCA
AGGAGTATTGAATGAAGGCCAGCCAAGGAGGAATGTCGATAAAGAGATCGATGTGTCACAATCCAATCAAA
AAGATAGTTCCCTTTCTAATGTACCAGAGGAAAATTCAGAAAACGTGCATCAGGAAAAGGGTCCAGAGAGTG
AGGGTGGTGCATCAGAATCTAGTCGAGTTTCTGTCCTACCAAAGGAGGAACCATACATAAAATCTTGTAAATC
AGATTCTCCTGTAAAAGAGGGGAACATCTCCCTGGTAGTTGGAACGGCTTCAGATTGTTCACCACAAATACAA
AGTAAAAAAATGGAAGAAGTTGTATCTCAATCTCATCAAGAACGAGTAACTTATTCAACAATGGCTGAGAAT
GCTATGTATAAATTGCGACCAAGGCGGAACCCTGATACGAGTGTCCAGGATTTGCCATCTGATGAAGGAGTC
AGGGATTTGCCATCTGATCAAGGAGTCACTCCCTTCAGTGAACCTGAAAAACCATTTGAAGATGGATATAACT

GGAGAAAATATGGTCAGAAACTTGTTAGAGGAAATATGTTTACTCGGAGTTATTACAAGTGCACACACTCCA
ATTGTCTAGCAAAAAAGCAAGTGGAGAGATCACATGATGGGCATATTACCAATATTCAGTATATAACAAACC
ATGAACATCCAAAACCTCTAAATAGTCCCCAAATCTCCCCTGAGGTTGTAGTGCCTTCGGAAATGAGACGACC
AGACATGCTAATGGGCACACCACAAGCTGAAGGTGAGAAATCTACTGCACTTGGTCAAGCATGTGAATCTAT
CGAACCATTGGAGAGCCTCATTTCAGCGGCTGTTGAATCGGCTGGTGGTAGCGCACGAGATACTGTCCCAAA
GTCACTTAAATCAGGAGATGAGGGTGATAGTAATGGTGGTCGGAACTCAAAGAGACGGAAGAAAGAAGTAC
CTAGGAGCGATGACATGACTCCACCTATGAAGTCTCATAGTGAACCACGACACATCGTTCAAACTAGGAGTG
AAGTGGATATACTCAATGATGGTTACCGATGGCGTAAATATGGGCAAAAAATTTGTGAAAGGAAATCCAAATC
CTAGGAGTTACTACCGATGCTCGAGTGCTGGTTGCCCTGCAAAGAAGCATGTGGAGAGGGCATCCCATGATC
CAAAATTAGTGATTACAACATATGAAGGGCAGCATGAGCATGACATCCCACTTTCCAGGACTGTTGCGCAAA
ATTCGGATTCTAATACAACCAGAATAAGTGGAGAGTCCACAGCTGAATCAGGTGGAAACAAACATGTTGACA
ATTGA
>CA07g10930
ATGGGCATATCTGAGGATGATATGACGGATGAAATTTCCTCCAGAAAATTAAAGCAGAAACAGGACCCTGAC
ACTGTCATCAACGGATCAGAGTCGAAAGAGAAAGGAACATGTGAATCAATGTCAGCTGAAGTTGTTTCTAAT
GAACTACATAAAAGACCGAACCCTGATGCTTTGGCAAAGGTATCACAAAGTAATCGAGATGAAAGTGCACAT
CCTACGACATGTCAAGGAGTATTGAATGAAGGCCAGCCAAGGAGGAATGTCGATAAAGAGATCGATGTGTCA
CAATCCAATCAAAAAGATAGTTCCCTTTCTAATGTACCAGAGGAAAATTCAGAAAACGTGCATCAGGAAAAG
GGTCCAGAGAGTGAGGGTGGTGCATCAGAATCTAGTCGAGTTTCTGTCCTACCAAAGGAGGAACCATACATA
AAATCTTGTAAATCAGATTCTCCTGTAAAAGAGGGGAACATCTCCCTGGTAGTTGGAACGGCTTCAGATTGTT
CACCACAAATACAAAGTAAAAAAATGGAAGAAGTTGTATCTCAATCTCATCAAGAACGAGTAACTTATTCAA
CAATGGCTGAGAATGCTATGTATAAATTGCGACCAAGGCGGAACCCTGATACGAGTGTCCAGGATTTGCCAT
CTGATGAAGGAGTCAGGGATTTGCCATCTGATCAAGGAGTCACTCCCTTCAGTGAACCTGAAAAACCATTTGA
AGATGGATATAACTGGAGAAAATATGGTCAGAAACTTGTTAGAGGAAATATGTTTACTCGGAGTTATTACAA
GTGCACACACTCCAATTGTCTAGCAAAAAAGCAAGTGGAGAGATCACATGATGGGCATATTACCAATATTCA

GTATATAACAAACCATGAACATCCAAAACCTCTAAATAGTCCCCAAATCTCCCCTGAGGTTGTAGTGCCTTCG
GAAATGAGACGACCAGACATGCTAATGGGCACACCACAAGCTGAAGGTGAGAAATCTACTGCACTTGGTCAA
GCATGTGAATCTATCGAACCATTGGAGAGCCTCATTTCAGCGGCTGTTGAATCGGCTGGTGGTAGCGCACGAG
ATACTGTCCCAAAGTCACTTAAATCAGGAGATGAGGGTGATAGTAATGGTGGTCGGAACTCAAAGAGACGGT
ATGTGAAAGTCAAGATCTTCTTTATTTGTCGTCACTGTTCAGTTCTGCAACTGCCTTGCGACAGGAAGAAAGA
AGTACCTAGGAGCGATGACATGACTCCACCTATGAAGTCTCATAGTGAACCACGACACATCGTTCAAACTAG
GAGTGAAGTGGATATACTCAATGATGGTTACCGATGGCGTAAATATGGGCAAAAATTTGTGAAAGGAAATCC
AAATCCTAGGAGTTACTACCGATGCTCGAGTGCTGGTTGCCCTGCAAAGAAGCATGTGGAGAGGGCATCCCA
TGATCCAAAATTAGTGATTACAACATATGAAGGGCAGCATGAGCATGACATCCCACTTTCCAGGACTGTTGCG
CAAAATTCGGATTCTAATACAACCAGAATAAGTGGAGAGTCCACAGCTGAATCAGGTGGAAACAAACATGTT
GACAATTGA

CaWRKY34

>Capana07g001387 [mRNA] locus=Chr07:177671908-177674692
ATGGAGTCATCAACATTCAAGAATAGGTCACCACCTTCTACTATTCAGTTCCCAGTGAACCTTAACTGCTCTA
CTACTGCTATTCATCATGATCACCAAGAAGAAGAAGAACATAATAACAGACCAGTTATTGATGAAATGG
ACTTCTTTGCTGATAAAAAAAATGGTAATAATTCTGAGGAGGCTGATGTTACAACAACAACCAATAATACTAT
CAATCACTCTGATAGAAAAGATTCCAACACCCCTCCTCCTGAATTGGATTTTAACATTAATACTGGTTTGCATC
TTCTCACGGCCAATACTTACAGTGATCAGTCCATAGTGGATGATGGTTTATCCCCTAATTCTGAAGATAAAAG
AACCAAGAGTGAGTTAGCAGTTCTTCGGGCTGAATTGGAAAGGATGAACGGTGAAAATCGACGTTTAAGGGA
CATGTTAAATCAGGTGACAAGCAATTACAGTACCCTGCAGATGCATATGATGACAATGATGCAACAACAACA
GCAACAAAATCAAGAAAATGGTCAACGTGATGGGAAAAGTACTCGCGAAGAAGTGAAACAGCAACACCATA
GCCATAATAGTCATGGAGGAGGAGGAGGACAAATGGTGCCTAGGCAATTTATGGATCTTGGCTTAGCTGCTG
CTGGTGCTACTGGTTCTGAGGCTGAAGAGGCTTCCCAGTCTTCGTCAGAGGGACGAAGTGGCAGGGAAAAT

CGCGATCACCAATGAATAACATGGAATCACGTTCCACATGTGGAATTGGAAGAGAAGATAGTCCTGAAAAAG
GGTCACCTGGTTGGGGTCCTAATAAAATTCCAAGACTTGGCAATGCCTCTACTAATAAACCTGCTGATCAAGC
TACCGAAGCTACCATGAGAAAGGCTCGAGTCTCGGTCAGGGCTCGATCAGAGGCTCCCATGATCACAGATGG
TTGCCAATGGCGAAAGTACGGACAGAAAATGGCGAAGGGAAATCCGTGTCCTCGGGCTTATTACCGGTGCAC
CATGGCAGCTGGTTGTCCAGTTCGGAAGCAAGTTCAAAGATGTGCAGAGGATAGAACAATCTTGATCACAAC
CTATGAAGGGACACACAACCATCCGTTGCCTCCGGCAGCGATGGCAATGGCCTCAACAACTTCCTCAGCAGC
ACGAATGTTGCTGTCGGGTTCTATGCCAAGTGCAGATGGGCTAATGAATTCCAATTTCTTCGCGAGAACTCTC
CTCCCTTGCTCTTCCAGCATGGCCACAATTTCAGCCTCGGCCCCGTTCCCTACTGTTACATTGGACCTAACTCA
ATCCCCAAACCCGTTGCAATTCCCAAGACCCCCTAACCAATTCCAAGTCCCATTTTCCAATCCACCTCACAGT
AACATCCTAGCAAATCCAGCTGCACTTTTGCCTCAGATATTTGGCCAGGCTTTGTATAACCAATCCAAATTCTC
TGGCCTCCAATTGTCCCAAGATTTGGAGGGACAACAACATCCTTCAACGATGTCATCATCGATTCATCCATCC
AACCACAACCCTCTGGCTGACACGGTGAACGCCCTCACCAATGATCCTAATTTCACCGCAGCATTAGCAGCAG
CCATCACTTCACTTATTGGAAATCCCGGGCAATCAAATAATGGTACAAATAATACCCCTGCCACCACAACGAC
CGCGAACAACAATAATGGCAGTGTTACAAGCAATGGCAATAACAGCAACAATGGCAATAATAAAGTGGCTA
ATTCAGGTTTTCCAGCAAATTGA
>CA07g11490
ATGGCCAAAGGTAGTGGACTCTCTTTTGATCCAGATCCCATCAAACACTTCCTTCCTATTCCTACTGTCCTCAA
TTCTTTTCTTGAACCCCACCACCAACAACAACAAGAATTTCCACATAACAAGTTTTTATTCAAGATTGAACCTT
TGTCATCCTCAATGGAGTCATCAACATTCAAGAATAGGTCACCACCTTCTACTATTCAGTTCCCAGTGAACCTT
AACTGCTCTACTACTGCTATTCATCATGATCACCAAGAAGAAGAAGAACATAATAACAGACCAGTTATT
GATGAAATGGACTTCTTTGCTGATAAAAAAAATGGTAATAATTCTGAGGAGGCTGATGTTACAACAACAACC
AATAATACTATCAATCACTCTGATAGAAAAGATTCCAACACCCCTCCTCCTGAATTGGATTTTAACATTAATA
CTGGTTTGCATCTTCTCACGGCCAATACTTACAGTGATCAGTCCATAGTGGATGATGGTTTATCCCCTAATTCT
GAAGATAAAAGAACCAAGAGTGAGTTAGCAGTTCTTCGGGCTGAATTGGAAAGGATGAACGGTGAAAATCG
ACGTTTAAGGGACATGTTAAATCAGGTGACAAGCAATTACAGTACCCTGCAGATGCATATGATGACAATGAT

GCAACAACAACAGCAACAAAATCAAGAAAATGGTCAACGTGATGGGAAAAGTACTCGCGAAGAAGTGAAAC
AGCAACACCATAGCCATAATAGTCATGGAGGAGGAGGAGGACAAATGGTGCCTAGGCAATTTATGGATCTTG
GCTTAGCTGCTGCTGGTGCTACTGGTTCTGAGGCTGAAGAGGCTTCCCAGTCTTCGTCAGAGGGACGAAGTGG
CAGGGAAAAATCGCGATCACCAATGAATAACATGGAATCACGTTCCACATGTGGAATTGGAAGAGAAGATA
GTCCTGAAAAAGGGTCACCTGGTTGGGGTCCTAATAAAATTCCAAGACTTGGCAATGCCTCTACTAATAAACC
TGCTGATCAAGCTACCGAAGCTACCATGAGAAAGGCTCGAGTCTCGGTCAGGGCTCGATCAGAGGCTCCCAT
GATCACAGATGGTTGCCAATGGCGAAAGTACGGACAGAAAATGGCGAAGGGAAATCCGTGTCCTCGGGCTTA
TTACCGGTGCACCATGGCAGCTGGTTGTCCAGTTCGGAAGCAAGTTCAAAGATGTGCAGAGGATAGAACAAT
CTTGATCACAACCTATGAAGGGACACACAACCATCCGTTGCCTCCGGCAGCGATGGCAATGGCCTCAACAAC
TTCCTCAGCAGCACGAATGTTGCTGTCGGGTTCTATGCCAAGTGCAGATGGGCTAATGAATTCCAATTTCTTC
GCGAGAACTCTCCTCCCTTGCTCTTCCAGCATGGCCACAATTTCAGCCTCGGCCCCATTCCCTACTGTTACATT
GGACCTAACTCAATCCCCAAACCCGTTGCAATTCCCAAGACCCCCTAACCAATTCCAAGTCCCATTTTCCAAT
CCACCTCACACTAACATCCTAGCAAATCCAGCTGCACTTTTGCCTCAGATATTTGGCCAGGCTTTGTATAACC
AGTCCAAATTCTCTGGCCTCCAATTGTCCCAAGATTTGGAGGGACAACAACATCCTTCAACGATGTCATCATC
GATTCATCCATCCAACCACAACCCTCTGGCTGACACGGTGAACGCCCTCACCAATGATCCTAATTTCACCGCA
GCATTAGCAGCAGCCATCACTTCACTTATTGGAAATCCCGGGCAATCAAATAATACCCCTGCCACCACAACGA
CCGCGAACAACAATAATGGCAGTGTTACAAGCAATGGCAATAACAGCAACAATGGCAATAATAAAGTGGCT
AATTCAGGTTTTCCAGCAAATTGA

CaWRKY35

>Capana07g001809 [mRNA] locus=Chr07:204092824-204096737
ATGGAAGATAGGCTATACAAAAGTTCATTTTTTCATAAGCAAGAAGATTCCACCGGAACTCCGCCGGATAAT
GCTGCTGATTCTTGTTTTTCCGGTGATGAAGCGGCTGAAGTTAACATGCCATCACCTAGAAAAAGGAGAGGA
GCAAAAAGGAAAGTAATATCAGTGCCAATAATTGAAGCTGATGGATCAAGGAGTAAAGGAGAAGTTTATCC

ACCACCAGATTCTTGGTCTTGGAGGAAATATGGACAGAAGCCAATTAAAGGTTCACCTTATCCCAGGGGATA
TTATCGATGCAGTAGTTCCAAAGGCTGTCCCGCCAGAAAACAAGTCGAACGTAGCTGCCTCGACCCCACCAT
GCTCCTCATCACCTACTGCTCCGATCACAACCACCAACTCCCCGCCGCCACCGCCACCAAACACCACCATACC
GCCGCCGGCGGCGCTACTTCACCATCCACCTCCACCGGCACCGCCGTAGACATCAACCCTTCCGCCGCCTCCG
ACACGGCCAAAAAATCGTCACCGGAAGAGCAAGAGACGAATATCTTTGCTGGATTCTCGGAGTTCGCCGGTG
AATTAGGTTGGTTTTCAGATATGGGAACCAGCACGTTAATGGAGAGTGCTTCGACTTCGGCAACATCCATAGT
GGGGTCCACATGGAATGATAGTGACGTGGCATTAATGTTGCCGATTCGGGAAGAGGACCAGTCATTGTACGG
TGATCTCGGTGAGTTGCCGGAATGTTCGTTTGTTTTCCGGCGGTATAGTGTCGAAACTACCTGTTGTGGCGGTA
CAGGATAA
>CA07g14560
ATGGAAGATAGGCTATACAAAAGTTCATTTTTTCATAAGCAAGAAGATTCCACCGGAACTCCGCCGGATAAT
GCTGCTGATTCTTGTTTTTCCGGTGATGAAGCGGCTGAAGTTAACATGCCATCACCTAGAAAAAGTAGGAGAG
GAGCAAAAAGGAAAGTAATATCAGTGCCAATAATTGAAGCTGATGGATCAAGGAGTAAAGGAGAAGTTTAT
CCACCACCAGATTCTTGGTCTTGGAGGAAATATGGACAGAAGCCAATTAAAGGTTCACCTTATCCCAGGGGA
TATTATCGATGCAGTAGTTCCAAAGGCTGTCCCGCCAGAAAACAAGTCGAACGTAGCTGCCTCGACCCCACC
ATGCTCCTCATCACCTACTGCTCCGATCACAACCACCAACTCCCCGCCGCCGCCGCCACCGCCACCAAACACC
ACCATACCGCCGCCGCCGGCGCTACTTCACCATCCACCTCCACCGGCACCGCCGTAGACATCAACCCTTCCGC
CGCCTCCGACACGGCCAAAAAATCGTCACCGGAAGAGCAAGAGACGAATATCTTTGCTGGATTCTCGGAGTT
CGCCGGTGAATTAGGTTGGTTTTCAGATATGGGAACCAGCACGTTAATGGAGAGTGCTTCGACTTCGGCAACA
TCCATAGTGGGGTCCACATGGAATGATAGTGACGTGGCATTAATGTTGGCGATTCGGGAAGAGGACCAGTCA
TTGTACGGTGATCTCGGTGAGTTGCCGGAATGTTCGTTTGTTTTCCGGCGGTATAGTGTCGAAACTACCTGTTG
TGGCGGTACAGGATAA

CaWRKY36

>Capana07g001968 [mRNA] locus=Chr07:210180549-210184089

ATGTCTGATAATAACCCATTTCATCATGATTATGCATTTCCTTTCTTTGGTGAAAATCCCTCAATTTATGATCA
TCAAGTAGAAAACACACAAAACCCTCATCAAGATTTTGATTATCCTTCATCTTATATGAGTAGTCTCACAGAG
TGTTTACATGGGGGTTCAATGGATCATTACAACTCTTTATCAAGTGCTTTTGGCATGAATCATCGTACATCATC
TTCTGAAGTTGTTTGTCCACCACCAATAGATCATCATCATCATCAAGAACTTTCTAGAAAAAATAGTGTTGAT
CATCATCATCAAATTCCATTGACACCTAACTCTTTAATCTCTTCATCATCTAATAGTGAGCCTGGATGTCATGA
AGAAGATTCTTCAAAAATCAAGAAAGATGATCAGTGTGAAGATGGAGGTGATGATGACAAGTCTAAGAAAG
TGAACAAAGCAAAGAAGAAAGGAGAAAAGAAGCAAAAGGAACCAAGATTTGCATTTATGACCAAGAGTGAG
ATTGATAATCTTGAAGATGGCTATAGATGGAGAAAATATGGACAAAAGGCAGTGAAGAATAGCCCTTTTCCA
AGGAGTTATTATAGGTGCACAAGTCAAAAGTGCAGTGTGAAGAAACGTGTGGAAAGATCATATCAAGACCCA
TCAATCGTGATCACTACATATGAAGGCCAACATAACCATCATTGTCCAGCAACCCTTAGAGGCAATGCTGCTG
CTGCTTTGTTATCACCAGCTTCATTCTTATCCTCCTCACAACAACAATTATTTCACAATCCAAGTGAACAACAA
CTTTTCTATAATCCAAATCTCCCTATTAATAATTCTTTCTACAATAATTATCATCAACATCAACACCAAATGCA
GCCACAATTAGGTCCTGATAATTATCAGTATGGAGTATTTCAAGATATGGTTGCATCATTGATCCACAAAAGA
GAGCCATG
>CA07g15490
ATGTCTGATAATAACCCATTTCATCATGATTATGCATTTCCTTTCTTTGGTGAAAATCCCTCAATTTATGATCA
TCAAGTAGAAAACACACAAAACCCTCATCAAGATTTTGATTATCCTTCATCTTATATGAGTAGTCTCACAGAG
TGTTTACATGGGGGTTCAATGGATCATTACAACTCTTTATCAAGTGCTTTTGGCATGAATCATCGTACATCATC
TTCTGAAGTTGTTTGTCCACCACCAATAGATCATCATCATCATCAAGAACTTTCTAGAAAAAATAGTGTTGAT
CATCATCATCAAATTCCATTGACACCTAACTCTTTAATCTCTTCATCATCTAATAGTGAGCCTGGATGTCATGA
AGAAGATTCTTCAAAAATCAAGAAAGATGATCAGTGTGAAGATGGAGGTGATGATGACAAGTCTAAGAAAG
TGAACAAAGCAAAGAAGAAAGGAGAAAAGAAGCAAAAGGAACCAAGATTTGCATTTATGACCAAGAGTGAG
ATTGATAATCTTGAAGATGGCTATAGATGGAGAAAATATGGACAAAAGGCAGTGAAGAATAGCCCTTTTCCA
AGGAGTTATTATAGGTGCACAAGTCAAAAGTGCAGTGTGAAGAAACGTGTGGAAAGATCATATCAAGACCCA
TCAATCGTGATCACTACATATGAAGGCCAACATAACCATCATTGTCCAGCAACCCTTAGAGGCAATGCTGCTG

CTGCTTTGTTATCACCAGCTTCATTCTTATCCTCCTCACAACAACAATTATTTCACAATCCAAGTGAACAACAA
CTTTTCTATAATCCAAATCTCCCTATTAATAATTCTTTCTACAATAATTATCATCAACATCAACACCAAATGCA
GCCACAATTAGGTCCTGATAATTATCAGTATGGAGTATTTCAAGATATGGTTGCATCATTGATCCACAAAAGA
GAGCCATGA

CaWRKY37

>Capana07g002350 [mRNA] locus=Chr07:219055519-219061043
ATGGCAAACTCCCACGCCGAGCAGCTCACTAATGGCAGAACCTCCGCTTCCTCCGCCGCAGCTCCCGGCGGAT
CGAACGGAGGCGGAGCCGTAGCAAAGTACAAGCTGATGACTCCAGCTATGTTACCGATCTCTAGGTCCACAT
GTATCACTATACCTCCCGGTCTCAGTCCTTCTTCCTTCCTCGAATCTCCTCTTCTCCTTTCTAACATCAAAGCTG
AGCCATCTCCGACTACAGGTTCCTTCTCCAAATTTCAAACAGTGCAAGGCTCTGGTGGGGCTGCTGCATTCTT
ATTGACGAGAGGTTATTCTAGTAGCAATTCATACATTGAAAGAAAATCCAGCTGCTTTGAGTTCAAAAATGCC
AGTGGATCTAGTTCTACATCAGGATCGTTTGCAACTGAACATGTGATTTCTACAGGTTTCAACCAACAACAAA
ATGATCCACTGAAAGAAGTTCAAGATCAAAGTCATCGTCAATTGTTGGTACCTTCATCTCTAGCTAAACTCGA
GATGGAATCATCAAAAGAACTGAGTATATCTGCGCCTGTTAATGTTGATGCTTCATCAAAAGAAGAGAGTCT
ATGTCAACCTATTAATGTTGATGCCATGAATCCGGGAGGTCCATCTAATGCAAGCATGCAAGGTTCACATGCC
GATCATAAAGATGTATCATCAGTAACATCTGAGAGATCATCAGATGATGGGTATAACTGGCGAAAGTATGGC
CAGAAACTTGTTAAAGGAAGTGAATTTCCAAGAAGCTATTACAAATGTACATACCCAAACTGTGAAGTGAAA
AAGATATTTGAGCGGTCACCTGAAGGACAAATAACAGAGATTGTTTATAAAGGTTCCCATGATCATCCTAAA
CCCCAACTGTGTCGCCGATTTTCTCCTGGTTCTCTCGTGTCTATCCAAGAAGATAAATGCGAGAAAGAAGCGT
GTTTCAGGGGTCAAGAAGTCTATGTTGAAGACAAGTTCAACACCAATGTCCAAACTAATAAAATTGAGCCTG
GTAGTACCCCTGTATCACCTCAAACGGACACTGATGCTCTTGAAGGAGCAGCGTCACAGATGCAAGGCACTA
ATGATGACATGGATGAGGATGATCAATTTGCGAAAAGAAGGAAAATGGATGGTGGGATGGATGTAACACCA
GTGATAAAGCCTATCCGTGAACCACGCGTGGTTGTTCAAACAGTCAGTGAAGTTGATATATTGGATGATGGGT
ACAAGTGGCGCAAATATGGACAGAAGGTGGTCCGAGGCAATCCTAATCCCAGGAGCTATTACAAGTGCACCA

ATGCTGGATGCCCTGTCAGGAAACACGTGGAGAGGGCTTCTCACGATCCCAAAGCTGTTATCACCACATATG
AAGGAAAACATAATCACGATGTACCTACTGCGAGGAATAATAACCATGAAATGACAGGATCCACGCCTGTAA
CTGGCGGTTCAAGGATCAGGGCAGAACAGACCAACTCACTTAGTCTGGATCTAGGTGTCGGTACTGGATATC
ATCTGGACAATGGGAACAACGGGCAACTTCACACCCTCCATAACCAAGTTCAAGTTTCACGTTCTGGTATGAT
GCTAGTACAACCAGGTGCAGTTGTAGCGCGATATGGTATTGTACATAATGGCATGAGCCGATTTGGAGCTATA
GATAATCGTGTTCAAGGACCTAGTTTCGAAACTTTACCTTTACAACCTTCTACCCAGTCTCTCCAAAGCTACGG
AAAGATACTCCTCGGCCCGTGA
>CA07g20160
ATGGCAAACTCCCACGCCGAGCAGCTCACTAATGGCAGAACCTCCGCTTCCTCCNNNNNNNNGAACGGAGGC
GGAGCCGTAGCAAAGTACAAGCTGATGACTCCAGCTATGTTACCGATCTCTAGGTCCACATGTATCACTATAC
CTCCCGGTCTCAGTCCTTCTTCCTTCCTCGAATCTCCTCTTCTCCTTTCTAACATCAAAGCTGAGCCATCTCCGA
CTACAGGTTCCTTCTCCAAATTTCAAACAGTGCAAGGCTCTGGTGGGGCTGCTGCATTCTTATTGACGAGAGG
TTATTCTAGTAGCAATTCATACATTGAAAGAAAATCCAGCTGCTTTGAGTTCAAAAATGCCAGTGGATCTAGT
TCTACATCAGGATCGTTTGCAACTGAACATGTGATTTCTACAGGTTTCAACCAACAACAAAATGATCCACTGA
AAGAAGTTCAAGATCAAAGTCATCGTCAATTGTTGGTACCTTCATCTCTAGCTAAACTCGAGATGGAATCATC
AAAAGAACTGAGTATATCTGCGCCTGTTAATGTTGATGCTTCATCAAAAGAAGAGAGTCTATGTCAACCTATT
AATGTTGATGCCATGAATCCGGGAGGTCCATCTAATGCAAGCATGCAAGGTTCACATGCCGATCATAAAGAT
GTATCATCAGTAACATCTGAGAGATCATCAGATGATGGGTATAACTGGCGAAAGTATGGCCAGAAACTTGTT
AAAGGAAGTGAATTTCCAAGAAGCTATTACAAATGTACATACCCAAACTGTGAAGTGAAAAAGATATTTGAG
CGGTCACCTGAAGGACAAATAACAGAGATTGTTTATAAAGGTTCCCATGATCATCCTAAACCCCAACTGTGTC
GCCGATTTTCTCCTGGTTCTCTCGTGTCTATCCAAGAAGATAAATGCGAGAAAGAAGCGTGTTTCAGGGGTCA
AGAAGTCAACCTTTATCGATTCTCAGTCTATGTTGAAGACAAGTTCAACACCAATGTCCAAACTAATAAAATT
GAGCCTGGTAGTACCCCTGTATCACCTCAAACGGACACTGATGCTCTTGAAGGAGCAGCGTCACAGATGCAA
GGCACTAATGATGACATGGATGAGGATGATCAATTTGCGAAAAGAAGCAGGAAAATGGATGGTGGGATGGA
TGTAACACCAGTGATAAAGCCTATCCGTGAACCACGCGTGGTTGTTCAAACAGTCAGTGAAGTTGATATATTG

GATGATGGGTACAAGTGGCGCAAATATGGACAGAAGGTGGTCCGAGGCAATCCTAATCCCAGGAGCTATTAC
AAGTGCACCAATGCTGGATGCCCTGTCAGGAAACACGTGGAGAGGGCTTCTCACGATCCCAAAGCTGTTATC
ACCACATATGAAGGAAAACATAATCACGATGTACCTACTGCGAGGAATAATAACCATGAAATGACAGGATCC
ACGCCTGTAACTGGCGGTTCAAGGATCAGGGCAGAACAGACCAACTCACTTAGTCTGGATCTAGGTGTCGGT
ACTGGATATCATCTGGACAATGGGAACAACGGGCAACTTCACACCCTCCATAACCAAGTTCAAGTTTCACGTT
CTGGTATGATGCTAGTACAACCAGGTGCAGTTGTAGCGCGATATGGTATTGTACATAATGGCATGAGCCGATT
TGGAGCTATAGATAATCGTGTTCAAGGACCTAGTTTCGAAACTTTACCTTTACAACCTTCTACCCAGTCTCTCC
AAAGCTACGGAAAGATACTCCTCGGCCCGTGA

CaWRKY38

>Capana07g002454 [mRNA] locus=Chr07:220545442-220548961
ATGGGTGGATTTGATGATCATGTTGCCATTATGGGAGATTGGATGCCTCCAAGTCCAAGTCCAAGAACTTTTT
TCTCGTCGCTGCTAGGTGATGATGTTGGGTCAAGATCAACTTTTCAGTGTACCAATGAAACTAAAAGTGGAAA
CTTAGCTTCTGGGCCTCAAGAAAACGTGGGAACTTTTGATGGAAATGATGAAGCACAAGCTGCAGTCAGTGA
ACAACAACCGGTGTCTGATCAGAAAATGAACCCTCGTGGAGGACTCTTGGAAAGAATGGCAGCTAGAGCTGG
ATTTAATGCTCCAAAGCTGAACACGGAGAGCCTTAGACCTGCTGATATGAGGCAGAATCAAGGAGTTCAGAA
TCAAGGAGTTCGGTCTCCTTATTTAACTATTCCTCCTGGTCTTAGTCCAACAACCTTGCTAGAGTCTCCTGTTTT
CCTCTCAAATTCACTGGTGCAACCATCCCCAACCACTGGAAAATTTCCATTTTCCTCGGGCATCGAGAGTAGA
AACTCAACATTGATGATGGAGGATCCAGATAATAGGAAAGAGAATGCTTTTGAGAGTATCAATGCGTCGTCC
TTTTCTTTCAAGCCAGTTCCAGAGACTGCTCCATCGCTTTTTCCTGGCACGAACAGCAGAGTGAACCCGCCCA
ATTTTTCTCAGCAAGGATTTCCAAACATTGAAGTTTCAGTTCATTCACAGAACTCGCTTCAATCTCACCGTATG
GAGGCTACACAAAATCTGGTTCAGAATGGAACGCTTAATCAAGCATCTGATTTCCCTAGATTTTCTGCTGAGA
TGGATGTCAAGGGTAGTAATGTCACACCAGAGTCAAGGACCTTTCAGACGGTTGGTAGTACTGTGGAGCATT
CTCCACCTCTCGATGAGCCGCAAGATGAGGACATTGATCAAAGAGGAGGTGGAGATCCAAATGTTGTTGGTG
CCCCAGCAGAAGATGGGTATAACTGGCGAAAATACGGGCAGAACAAGTTAAAGGGAGCGAGTATCCCCGG

AGCTACTATAAGTGCACACATCCAAACTGTCCAGTGAAGAAGAAAGTTGAGCGATCTCAAGAGGGTCATATT
ACTGAGATTATATACAAGGGAGCCCACAATCACCCAAAACCGCCACCTAACCGTAGATCAGCCCTTGGATCC
ACAAATTCACTTGGTGACCTACGGCTGGACGGTGTAGAACAAGGTGCAAGTGGTGTTAATGGTGATCTGGGT
CGGGCAAACATCCAGAAAGCACCTGGTTCTGGAGGAGGTTTTGATTGGAGGAGCAACAACCTTGACGCAACA
TCATCAGTGAACTTGGGCTCCGAGTACTGCAACAGATCTGCCCCTTTCCCTGCTCAAAACGACAGTCAGTTGG
AATCAGGGGATGCAGTAGATGTGTCGTCGACTTTTTCAAATGATGAAGATGAAGATGATCGTGGAACTCATG
GCAGCATATCACAAGGTTATGATGGTGAAGGAGATGAGTCCGAGTCTAAAAGAAGGAAGCTCGAGACTTACT
CTGCAGATATGACTGGTGCCACTAGAGCCATCAGAGAGCCAAGAGTTGTGGTTCAAACTACAAGCGAAGTGG
ACATCCTTGATGATGGATATCGCTGGCGCAAGTATGGGCAAAAGGTTGTTAAAGGGAATCCAAATCCAAGGA
GTTACTACAAGTGCACAAGTGCTGGCTGCAATGTCAGGAAGCATGTTGAGAGGGCCTCACATGACCTGAAGT
CAGTGATTACCACCTATGAAGGGAAGCACAACCACGATGTTCCTGCAGCTCGCAACAGTAGTCACGTTAATTC
AGGAGCCTCCAACACTCATCCAACTGCAGTAACTGCCCCTGCTCAAAACCATTTACATAGGCCTGAGCCTGCA
CAACTTCAGAATGCCATGGCGCGGTTCGACAGGCAACCTTCACTTGGCTCCTTTGGTTTGGCAGGGAGGCCCC
AGCTTGGACCTACCCCAGGATTTAGCTACGGAATGAACCCACAAGGCCTATCCAGTCTATCAATGGCTGGATT
TCACCCTAACCAAAACAAGCCAGGGGAGGTTCCTATTCATCCATATCTAGGACAGCCACGACCCATGCATGA
CATGGGATTTATGTTCCCGAAGGAAGAACCAAAGGTGGAACCTATGTCTGATCCTGGATTGAACCTCTCCAAT
GGCTCAAACGTTTATCAGCAATTCATGAACAGGTTGCCGCTCGGACCTCAGATGTAA
>CA07g21030
ATGGGTGGATTTGATGATCATGTTGCCATTATGGGAGATTGGATGCCTCCAAGTCCAAGTCCAAGAACTTTTT
TCTCGTCGCTGCTAGGTGATGATGTTGGGTCAAGATCAACTTTTCAGTGTACCAATGAAACTAAAAGTGGAAA
CTTAGCTTCTGGGCCTCAAGAAAACGTGGGAACTTTTGATGGAAATGATGAAGCACAAGCTGCAGTCAGTGA
ACAACAACCGGTGTCTGATCAGAAAATGAACCCTCGTGGAGGACTCTTGGAAAGAATGGCAGCTAGAGCTGG
ATTTAATGCTCCAAAGCTGAACACGGAGAGCCTTAGACCTGCTGATATGAGGCAGAATCAAGGAGTTCAGAA
TCAAGGAGTTCGGTCTCCTTATTTAACTATTCCTCCTGGTCTTAGTCCAACAACCTTGCTAGAGTCTCCTGTTTT
CCTCTCAAATTCACTGGTGCAACCATCCCCAACCACTGGAAAATTTCCATTTTCCTCGGGCATCGAGAGTAGA

AACTCAACATTGATGATGGAGGATCCAGATAATAGGAAAGAGAATGCTTTTGAGAGTATCAATGCGTCGTCC
TTTTCTTTCAAGCCAGTTCCAGAGACTGCTCCATCGCTTTTTCCTGGCACGAACAGCAGATCTTGGTTGCAGGT
GAACCCGCCCAATTTTTCTCAGCAAGGATTTCCAAACATTGAAGTTTCAGTTCATTCACAGAACTCGCTTCAA
TCTCACCGTATGGAGGCTACACAAAATCTGGTTCAGAATGGAACGCTTAATCAAGCATCTGATTTCCCTAGAT
TTTCTGCTGAGATGGATGTCAAGGGTAGTAATGTCACACCAGAGTCAAGGACCTTTCAGACGGTTGGTAGTAC
TGTGGAGCATTCTCCACCTCTCGATGAGCCGCAAGATGAGGACATTGATCAAAGAGGAGGTGGAGATCCAAA
TGTTGTTGGTGCCCCAGCAGAAGATGGGTATAACTGGCGAAAATACGGGCAGAAACAAGTTAAAGGGAGCG
AGTATCCCCGGAGCTACTATAAGTGCACACATCCAAACTGTCCAGTGAAGAAGAAAGTTGAGCGATCTCAAG
AGGGTCATATTACTGAGATTATATACAAGGGAGCCCACAATCACCCAAAACCGCCACCTAACCGTAGATCAG
CCCTTGGATCCACAAATTCACTTGGTGACCTACGGCTGGACGGTGTAGAACAAGGTGCAAGTGGTGTTAATG
GTGATCTGGGTCGGGCAAACATCCAGAAAGCACCTGGTTCTGGAGGAGGTTTTGATTGGAGGAGCAACAACC
TTGACGCAACATCATCAGTGAACTTGGGCTCCGAGTACTGCAACAGATCTGCCCCTTTCCCTGCTCAAAACGA
CAGTCAGTTGGAATCAGGGGATGCAGTAGATGTGTCGTCGACTTTTTCAAATGATGAAGATGAAGATGATCG
TGGAACTCATGGCAGCATATCACAAGGTTATGATGGTGAAGGAGATGAGTCCGAGTCTAAAAGAAGGAAGCT
CGAGACTTACTCTGCAGATATGACTGGTGCCACTAGAGCCATCAGAGAGCCAAGAGTTGTGGTTCAAACTAC
AAGCGAAGTGGACATCCTTGATGATGGATATCGCTGGCGCAAGTATGGGCAAAAGGTTGTTAAAGGGAATCC
AAATCCAAGGAGTTACTACAAGTGCACAAGTGCTGGCTGCAATGTCAGGAAGCATGTTGAGAGGGCCTCACA
TGACCTGAAGTCAGTGATTACCACCTATGAAGGGAAGCACAACCACGATGTTCCTGCAGCTCGCAACAGTAG
TCACGTTAATTCAGGAGCCTCCAACACTCATCCAACTGCAGTAACTGCCCCTGCTCAAAACCATTTACATAGG
CCTGAGCCTGCACAACTTCAGAATGCCATGGCGCGGTTCGACAGGCAACCTTCACTTGGCTCCTTTGGTTTGG
CAGGGAGGCCCCAGCTTGGACCTACCCCAGGATTTAGCTACGGAATGAACCCACAAGGCCTATCCAGTCTAT
CAATGGCTGGATTTCACCCTAACCAAAACAAGCCAGGGGAGGTTCCTATTCATCCATATCTAGGACAGCCGC
GACCCATGCATGACATGGGATTTATGTTCCCGAAGGAAGAACCAAAGGTGGAACCTATGTCTGATCCTGGAT
TGAACCTCTCCAATGGCTCAAACGTTTATCAGCAATTCATGAACAGGTTGCCGCTCGGACCTCAGATGTAA

CaWRKY39

>Capana08g000429 [mRNA] locus=Chr08:60198759-60200111
ATGGCTGCAAATAATCCCAGTGCAAACATGCTTGATGGGAGTTTTAGATCATTGGACTCACCTGACAGTGATG
ATTTCTCAAACCACCTAATTAACTTTGAGCTTTCTGATATTCTCGAAATAGATAATTGGCCCATTCAACAAGAT
CCGACACTCATACCCCAGTACTCAAATTATGCAGCAAACCAAGTGGTTAACACCAGCAGTTACCAGGAAGAA
CCTAGCAACAACATTGGAAGCAGCAGCAGCAAGAGGAAAGAAGTAAAGGACAAGGTTGCTTTCAGAACGCT
ATCACAGATTGAAATACTAGACGATGGCTATAAGTGGAGAAAGTATGGAAAGAAGATGGTGAAAAATAGTC
CCAACCCGAGGAATTACTATAGGTGCTCCGTAGAAGGTTGTCCAGTGAAGAAGAGAGTTGAACGAGACAAAG
AGGACTCTCGGTATGTGATAACCACCTACGAGGGTGTCCACAACCATCAAGGTCTATCCCCATTCTGA
>CA08g03020
ATGGCTGCAAATAATCCCAGTGCAAACATGCTTGATGGGAGTTTTAGATCATTGGACTCACCTGACAGTGATG
ATTTCTCAAACCACCTAATTAACTTTGAGCTTTCTGATATTCTCGAAATAGATAATTGGCCCATTCAACAAGAT
CCGACACTCATACCCCAGTACTCAAATTATGCAGCAAACCAAGTGGTTAACACCAGCAGTTACCAGGAAGAA
CCTAGCAACAACATTGGAAGCAGCAGCAGCAAGAGGAAAGAAGTAAAGGACAAGGTTGCTTTCAGAATGCT
ATCACAGATTGAAATACTAGACGATGGCTATAAGTGGAGAAAGTATGGAAAGAAGATGGTGAAAAATAGTC
CCAACCCGAGGAATTACTATAGGTGCTCCGTAGAAGGTTGTCCAGTGAAGAAGAGAGTTGAACGAGACAAAG
AGGACTCTCGGTATGTGATAACCACCTACGAGGGTGTCCACAACCATCAAGGTCTATCCCCATTCTGA

CaWRKY40

>Capana08g000683 [mRNA] locus=Chr08:101219429-101222082
ATGGATACCAATTTGGGAGACAAAACATTTTCTATTGACCTCAACACAAACCCCTCATTGCACAACACCAGTA
GAAGTCCGCATGACACGTTGGATGAAAAGTTGGTTAGGATGAGAGAAGAGAACAAAAAACTTGTAACAATG
CTAACTACTTTGTGCGAAAACTACAATTCATTGCACTCTCACCTAATTGAGTTGCTGCAGAAATACTTCAGTC

ATAATGAAGAGGACAATTTCAAATTTTTTTTAAGGAAAAGAAAGGCTGAAGGAGAATGTTGTGAGAATAATT
CAGACATCCATTTTGAAGAAGCATCACCAAAGAGGCCAAGAGAAATCACAACCAATGTTTCAACTGTTTGCG
TTAAAACCAATCCCTCCGATCAAACCTCGTTGGTGAAGGATGGATATAACTGGAGAAAATATGGTCAAAAAG
TGACAAGAGATAATCCTTATCCAAGAGCCTACTACAAGTGTTCATTTGCACCAACATGTCCAGTCAAGAAGA
AGGTACAAAGAAGTATTGAAGATCCATCAATTTTAGTAGCTGTATATGAAGGGGAGCACAACCACCCTCACC
CATCCCAAGCTGAAATAACAGTGCCATTACTCAACCAAGGTGTTACAACAGATCCAACATTTTTGAACAAATT
AATGGAAGAGATTGACACAAATTCACTGCAGCAACATTTAGTCGAACAAATGGCGTCTTCCTTGACTAGCAG
CCCTAGTTTCACTGCTGCAGTTGCTGCGGCCATCTCTGGAAAGATTTTCGAATATGATTTACCTTTCAAATAA
>CA00g00130
ATGGATACCAATTTGGGAGACAAAACATTTTCTATTGACCTCAACACAAACCCCTCATTGCACAACACCAGTA
GAAGTCCGCATGACACGTTGGATGAAAAGTTGGTTAGGATGAGAGAAGAGAACAAAAAACTTGTAACAATG
CTAACTACTTTGTGCGAAAACTACAATTCATTGCACTCTCACCTAATTGAGTTGCTGCAGAAATACTTCAGTC
ATAATGAAGAGGACAATTTCAAATTTTTTTTAAGGAAAAGAAAGGCTGAAGGAGAATGTTGTGAGAATAATT
CAGACATCCATTTTGAAGAAGCATCACCAAAGAGGCCAAGAGAAATCACAACCAATGTTTCAACTGTTTGCG
TTAAAACCAATCCCTCCGATCAAACCTCGTTGGTGAAGGATGGATATAACTGGAGAAAATATGGTCAAAAAG
TGACAAGAGATAATCCTTATCCAAGAGCCTACTACAAGTGTTCATTTGCACCAACATGTCCAGTCAAGAAGA
AGGTACAAAGAAGTATTGAAGATCCATCAATTTTAGTAGCTGTATATGAAGGGGAGCACAACCACCCTCACC
CATCCCAAGCTGAAATAACAGTGCCATTACTCAACCAAGGTGTTACAACAGATCCAACATTTTTGAACAAATT
AATGGAAGAGATTGACACAAATTCACTGCAGCAACATTTAGTCGAACAAATGGCGTCTTCCTTGACTAGCAG
CCCTAGTTTCACTGCTGCAGTTGCTGCGGCCATCTCTGGAAAGATTTTCGAATATGATTTACCTTTCAAATAA

CaWRKY41

>Capana08g001012 [mRNA] locus=Chr08:123573760-123575138
ATGGAGGAAGATTGGGATCTACATGCAGTGGTCAGAGGCTGCACAGCTAGTTCCACCACCACAACCACCAGC

ACCACCACCACTGCAACTTCATGTTGTAGCTTCCAACCAAGACAAGATGGCAACTTTTTCAGCTTTCAAGATC
CATTTGTGCCAAGATTTGACAACCCCACAAGTGATTTTGAAGAGTTGCATAATCTTTACAAGCCTTTCTTTCCT
AAATCACAAGTACAACCACAACAACAAGTACCTCGTTCTCCTCAAAATAATATAATACCCATTTCACCCCTCT
CTGTTCTTGGTGGACTACAAGATCTATCAGCACCTCAACCAACACTAAAACAACAGCAGCAGCAGCAGCAAC
AACAGCAACAACATATTCATCAGTTTTTTAACAGTACAAGACTAACACAACCCAAACAATCTCTGTCTGTAAA
TGGTTCAACAAATAGTACAATTACTGCTTCTTCTTCACTTGGTGTCTCACATACTCAAAGCCCAAGACCTAAA
AGAAGGAAAAATCAATTAAAGAAGGTATGCCAAGTACCTGCTGAAGGTTTATCTTCTGACATGTGGTCTTGG
AGAAAATATGGACAAAAACCCATCAAAGGCTCTCCATATCCAAGGGGATATTACAGGTGTAGCACGTCAAAG
GGTTGTTTAGCCCGAAAACAAGTGGAGCGAAATAGATCCGACCCGAATATGTTCATTGTCACCTATACAGCTG
AGCACAACCACCCCATGCCAACTCACCGGAATTCCCTCGCCGGAAGTACCCGTCAGAAGCCGGCGAATTCCG
AAGCTGGCACCACTGCAAGCGACTCTAACAAACCTACCAGCTCATCGCCGGTATCTTCGCCGGCGTGCCATTC
TACGGCGACGGAGAAGCAAGAAAGCAGCAGGGAAGAAAAAGAAGACATTTTCGAAGACGAGGACGAAGAA
TTTGGCAGTTCCAATATGGGGTTAGATAACATGGAGCCTGCAGATGATGATTTCTTTGAAGGGTTAGATGAAC
TCGCTGCTCAGGCCACCGGGGATTGCTTTTCCGATAACTTTCAGGGGTCTATGCAGCTGCCATGGCTGTCAAA
TAATGCCACAACCACCGCAGCTGGCGGTGTTTGA
>CA08g07730
ATGGAGGAAGATTGGGATCTACATGCAGTGGTCAGAGGCTGCACAGCTAGTTCCACCACCACAACCACCAGC
ACCACCACCACTGCAACTTCATGTTGTAGCTTCCAACCAAGACAAGATGGCAACTTTTTCAGCTTTCAAGATC
CATTTGTGCCAAGATTTGACAACCCCACAAGTGATTTTGAAGAGTTGCATAATCTTTACAAGCCTTTCTTTCCT
AAATCACAAGTACAACCACAACAACAAGTACCTCGTTCTCCTCAAAATAATATAATACCCATTTCACCCCTCT
CTGTTCTTGGTGGACTACAAGATCTATCAGCACCTCAACCAACACTAAAACAACAGCAGCAGCAGCAGCAAC
AACAGCAACAACATATTCATCAGTTTTTTAACAGTACAAGACTAACACAACCCAAACAATCTCTGTCTGTAAA
TGGTTCAACAAATAGTACAATTACTGCTTCTTCTTCACTTGGTGTCTCACATACTCAAAGCCCAAGACCTAAA
AGAAGGAAAAATCAATTAAAGAAGGTATGCCAAGTACCTGCTGAAGGTTTATCTTCTGACATGTGGTCTTGG
AGAAAATATGGACAAAAACCCATCAAAGGCTCTCCATATCCAAGGGGATATTACAGGTGTAGCACGTCAAAG

GGTTGTTTAGCCCGAAAACAAGTGGAGCGAAATAGATCCGACCCGAATATGTTCATTGTCACCTATACAGCTG
AGCACAACCACCCCATGCCAACTCACCGGAATTCCCTCGCCGGAAGTACCCGTCAGAAGCCGGCGAATTCCG
AAGCTGGCACCACTGCAAGCGACTCTAACAAACCTACCAGCTCATCGCCGGTATCTTCGCCGGCGTGCCATTC
TACGGCGACGGAGAAGCAAGAAAGCAGCAGGGAAGAAAAAGAAGACATTTTCGAAGACGAGGACGAAGAA
TTTGGCAGTTCCAATATGGGGTTAGATAACATGGAGCCTGCAGATGATGATTTCTTTGAAGGGTTAGATGAAC
TCGCTGCTCAGGCCACCGGGGATTGCTTTTCCGATAACTTTCAGGGGTCTATGCAGCTGCCATGGCTGTCAAA
TAATGCCACAACCACCGCAGCTGGCGGTGTTTGA

CaWRKY42

>Capana08g001044 [mRNA] locus=Chr08:124129873-124131797
ATGGAGAAAGTTAAAGGATTGGAGAAAAAGAAATTGATTAGTGAGCTAACACAAGGGAAGGAGTTCGTAAA
GCAACTGAAAAAACAGATTGGTCCATTGGCTTCACCTGAAGAATGTGATTTACTACTTGGGAAAATATTGTCA
TCACTAGAGAAATCATTGTCAATTCTTAATTTGAAAGCACTTCTTCTTGAAGGTGGAATTAATGCTAATAATTC
AACATCTTCATGTTCATCAATTTCATTTCTTGGTAATAATAATAGTCCCATGAGTGAAGTTTTTGATTCTCCAA
GTCATCATTTGGACAAAAATATGGTCTCCAAGAAGAGAAAGAAATCACAAGAGACTAATCAAATAACCATTT
CTGGGACGGGGCTTGAAGGTTCACATGAAGATGGATTTAGTTGGAGAAAATATGGCCAGAAAGATATTTTAG
GGGCTAATCATCCAAGGGCTTATTATCGGTGCACACACAGGCATACACAGGGGTGTTTGGCAACAAAACAAG
TCCAAAGATCAGATGGAAACTCAACAATCTTTGAGGTAACATACAAAGGAAGGCACAGTTGCAAAGTTGCAC
AATCAGATATCTTTTCACTTAATAATCAAAAACGCCAGAAACACAACAAAAAACAAGAACAAGAGATGGTAA
TATTCAACTCTACACCAAACCATGACGCAGAAAACTTCAACATCACAACAAAAGAAGAAGTTTTCACACCAT
TTTCATTTCCACCTACACCCCTAAACCTAGAGAATATTGAAGAGACAAAATTCTTTTGTGATTCCATGGTGCC
ATTGTTAACGTCCCAAAATCAAGAATTTGGAATGGACTACATGACTCTGCACTGCTCAAATTCAGATCTCACT
GAATTGATCTCGACCCCGACCCCAACCTCGATTTCAAATTCCTCATTTGTCGGAGATTGGGATTTGTCTGAGG
ATTTTGAACCTAATGTCATATTTGACATTGAAGAGTTCTTTAGTTAA
>CA08g08240

ATGGAGAAAGTTAAAGGATTGGAGAAAAAGAAATTGATTAGTGAGCTAACACAAGGGAAGGAGTTCGTAAA
GCAACTGAAAAAACAGATTGGTCCATTGGCTTCACCTGAAGAATGTGATTTACTACTTGGGAAAATATTGTCA
TCACTAGAGAAATCATTGTCAATTCTTAATTTGAAAGCACTTCTTCTTGAAGGTGGAATTAATGCTAATAATTC
AACATCTTCATGTTCATCAATTTCATTTCTTGGTAATAATAATAGTCCCATGAGTGAAGTTTTTGATTCTCCAA
GTCATCATTTGGACAAAAATATGGTCTCCAAGAAGAGAAAGAAATCACAAGAGACTAATCAAATAACCATTT
CTGGGACGGGGCTTGAAGGTTCACATGAAGATGGATTTAGTTGGAGAAAATATGGCCAGAAAGATATTTTAG
GGGCTAATCATCCAAGGGCTTATTATCGGTGCACACACAGGCATACACAGGGGTGTTTGGCAACAAAACAAG
TCCAAAGATCAGATGGAAACTCAACAATCTTTGAGGTAACATACAAAGGAAGGCACAGTTGCAAAGTTGCAC
AATCAGATATCTTTTCACTTAATAATCAAAAACGCCAGAAACACAACAAAAAACAAGAACAAGAGATGGTAA
TATTCAACTCTACACCAAACCATGACGCAGAAAACTTCAACATCACAACAAAAGAAGAAGTTTTCACACCAT
TTTCATTTCCACCTACACCCCTAAACCTAGAGAATATTGAAGAGACAAAATTCTTTTGTGATTCCATGGTGCC
ATTGTTAACGTCCCAAAATCAAGAATTTGGAATGGACTACATGACTCTGCACTGCTCAAATTCAGATCTCACT
GAATTGATCTCGACCCCGACCCCAACCTCGATTTCAAATTCCTCATTTGTCGGAGATTGGGATTTGTCTGAGG
ATTTTGAACCTAATGTCATATTTGACATTGAAGAGTTCTTTAGTTAA

CaWRKY43

>Capana08g001961 [mRNA] locus=Chr08:139093292-139095397
ATGGAAAACAATAACAAATCAGAAAGTGATAATGAGATGGAAATTGATCTTAGGTTGAAGCTTGATGCTAGA
GAGGAAGAAAATGAAGAAAACAAAATTGGAGAACCATCACAATTGACTGAGAAAACACAAGCAAAAGATCA
AGAAATACCAAAAAATGACCAAGAGTTATCGATGCTAGAAAAGGAGATGAAGAGGATGAAAGAGGAGAAC
AAAGTATTGAGGATGGCAGTGGAGCAAACAATGAAAGATTACTATGATCTACAAGCAAAATTCTCAACTATT
CATCAAAATAATCACAAGGATCACAAAAAATTTCTTTCATTGAGTGGAAATGATGATAGTACTACTAGTGAA
GGACTAACTACAAGGGTTCCAAAAATCTTGGATATTATAAATACTACTAATAGAACTTCATCACCAACATCTC
ATGAAGATGATACTATGGATGGTGATCAATTAGGGTTATCTTTGACTTTGGTTAGTAGTAACAGCACTACATC

AAGCAAATTATTGGAAATGCTAGAAGAAGATCAAAGAAAGGAAAAAAAAGAAGACCATCCTACAATTACTC
ATCAAATTCAAAATAATAAATCACAAAATTTGGGAGGATTAACAAGTCATCATGTCACTACTGCTTCACCACC
AAACAGAAAATCTAGGGTTTCCGTCCGGGCAAGATGTGAATCGGCTACAATGAATGATGGCTGCCAATGGAG
GAAGTATGGTCAGAAAATTGCTAAAGGAAGTCCTAATTGTCCTAGAGCTTACTACCGTTGCACGGTGGCGCCT
GGTTGTCCCGTCAGAAAGCAGGTGCAAAGATGTTTGGAGGACATGTCAATATTGATCACAACATATGAAGGA
ACACATAATCATCCACTTCCAGTAGGTGCAACAGCAATGGCATCAACAGCATCAGCAGCAGCTTCATTCATGT
TAGTGGATTCAAGTATTAGCCCTCTTCTTAACAATCCCAATTCCAGCTTAAACCAACCACTTAATTTCCCTAAT
TACCATCATAATTTAGCACCTAATTACCATCATAATATTCCAAACTCATCCTCTTCTTCTTCACTAATACCTTAT
AACCTTTCCATGATAAGAAACAACATCCTTAATTCTAGTGATCCTAATTCACAAGGGAACATAGTACTTGATC
TCACCAAGAATATTTCTAATAACCATCAATTCCCCTTTGCAAGTTCTTCTTCAAATTCTCATGAAATGGGCCAT
TCTAATTGGATGCCTAAATTGCCTAATTATGAAGGGAACAGCCTTTTAGCTGGTCCTAAATTACAAGGTGAAC
ATCATTATAGTCATAATAATAATAATATTCCTCCTACGTTGGCTCATGATGAAAATATGAGTGCAATTGCTGC
TGATCCTAAGTTTAGGGTTGCTGTGGCCGCTGCTATTTCTTCACTCATAAATAAAGATCAAAGCCATTCAACT
GGAGAGAGTAATGGTGGCTCTATTAATAGGCACACAGATTCTTGA
>CA00g60490
ATGGAAAACAATAACAAATCAGAAAGTGATAATGAGATGGAAATTGATCTTAGGTTGAAGCTTGATGCTAGA
GAGGAAGAAAATGAAGAAAACAAAATTGGAGAACCATCACAATTGACTGAGAAAACACAAGCAAAAGATCA
AGAAATACCAAAAAATGACCAAGAGTTATCGATGCTAGAAAAGGAGATGAAGAGGATGAAAGAGGAGAAC
AAAGTATTGAGGATGGCAGTGGAGCAAACAATGAAAGATTACTATGATCTACAAGCAAAATTCTCAGCTATT
CATCAAAATAATCACAAGGATCACAAAAATTTTCTTTCATTGAGTGGAAATGATGATAGTACTACTAGTGAAG
GACTAACTACAAGGGTTCCAAAAATCTTGGATATTATAAATACTACTAATAGAACTTCATCACCAACATCTCA
TGAAGATGATACTATGGATGGTGATCAATTAGGGTTATCTTTGACTTTGGTTAGTAGTAACAGCACCACATCA
AGCAAATTATTGGAAATGCTAGAAGAAGATCAAAGAAAGGAGAAAAAAGAAGACCATCCTACAATTACTCA
TCAAATTCAAAATAATAAATCACAAAATTTGGGAGGATTAACAAGTCATCATGTCACTACTGCTTCACCACCA
AACAGAAAATCTAGGGTTTCCGTCCGGGCAAGATGTGAATCGGCTACAATGAATGATGGCTGCCAATGGAGG

AAGTATGGTCAGAAAATTGCTAAAGGAAGTCCTAATTGTCCTAGAGCTTACTACCGTTGCACGGTGGCGCCTG
GTTGTCCCGTCAGAAAGCAGGTGCAAAGATGTTTGGAGGACATGTCAATATTGATCACAACATATGAAGGAA
CACATAATCATCCACTTCCAGTAGGTGCAACAGCAATGGCATCAACAGCATCAGCAGCAGCTTCATTCATGTT
AGTGGATTCAAGTATTAGCCCTCTTCTTAACAATCCCAATTCCAGCTTAAACCAACCACTTAATTTCCCTAATT
ACCATCATAATTTAGCACCTAATTACCATCATAATATTCCAAACTCATCCTCTTCTTCTTCACTAATACCTTAT
AACCTTTCCATGATAAGAAACAACATCCTTAATTCTAGTGATCCTAATTCACAAGGGAACATAGTACTTGATC
TCACCAAGAATATTTCTAATAACCATCAATTCCCCTTTGCAAGTTCTTCTTCAAATTCTCATGAAATGGGCCAT
TCTAATTGGATGCCTAAATTGCCTAATTATGAAGGGAACAGCCTTTTAGCTGGTCCTAAATTACAAGGTGAAC
ATCATTATAGTCATAATAATAATAATATTCCTCCTACGTTGGCTCATGATGAAAATATGAGTGCAATTGCTGC
TGATCCTAAGTTTAGGGTTGCTGTGGCCGCTGCTATTTCTTCACTCATAAATAAAGATCAAAGCCATTCAACT
GGAGAGAGTAATGGTGGCTCTATTAATAGGCACACAGATTCTTGA

CaWRKY44

>Capana09g000676 [mRNA] locus=Chr09:32179774-32181439
ATGGGAGATCAAACGAGCGTTGAAGTCCCACCAACGCCAAATTCTTCGATATCTTCTACTTCTAATGAGGCTG
GAGGGCAAGAAGATTCTTCCAAAATCAAGAAACACATGCAGAATAAAGATGGTCAAGAAGGAAGAGATGAC
AAATCAAAGAAAGAGTGCAAAGCAACAAAGAAAGGAGAAAAAAAGGTAAAAGAACCAAGATTTGCCTTCAT
GACAAAAAGTGAGATTGACAATCTTGAAGATGGTTATAGATGGAGAAAATATGGACAAAAAGCAGTGAAGA
ACAGCCCTTTCCCCAGGAACTATTACAGATGCACAACTCAAAAGTGCAGTGTGAAGAAACGTGTGGAAAGGT
CATATGAAGATGCATCAATTGTGATAACTACATATGAAGGCCAGCACAATCATCATTGTCCAGCAGCCCTTAG
GGGAAATGCATCCTTCTTATCTTCACCACATTTTATGCCTAGTTTTCCTCCACAACTATTTTCCCAAATGCTAA
TTCCACCAACAAGCAACCAAAATCTCCTCATTACTTCTGAAGCCTATAATAATATTAATAATAACAATTATCA
TCAACAAAACCAAGGTTCAGAATACAACCTATTTGGTGGAGGCACAAATGATGCATCATGGATCCAGAAACA
AGAGCCATCCTAG

>CA09g08120
ATGTCTGATAACCCTTTTTATCATGATTACATGGGAACTAGTGGAGGGATCAATACATTTCCTTTTTTTGGTGA
AAATCCCTCAAATTATCATGACCAACCAATTATTCCAAATATTCAAAATCCAAATCATGAGCATCAGTTCGTA
CCTTCTTCTTATATGACTCTCACTGAGTGTTTACATGGCTCTATGGACTACAATACTCTATCAAGTGTTTTTGG
CATGTCTTGCTCATCATCATCCGAAGTTGTTTGTCCACATATCGATAATCAAGGCTCTACTAGAAAAAGTAGC
GTCTCTGCTACTGCTGAACCCATCTTAGATTCGCCTATGGGAGATCAAACGAGCGTTGAAGTCCCACCAACGC
CAAATTCTTCGATATCTTCTACTTCTAATGAGGCTGGAGGGCAAGAAGATTCTTCCAAAATCAAGAAACACAT
GCAGAATAAAGATGGTCAAGAAGGAAGAGATGACAAATCAAAGAAAGAGTGCAAAGCAACAAAGAAAGGA
GAAAAAAAGGTAAAAGAACCAAGATTTGCCTTCATGACAAAAAGTGAGATTGACAATCTTGAAGATGGTTAT
AGATGGAGAAAATATGGACAAAAAGCAGTGAAGAACAGCCCTTTCCCCAGGAACTATTACAGATGCACAACT
CAAAAGTGCAGTGTGAAGAAACGTGTGGAAAGGTCATATGAAGATGCATCAATTGTGATAACTACATATGAA
GGCCAGCACAATCATCATTGTCCAGCAGCCCTTAGGGGAAATGCATCCTTCTTATCTTCACCACATTTTATGCC
TAGTTTTCCTCCACAACTATTTTCCCAAATGCTAATTCCACCAACAAGCAACCAAAATCTCCTCATTACTTCTG
AAGCCTATAATAATATTAATAATAACAATTATCATCAACAAAACCAAGGTTCAGAATACAACCTATTTGGTGG
AGGCACAAATGATGCATCATGGATCCAGAAACAAGAGCCATCCTAG

CaWRKY45

>Capana09g001251 [mRNA] locus=Chr09:129708552-129712643
ATGGCTTCTTCAGGTGGAAATACGAACACTTTTATGAATTCTTTCAACAGCAACTATTCATTTTCATCTTCCCA
ATTCATGACTTCTTCTTTTAGTGACCTTCTTTCTGATAATAATGATGATAATAACAACAACAGGAACTGGGGA
TTTAGTTATCAGAGAATTATGAATTCAATTAACAAAGATGAGGTTCCAAAGTTCAAGTCTTTTCCACCTTCTTC
TTTGCCTATGATCTCTTCTTCATCACCAGCTTCTCCTTCTTCTTATCTTGCTTTTCCTCATTCTTTAAGTCCATCG
GTTCTTTTGGACTCACCAGTTTTGTTTAACAATTCCAATACTCTTCCATCACCAACAACAGGGAGTTTTGGTAG
TTTGAATTCCAAGGAGGATAATTCAAGGACTTCTGATTTCTCTTTCCATAGTAGGCCTGCTACTTCATCATCAA

TATTTCACTCTTCTGCTCCAAGAAACTCATTGGACGACTTAATAACAAGGCAACAACAGACTACTGAATTCTC
CACAGCAAAAATTGGGGTGAAATCAGAAGTAGCTCCAATTCAAAGTTTCTCCCAAGAGAACATGCAGAATAA
TCCTGCCCCAATGCATTACTGTCAACCATCTCAATATGTTAGAGAACAGAAGGCAGAAGATGGTTATAATTGG
AGGAAATATGGGCAAAAGCAAGTGAAAGGAAGTGAGAATCCGCGAAGCTATTACAAGTGTACGTTTCCTAAT
TGTCCTACAAAGAAGAAGGTTGAAAGGAACTTGGATGGACACGTTACTGAGATAGTCTATAAGGGGAGCCAT
AATCATCCAAAGCCTCAATCCACCAGAAGATCATCCGCACAATCGATTCAGAACCTTGCTTACTCCAACTTGG
ATATAACAAATCAGCCAAATGCTTTTCTTGAAAATGCTCAAAGGGATTCCTTGGCCGTAACAGACAATTCTTC
AGCTTCTTTTGGAGATGAGGATGTTGATCAAGGGTCTCCTATCAGTAAGTCAGGAGAAAATGATGAAAATGA
ACCTGAGGCAAAGAGATGGAAGGGAGACAATGAAAACGAGGTCATATCATCTGCAAGTAGAACAGTACGTG
AACCTAGAATCGTAGTACAAACCACAAGTGACATTGATATTCTTGATGATGGTTATAGATGGAGAAAATATG
GACAAAAAGTTGTCAAAGGCAATCCAAACCCAAGAAGTTACTACAAATGCACATTTATTGGCTGTCCAGTTA
GGAAGCATGTAGAGCGAGCATCGCATGATCTAAGAGCAGTGATCACAACTTATGAAGGAAAACATAACCATG
ATGTTCCTGCAGCACGTGGTAGCGGTAGTTACTCCATGAATAAACCTCCATCTGGAAGCAACAATAACATGCC
AGTAGTTCCAAGGCCTTCACTGTTGGCTAACAATTCTAATCAAGGAATGAATGTTAGTAACACTTTATTTAAC
ACAGCACAGGTTGAACCACCAATCACCTTGCAGATGCTACAAAGCTCTGGAAGTTCAAGTTATTCAGGATTTG
GAACCTCATCAGGATCTTATATGAATCAAATGCAGCCCACGAACAATTCCAAGCTGATAAGCAAAGAAGAAC
CTAAAGATGATTTATTCTTCAGCTCTTTCCTTAACTGA
>CA09g11930
ATGACTTCTTCTTTTAGTGACCTTCTTTCTGATAATAATGATGATAATAACAACAACAGGAACTGGGGATTTA
GTTATCAGAGAATTATGAATTCAATTAACAAAGATGAGGTTCCAAAGTTCAAGTCTTTTCCACCTTCTTCTTTG
CCTATGATCTCTTCTTCATCACCAGCTTCTCCTTCTTCTTATCTTGCTTTTCCTCATTCTTTAAGTCCATCGGTTC
TTTTGGACTCACCAGTTTTGTTTAACAATTCCAATACTCTTCCATCACCAACAACAGGGAGTTTTGGTAGTTTG
AATTCCAAGGAGGATAATTCAAGGACTTCTGATTTCTCTTTCCATAGTAGGCCTGCTACTTCATCATCAATATT
TCACTCTTCTGCTCCAAGAAACTCATTGGTATAA
>CA09g11940

ATGAAACAGGACGACTTAATAACAAGGCAACAACAGACTACTGAATTCTCCACAGCAAAAATTGGGGTGAA
ATCAGAAGTAGCTCCAATTCAAAGTTTCTCCCAAGAGAACATGCAGAATAATCCTGCCCCAATGCATTACTGT
CAACCATCTCAATATGTTAGAGAACAGAAGGCAGAAGATGGTTATAATTGGAGGAAATATGGGCAAAAGCA
AGTGAAAGGAAGTGAGAATCCGCGAAGCTATTACAAGTGTACGTTTCCTAATTGTCCTACAAAGAAGAAGGT
TGAAAGGAACTTGGATGGACACGTTACTGAGATAGTCTATAAGGGGAGCCATAATCATCCAAAGCCTCAATC
CACCAGAAGATCATCCGCACAATCGATTCAGAACCTTGCTTACTCCAACTTGGATATAACAAATCAGCCAAAT
GCTTTTCTTGAAAATGCTCAAAGGGATTCCTTGGCCGTAACAGACAATTCTTCAGCTTCTTTGGAGATGAGG
ATGTTGATCAAGGGTCTCCTATCAGTAAGTCAGGAGAAAATGATGAAAATGAACCTGAGGCAAAGAGATGGT
AA
>CA09g11950
ATGTTCTTTCGTGTTAATTGTCCTTTCAGGAAGGGAGACAATGAAAACGAGGTCATATCATCTGCAAGTAGAA
CAGTACGTGAACCTAGAATCGTAGTACAAACCACAAGTGACATTGATATTCTTGATGATGGTTATAGATGGA
GAAAATATGGACAAAAAGTTGTCAAAGGCAATCCAAACCCAAGAAGTTACTACAAATGCACATTTATTGGCT
GTCCAGTTAGGAAGCATGTAGAGCGAGCATCGCATGATCTAAGAGCAGTGATCACAACTTATGAAGGAAAAC
ATAACCATGATGTTCCTGCAGCACGTGGTAGCGGTAGTTACTCCATGAATAAACCTCCATCTGGAAGCAACAA
TAACATGCCAGTAGTTCCAAGGCCTTCACTGTTGGCTAACAATTCTAATCAAGGAATGAATGTTAGTAACACT
TTATTTAACACAGCACAGGTTGAACCACCAATCACCTTGCAGATGCTACAAAGCTCTGGAAGTTCAAGTTATT
CAGGATTTGGAACCTCATCAGGATCTTATATGAATCAAATGCAGCCCACGAACAATTCCAAGCTGATAAGCA
AAGAAGAACCTAAAGATGATTTATTCTTCAGCTCTTTCCTTAACTGA

CaWRKY46

>Capana09g001790 [mRNA] locus=Chr09:205298779-205300747
ATGGAGGAAGAAGGACTTATCAAGAATTCATGGTCTTATGAAGATGAGTTGATAAAAGAGCTTCTTGATGAT
GAATCACCATTCTTGCTAGCTCCTCATGAAGAATATTATTCAACTTCTTCAAGTGAGACTAGTTATTCACTTGA

TGTAACAAAGAGCTCTATTTCTTCTCTTTCCAAAGGGTCATTTATTGATGATATAGAGAGTGATGTGTCCATGA
CAAGAAATGGTGTTCAATCTCATGATGTTTCACATGATGCTAGGAATATAGGGTTGGAAAGAGGGTTGAACTT
GATGATGAATAAGCAAGAGGCTCTTGAGAATAAATATACTTTGAGAATAAAGACTTGTGGTAATGCAATGGC
TGATGATGGTTATAAGTGGAGAAAATATGGCCAAAAATCCATCAAGAATAGCCCATATCCCAGGAGTTACTA
CAAATGCACTAATCCAAGGTGTGGAGCCAAAAAACAAGTTGAGAGGTCCAGCAATGAGCCAGACACTTTCAT
AATCACTTATGAAGGACTTCATCTACATTTTGCTTACCCATTTATCACTCTCAATCCACCTCAATTTCTTGACC
AGCCCACTAAAAAGCCCAAATTAACAAACCCCAAAGCCCAAAACAATGAAGAAAATGCAAGTGAAGTTGAT
GAAAGCCCAAAATTTGTCAATCCAAGCCCAATTGTGGACCTTGAAGATGGATTGGGTTTTGGTGAAATGGGCT
CACAAGGGTTACTTGAAGATATGGTGCCATTAATGATTAGAAACCCATTTATTAAGCCCACAAATTCCTATTC
TTCATCTTGCTCTTATTCATCCCCACCAACTTCTCCTTCATTTTCTTGGTCAAATAATTAG
>CA09g05110
ATGAATAAGCAAGAGGCTCTTGAGAATAAATATACTTTGAGAATAAAGACTTGTGGTAATGCAATGGCTGAT
GATGGTTATAAGTGGAGAAAATATGGCCAAAAATCCATCAAGAATAGCCCATATCCCAGGAGTTACTACAAA
TGCACTAATCCAAGGTGTGGAGCCAAAAAACAAGTTGAGAGGTCCAGCAATGAGCCAGACACTTTCATAATC
ACTTATGAAGGACTTCATCTACATTTTGCTTACCCATTTATCACTCTCAATCCACCTCAATTTCTTGACCAGCC
CACTAAAAAGCCCAAATTAACAAACCCCAAAGCCCAAAACAATGAAGAAAATGCAAGTGAAATTGATGAAA
GCCCAAAATTTGTCAATCCAAGCCCAATTGTGGACCTTGAAGATGGATTGGGTTTTGGTGAAATGGGCTCACA
AGGGTTACTTGAAGATATGGTGCCATTAATGATTAGAAACCCATTTATTAAGCCCACAAATTCCTATTCTTCA
TCTTGCTCTTATTCATCCCCACCAACTTCTCCTTCATTTTCTTGGTCAAATAATTAG

CaWRKY47

>Capana10g000205 [mRNA] locus=Chr10:3656310-3659300
ATGGGCGGGTTCGATGATCATGTTGCTATCTTCGGAGACTGGATAACACCTAGCCCGAGCCCAAGAGCCTTCG
TGTCTTCACTGCTAGTCGATGATGTTGGAGGATGGTTACCTCTTATGGAGCACACTAATGAAAGTAACTGCAG

AAACTTCAATGCCGAACCTCAACAGAATGTCACCGCCTTGTGCAGCACTGATGGAAAGGATGGGGCACGGGC
TGGTGCTTCGACTGATCAAACAGTCAAGTCGAGTGCACCATCAGAGCAGAAACCGAGTACTCGTGGAGGAGG
GCTCATGGAAAGAATGGTAGCTAGATCTGGATTTCATGCTCCAAGGCTGAATACCGATGGCCTTAGACCTCCT
GTTCTTTCACAGAATCAAGAAGCTAGGTCTCCTTATTTGACTATTCCTCCGGGTCTCAGTCCATCAGTCCTATT
ATATTCACCTGTTTTGATCTATAATCCACTGGTTTGTCCATCTCCTACAACTGGACTATTACCATTAGCATCGG
GCGATGAGAGTAAAAGTCTTATGTTGACGGCTGGAATTGCAGATAAGAGGAAAGAGACTGCTTTTGGCAGCA
ATACTTCATCTTCCTTCTCTTCCAATCCAGTAAATCCGTCCAGTGATCTTTCTCAGCAACTCTTACCTCAAATTG
AGGTTTCAGCTCATCCAAATAACTCTCTTCAACCTCAAAGTATGGAAGTAACTCAAAGCGAACAGATACGTCA
TGGAATATCTAAGTTCCCTATGTTGTCTACTGAAGAGGATTTCCGGGGTAGTCATATCAAGCCAGAGGTAAGG
CCCTTTAATATAGTTGGTGGTAGTATGCAACATTCTCAGACTCTTGATGAGCAGAAAGATGAGGACACTAAGC
AAAGAGGAGGTGGAGACTCAAAAGATGTCAATCCTCCTGCCGAAGATGGTTATAACTGGAGAAAGTATGGAC
AAAACCAAGCTAATGGAAGAACGTATCCTCGGAGTTATTATAAGTGCGCGTATCCAAAGTGTCCTGTGAAGA
AGAGAGTAGGGGGATATCATGACTGTCAAGTCATGGAAATTATATACAAGGGGATTCACAATCACCCTAAGC
CACTCTCAAACCCGATATCAGCCCTCGGATCTTTAAACTCATTTGGTGACGTGCAACTAGACAATGTGGATCC
AAGTGGAACAGGTTTTAACAGTGAGCTGGCTTTGGCAACTAGCCAACAAGGACCTACTGCTAAAGGCCTCAT
GTGGAGCAACAACAAACTTGAAGCAACATCATTAGCAGCTTTGCACTCTGAGTACTGCAGTGGATCTACCACT
TTACAATCAAAGGGTGCTCAGCAGGGATCAGCAGATGCAGTTGAAGTATCATCAGTGTTTTCAAATGATGAA
GATGATCATGGTACCCGTGGCAGTGTATTACTAGGCTATGATGGTGCAGAAGATGAGTTCGAGCCCAAAAGA
AGGAGTTACTACAAGTGCACAAGTTCTGGCTGCAATGTCAGGAAGCACATCCAGAGGTCCCCATATGATCAG
AAGTCTGTTATCACCACTTATGATGGGAAGCACTACCATGAAGTTCCTCCAGCACGCACCAGCAGCCAAGGT
AGCTCGGGAGCGTCAAAGTCTCCTTCCAACCCGATAACTACTGATGCTCAAAGTCATGTAGGTAGGCCTGAGC
CCACACAAGTTCAGAACACCAAGCCCGCACAAGTTCAGAACACCAAGTCCACACAAGTTCAGAACACCAACG
AGCGTTATGGAAGAGCTCCACAAATCCAGAACACCAACGAGCGTTATGGAAGAGCTCCACAAGTTCAGAACA
CCAACGAGCGTTATGGAAGAGTTCCCTCACTTGGATCAGCTGGTCCTATCTCAGGATTTGATTGTTTCGGAAC
TAACGAGCAGCAAGATCTATCCAGTCTTGCTATGGCCGGATTTAACTCTAATCAGCACCAGTTTTCAGTTCCT

CTCAATCCATACATAGGATGGCAACGACCTGTGAATGATGTTGGTTTCGTGCTTCCAGAAGGAGAAGCAATG
CCAGATCCTAATTTGAACTACTCCAATGGTTCATCAACTTATCAGAAAATTATGAATGGATTGCCTCCTCAGA
TGTAA
>CA10g00950
ATGGGCGGGTTCGATGATCATGTTGCTATCTTCGGAGACTGGATAACACCTAGCCCGAGTCCAAGAGCCTTCG
TGTCTTCACTGCTAGTCGATGATGTTGGAGGATGGTTACCTCTTATGGAGCACACTAATGAAAGTAACTGCAG
AAACTTCAATGCCGAACCTCAACAGAATGTCACCGCCTTGTGCAGCACTGATGGAAAGGATGGGGCACGGGC
TGGTGCTTCGACTGATCAAACAGTCAAGTCGAGTGCACCATCAGAGCAGAAACCGAGTACTCGTGGAGGAGG
GCTCATGGAAAGAATGGTAGCTAGATCTGGATTTCATGCTCCAAGGCTGAATACCGATGGCCTTAGACCTCCT
GTTCTTTCACAGAATCAAGAAGCTAGGTCTCCTTATTTGACTATTCCTCCGGGTCTCAGTCCATCAGTCCTATT
ATATTCACCTGTTTTGATCTATAATCCACTGGTTTGTCCATCTCCTACAACTGGACTATTACCATTAGCATCGG
GCGATGAGAGTAAAAGTCTTATGTTGACGGCTGGAATTGCAGATAAGAGGAAAGAGACTGCTTTTGGCAGCA
ATACTTCATCTTCCTTCTCTTCCAATCCAGTAACCACTACTCTCTCTCAGACACACACATACACATACACACAC
ATTGCATGCCAGTTTCCTGCCGGTTTCCATGATCTTTCTCAGCAACTCTTACCTCAAATTGAGGTTTCAGCTCA
TCCAAATAACTCTCTTCAACCTCAAAGTATGGAAGTAACTCAAAGCGAACAGATACGTCATGGAATATCTAA
GTTCCCTATGTTGTCTACTGAAGAGGATTTCCGGGGTAGTCATATCAAGCCAGAGGTAAGGCCCTTTAATATA
GTTGGTGGTAGTATGCAACATTCTCAGACTCTTGATGAGCAGAAAGATGAGGACACTAAGCAAAGAGGAGGT
GGAGACTCAAAAGATGTCAATCCTCCTGCCGAAGATGGTTATAACTGGAGAAAGTATGGACAAAACCAAGCT
AATGGAAGAACGTATCCTCGGAGTTATTATAAGTGCGCGTATCCAAAGTGTCCTGTGAAGAAGAGAGTAGGG
GGATATCATGACTGTCAAGTCATGGAAATTATATACAAGGGGATTCACAATCACCCTAAGCCACTCTCAAACC
CGATATCAGCCCTCGGATCTTTAAACTCATTTGGTGACGTGCAACTAGACAATGTGGATCCAAGTGGAACAGG
TTTTAACAGTGAGCTGGCTTTGGCAACTAGCCAACAAGGACCTACTGCTAAAGGCCTCATGTGGAGCAACAA
CAAACTTGAAGCAACATCATTAGCAGCTTTGCACTCTGAGTACTGCAGTGGATCTACCACTTTACAATCAAAG
GGTGCTCAGCAGGGATCAGCAGATGCAGTTGAAGTATCATCAGTGTTTTCAAATGATGAAGATGATCATGGT
ACCCGTGGCAGTGTATTACTAGGCTATGATGGTGCAGAAGATGAGTTCGAGCCCAAAAGAAGGAAGTCTACT

GTGTCGGATACAAGTGGCACCATCAGAGCAATCAGGGAGCCAAGAGTTGTGGTGCATACTATCAGTGAGGTA
GACATCATTGATGATGGATATCGCTGGCGCAAGTACGGGCAAAAGGTGGTTAAAGGCAATCCAAATCCAAGG
AGTTACTACAAGTGCACAAGTTCTGGCTGCAATGTCAGGAAGCACATCCAGAGGTCCCCATATGATCAGAAG
TCTGTTATCACCACTTATGATGGGAAGCACTACCATGAAGTTCCTCCAGCACGCACCAGCAGCCAAGGTAGCT
CGGGAGCGTCAAAGTCTCCTTCCAACCCGATAACTACTGATGCTCAAAGTCATGTAGGTAGGCCTGAGCCCAC
ACAAGTTCAGAACACCAAGCCCGCACAAGTTCAGAACACCAAGTCCACACAAGTTCAGAACACCAACGAGC
GTTATGGAAGAGCTCCACAAGTCCAGAACACCAACGAGCGTTATGGAAGAGCTCCACAAGTTCAGAACACCA
ACGAGCGTTATGGAAGAGTTCCCTCACTTGGATCAGCTGGTCCTATCTCAGGATTTGATAGTTTCGGAACTAA
CGAGCAGCAAGATCTATCCAGTCTTAATCAGCACCAGTTTTCAGTTCCTCTCAATCCATACATAGGATGGCAA
CGACCTGTGAATGATGCTGGTTTCGTGCTTCCAGAAGGAGAAGCAATGCCAGATCCTAATTTGAACTACTCCA
ATGGTTCATCAACTTATCAGAAAATTATGAATGGATTGCCTCCTCAGATGTAA

CaWRKY48

>Capana10g000754 [mRNA] locus=Chr10:46615318-46617494
ATGGAAGAAGATTGGGATCTACATGCCGTGGTCAGAAGTTGCACCCCCGCTAACACCGCCAGCACCACAACG
AATGATTGTGATCTAGTTCATGGTATATTAAAAGTAGACGGTGCTCTGATGAAATCTTTACGAATAGAAAAAG
ATTATCGTCAATTTGATAATGATCAAATGACATTGCAGAGGGGCTCTACCACCACTTCTTGCAATCCCACAAA
CAATATTCATTATTTATGCAGCTTCCAACCAAGACCAAATGATAACAACAACAACTCTTTATTTTGCTTTAAA
GATCTGCTAGAGCAAAGAATATTGACCACAAATAGTGCTACTGATTTCGAAGAGTCGCATGAATTGTGCAAG
CCCTTCTTTACTGCATCAGAAAGTTTAACCATTTCATCACCACGGAGGGGATTACCTATATCACCTATCTCTGT
TCTTGGACGACTACAAGATCTACCACCATCGTCGCAGCAGCAGCAACAACAACATCTTCATCAGCTTACTAAT
ACAAAACCAATTCAGCCTAAGAGACCTCTGTCGTCTCTAAATGGTTCAATAACTAATTGTACTTTACATGCTC
AAAGTTCAAGAACTAAAAGAAGGAAGAACCAATTGAAGAAGGTATGCCAAGTAGCTGCTGATGCTTTATCTT
CTGATATGTGGTCTTGGAGAAAATATGGGCAAAAACCCATTAAAGGTTCCCCATACCCAAGGGGTTATTACA

AATGTAGCACTTCAAAGGCTTGTTTGGCCCGCAAACAAGTGGAGCGAAATAGATCCGACCCGAATATGTTCA
TTGTCACTTATACAGCTGAGCACAATCATCCTATGCCTACGCACAGAAATTCCTTAGCCGGAATCAGCCGCCA
CAAAACGGCGAATCCCAACAAACCCACCAGCTTATCGCCGGCGACCAACTCTCCGGCACCGGAAAATCAAGA
AAGCAGCAGGGATGACAAAGAGGATATTTTTGAAGATGACGACGATGAATTTGGTAAAACTGAACCGGACG
ATGATTTCTTCGATGGTTTGGATGAGCTTGTAATCCAGGCTACCGGAGATAGCTTGCCGGAGAAATTTTCGGG
GACTTTGCAGTTCCCTTGGTTGGTGAATAATGCCGCTACCACGGCGGCCGGCGGTGGTTGA
>CA10g06160
ATGGAAGAAGATTGGGATCTACATGCCGTGGTCAGAAGTTGCACCCCCGCTAACACCGCCAGCACCACAACG
AATGATTGTGATCTAGTTCATGGTATATTAAAAGTAGACGGTGCTCTGATGAAATCTTTACGAATAGAAAAAG
ATTATCGTCAATTTGATAATGATCAAATGACATTGCAGAGGGGCTCTACCACCACTTCTTGCAATCCCACAAA
CAATATTCATTATTTATGCAGCTTCCAACCAAGACCAAATGATAACAACAACAACTCTTTATTTTGCTTTAAA
GATCTGCTAGAGCAAAGAATATTGACCACAAATAGTGCTACTGATTTCGAAGAGTCGCATGAATTGTGCAAG
CCCTTCTTTACTGCATCAGAAAGTTTAACCATTTCATCACCACGGAGGGGATTACCTATATCACCTATCTCTGT
TCTTGGACGACTACAAGATCTACCACCATCGTCGCAGCAGCAGCAACAACAACATCTTCATCAGCTTACTAAT
ACAAAACCAATTCAGCCTAAGAGACCTCTGTCGTCTCTAAATGGTTCAATAACTAATTGTACTTTACATGCTC
AAAGTTCAAGAACTAAAAGAAGGAAGAACCAATTGAAGAAGGTATGCCAAGTAGCTGCTGATGCTTTATCTT
CTGATATGTGGTCTTGGAGAAAATATGGGCAAAAACCCATTAAAGGTTCCCCATACCCAAGGGGTTATTACA
AATGTAGCACTTCAAAGGCTTGTTTGGCCCGCAAACAAGTGGAGCGAAATAGATCCGACCCGAATATGTTCA
TTGTCACTTATACAGCTGAGCACAATCATCCTATGCCTACGCACAGAAATTCCTTAGCCGGAATCAGCCGCCA
CAAAACGGCGAATCCCAACAAACCCACCAGCTTATCGCCGGCGACCAACTCTCCGGCACCGGAAAATCAAGA
AAGCAGCAGGGATGACAAAGAGGATATTTTTGAAGATGACGACGATGAATTTGGTAAAACTGAACCGGACG
ATGATTTCTTCGATGGTTTGGATGAGCTTGTAATCCAGGCTACCGGAGATAGCTTGCCGGAGAAATTTTCGGG
GACTTTGCAGTTCCCTTGGTTGGTGAATAATGCCGCTACNNNNNGGTGGTTGACTTGA

CaWRKY49

>Capana10g001220 [mRNA] locus=Chr10:128628965-128629689
ATGGAGTTCAATTATTGGTGGTATAATTATGTGTGTGTGTCTAACCAATGCTCGCCAAATATTGTGCGCAGAA
AAACTTCAGCTACAACCATAAAAGAAGCCTCGACTTTAGTAGATGATGGCCATGTTTGGAGAAAATATGGTC
AGAAAGAAATACTCAATTTCCCACATCCAAGGAACTATTATAGATGTACCCATAAATTTGATCGAGGATGCG
AAGCAACTAAACAAGTGCAAAGAATCCAAGAAAATCCACCAAAGTTTCGTACCACATACCAAGGTCATCACT
CGTGCACAACTTATCCTTCGATTTCTCAAATACTCTTTGATTCTTCAACAAATGAGGATTGTTCGGTGTTACTA
AGTTTCAACACCAATAAAATTAATTATCAGCAACCATATATTCACTCATTTCATTCAACAAAACAAGAAACTA
AAGGGGAGATCTCTTCAAATTGTTTTTCCCCTAATGTTGGCCAAAGTCAATCCGATGATCATCTGATTAGAGC
AGCATTGTCACCAGGGTCGTCCGATCATGATGTCAACTACTCTTCTTGTACTACTAATTGTAGTTTGGAGATGG
AGATGAAGATAGATATGATGGTGGACTCTGTTGATTTTGAGGATTTAATTCCTTTTGATTTTTGA
>CA10g06890
ATGAAGTTCAATTATTGGTGGTATAATTATGTGTGTGTGTCTAACCAATGCTCGCCAAATATTGTGCGCAGAA
AAACTTCAGCTACAACCATAAAAGAAGCCTCGACTTTAGTAGATGATGGCCATGTTTGGAGAAAATATGGTC
AGAAAGAAATACTCAATTTCCCACATCCAAGGAACTATTATAGATGTACCCATAAATTTGATCAAGGATGCG
AAGCAACTAAACAAGTGCAAAGAATCCAAGAAAATCCACCAAAGTTTCGTACCACATACCAAGGTCATCACT
CGTGCACAACTTATCCTTCGATTTCTCAAATACTCTTTGATTCTTCAACAAATGAGGATTGTTCGGTGTTACTA
AGTTTCAACACCAATAAAATTAATTATCACCAACCATATATTCACTCATTTCATTCAACAAAACAAGAAACTA
AAGGGGAGATCTCTTCAAATTGTTTTTCCCCTAATGTTGGCCAAAGTCAATCCGATGATCATCTGATTAGAGC
AGCATTGTCACCAGGGTCGTCCGATCATGATGTCAACTACTCTTCTTGTACTACTAATTGTAGTTTGGAGATGG
AGATGAAGATAGATATGATGGTGGACTCTGTTGATTTTGAGGATTTAATTCCTTTTGATTTTTGA

CaWRKY50

>Capana10g001548 [mRNA] locus=Chr10:164830295-164833345
ATGGAGTCGTTTTTATTACAAAATACAATATCTGATCTGGAGAAGGTAATGGAAGAGTTGAATCGCGGCAAG

AAATTTACGCGCCGGCTAAGAGAGATAATAAAGAAACCTAAGATAAATGTGGGTAACGAAGATGCGTATATG
TCTAGCGCTGAGGATTTGGTGGGGAAAATAATGAATTCATTTTGTGCGTCTCTATCGATATTAAGCTCTGAAG
AATCTACTGAAGAAGTTTCTCAAAAGTCGATGGAAGACTCAAGTGGTAGTTGCAAGACTTCGTCACTTAAAG
ATCGACGAGGATGCTACAAGAGAAGGAGAACTTTAGAAACAAGCATAAAAGAAACCTCAACTTTGGTGGAT
GATGGCCATGGTTGGAGAAAATATGGTCAAAAACAGATCCTCAATGCCAAATTTCCAAGGAACTATTTTAGA
TGCACTCATAAATTTGATCAAGGATGCCAAGCAAGCAAACAGGTGCAAAGAATTCAAGAAAATCCACCACTG
TTTCGTATAACATATTATGGTCATCACACTTGCAAAACTTTTCCTAAAGTTTCTCAAATGATATTTGATTCTCC
AAATGATCATGAAGATTCTAATTCAGTCCTACTTAACTTTAATTCTGGCAATAATCATCACCAGTTTTTGGATA
TGACGGTGGAAACTCTTGATTTTGTGGATTTGTCTTTTGAATTTTGA

CaWRKY51

>Capana10g001791 [mRNA] locus=Chr10:183798726-183801612
ATGGAAGTCAATGAAGCAGTAAAAATACCTATAGCTAGACCAGTCGCTTCAAGGCCAAGATGTCCTGTTTAC
AAATCTTTCTCAGAGCTCCTGACTGGTACAGTAGATATATCATCGACAAATGTTCATTCTGAAATGGCTGTTA
CCGCCATAAGACCAAAGACTATCAGGTTGAAGCCTGCAACAAACCATGCTTTAGTTGGAGAGCCTTCTTCACA
GGTTGGCGTGTCCAAGGCACCAGTTGGTTTTGGCTCTGATAACATCTTGCAATCGGTAGAGAAGCCCAAGGTC
CTGTATAAGCCTATAGGTAAACTTGCACAAAGGAAAACAATTCCTCTACTTGAAAATAAGGGAAGCTCCGTA
TCTGATCAGCAACGAGTAATAGCTGACTCTGAGGCTCATGTTCAATCAGCAAATGAAGTTAAGCAACAACAT
GACCCTACGACAGAATCTAAACAAAGTCTCTCAGAAAAATCAGGACAGGACAAAAAAAAAGTGCGTTCAAC
AATTGTATCTGGGAGCACAGAGGAGGTTGCACAATCTTTGATCAACACAAGTAATGTTGATCGTCCTAGTTAT
GATGGATATAATTGGAGAAAATACGGACAAAAGAAAGTTAAAGGAAGCGAATACCCAAGAAGTTACTACAA
GTGCACACATCTGAAGTGTCCTGTGAAAAAGAAGGTTGAAAGATCATATGACGGCCAGATAACTGAAATTGT
TTACAGGGGTGACCACAACCACCCAAAGCCTCAGCCTCCAAAGCGCAACTTGTCAGATGGCCATAGGCAAAC
AGCCATATGCAATGACACTTCTAAAGAAACAATAACCCTGCATGGGTCAGAAATTCTGAAGATTCTGCTGT

AGGAAGTAAAAAATTGAAGGCTACTTGTGATGAACAAAAAAGTAAAAGAAGGAAAATTAAAGGTCCATCCA
GTGGAGCAGGTACATCAGGGGAAAGTACATTTCCTTATATACCAAACCAAAGTACTACTGACTCGGAAATTA
CCGAGGACGGTTTTCGCTGGAGAAAATATGGCCATAAGGTTGTGAAGGGAAGTTCATATCCCAGGAGCTATT
ACAGATGCACAAGTCCTAAATGCAGTGTGCGGAAGTTTGTTGAAAGAACCACGGATGATCCCAGAGCCTTTA
TTACTACATACGAGGGAAAACACAACCATGGTGTTCCAAACAGAAGACCAAATTCAGAGGCATCCAAAACAA
GCTCAAAATCTTCAGCTATGAAAGAGAAATTATAG
>CA10g14950
ATGGAAGTCAATGAAGCAGTAAAAATACCTATAGCTAGACCAGTCGCTTCAAGGCCAAGATGTCCTGTTTAC
AAATCTTTCTCAGAGCTCCTGACTGGTACAGTAGATATATCATCGACAAATGTTCATTCTGAAATGGCTGTTA
CCGCCATAAGACCAAAGACTATCAGGTTGAAGCCTGCAACAAACCATGCTTTAGTTGGAGAGCCTTCTTCACA
GGTTGGCGTGTCCAAGGCACCAGTTGGTTTTGGCTCTGATAACATCTTGCAATCGGTAGAGAAGCCCAAGGTC
CTGTATAAGCCTATAGGTAAACTTGCACAAAGGAAAACAATTCCTCTACTTGAAAATAAGGGAAGCTCCGTA
TCTGATCAGCAACGAGTAATAGCTGACTCTGAGGCTCATGTTCAATCAGCAAATGAAGTTAAGCAACAACAT
GACCCTACGACAGAATCTAAACAAAGTCTCTCAGAAAAATCAGGACAGGACAAAAAAAAAGTGCGTTCAAC
AATTGTATCTGGGAGCACAGAGGAGGTTGCACAATCTTTGATCAACACAAGTAATGTTGATCGTCCTAGTTAT
GATGGATATAATTGGAGAAAATACGGACAAAAGAAAGTTAAAGGAAGCGAATACCCAAGAAGTTACTACAA
GTGCACACATCTGAAGTGTCCTGTGAAAAAGAAGGTTGAAAGATCATATGACGGCCAGATAACTGAAATTGT
TTACAGGGGTGAGCACAACCACCCAAAGCCTCAGCCTCCAAAGCGCAACTTGTCAGATGGCCATAGGCAAAC
AGCCATATGCAATGACACTTCTAAAGAAACAAATAACCCTGCATGGGGTAACCAACATCCTCAGATGAGTGA
AGCTTACGTCTGTAGGATAGAAAATCAGAATGATCGCGGGTTAACTATACATTCCAGCAAAGTACCATGCTTT
TATGATCCCATTGTAGCTGCAGGAATGCACACTGCAGTCAGAAATTCTGAAGATTCTGCTGTAGGAAGTAAA
AAATTGAAGGCTACTTGTGATGAACAAAAAAGTAAAAGAAGGAAAATTAAAGGTCCATCCAGTGGAGCAGG
TACATCAGGGGAAAGTACATTTCCTTATATACCAAACCAAAGTACTACTGACTCGGAAATTACCGAGGACGG
TTTTCGCTGGAGAAAATATGGCCATAAGGTTGTGAAGGGAAGTTCATATCCCAGGAGCTATTACAGATGCAC
AAGTCCTAAATGCAGTGTGCGGAAGTTTGTTGAAAGAACCACGGATGATCCCAGAGCCTTTATTACTACATAC

GAGGGAAAACACAACCATGGTGTTCCAAACAGAAGACCAAATTCAGAGGCATCCAAAACAAGCTCAAAATC
TTCAGCTATGAAAGAGAAATTATAG

CaWRKY52

>Capana10g001805 [mRNA] locus=Chr10:185896489-185897886
ATGGAAGTCAATGAAGCAGCGAAATTACCTATAGCTAGACCAGTCGCTTCAAGGCCAAGATGTCCTCTTTAC
AAATCTTTCTCAGAGCTCCTGACTGGTGCAGTAGATATATCATCGACAAATGTTCATTCTGAAATGGCTATTA
CAGCCATAAGACCAAAGACTATCAGGTTGAAGCCTGCAACAAACCATGCTTTAGTTGGAGAGCCTTCTTCAC
AGGTTGGTGTGTCCGAGGCACCAGTTGGTTTTGGCGCTGATAACATCTTGCAATCGGTAGAGAAACCCAAGGT
CCTGTATAAGCCTATAGCTAAACTTGCACCAAAGAAAACAATTCCTCTACTTGAAAATAAGGGAAGCTCCGT
ATCTGATCAGCAACGAGTAATAGCTGATGCTGAGGCTCATATTCAATCAGCAAATGAAGTTAAACAACAACA
TGACCCTACGACAGAATCTAAACAAAGTCTCTCGGCAAAATCAGGACAGGACAAAAAAAAAGTGCGCTCAA
CAATTGTATCAGGGAGCACAGAGGAGGTTGCACAATCTTTGATCAACACAAGTAATGTCGATCGTCCTAGTTA
TGATGGATATAATTGGAGAAAATATGGACAAAAGCAAGTTAAAGGAAGCGAATACCCAAGAAGTTACTACA
AGTGCACACATCTGAAGTGTCTTGTGAAAAAGAAGGTTGAAAGATCATATGATGGCCAGATAACTGAAATTG
TTTACAGGGGTGAGCACAACCACCCAAAGCCTCAGCCTCCAAAGCGCAACTTGTCAGATGGCCATAGGCGAA
CAGCCATATGCAATGACACTTCTAAAGAAACAAATAACCCTGCATGGAGTAACCAACATCCTCAGATGAGTG
AAGCTTACGTCTGTAGGAGAGAAAATCAGAATGATGGCGGGTTAACTATACATTCCAGCAAAGTACCATGCT
TTTATGATCCCATTGTAGCAGCCGGAATGCACACTGCAGTCAGAACACTGCAGTCAGAAATTCTGAAGATTCT
GCTGTAG
>CA10g14770
ATGGAAGTCAATGAAGCAGCGAAATTACCTATAGCTAGACCAGTCGCTTCAAGGCCAAGATGTCCTCTTTAC
AAATCTTTCTCAGAGCTCCTGACTGGTGCAGTAGATATATCATCGACAAATGTTCATTCTGAAATGGCTATTA
CAACCATAAGACCAAAGACTATCAGGTTGAAGCCTGCAACAAACCATGCTTTAGTTGGAGAGCCTTCTTCAC

AGGTTGGTGTGTCCGAGGCACCAGTTGGTTTTGGCGCTGATAACATCTTGCAATCGGTAAAGAAACCCAAGGT
CCTGTATAAGCCTATAGCTAAACTTGCACCAAAGAAAACAATTCCTCTACTTGAAAATAAGGGAAGCTCCGT
ATCTGATCAGCAACGAGTAATAGCTGATGCTGAGGCTCATATTCAATCAGCAAATGAAGTTAAACAACAACA
TGACCCTACGACAGAATCTAAACAAAGTCTCTCGGCAAAATCAGGACAGGACAAAAAAAAAGTGCGCTCAA
CAATTGTATCAGGGAGCACAGAGGAGGTTGCACAATCTTTGATCAACACAAGTAATGTCGATCGTCCTAGTTA
TGATGGATATAATTGGAGAAAATATGGACAAAAGCAAGTTAAAGGAAGCGAATACCCAAGAAGTTACTACA
AGTGCACACATCTGAAGTGTCTTGTGAAAAAGAAGGTTGAAAGATCATATGATGGCCAGATAACTGAAATTG
TTTACAGGGGTGAGCACAACCACCCAAAGCCTCAGCCTCCAAAGCGCAACTTGTCAGATGGCCATAGGCGAA
CAGCCATATGCAATGACACTTCTAAAGAAACAAATAACCCTGCATGGAGTAACCAACATCCTCAGATGAGTG
AAGCTTACGTCTGTAGGAGAGAAAATCAGAATGATGGCGGGTTAACTATACATTCCAGCAAAGTACCATGCT
TTTATGATCCCATTGTAGCAGCAGGAATGCACACTGCAGTCAGAAATTCTGAAGATTCTGCTGAAGGAAGTA
AAAAATTGAAGGCTACTTGTGATGAACAAAAAAGTAAAAGAAGGAAAATTAAATGTCCATCCAGTGGAGCA
GGTACATCAGGGGAAAGTACATTTCCTTATATACCAAACCAAAGTACTACTGACTCTGAAATTACCGAGGAC
GGTTTTCGCTGGAGAAAATATGGCCAGAAGGTTGTGAAGGGAAGTTCATATCCCAGGAGATATTACAGATGC
ATAAGTCCTAAATGCAATGTGTGGAAGTTTGTTGAAAGAACCACGGATGATCCCAGAGCCTTTATTACTACAT
ACGAGGGAAAACACAACCATGGTGTTCCAAACAGAAGACCAAATTCAGAGGCATCCAAAACAAGCTCAAAA
TCTTCAGCTATGAAAGAGAAATTATAG

CaWRKY53

>Capana11g001882 [mRNA] locus=Chr11:200570029-200575124
ATGGCGGAGAATGAAGGATTATCATCTTCATCAGCGACATCAAGAGGACAATTAGTACGTCCAACAATTACT
TTACCACCAAGAAATTCAATGGACAGTTTATTTTCCGGTGGTATTAGCCCGGGTCCGATGACACTCGTATCCA
GTTTCTTCTCCGATAATGATCCTGATTCTGAGTGTCGGTCTTTTTCTCAGCTTTTAGCCGGTGCAATGACATCTC
CGGCGGGTATTTCCGGTGTTAGACCGGGTTTCTCGCCACCATCTACGGCGGCACCAGCGATGACTCCCACTTT

TAGTGTACCGCCTGGATTAAATCCAACTAATTTGTTTGATGGGTTCTTTTCACCTGGACAGGGCCCTTATGGAA
TGTCTCATCAGCAAGTGCTTGCTCAACTTACAGCTCAGGCATCCCAGCCTCAGTCGCAAATGCACATTCAGCC
TGATTACTCATCTTCTTCAGCAGCAACTGCACTGTCAATGTCACCATTCCAATCCTTAACATCAAATACAGCA
GCAAACCAACAGATACCTCCTGCATTGGATCCTAACATAATGAAAGAGTCTTCTGATGTTTCCCTGTCCGACC
AGAGGTCTGAACCTGCTTCCTTTGTTGTTGATAAACCTGCTGATGATGGCTACAACTGGCGGAAGTATGGGCA
GAAGCAGGTCAAGGGAAGCGAATATCCTCGTAGCTATTACAAGTGTACGCAGCCAAATTGTCCAGTCAAGAA
GAAGGTTGAGCGCTCCCTAGATGGACAGGTGACTGAGATTATATATAAGGGCCAACACAATCATCAGCCACC
TCAAGCTAGTAAGCGTTCAAAAGAAAGTGGAAATCCAAATGGAAACTATAATCTTCAGGGGACCTATGAGCC
CAAGGAGGGCGAACCTTCTTATTCCTTAAGAATGAAGGATCAAGAATCTAGCCTAGCAAATGACCAAATCTC
CGGCTCAAGTGACAGTGAGGAAGTAGGTAATGCAGAGACCAGAGTGGATGGGAGGGACATTGACGAACGAG
AATCAAAGCGGAGGGCGGTAGAAGTACAAACTTCTGAGGCCGTTTGTTCTCACCGGACTGTTGCAGAACCTA
GGATCATTGTTCAAACAACCAGTGAAGTTGATCTGTTGGACGATGGTTATAGATGGCGTAAGTATGGCCAGA
AGGTTGTTAAAGGAAACCCTTATCCAAGAAGCTATTACAAATGTACCAGCCAGGGATGTAACGTAAGAAAGC
ATGTCGAAAGGGCTGCAAGTGACCCTAAAGCAGTCATAACAACTTATGAAGGAAAACATAATCATGATGTGC
CTGCAGCCAGGAATAGTAGCCACAATACAGCCAACAATTCCACGTCACAATTGAGGCCACACAACCCTGTAT
TCGATAAACCAACTGCAATGCGAAGATCAGACTTTCCAAGCAATGAACAACAACCTATAGCACTTCTACGTTT
CAAAGAAGAACAAATTACATGA
>CA11g03750
ATGGCGGAGAATGAAGGATTATCATCTTCATCAGCGACATCAAGAGGACAATTAGTACGTCCAACAATTACT
TTACCACCAAGAAATTCAATGGACAGTTTATTTTCCGGTGGTATTAGCCCGGGTCCGATGACACTCGTATCCA
GTTTCTTCTCCGATAATGATCCTGATTCTGAGTGTCGGTCTTTTTCTCAGCTTTTAGCCGGTGCAATGACATCTC
CGGCGGGTATTTCCGCTTGTGACCATGTCAAAGACAATTTAGGGTGCTACAGCTTTTACGGTCTTAGGTTTGC
AAATCCTTTCAACGTATGCCATTTAAACAGATATAAATTAAGATTATCCTGCACTTATCACTTCTCTGATATTT
ATAATTCTGATTTATGTATCTTCATCTGTCAGGGCCCTTTTGGAATGTCTCATCAGCAAGTGCTTGCTCAACTT
ACAGCTCAGGCATCCCAGCCTCAGTCGCAAATGCACATTCAGCCTGATTACTCATCTTCTTCAGCAGCAACTG

CACTGTCAATGTCACCATTCCAATCCTTAACATCAAATACAGCAGCAAACCAACAGATACCTCCTGCATTGGA
TCCTAACACAATAAAAGAGTCTTCTGATGTTTCCCTGTCCGACCAGAGGTCTGAACCTGCTTCCTTTGTTGTTG
ATAAACCTGCTGATGATGGCTACAACTGGCGGAAGTATGGGCAGAAGCAGGTCAAGGGAAGCGAATATCCTC
GTAGCTATTACAAGTGCACGCAGCCAAATTGTCCAGTCAAGAAGAAGGTTGAGCGCTCCCTAGATGGACAGG
TGACTGAGATTATATATAAGGGCCAACACAACCATCAGCCACCTCAAGCTAGTAAGCGTTCAAAAGAAAGTG
GAAATCCAAATGGAAACTATAATCTTCAGGGGACCTATGAGCCCAAGGAGGGCGAACCTTCTTATTCCTTAA
GAATGAAGGATCAAGAATCTAGCCTAGCAAATGACCAAATCTCCGGCTCAAGTGACAGTGAGGAAGTAGGTA
ATGCAGAGACCAGAGTGGATGGAGGGACATTGACGAACGAGAATCAAAGCGGAGGGCGGTAGAAGTACAA
ACTTCTGAGGCCGTTTGTTCTCACCGGACTGTTGCAGAACCTAGGATCATTGTTCAAACAACCAGTGAAGTTG
ATCTGTTGGACGATGGTTATAGATGGCGTAAGTATGGCCAGAAGGTTGTTAAAGGAAACCCTTATCCAAGAA
GCTATTACAAATGTACCAGCCAGGGATGTAACGTAAGAAAGCATGTCGAAAGGGCTGCAAGTGACCCTAAAG
CAGTCATAACAACTTATGAAGGAAAACATAATCATGATGTGCCTGCAGCCAGGAATAGTAGCCACAATACAG
CCAACAATTCCACGTCACAATTGAGGCCACACAACCCTGTATTCGATAAACCAACTGCAATGCGAAGATCAG
ACTTTCCAAGCAATGAACAACAACCTATAGCACTTCTACGTTTCAAAGAAGAACAAATTACATGA

CaWRKY54

>Capana11g001905 [mRNA] locus=Chr11:201981442-201987021
ATGGATGAAAAAGATAAGGTTGATGATCAATTACCTATTGAATCGAGCTGGTCACAGCTCAATCCTGATGAT
GATGCAGATCATGCTTACTTCTTCGAAAATAATATTAATACAAATAACGATACCAGTAGTATACTGAGTGAAT
TCGGTTGGAATTTTCAACCAGTTGAAGAGAACAGTAGTAGTAGATTTGACAAGATCGATGAGCATTTGGCGG
GAAATAGTAGTATTACTACTATGAGTACGTCTCCAGCTTCTGCTACTGCTGCGACTGAACTGACAACGGCGAA
AATTAGTACCGATGAACCTGTTTCTTCCAGCTGTTCTGATGATCCGCCGGAAAAATCTACTGCTTCCGGTGGCT
CCTCCGCCTCTAAACCGCCGTCAGATACAGTAAGCAAGGTTAAAAAGAAGGGTCAGAAACGAATCAGGCAGC
CTCGATTTGCATTTATGACAAAAAGTGAAGTTGATCATCTTGAAGATGGCTATAGATGGAGAAAATATGGCC

AAAAAGCTGTTAAAAATAGTCCATTTCCAAGGAGTTACTATCGTTGTACAAATACAAAGTGCACAGTAAAGA
AGAGAGTGGAACGATCCTCTGAAGATTCCTCAATTGTAATCACGACATATGAAGGACAGCATTGTCATCATA
CAGTTGGATTTCCTAGAGGTGGACTTATCAATCATGAAGCTGCATTTACATCTCAATTATCACCTTTACCCTCA
CAATACTATCATCCCTCCGGTGTTCAGTACCCGCATGAATTAGTTCCTATGACTGCGGCTGCACCAGTAGAAT
CGCGTACAATGCCAGGTGAAACTGGATCATCAGAAGCTCGTAGATTGCCAGAAACAAGTCAACCTGCTGCAA
CTGATGAGGGATTGCTTGGAGATATTGTACCTCCTGGGATGCGAAGCAAATAA
>CA10g13480
ATGACAAAAAGTGAAGTTGATCATCTTGAAGATGGCTATAGATGGAGAAAATATGGCCAAAAAGCTGTTAAA
AATAGTCCATTTCCAAGGAGTTACTATCGTTGTACAAATACAAAGTGCACAGTAAAGAAGAGAGTGGAACGA
TCCTCTGAAGATTCCTCAATTGTAATCACGACATATGAAGGACAGCATTGTCATCATACAGTTGGATTTCCTA
GAGGTGGACTTATCAATCATGAAGCTGCATTTACATCTCAATTATCACCTTTACCCTCACAATACTATCATCCC
TCCGGTGTTCAGTACCCGCATGAATTAGTTCCTATGACTGCGGCTGCACCAGTAGAATCGCGTACAATGCCAG
GTGAAACTGGATCATCAGAAGCTCGTAGATTGCCAGAAACAAGTCAACCTGCTGCAACTGATGAGGGATTGC
TTGGAGATATTGTACCTCCTGGGATGCGAAGCAAATAA

CaWRKY55

>Capana12g001134 [mRNA] locus=Chr12:48289811-48291121
ATGTTGGAAGAGGAATTGAAGAGGATGAAAGAGGAGAACAAGAAGCTAGCAACTATGCTAACAACTTTGGG
TGAAAACTACAATTCCTTGAGAACTAATCTAATTGAGTTGCAGCAAAAACATTCCACTCATGAAGAGGACAA
TAATTCTAAATTATTGTCAAGGAAAAGAAAGGCTGAAGATGTATGTTGTGTAAATAATTCAGACATCAACTTT
GAAGAAGCATCACCAAAGAGGCCAAGGGAAATCATCACAACCAGTATTTCAACTGTTAGTGTTAAAACCACT
CTCTCTGATCAAACCTCATGGGTAAAAGATGGATATAACTGGAGAAAATATGGTCAAAAGGTGACAAGAGAT
AACCCTTCTCCAAGAGCCTACTACAAGTGTTCATTTGCACCAACATGCCCAGTCAAGAAGAAGGTACAAAGA
AGTGTTAAAGATCCATCAGTTTTAGTAGCTACATATGAAGGGGAGCACAACCACCCCCACCCATCCCAAGCT

GAAACAACAGCGCCATTAGTTAACCAAGGTGTTATAACAAATCCAACATTTTTTAACAAATTCATGGAAGAC
ATCAACACAAGTTCACTGCAGAAAGATTTAGTCGCTAAAATGGTGCCTTCCTTGTCGAAGAACCCTAGTTTTG
CTGCTACAGTTGCTCAGCCATCTCTGGAATATTTTCTCGAATATGATTTGCAGTTACTCAAATGGGTGTTGAGG
GGGTAA>CA00g00230ATGTTGGAAGAGGAATTGAAGAGGATGAAAGAGGAGAACAAGAAGCTAGCAACTAT
GCTAACAACTTTGGGTGAAAACTACAATTCCTTGAGAACTAATCTAATTGAGTTGCAGCAAAAACATTCCACT
CATGAAGAGGACAATAATTCTAAATTATTGTCAAGGAAAAGAAAGGCTGAAGATGTATGTTGTGTAAATAAT
TCAGACATCAACTTTGAAGAAGCATCACCAAAGAGGCCAAGGGAAATCATCACAACCAGTATTTCAACTGTT
AGTGTTAAAACCACTCTCTCTGATCAAACCTCATTGGTAAAAGATGGATATAACTGGAGAAAATATGGTCAA
AAGGTGACAAGAGATAACCCTTCTCCAAGAGCCTACTACAAGTGTTCATTTGCACCAACATGCCCAGTCAAG
AAGAAGGTACAAAGAAGTGTTAAAGATCCATCAGTTTTAGTAGCTACATATGAAGGGGAGCACAACCACCCC
CACCCATCCCAAGCTGAAACAACAGCGCCATTAGTTAACCAAGGTGTTATAACAAATCCAACATTTTTTAACA
AATTCATGGAAGACATCAACACAAGTTCACTGCAGAAAGATTTAGTCGCTAAAATGGTGCCTTCCTTGTCGAA
GAACCCTAGTTTTGCTGCTACAGTTGCTCAGCCATCTCTGGAATATTTTCTCGAATATGATTTGCAGTTACTCA
AATGGGTGTTGAGGGGGTAA

CaWRKY56

>Capana12g001826 [mRNA] locus=Chr12:170920299-70933933
ATGGAAAATTTTTCCTACAATTACTCAAACCCTAACCCTAATAATGGAGATATTTATAGCTCGAATTTTATCG
ATACACCGGAGAATTTTGAGCTCTCCAGTTATTATCTCTTCCCTGAAGATGGATTGAGTGATGAGTTTTTGTCA
CAAAATGAATTTGTTCAAAGTGCTTCCGATAGTAGAAGATCATATTCCAATATTAAACCCGCTCCAACAACTA
CTCATCATAACATGCAAGTAAAATGTACAAAAGGTGTAATGAAGAAGGTGGATGCAAAGTCTAGGGTTGCAT
TTAGATTTAGATCAGAGTTGGAGGTGTTGGATGATGGATATAAATGGAGGAAATATGGCAAGAAGATGGTCA
AGAATAGTCCAAATCCAAGGAATTACTACAAATGTTCAAATGGAGGATGCAATGTGAAAAAGAGAGTAGAA
AGGGACAATGAAGATTCAAGCTATGTCATTACTACCTATGAAGGAATTCACAACCATGAGAGTCCCCATGTG

ATTCACTACACACAGTTCCCTCCCAATAATATTGCCCTTCATAACCTTCACCTATAA
>CA12g09290
ATGTATGAATTCAGGAATTACTACAAATGTTCAAATGGAGGATGCAATGTGAAAAAGAGAGTAGAAAGGGA
CAATGAAGATTCAAGCTATGTCATTACTACCTATGAAGGAATTCACAACCATGAGAGTCCCCATGTGATTCAC
TACACACAGTTTCCTCCCAATAATATTGCCCTTCATAACCTTCACCTATAG

CaWRKY57

>Capana12g001851 [mRNA] locus=Chr12:173653320-173658337
ATGTTTAGCCAGAGCTTGCTTGAAGATCATCAAGATATGTCATCACAACTTGGATTTTTCTCTTTTCCTCCAAA
TTACAACAACGTGGGCATGATTAGTACTACTACTACTGCTACTCTACCATTTATTGGATACAACCAAAATACT
CTAAAGACCCTCACTATGAATATCCCTCCCTCTTTTGATCATTCCTTAAATATTCAAGAATCTACACATGATCC
AAGGCGCAAAGAGGACCTTAGTCCTATATTTGGGGGACCCCATCTTCATTCCTTGCAAAAATCCACTCCAAAT
ACATGGGCATGGGGAGAAGTGAATGAGAGCAGCAATATTATTAAGAGAAGGGAGTTTGATCATGATCATAAT
TTAGGGGTTTCATCAATCAAGATGAAGAAGATCAAATCATCAAGAAGGAAAGTAAGAGAACCAAGATTTTGT
TTCAAGACTATGAGTGATGTGGATGTGTTGGATGATGGTTATAAATGGAGAAAATATGGCCAGAAAGTTGTT
AAAAATACCCAACATCCCAGGAGCTATTATCGATGTACACAAGATAATTGTAGAGTTAAGAAACGAGTGGAG
AGATTAGCAGAAGATCCGAGAATGGTGATCACAACATATGAAGGGAGACATGTTCACTCTCCATCACATGAT
GAAGAGGATTCACAGGCTTCATCACAACTTAATAATCTCTTATGGTAG
>CA12g09140
ATGAAGAAGATCAAATCATCAAGAAGGAAAGTAAGAGAACCAAGATTTTGTTTCAAGACTATGAGTGATGTG
GATGTGTTGGATGATGGTTATAAATGGAGAAAATATGGCCAGAAAGTTGTTAAAAATACCCAACATCCCAGG
AGCTATTATCGATGTACACAAGATAATTGTAGAGTTAAGAAACGAGTGGAGAGATTAGCAGAAGATCCGAGA
ATGGTGATCACAACATATGAAGGGAGACATGTTCACTCTCCATCACATGATGAAGAGGATTCACAGGCTTCA
TCACAACTTAATAATCTCTTATGGTAG

CaWRKY58

>Capana12g001851 [mRNA] locus= Chr00:245677269..245678729
ATGGAGAATTACCCACCACTATTTCCATCATCATTATCGTCTCATGAGTTTTCATTAATGAATAAGAAGAGGA
GTAATACTCATGCAAAAGAGGTTTTATTATTCCAAGGAAAGAACAACGGGTTCTTGGGGCTAATGGCAAGCA
TGGAAACTCCGAGCGGTGTAACAAATAGTTTTGAGGACGATGTGATGAAATCGTGTAAGAAGAAGGGAGAG
AAGAAGATTAAGAAACCAAGGTACGCTTTCCAAACAAGGAGCCAAGTGGATATTCTTGATGACGGTTATAGA
TGGAGGAAATATGGACAGAAGGCTGTCAAGAACAACAAATTCCCAAGGTCAGTTATTAACTTAATTCGATCC
ATGTATAAATTAATTATGACTTTCCCAAATGGAAATTATCTTTTAGTTCAGCGTCTAGGTGTATTCCCAAATAA
AATTTTCTACGCCAACGAATCTACGTTCTAATCCTTGTCAAGATTCATTTAAGTTAGTTTTAGGGATTCTTTTTT
TACTTTTTTCTTTTTCTTTAAAAAGAGCCGTGGCCCGAAAATCAAATAGGTCTGCAACTTGATGCTTTACAAAG
GGAAAAATGATATATGTTTGACGGAGGTGTTCTACTTTCTGGCAATAACTGTCCAACTACTGTAATATATTAG
ATCTTCACGTGCATGCCTTAAGAGATCCAAATTTTTATTATTTGGTTTTGAATAAATATGATCTTATTATGTGC
ATTTATTTTCATTTGTATATATAGTTCGACCCGATAGAACCTGTGCTAGATTCGCTTCTCTACTTTCAATATTAC
TTGGTTTATGCTAGATAGTAAATTTGTTACAAAACGAAGGTGCAAGGAAATTTATTTAATTAGTCCTTTGATA
ACAAATAAGAGACGTAACGATCCTGAGAAACCGAGGAGTTTTGATGTGGATTTTGATTTTAAAAAATGGATG
GGTATATACTGGAAGTTTTGAAGTGAAAACATGAAATTGAAAAAATCAGTAGTTATTGGAAATGGAACAATA
AATTGGAACCTATATATATTACCGGCTACAAACAATGATCGGTGTCTCTAATCCATGAAATTGATCTGGCTGT
GGTCATCATAAGAAAAATTAAGTGATATCCCTCCCTGCATACTTTCCTCTTTTTAATTACTACTATTACTTCGG
TGGCGCTAATACTAGTGTCTTAGACATTGGAAAATGTTAGGTGGAATTAGTATTACTCCAATTTACTTCTACA
AAAATTAAGTGTATTGCCTAATAAATGTTTGTGGTGATTAGGAGCTACTACCGATGCACGCATCAAGGATGTA
ACGTGAAGAAACAAGTACAAAGGTTGTCCAAGGATGAAGGAGTAGTGGTAACTACTTATGAAGGCATGCATT
CACATCCCATTGACAAGTCTACCGATAACTTTGAGCAGATTTTGAGTCAGATGCAAGTCTATGCTTCCTTCTA
A

CaWRKY59

>Capana00g001033 [mRNA] locus=Chr00:331116291-331117751
ATGGAGAATTACCCACCACTATTTCCATCATCATTATCGTCTCATGAGTTTTCATTAATGAATAAGAAGAGGA
GTAATACTCATGCAAAAGAGGTTTTATTATTCCAAGGAAAGAACAACGGGTTCTTGGGGCTAATGGCAAGCA
TGGAAACTCCGAGCGGTGTAACAAATAGTTTTGAGGACGATGTGATGAAATCGTGTAAGAAGAAGGGAGAG
AAGAAGATTAAGAAACCAAGGTACGCTTTCCAAACAAGGAGCCAAGTGGATATTCTTGATGACGGTTATAGA
TGGAGGAAATATGGACAGAAGGCTGTCAAGAACAACAAATTCCCAAGGAGCTACTACCGATGCACGCATCA
AGGATGTAACGTGAAGAAACAAGTACAAAGGTTGTCCAAGGATGAAGGAGTAGTGGTAACTACTTATGAAG
GCATGCATTCACATCCCATTGACAAGTCTACCGATAACTTTGAGCAGATTTTGAGTCAGATGCAAGTCTATGC
TTCCTTCTAA
>CA11g12710
ATGAATAAGAAGAGGAGTAATACTCATGCAAAAGAGGTTTTATTATTCCAAGGAAAGAACAACGGGTTCTTG
GGGCTAATGGCAAGCATGGAAACTCCGAGCGGTGTAACAAATAGTTTTGAGGACGATGTGATGAAATCGTGT
AAGAAGAAGGGAGAGAAGAAGATTAAGAAACCAAGGTACGCTTTCCAAACAAGGAGCCAAGTGGATATTCT
TGATGACGGTTATAGATGGAGGAAATATGGACAGAAGGCTGTCAAGAACAACAAATTCCCAAGGAGCTACTA
CCGATGCACGCATCAAGGATGTAACGTGAAGAAACAAGTACAAAGGTTGTCCAAGGATGAAGGAGTAGTGG
TAACTACTTATGAAGGCATGCATTCACATCCCATTGACAAGTCTACCGATAACTTTGAGCAGATTTTGAGTCA
GATGCAAGTCTATGCTTCCTTCTAA

CaWRKY60

>Capana00g003083 [mRNA] locus=Chr00:523068129-523069793
ATGGCTTTAGATTTGTTTGCGATAGAACAAACTGCTTCAGCTGGTTTAAAATCTATGGACCATTTAATCCAATT
TGTTTCATCGAACCCTACAGCTAAACCGGATTGTAGAGAGATAACTGAGTATACTGTTTCGAATTTTCGGAAT

GTTATCTCTATGCTTAACCGGCCTACCTGCCATGCCCGGGTCAGACGTGTTGGTCCGGTTCAGCCGGTTAAGG
TTGCTCCACCGGTGGTTTCTTCGCCTGTAGTTGCTCCACCGATGGTTGCTGCTCCCGTGGAGAAGGAAAAGGA
GAAAGAGAAAATGTTTAGGTCAACGCCAGCGTTGACTTTTGACTTTACCAAGCGTAAGGTTGCTGTTCCTGCT
GCCCCTTCTCCTGCTGCTGGTGTTGGTGTTGTTTCGAAGGATGTGGCGATGGCAAATTCGACGAATTCGTCGTC
GTCGTCGTTTGTGTCGACGATTACTGCTGAGGGAAGTGTATCGAATGGGAGGGTATTCCCGTCAATGGATTTG
CCTCCGCGCCCGCCGGTGACGGCGCCGGCTGCTTTCTCCGGGAAGCCTCCGATTGCAGGAAAAAGGTGTCGC
GATCATGATGTGTCTGATGAGTTTTCCGGCAGAACTTCTAGCGCCGGCAAGTGCCCCTGCAAAAAGAGCAAA
CCAAAGGTGAAGAAAGTGATCAGAGTACCGGCGATAAGCTCAAAGACTTCTGATATACCGGCAGATGAATTC
ACATGGAGGAAGTACGGTCAAAAGCCGATCAAAGGTTCACCATACCCAAGGGGTTACTACAGATGCAGCAGC
TTGAAAGGATGCCCGGCTAGAAAGCACGTTGAGAGGGCAACTGATGATCCAAGAATGCTGATTGTAACTTAT
GAAAATGATCATGAACATCATCATAACATTCAAACTGCATTTTCTGGTGCTGCCATTGGATCCAGAGATGGAA
GTTCTGGCCAGAGAATGATGGTGTTTGAGTCAATGGGACAAAAGTGA
>CA01g29700
ATGGCTTTAGATTTGTTTGCGATAGAACAAACTGCTTCAGCTGGTTTAAAATCTATGGACCATTTAATCCAATT
TGTTTCATCGAACCCTACAGCTAAACCGGATTGTAGAGAGATAACTGAGTATACTGTTTCGAATTTTCGGAAT
GTTATCTCTATGCTGAACCGGCCTACCGGTCATGCCCGGGTCAGACGTGGTGGNNCGGTTAAGGTTGCTCCAC
CGGTGGTTTCTTCGCCTGTAGTTGCTCCACCGATGGTTGCTGCTCCCGTGGAGAAGGAAAAGGAGAAAGAGA
AAATGTTTAGGTCAACGCCAGCGTTGACTTTTGACTTTACCAAGCGTAAGGTTGCTGTTCCTGCTGCCCCTTCT
CCTGCTGCTGGTGTTGGTGTTGTTTCGAAGGATGTGGCGATGGCAAATTCGACGAATTCGTCGTCGTCGTCGT
TTGTGTCGACGATTACTGCTGAGGGAAGTGTATCGAATGGGAGGCCTCCGATTGCAGGAAAAAGGTGTCGCG
ATCATGATGTGTCTGATGAGTTTTCCGGCAGAACTTCTAGCGCCGGCAAGTGCCCCTGCAAAAAGAGCAAAC
CAAAGGTGAAGAAAGTGATCAGAGTACCGGCGATAAGCTCAAAGACTTCTGATATACCGGCAGATGAATTCA
CATGGAGGAAGTACGGTCAAAAGCCGATCAAAGGTTCACCATACCCAAGGGGTTACTACAGATGCAGCAGCT
TGAAAGGATGCCCGGCTAGAAAGCACGTTGAGAGGGCAACTGATGATCCAAGAATGCTGATTGTAACTTATG
AAAATGATCATGAACATCATCATAACATTCAAACTGCATTTTCTGGTGCTGCCATTGGATCCAGAGATGGAAG

TTCTGGCCAGAGAATGATGGTGTTTGAGTCAATGGGACAAAAGTGA

CaWRKY61

>Capana00g004057 [mRNA] locus=Chr00:607660271-607664416
ATGGGTACCCCTAAAGAAGAGACAACAGATGAAGTTTTCTCCGAGAATTTAGAGCAAAAGCCGGAGCCTGAT
CCTGCAACCAAATCAGAGTTAAAAGAGAAACGGAGCTTTGAATCGACTTCAGCTGATGTTGTTTCTGGTGAGC
TGCAGAAGAGATTGAGTCCTGATGCTGACAAACAAGCATCAAAGAATAATGAAGAGGAAAGTACATATCCTC
CAACAGGCCAAGAAGTGTCAAGGATTAGTCAATGTGATAAAGGGATTAATGTGTCACAATCCAATCAAGAAG
ATATTTCTCTTTCTAGAGTACTAGAGAACCCGTCCGAAAATGTGGGGCAGCTGCAGGTTCTTAAGAGTGAAGC
CGGTGCATCTGGATCTAGTCAACTTTCTAGTTTACCGAAGGACTCAGATGCAAAATCATGTGGATCAGAATCT
GGTGTAAAACGTTTATCTGGTAAGGCTTCAGATTCTTCAGATCAAATGCAAAGTTCGAACACGGAGATTTTAT
TATCACAATCTGATCAACAACGAGTAAATTATCCCATACAAAAGCGTGAGAAAGCCCTAGATAAGTTGCAAC
CAAGGCGGAACCCTGACACTAGTGTCCATGGGTTGACGTCTGATCAAGGAGTGACTCTCCTCAGGGTGCCTG
AAAAACCATCCGAAGATGGATATAACTGGCGAAAGTATGGTCAGAAGCTTGTCAGAGGAAATGAGTATACTC
GGAGTTATTACAAGTGCACATACCCTAATTGTCAAGCAAAAAAGCAAGTGGAGAGATCACATGACGGGCATA
TTACAGATATCCACTATATTGGGAAGCATGAACATCCGGAAACTCCAAGTGGTCCTCAGATGCTCCCCGAGTT
GGTACTCCCTTTGCAAATGAAACAACCAGAGATTCCAATAATCTCTACATTAGAAGCTGAAGGCGAGAAATC
TACTAGGCCCCAAGAAACATGTGAACCTAGTAAGCCATCAGAAGCTCCGCTTGTATTGGACATTGTATCAGCT
TGTGGCGGTGTGAAGGGTACGCCTTTAAAGCGACATAAATCAGAAACTGAGGTCGATAAGGATGATGGATCA
GACTCAAAAAGACAAAAAAAGGATATAGTAGCTACGGTTGATACTCCACCTATTAAGTCCCAAAGTGAACCA
CGACACATTGTTCAGACCGTGAGCGAAGTAGATATAATCAATGATGGTCAGCGCTGGCGCAAATATGGGCAA
AAAATTGTAAAAGGCAATCCAAATCCGAGGAGTTACTACAGATGCTCAGTTGCTGGTTGCCCCGTGAAGAAG
CATGTGGAGAGGGCATCCCATGATCCAAAAGTGGTCATTACAACATATGAAGGGCAGCATGTCCATAATTTC
CCAACTCCTAGGGATATAAGCCAAATCTCACCAGTGCCTGATGTCGTTACAACAGCCATACGTACAGATTCCA

GAATTGAATCAGGTCACAAACATGTCGTAGAGTCCAAATCTGAATCAGGTGAAAGAAAACATGTTGGAGATT
CCAGAACTGAATTGGGTGAAAACAAACATATTGAAAAGTCCAAACCTGAATTGGGTGGAAACAAACATGTTG
GAGACTCCAAATCTGAATCAGGTGAAAGCAGACATATCGGTAAGTCCAAAATTGAATCAGGTGAAAACAAAC
ATGTTGGAGGGTCCAGAATTGAATCGGGTGGAAACAAACATGTTGAAGAGTCTAAGCTTGAATTGGGTGGAA
ACAAACATGTCGGAGAGTCCATACCTGAATCTGCTGAAAACAAACATGTTGGTCTTGACATGGCTGTTCATAT
TGGTGCAAATTGA
>CA09g14010
ATGGGTACCCCTAAAGAAGAGACAACAGATGAAGTTTTCTCCGAGAATTTAGAGCAAAAGCCGGAGCCTGAT
CCTGCAACCAAATCAGAGTTAAAAGAGAAACGGAGCTTTGAATCGACTTCAGCTGATGTTGTTTCTGGTGAGC
TGCAGAAGAGATTGAGTCCTGATGCTGACAAACAAGCATCAAAGAATAATGAAGAGGAAAGTACATATCCTC
CAACAGGCCAAGAAGTGTCAAGGATTAGTCAATGTGATAAAGGGATTAATGTGTCACAATCCAATCAAGAAG
ATATTTCTCTTTCTAGAGTACTAGAGAACCCGTCCGAAAATGTGGGGCAGCTGCAGGTTCTTAAGAGTGAAGC
CGGTGCATCTGGATCTAGTCAACTTTCTAGTTTACCGAAGGACTCAGATGCAAAATCATGTGGATCAGAATCT
GGTGTAAAACGTTTATCTGGTAAGGCTTCAGATTCTTCAGATCAAATGCAAAGTTCGAACACGGAGATTTTAT
TATCACAATCTGATCAACAACGAGTAAATTATCCCATACAAAAGCGTGAGAAAGCCCTAGATAAGTTGCAAC
CAAGGCGGAACCCTGACACTAGTGTCCATGGGTTGACGTCTGATCAAGGAGTGACTCTCCTCAGGGTGCCTG
AAAAACCATCCGAAGATGGATATAACTGGCGAAAGTATGGTCAGAAGCTTGTCAGAGGAAATGAGTATACTC
GGAGTTATTACAAGTGCACATACCCTAATTGTCAAGCAAAAAAGCAAGTGGAGAGATCACATGACGGGCATA
TTACAGATATCCACTATATTGGGAAGCATGAACATCCGGAAACTCCAAGTGGTCCTCAGATGCTCCCCGAGTT
GGTACTCCCTTTGCAAATGAAACAACCAGAGATTCCAATAATCTCTACATTAGAAGCTGAAGGCGAGAAATC
TACTAGGCCCCAAGAAACATGTGAACCTAGTAAGCCATCAGAAGCTCCGCTTGTATTGGACATTGTATCAGCT
TGTGGCGGTGTGAAGGGTACGCCTTTAAAGCGACATAAATCAGAAACTGAGGTCGATAAGGATGATGGATCA
GACTCAAAAAGACAGTATGTTGAAACCCCAAGCTGTGTTTGTCTTTGGTGTTTCAGGTCTAGACTCTATTTTAA
ATTAATAGTTGTGTTATTATCTTGCAACAGAAAAAAGGATATAGTAGCTACGGTTGATACTCCACCTATTAAG
TCCCAAAGTGAACCACGACACATTGTTCAGACCGTGAGCGAAGTAGATATAATCAATGATGGTCAGCGCTGG

CGCAAATATGGGCAAAAAATTGTAAAAGGCAATCCAAATCCGAGGAGTTACTACAGATGCTCAGTTGCTGGT
TGCCCCGTGAAGAAGCATGTGGAGAGGGCATCCCATGATCCAAAAGTGGTCATTACAACATATGAAGGGCAG
CATGTCCATAATTTCCCAACTCCTAGGGATATAAGCCAAATCTCACCAGTGCCTGATGTCGTTACAACAGCCA
TACGTACAGATTCCAGAATTGAATCAGGTCACAAACATGTCGTAGAGTCCAAATCTGAATCAGGTGAAAGAA
AACATGTTGGAGATTCCAGAACTGAATTGGGTGAAAACAAACATATTGAAAAGTCCAAACCTGAATTGGGTG
GAAACAAACATGTTGGAGACTCCAAATCTGAATCAGGTGAAAGCAGACATATCGGTAAGTCCAAAATTGAAT
CAGGTGAAAACAAACATGTTGGAGGGTCCAGAATTGAATCGGGTGGAAACAAACATGTTGAAGAGTCTAAG
CTTGAATTGGGTGGAAACAAACATGTCGGAGAGTCCATACCTGAATCTGCTGAAAACAAACATGTTGGTCTT
GACATGGCTGTTCATATTGGTGCAAATTGA

CaWRKY62

> Capana00g004112 [mRNA] locus= Chr00:612680042..612682368
ATGTTTGGCTCTTCAACTTTCCAAGAAACAAGCAATGTCACTTCTCATCATTATCAAACTATAAACCCTAATTT
TGCTTTTCATGATCCACTCATCAACATGAATCAAGATCATGGTCATAACAATAATAAGTATCAAGATTTTGAT
ACTTCATTTCTTGATATGTTGCTTGATGGTGGTGATCAAGAATATTATTCCAATTACTTAAATAATAGCTACAA
TAATAATGTTTCATTTTATTCAGAAAATCCTTTCACGCAGCAAGAGATAAGCAGTAGTACCTATAGTACTAGT
GGAAACTCATCAACAGCTAGTTCATTTGATGCCACACTAACAAATATTCACATGAATCATGAGTAAGTCACTA
AATCAAACTACGTAAGTAGAGGTGGAGTCAAGATTTTAAATTTATGATTCTGCCTTCTAGAAAAGACACATGA
ATTACTGAATTCTGACTAAATTATATATCTATATATATATTAAGTGGCTTTTTTTAACATAAATATAAGGTCTA
GGCCAAAGCTACGAATAATCAGTATTATTGATTGTTGACTTATTTTAATGTTTATTATTTATTTACTTGTGTTTG
TTTGATTTTGGTTATTTTATAGAAACTCAAGCATGGGGATAGAAAAAGAAAAGAAGGGTGAAAAGCATGCAA
TTGCTTTTAGAACAAAGACAGAGCTTGAGATCTTGGATGATGGATACAAATGGAGGAAATATGGGAAAAAGA
AGGTCAAAAGCAACACAAATCTAAGGTAGTTAATTAAAATTCTTATATTATATCATATTACTATTATAATTTG
CTTTTGAAGGTTGATTATATTATTTCAGAAATATTCTTGAATAAATTGTACTCATTAAATATAGTAACAAATTA

AAGCCAATTTTGGCTGCTAGAAGTCTTTAAGATATGTGTTTGCTTAATAAACTAGAATCGCAAAGATTAGAAT
TGAATTCTTTTACAAGTACGTAGATTGATATACATAAACAAAATAGAAAGTTCTCCTATTATGTGTCACAAAG
ATATGGACGATATTGGTGACTCACTTAGATTCTTGATTATCTTTTTCATAACCCAACCCCACCATAGTGACCCT
ACCCCCAACCCTGCATCCCAAAACACACACATTATGAACGGCACCAGTGGTAATGCTACCTTTGTTTATGGAG
AGTCAATTGACACTTTTTGTCCGAAAAATTATATAGTATAAAGATATTAAAGAAGAAAAAAAATCGTCAATT
GATATCTTAATTTGTACGACATTTTTCTTTATAGTGCATTCCAAAAAAAAAAAAAAAAAAATATTTTGTCCAATGT
GAGACATAAATAAAGCTTTAAAGGAGAGATAGAGAGAAGACCTACTTGTTATTAAAATATGTATATTTTTTG
AAGACTAAGATCAGTAATTCTACTATTGGTTCATTCATTATATTCTTGTCGGTACCCATCGAATTTATTCCAAA
GTATATTATTTACTGTATAAAATTTTAGATTCTTAATTCTTTTGACCTACTTTACATACAATCTTATATTTTATT
GGTCTTATCTAAAATTAATATTTATGAGAAAAAAACTTACAATCTCTAAAAACTCATGCAAATTTAACGAAAT
AGAGGGCATACACACACACACACACACACAAAAAAAAAAAAAAAAAAAAAAAAAAAAAAAAAAAAAAA
AAAAAAAATATATTTTTTTTNNNNNNNNNNNNNNNNNNNNNNNNNNNNNNNNNNNNNNNN
NNNNNNNNNNNNNNNNNNNNNNNNNAAAAAAAAAAAAAAAAAAAAAAAAAAAAAAAAAAAAAAAAACAGAAAA
TAATTGAGATTATTTGTTCTAGTCATGTTAATTTATTTACATTAAATTTATTAATATTTGTTAGGATTCCTTAAG
TTCTACCAGAGATTTACATGCTAGCAAAAAGCGAAAAAACTTTTGATGCTTGAAAAATTAATAGTAATTTTGT
AATATTGAATAGAGACGGACTTATATTAGACACACCAAACATCCAGGTTCATTAACTGACCCAAGTCAATAG
AGGATTCATGTTCAAGTATTGTTTAATTTCTTTCTTTTATCTTTTTTTCCGAAAAAGATATTACTGAAAATTCAG
AAATAGTAGTGATTTCTTTACTCGTGGGGGAAATTAAACAGGAATTACTACAAGTGTTCAAGTGGAGATTGCA
AAGTAAAGAAGAGAGTAGAAAGAGATGGAAATGATTCAAGCTATTTGATAACTACGTATGAAGGACGACAC
AACCATGAAAGCCCCTTTGTCATTTATTGCCATGATGAAATGCCAACATCCAAGTATTTGAGATGA

Part Ⅱ The protein sequences of CaWRKY gene family in pepper genome database

CaWRKY1

>Capana01g000165 [mRNA] locus=Chr01:2380410-2384292
MQHYNSTIMDSGKEPPPPPPPPLLSTDLSPANFFMQQPNDQMPMQNNYHSHVGLDCDNIDWAGLLSAGPSINNESI
ATTSNVSINHRNMNTNMMEGGEQHQQLLPQQQQHEVCRRDKGRIIKKRKYVPPRIAFHTRSTEDILDDGFKWRK
YGQKAVKNSTHPRDVGETSMQELGYAGLKLTTDVNYLSR
>CA01g01900
MQNNYHSHVGLDCDNIDWAGLLSAGPSINNESIATTSNVSINHRNMNTNMMEGGEQHQQLLPQQQQHEVCRRD
KGRIIKKRKYVPPRIAFHTRSTEDILDDGFKWRKYGQKAVKNSTHPRSYYRCTHHTCNVKKQIQRHSKDTSIVVTT
YEGIHNHPCEKLMETLSPILKQLQFLSRF

CaWRKY2

>Capana01g000167 [mRNA] locus=Chr01:2417686-2419547
MDWGLQAVVIGSSSTSCSDIHGSNYVDFSPNLDFQESEYLSFSHHHHQHHEMKKEVYSDELEQLYKPFYHVGGQN
MLMGSSISLVPKEVIKEEKREEEQQQVVAASNTYVPKYKKRKNEQKRVVLQLKADDLSSDKWAWRKYGQKPIK
GSPYPRSYYRCSSSKGCLARKQVEQSCTEHGIFIVTYTAEHNHSQPTRRNSLAGTTKSKFPNSKNSINSPKKIVKEEK
ITSPHCSTSNLGLSPEAARMIDEFPEINQENNIIIGGDNNYYDEDGMRNIFEGSDENECVSIEEMFDGDFFAGLEDIHD
GFNSSFGCNNSAFPFSST
>CA01g01920
MIMDWGLQAVVIGSSSTSCSDIHGSNYVDFSPNLDFQESEYLSFSHHHHQHHEMKKEVYSDELEQLYKPFYHVGG

QNMLMGSSISLVPKEVIKEEKREEEQQQVVAASNTYVPKYKKRKNEQKRVVLQLKADDLSSDKWAWRKYGQKP
IKGSPYPRSYYRCSSSKGCLARKQVEQSCTEHGIFIVTYTAEHNHSQPTRRNSLAGTTKSKFPNSKNSINSPKKIVKE
EKITSPHCSTSNLGLSPEAARMIDEFPEINQENNIIIGGDNNYYDEDGMRNIFEGSDENECVSIEEMFDGDFFAGLEDI
HDGFNSSFGCNNSAFPFSST

CaWRKY3

>Capana01g002803 [mRNA] locus=Chr01:178651461-178653597
MYYLDNNFAVTNSHSFTGLISDYYGVEGRNIMNTSSSLGFMELLGFQDLMCSSSASFFELPKEENSCPAVCVSEEV
KPTAGESQNKLISTVAAANVFNTPSTPNCSSISSETNEGHTNTTHEDAEAGEVLDHHDQQHTNTKQQLKAKKTVSQ
KKQREPRFAFMTKSEVDFLEDGYRWRKYGQKAVKNSPFPRNYYRCTSATCNVKKRVERCFSDPSIVVTTYEGKVP
>CA01g22410
MYYLDNNFAVTNSHSFTGLISDYYGVEGRNIMNTSSSLGFMELLGFQDLMCSSSASFFELPKEENSCPAVCVSEEV
KPTAGESQNKLISTVAAANVFNTPSTPNCSSISSETNEGHTNTTHEDAEAGEVLDHQDQQHTNTKQQLKAKKTVSQ
KKQREPRFAFMTKSEVDFLEDGYRWRKYGQKAVKNSPFPRNYYRCTSATCNVKKRVERCFSDPSIVVTTYEGKH
THLSPMNTIMPRPSCYPITPVPASPGAFPLPMQFNINQSFNNLTSSLAMNNQLDHAAFVAQGRRFCTSEMLGDEGLL
QDLMPSTLIKENYR

CaWRKY4

>Capana01g003441 [mRNA] locus=Chr01:226400966-226405165
MERGGAERDHQLNNYNLQVSFSSSSVAANNIHELGFVHFADHNLSFLAPSSQSSQISQPLQAASVSVTPPTTINTNV
AAGGSNNVTGGLGFSHNELVINRSSWNSDQVETLDPKAV
NDENCGGNANEGNNSWWKTSSSDKGKVKIRRKLREPRFCFQTRSDIDVLDDGYKWRKYGQKVVKNSLHPRSYY
RCTHSNCRVKKRVERLSEDCRMVITTYEGRHNHSPCDDSNSSD

HDCFTSF
>CA01g28510
MERGGAERDHQLNNYNLQVSFSSSSVAANNIHELGFVHFADHNLSFLAPSSQSSQISQPLQAASVSVTPPTTINTNV
AAGGSNNVTGGLGFSHNELVINRSSWNSDQVETLDPKAV
NDENCGGNANEGNNSWWKTSSSDKGKVKIRRKLREPRFCFQTRSDIDVLDDGYKWRKYGQKVVKNSLHPRSYY
RCTHSNCRVKKRVERLSEDCRMVITTYEGRHNHSPCDDSNSSD
HDCFTSF

CaWRKY5
>Capana01g004471 [mRNA] locus=Chr01:300738347-300739954
MDCSFNWQYKTLINELTRGIEHAKQLKAYLSSVASTSENQELLLQKILSSYEQSLSDDKKRCFKEHQELIDISKKRK
SQLTRTEQVKVSAESGFEGPTDDGYSWRKYGQKHILGAKYPRSYYRCTYRHMQNCWATKQVQRSDDDATVYEI
TYRGSHNCRQATNRASLEKQELKKQAVYQTGQQYSNQALMNSRANLKVDTDDLEKNETACPFSFPPTFSGLTDE
NQHFQISDVDDNRTTSQSQAAAR
>CA01g34460
MDCSFNWQYKTLINELTRGIEHAKQLKAYLSSVASTSENQELLLQKILSSYEQSLVILKRTGSTVHSSKPLPPMCGA
IESSVSVDGSPKSDDKKRCFKEHQELLDISKKRKSQLTRTEQVKVSAESGFEGPTDDGYSWRKYGQKHILGAKYPR
SYYRCTYRHMQNCWATKQVQRSDDDATVYEITYRGSHNCRQATNRASLEKQELKKQAVYQTGQQYSNQALMN
SRANLKVDTDDLEKNETACPFSFPPTFSGLTDENQHFQISDVDDNRTTSQSQAAAR

CaWRKY6
>Capana01g004472 [mRNA] locus=Chr01:300742280-300745209
MRKLQPTWTEQVKVSPKSGFEGPTDDGYSWRKYGQKDILGAKYPRSYYRCTYRHMQNCWATKQVQRSDDDPT
VFDVTYRGSHSCHHATYYVQQSTPPEKREFKKEAVYQNRQNYSTQALMSLRANLRVDTNDLDKNEQAACHFSFP
PTFSSGLTDENHRRFQISHVDENLIGSGYSASFVSPTTPESNYFSVSSSSQMNGYGMIHNLNHSESDLTDIFSANTSTT

SSPIVGDFSLDNLELDTNFPFNNPNFFS
>CA01g34470
MDCAVNWEYKTLINELTQGIEHTKQLRAHFSSVDSTIQNQELLLQKILSSYEQSLLILKCSVGGSMVQSSSAMMPT
CGVIESSVVSVYGSPKSDDKKRSFQDHHEVIDISKKRKLQPTWTEQVKVSPKSGFEGPTDDGYSWRKYGQKDILG
AKYPRSYYRCTYRHMQNCWATKQVQRSDDDPTVFDVTYRGSHSCHHATYYVQQSTSPEKREFKKEAVYQNRQN
YSTQALMSLRANLRVDTNDLDKNEQAACHFSFPPTFSSGLTDENHRRFQISHVDENLIGSGYSASFVSPTTPESNYF
SVSSSSQMNGYGMIHNLNHSESDLTDIFSANTSTTSSPIVGDFSLDNLELDTNFPFNNPNFFS

CaWRKY7

>Capana02g000212 [mRNA] locus=Chr02:26415840-26420359
MIPQASSSLFNPTQENNNHFGPNIDSPSMKIRRPNIFSTMLQISPTTKLAMSPCDIACSSPNSNVNVIGALVPTQDAIIS
PNSSKTCLVENSGLQISSPRNTGIKRRKSQAKKVVCIPAPAPANSRQGGEVVPSDLWAWRKYGQKPIKGSPYPRGY
YRCSSSKGCSARKQVERSRTDPNMLVITYTSEHNHPWPTQRNALAGSTRSQPNNSKHTTTSKNNTNIMPNNSQYQ
GDTSFNEDEQNERNINHDNNVAQANISTYPKVKEEVAEEDLQQQLGEMRNVEFSKGSYQPILPDSSNQCHEDFFA
DLVELEADPLNFLFANTLSGDINEVGQKKAIDAFNLYNWSKDRNTNINNKGTQADT
>CA02g03480
MCSPQKDMTNNYQGDLADIFRGGNSTTSGDQSSTSVVPVPDGWQFPSINYSASVIEEPTASLVQDFGDPFCNLRDP
LLFHDLDMIPQASSSLFNPTQENNNHFGPNIDSPSMKIRRPNIFSTMLQISPTTKLAMSPCDIACSSPNSNVNVIGALV
PTQDAIISPNSSKTCLVENSGLQISSPRNTGIKRRKSQAKKVVCIPAPAPANSRQGGEVVPSDLWAWRKYGQKPIKG
SPYPRGYYRCSSSKGCSARKQVERSRTDPNMLVITYTSEHNHPWPTQRNALAGSTRSQPNNSKHTTTSKNNTNIMP
NNSQYQGDTSFNEDEQNERNINHDNNVAQANISTYPKVKEEVAEEDLQQQLGEMRNVEFSKGSYQPILPDSSNQC
HEDFFADLVELEADPLNFLFANTLSGDINEVGQKKAIDAFNLYNWSKDRNTNINNKGTQADT

CaWRKY8

>Capana02g000680 [mRNA] locus=Chr02:83748217-83749821

MEIEIAADAAITKFKKVNSLLDRFRTGHARFRRAPIDNLKKDYVDPEVSSAVKPPLSSSSSFKIRKCTSSENGFSGKC
SGSSGRCHCSKRRKLRPKRVVRVPAISMKLSDIPPDDYSWRKYGQKPIKGSPHPRAYYKCSSVRGCPARKHVERAL
DEPTMLVVTYESEHNHSLSVAETSSLILESS
>CA02g01800
MEIEIAADAAITKFKKVNSLLDRFRTGHARFRRAPIDNLKKDYVDPEVYCLTPIQQLPPSAYDLNNNQIFQNPKQEL
VTKSINFSHAPEIWCANSFNMSTLTGETESEQISNLSQVSSAVKPPLSSSSSFKIRKCTSSENGFSGKCSGSSGRCHCS
KRRKLRPKRVVRVPAISMKLSDIPPDDYSWRKYGQKPIKGSPHPRAYYKCSSVRGCPARKHVERALDEPTMLVVT
YESEHNHSLSVAETSSLILESS

CaWRKY9

>Capana02g000918 [mRNA] locus=Chr02:102411100-102416945
MEKNLDNSLSGASAVKEIHNHEEDDIRKALKDDEFKSALAEVNEVKLENARLKMLLQQIENDYNSLQTRFFNICQP
DLKKSVNPTCTSEKIADEEECELVSLRLGRSPSPSESKKVDKKRTREDYHCVQSNDGLKLGLDYSSGVSESDLLKP
NNDPSPGPTSEVVKTMRKGDDESVKKRAGDDEVSQPNVKRARVSVRTKCDYPTINDGCQWRKYGQKISRGNPCP
RAYYRCSVAPLCPVRKQVQRCLEDMSILITTYEGTHNHSLPIEATAMASTTAAAASMLLSGSSTSSQSPKNFTNLA
NYSKTTPLYLSNSSSNPFPTITLDFTAFPTTSSFTSFNFPSNFQPGSGLLSNSLSFSSPESSTIPKILGSGCLNYDSTSTLP
YHKNLINIGSSQKQFDQPFIGKNNTSTSDKLKEDSSQQALTETLTKAITSDPSFQSVLAAAISSMVGATKT>CA02g08
530MRKGDDESVKKRAGDDEVSQPNVKRARVSVRTKCDYPTINDGCQWRKYGQKISRGNPCPRSYYRCSVAPLCP
VRKQVQRCLEDMSILITTYEGTHNHSLPIEATAMASTTAAAASMLLSGSSTSSQSPKNFTNLANYSKTTPLYLSNSS
SNPFPTITLDFTAFPTTSSFTSFNFPSNFQPGSGLLSNSLSFSSPESSTIPKILGSGCLNYDSTSTLPYHKNLINIGSSQKQ
FDQPFIGKNNTSTSDKLKEDSSQQALTETLTKAITSDPSFQSVLAAAISSMVGATKT

CaWRKY10

>Capana02g001642 [mRNA] locus=Chr02:129707611-129709054
MEGRFNNFFVSEQDDSENSPENSSDSPRSAMFNDNKMITSTSSPNRRSIEKRVVSVPIKEVEGSKMKGEISMPPSDS

WAWRKYGQKPIKGSPYPRGYYRCSSSKGCPARKQVERSRADPNMLIVTYSCEHNHPWPASRSNQHNHRTITPTSC
TNNNTKTKTKTIASLTASPATTTIITTSDIPVLHFEQEKATTDFAVRPSEPNSDEKFVNLGESSLINAEFGWFSDLVEC
NSTTILESPILTQVEVNDFDMSSTLTMQEEDVSLFADLGELPECSRVFGRGMMERDEERDRHSLTPWCGTTG>CA02
g13500MEGRFNNFFVSEQDDSENSPENSSDSPRSAMFNDNKMITSTSSPKRSRRSIEKRVVSVPIKEVEGSKMKGEIS
MPPSDSWAWRKYGQKPIKGSPYPRGYYRCSSSKGCPARKQVERSRADPNMLIVTYSCEHNHPWPASRSNQHNHR
TITPTSCTNNNTKTKTKTIASLTASPATTTIITTSDIPILHFEQQKATTDFAVRPSEPNSDEKFVNLGESSLINAEFGWF
SDLVECNSTTILESPILTQVEVNDFDMSSTLTMQEEDVSLFADLGELPECSRVFGRGMMERDEERDRHSLTPWCGT
TG

CaWRKY11

>Capana02g002230 [mRNA] locus=Chr02:142213621-142216150
MDKGWGLTLESSSSSDKVGFFMNKPVFGFNLSPRLNPAEMFPSSDDKRAIVNEVDFFSEKKPIVKKENSQGDRTDQ
CVVNTGLQLVIANAGSDQSTVDDGISSELVLEDKRAKIQQLAQLQVELQRMNSENQRLKGMLTQVNNSYSALQM
HLVVEAKFNDEKKQEKEGTIVPRQFMELGPSGSKADPLDEPSNSHTSSEERTLSGSPRNNMELLSRDKAIGREESPE
SESWAPNKVPKLMNSSKPVEQPTEATMRKARVSVRARSEAPMISDGCQWRKYGQKMAKGNPCPRAYYRCTMAV
GCPVRKQVQRCAEDRTILITTYEGTHNHPLPPAAMAMASTTSAAANMLLSGSMPSADGLMNTNFLARAMLPCSS
NMATISASAPFPTVTLDLTAQNSNAALPNYHQRVNHANNAQFQFPLPAGLNHPNFIASMSAPQMPQVLGQAMYN
QSKFSGLQVSQDNIHHPSISHDTLSAATAAITADPNFTAALAAAISSIIGCGSHPNNNGNSTMSGPSSNNNNTSSFPG
N>CA02g18540MDKGWGLTLESSSSSDKVGFFMNKPVFGFNLSPRLNPAEMFPSSDDKRAIVNEVDFFSEKKPIVKK
ENSQGDRTDQCVVNTGLQLVIANAGSDQSTVDDGISSELVLEDKRAKIQLAQLQVELQRMNSENQRLKGMLTQV
NNSYSALQMHLVTLMQQQQQQQQQQMISRTESTHAHEVVEAKFNDEKKQEKEGTIVPRQFMELGPSGSKADPL
DEPSNSHTSSEERTLSGSPRNNMELLSRDKAIGREESPESESWAPNKVPKLMNSSKPVEQPTEATMRKARVSVRAR
SEAPMISDGCQWRKYGQKMAKGNPCPRAYYRCTMAVGCPVRKQVQRCAEDRTILITTYEGTHNHPLPPAAMAM
ASTTSAAANMLLSGSMPSADGLMNTNFLARAMLPCSSNMATISASAPFPTVTLDLTAQNSNAALPNYHQRVNHA
NNAQFQFPLPAGLNHPNFIASMSAPQMPQVLGQAMYNQSKFSGLQVSQDNIHHPSISHDTLSAATAAITADPNFTA

ALAAAISSIIGCGSHPNNNGNSTMSGPSSNNNNTSSFPGN

CaWRKY12

>Capana02g003053 [mRNA] locus=Chr02:154730960-154732560
MAVDLMMDYRNTSNSSSNNCINFVTKLEEKAVVQEAASGLESVEKLIRMLSRNKSPQIQQNKSPMEIELVADAA
VTKFKKVISLLDRNRTGHARFRRAPLAATTSPSPTNCNKDIVDTKVYSPTPIQQVPLVSYEHYNPLVPPKTISFSYSP
EMSRTNSFNISSLTGDTESKQHSSSSAAFQITNLSSQVTNSAGKPPLSSSSLKRKCSLSENAVSGKCSGPSGRCHCSK
RRKLRLKRVIRVPAISMKLADIPPDDYSWRKYGQKPIKGSPHPSFVCPHEKKKGFPINVNWLLIRLVNQSVE
>CA02g14640
MAVDLMMDYRNTSNSSSNNCINFVTKLEEKAVVQEAASGLESVEKLIRMLSRNKSPQIQQNKSPMEIELVADAA
VTKFKKVISLLDRNRTGHARFRRAPLAATTSPSPTNCNKDIVDTKVYSPTPIQQVPLVSYEHYNPLVPPKTISFSYSP
EMSRTNSFNISSLTGDTESKQHSSSSAAFQITNLSSQVTNSAGKPPLSSSSLKRKCSLSENAVSGKCSGPSGRCHCSK
RRKLRLKRVIRVPAISMKLADIPPDDYSWRKYGQKPIKGSPHPRGYYKCSSVRGCPARKHVERASDDPTMLIVTYE
GEHNHSLSVAETSSLILESS

CaWRKY13

>Capana02g003339 [mRNA] locus=Chr02:159222405-159225272
MGETGGEASAISFPALTIPPRPSYESFFNMSSFSPGPMSLVSSFLSEQSPDSADRPSFSQLLAGVIASPTLLPDDSGDPS
GSEKSTGYRKNRPMNLALAQSPLFMIPSGFSPSGFLNSPGFLSPLQSPFGMSHQQALAHVTAQAECSSSYMQMQAE
DQCSAQVASAEAALGNELLTDPKESSLQIKECLQPRLDKKPSDKQGKQFELTEVPQFENKTSFGAFDKSACDGYN
WRKYGQKKVKATECPRSYYKCTHLKCPAKKKVEKSVDGHITEITYNGRHNHAQPTKQRKDGSALDSTDGSGVQP
DISTHDWTVMNSSDGSSPSHSEQVPNQMASELVKKECDETKSNLIEVDEGHDEPDAKRTSEVDILDDGYRWRKYG
QKAVKGTQHPRSYYRCTYAGCNVRKQVERASTDPKAVITTYEGKHNHDIPTVIRNRGTRNTAKDTWR
>CA02g27910
MGETGGEASAISFPALTIPPRPSYESFFNMSSFSPGPMSLVSSFLSEQSPDSADRPSFSQLLAGVIASPTLLPDDSGDPS

GSEKSTGYRKNRPMNLALAQSPLFMIPSGFSPSGFLNSPGFLSPLQSPFGMSHQQALAHVTAQAECSSSYMQMQAE
DQCSAQVASAEAALGNELLTDPKESSLQIKECLQPRLDKKPSDKQGKQFELTEVPQFENKTSFGAFDKSACDGYN
WRKYGQKKVKATECPRSYYKCTHLKCPAKKKVEKSVDGHITEITYNGRHNHAQPTKQRKDGSALDSTDGSGVQP
DISTHDWTVMNSSDGSSPSHSEQVPNQMASELVKKECDETKSNLIEVDEGHDEPDAKRTMSHPSVVKQEEKLYSN
KSMYLIQLLCCRKMAVETLASSHGTVAESKIILQTRSEVDILDDGYRWRKYGQKAVKGTQHPRSYYRCTYAGCN
VRKQVERASTDPKAVITTYEGKHNHDIPTVIRNRGTRNTAKDTWR

CaWRKY14

>Capana02g003661 [mRNA] locus=Chr02:163357641-163359108
MENNQPMLLLGSASSYYNSMNGGLKTSFTQISRDQMEVDTSENHNKYISSLSVKKKGDNKKIKKPRFAFQTRSQV
DILDDGYRWRKYGQKAVKNNNYPRSYYRCTHEGCNVKKQVQRLSKDEGVVVTTYEGMHTHPIDKPNDNFEQIL
HQMQIFPNHPLN
>CA02g30960
MLLLGSASSYYNSMNGGLKTSFTQISRDQMEVDTSENHNKYISSLSVKKKGDNKKIKKPRFAFQTRSQVDILDDGY
RWRKYGQKAVKNNNYPRSYYRCTHEGCNVKKQVQRLSKDEGVVVTTYEGMHTHPIDKPNDNFEQILHQMQIFP
NHPLN

CaWRKY15

>Capana03g000473 [mRNA] locus=Chr03:6501568-6503375
MEFTSLVDTSLDLSFRPLRVPDDIPKQEVESNFIGLGRDLMPDKDDQAGDLLEELNRVSAENKKLTEMLTVMCQN
YNALRNQLTEYLNKQNSTTSTAADNNHDHHSDGSKKRKVENNNNEIVKSVQGLHSESSSSDEDSSNKKPREQHIK
TNTCRVYVKTEASDTSLIVKDGYQWRKYGQKVTRDNPSPRAYFKCSFAPTCPVKKKVQRSLEDQSILVATYEGEH
NHSKMDGSGPVTTSPSSRLNPKNTLVGANTTTVMPCSSTSIINTPSGPTLTLDLTQPKKLQNDEKKVNSNTSTSNAS
GQKSKSPGGHDHHQQNRPEFQQLFIDQMASSLTKDPSFQAALAAAISGKFLQNNHTDK
>CA03g32070

MEFTSLVDTSLDLSFRPLRVPDDIPKQEVESNFIGLGRDLMPDKDDQAGDLLEELNRVSAENKKLTEMLTVMCQN
YNALRNQLTEYLNKQNSTTSTAADNNHDHHSDGSKKRKVENNNNEIVKSVQGLHSESSSSDEDSSNKKPREQHIK
TNTCRVYVKTEASDTSLIVKDGYQWRKYGQKVTRDNPSPRAYFKCSFAPTCPVKKKVQRSVEDQSILVATYEGEH
NHSKMDGSGPVTTSPSSRLNPKNTLVGANTTTVMPCSSTSIINTPSGPTLTLDLTQPKKLQNDQKKVNSNTSTSNAS
GQKSKSPGGHDHHQQNRPEFQQLFIDQMASSLTKDPSFQAALAAAISGKFLQNNHTDK

CaWRKY16

>Capana03g001099 [mRNA] locus=Chr03:18684171-18687268
MEASFKKSNIHGHVFKVEKINADDKGFVEDTKVLKFSKKRELHEDHKSKSSQLQKDYLTSDKEDDQLESAKADM
EEVMEENQRLKKHLDRVMKDYQNLQMQFHEISQRGVEKSNNVKHDEAELVSLSLGRTSSDTKIELSKILNKKENV
EEEDNLTLGLDCKFQSSANAPTKSSPSNLSPENSLGEVKDEKGTETWPAHKGLKTIRDEEDDVVQQNPTKRAKVS
VRIRCDTPTMNDGCQWRKYGQKIAKGNPCPRAYYRCTVAPSCPVRKQVQRCIQDMSILIITYEGTHNHPLPLSATS
MAFTTSAAASMLLSGSSTSESGST
>CA00g80710
MIFIFQFSKKRELHEDHKSKSSQLQKDYLTSDKEDDQLESAKADMEEVMEENQRLKKHLDRVMKDYQNLQMQF
QEISQRGVEKSNNVKHDEAELVSLSLGRTSSDTKIELSKILNKKENVEEEDNLTLGLDCKFQSSANAPTKSSPSNLSP
ENSLGEVKDEKGTETWPAHKGLKTIRDEEDDVVQQNPTKRAKVSVRIRCDTPTMNDGCQWRKYGQKIAKGNPCP
RAYYRCTVAPSCPVRKQ

CaWRKY17

>Capana03g001962 [mRNA] locus=Chr03:39904102-39904887
MAENQNDWDLWAIVRSCCNMNNSVHDDVISFDNVNSTSVLVDHGVHEDPTHGNSANNARTSTSFQEQSGCAGD
FSDSFATENKHYFGLDEVLGLSKNVNTNSRIEPHNPENQTESITDTPLVEHEKKKNKNKKTRYSLSSSSIEAGKAFP
YCRKSTERYEVLAEKLSEADQWRWRKYGMKRTGGSPFLKSYYRCNQGEDCPARRHVQQSSTDSNKVIVTYRGQ
HSHPPPNQHIATVQGNHNAAAPVEDPPFPSSPSTLLFN

>CA03g20260
MAENQNDWDLWAIVRSCCNMNNSVHDDVISFDNVNSTSVLVDHGVHEDPTHGNSANNARTSTSFQEQSGCAGD
FSDSFATENKHYFGLDEVLGLSKNVNTNSRIEPHNPENQTESITDTPLVEHEKKKNKNKKTRYSLSSSSIEAGKAFP
YCRKSTERYEVLAEKLSEADQWRWRKYGMKRTGGSPFLKSYYRCNQGEDCPARRHVQQSSTDSNKVIVTYRGQ
HSHPPPNQHIATVQGNHNAAAPVEDPPFPSSPSTLLFN

CaWRKY18

>Capana03g002072 [mRNA] locus=Chr03:44184049-44185319
MDNYGADNTNVEFNRIINELTQGQDLVQQLQLHLNAPNYNSSASFENTREILLHNIQSKFDKALSILQYNSTTGDN
SNSPLTHSTSPAIPVFGVSDSPRSSPPHSEDSDRDLESKDPHATRKRKSTTPRWTKQVQIHPGAPLEGTLDDGFSWR
KYGQKDILGAKHPRGYYRCTLRHVQGCLATKQVQRSDEDPTIFEVTYRGRHTCSQGGGGGASASNVHPAPLPLV
VTIPQNQEPNLGNHEQYQLIPAPHQNSPEILLDFQKNLSISKDDFNFNTHHDHPNNVPYIPPYSNFPSSSSSHVNTDH
QDYTFVANSSTIPINNFVENFPPSSNNMSAGTSQMNNADYQFNSMGFESNFPYNYQGFSS
>CA03g19220
MDNYGADNTNVEFNRIINELTQGRDLVQQLQLHLNAPNYNSSASFENTREILLHNIQSKFDKALSILQYNSTTGDNS
NSPLTHSTSPAIPVFGVSDSPRSSPPHSEDSDRDLESKDPHATRKRKSTTPRWTKQVQIHPGAPLEGTLDDGFSWRK
YGQKDILGAKHPRGYYRCTLRHVQGCLATKQVQRSDEDPTIFEVTYRGRHTCSQGGGGGASASNVHPAPLPLVVT
IPQNQEPNLGNHEQYQLIPAPHQNSPEILLDFQKNLSISKDDFNFNTHHDHPNNVPYIPPYSNFPSSSSSHVNTDHQD
YTFVANFSTIPINNFVENFPPSSNNMSAGTSQMNNADYQFNSMGFESNFPYNYQGFSS

CaWRKY19

>Capana03g002134 [mRNA] locus=Chr03:48509522-48510754
MDDNNWDLGAVIRNCGINRPSNDITPNLGSESLNFDDDDLNFLDRIFGVDNNNFDYIAPTNFSISRQEKSYDQFDDII
NPTTPVSIIANPFKITNQNDNQQIYLPPIQPAQVSQQVFLSSPSGVEARECVPTTTTTTISHECINLQQQLLMWDSTLT
MRNPPIQIRKSKNQSIWTTYELFQEELTDDIWSWRKYGQKFIKGSSFPRNYFKCNTSELCQARKQIEKSSKNDCFFL

VAYSGMHNHDPPIIRRSFSDWNHSSKYKLPKGINIIPKALKLNASPFSSKSGKRYRASSTLETESTSRKKNKMIVET
MKNNVDDEEEENINEDVPKGFEELN
>CA03g25570
MDDNNWDLGAVIRNCGINRPSNDITPNLGSESLNFDDDDLNFLDRIFGVDNNNFDYIAPTNFSISRQEKSYDQFDDII
NPTTPVSIIANPFKITNQNDNQQIYLPPIQPAQVSQQVFLSSPSGVEARECVPTTTTTTISHECINLQQQLLMWDSTLT
MRNPPIQIRKSKNQSIWTTYELFQEELTDDIWSWRKYGQKFIKGSPFPRNYFKCNTSELCQARKQIEKSSKNDCFFL
VAYSGMHNHDPPIIRRSFSDWNHSSKYKLPKGINIIPKALKLNASPFSSKSGKRYRASSTLETESTSRKKNKMIVET
MKNNVDDKEEENINEDVPKGFEELN

CaWRKY20

>Capana03g002635 [mRNA] locus=Chr03:93204626-93207341
MEELVDETPRKRLIKELVEGKSFAKQLQSLLQQPNIEHYDGSVLADELVLKIWRSFTQAITELNTLVDSNSILVQTQ
MEVEKTEEVDQADTGDRSNSELKKKGKQGGKDRRGCYKRRNNSGSWMRESETMNDGCAWRKYGQKSILNSKY
PRCYYRCTHKYDQDCRATKQVHIMQENPKLMYHTTYFGNHTCNPAKIRKHINNAQFNHSMLECPPFEVKPKIPSS
VTHDSTEEEEESLKGQSDNVSSTMDSYLWEDFMPSSPSAHDSTLASHNSSYFQGLISSEMGDLVKFSDFEAIEFF
>CA03g12230
MEELVDETPRKRLIKELVEGKSFAKQLQSLLQQPNIEHYDGSVLADELVLKIWRSFTQAITELNTLVDSNSILVQTQ
MEPIXXXXNSELKKKGKQGGKDRRGCYKRRNNSGSWMRESETMNDGCAWRKYGQKSILNSKYPRCYYRCTHK
YDQDCRATKQVHIMQENPKIMYHTTYFGNHTCNPAKIRKHINNAQFNHSMLECPPFEVKPKIPSSVTHDSTEEEEE
SLKGQSDNVSSTMDSYLWEDFMPPSPSAHDSTLASHNSSYFQGLISSEMGDLVKFSDFEAIEFF

CaWRKY21

>Capana03g003085 [mRNA] locus=Chr03:167540037-167548534
MEDSHSHSHYPRPYSNSAPLSSINETSEQVKFSSSDAALFSSSDAAFVYSSAFGSNSSSSAKYKLMSPAKLPISRSPCI
TIPPGLSPSSFLESPVLLSNIKAEPSPTTGSFSKFQLMQGSSGSAAFSLMRSCSSGNAYGETTGEFEFEFPIGSSSTSGSL

AKEAVICAGFNQQQSEPLIQVQNRCPSQSLAPPALVKSEMPNSKELSLPTPVCLDASLISTAAAATDNEEVNQRGQS
NPSSHRSSADNKNVSSVTADRSSEDGYNWRKYGQKLVKGSEFPRSYYKCTYPNCEVKKIFERPPDGQITEIVYKGS
HDHPKPQPNHRFTPGALTSVQEDRGEREACLTGQEDKFNTNAQTSNTEPSGTPLSPQQADDDGLEGTVSQLHSSN
DQMDEDDSFAKRRKMDGVMDIIPVVKPIREPRVVVQTVSEVDILDDGYRWRKYGQKVVRGNPNPRSYYKCTNA
RCPVRKHVERASHDPKAVITTYEGKHNHDVPTARTNSHEMAGSAPVTGSSRVRVEENGAISLDLGVGIGHGMENR
RNGQLHTLPAETVRSQGQVSSSIVMVVQPAAVAACYSIVNGGMNRFGTIENRVQGTGFETLPLQSSAQYPQNYGM
ILLGP
>CA03g12030
MPNSKELSLPTPVCLDASLISTAAAATDNEEVNQRGQSNPSSHRSSADNKNVSSVTADRSSEDGYNWRKYGQKLV
KGSEFPRSYYKCTYPNCEVKKIFERPPDGQITEIVYKGSHDHPKPQPNHRFTPGALTSVQEDRGEREACLTGQEVIP
LSGLSFLEDKFNTNAQTSNTEPSGTPLSPQQADDDGLEGTVSQLHSSNDQMDEDDSFAKRSRKMDGVMDIIPVVKP
IREPRVVVQTVSEVDILDDGYRWRKYGQKVVRGNPNPRSYYKCTNARCPVRKHVERASHDPKAVITTYEGKHNH
DVPTARTNSHEMAGSAPVTGSSRVRVEENGAISLDLGVGIGHGMENRRNGQLHTLPAETVRSQGQVSSSIVMVVQ
PAAVAACYSIVNGGMNRFGTIENRVQGTGFETLPLQSSAQYPQNYGMILLGP

CaWRKY22

>Capana03g003279 [mRNA] locus=Chr03:203453030-203454904
MEEIEEANRVAVESCHRVITMLSQPHDQKQFGNVARETGEAVHKFKKVATLLNSNLGHARVRKAKKIITPLPQNL
LLESPSCKTYDQPKSLQLLPITEIGSNVKSTLTLANPSLELSSHSKNPLQLAQQTPLSSYHFLQQQQRRYQLQQQL
KQQTDMMYRRSNSGISLNFDSSTCTPTMSSTRSFISSLSIDGSVANLDGNANAFHFIGASRSADQSSFQHRKRCSGR
GEEGSVKCGSSGRCHCSKKRKHRVKRSIKVPAISNKLADIPPDEYSWRKYGQKPIKGSPHPRGYYKCSSMRGCPAR
KHVERCLEEPSMLIVTYEGEHNHSRLPX
>CA06g07080
MEEIEEANRVAVESCHRVITMLSQPHDQKQFGNVARETGEAVHKFKKVATLLNSNLGHARVRKAKKIITPLPQNL
LLESPSCKTYDQPKSLQLLPITEIGSNVKSTLTLANPSLELSSHSKNPLQLAQQTPLSSYHFLQQQQRRYQLQQQL

KQQTDMMYRRSNSGISLNFDSSTCTPTMSSTRSFISSLSIDGSVANLDGNANAFHFIGASRSADQSSFQHRKRCSGR
GEEGSVKCGSSGRCHCSKKRKHRVKRSIKVPAISNKLADIPPDEYSWRKYGQKPIKGSPHPRGYYKCSSMRGCPAR
KHVERCLEEPSMLIVTYEGEHNHSRLPSQSANA

CaWRKY23

>Capana04g000568 [mRNA] locus=Chr04:9062869-9064395
MAVELMSTGYRPNNFSSKMEENSVQEAAAAGLQSVEKLIRLLSQSQQQQQQNHQQQTNFQNSSSNDYQVVADV
AVNKFKKFISLLDKNRTGHARFRRGPITSPPPPPPPPPVPAKPQQNKQQMEESEKQQSSATKIYCPTPIQRLPPLPHN
HHQQILIKNGSIERKEAASSTTINFASPSPATSFMSSLTGETESLQNSLSSGFQITNLSQVSSAGRPPISTSSFKRKCSSM
DDIALKCNSAGGSSGRCHCPKKRKSRVKRVVRVPAISMKMADIPPDDYSWRKYGQKPIKGSPHPRGYYKCSSVRG
CPARKHVERALDDPAMLIVTYEGEHNHSHSITETPATHVLESS
>CA04g18300
MSTGYRPNNFSSKMEENSVQEAAAAGLQSVEKLIRLLSQSQQQQQQNHQQQTNFQNSSSNDYQVVADVAVNKFK
KFISLLDKNRTGHARFRRAKPQQNKQQMEESEKQQSSATKIYCPTPIQRLPPLPHNHHQQILIKNGSIERKEAASSTT
INFASPSPATSFMSSLTGETESLQNSLSSGFQITNLSQRKCSSMDDIALKCNSAGGSSGRCHCPKKRKSRVKRVVRV
PAISMKMADIPPDDYSWRKYGQKPIKGSPHPRGYYKCSSVRGCPARKHVERALDDPAMLIVTYEGEHNHSHSITET
PATHVLESS

CaWRKY24

>Capana04g001820 [mRNA] locus=Chr04:115696954-115705628
MGEKLKVPAVSALPALTIPPRETFFGGGNMSYFSPGPITLVSSYFSESEHPSFSQLLAGAMASPLAKPLLTKEEEANC
KEGNLGYKQNRPMSLMVAHSPFFTPFSPSGLLNSPAFLSPLQSPFGMSHQQALAHVTAQAALSQSYLQGTSAQVL
GTSDLDESSLQPQLDTMPSDQQIKKFELPQISQSEEKPYLNSVDKPASDGYNWRKYGQKMVKASECPRSYYKCTH
VKCPVRKKVERSVDGHVTEITYKGHHNHELPQPNKRRRDSGAQDGSDCSKANPEIETHTEIETSGLNGAHLAHSE
QVSTERASEPPVLKDYDEIVDTATATGKEQDDESNVKRMKTTVETPILFSSHKAESESKIVVQTRSEVDILDDGFK

WRKYGQKVVKGNHHPRSYYRCTYPGCNVRKHVERASTDPKAVITTYEGKHNHESPIARNRSHSAAQDSTCQLNE
QEIATWRPSLHEKVALHANEIPVCRQLKDEHMAA
>CA04g11710
SPFGMSHQQALAHVTAQAALSQSYLQGTSAQVLGTSDLDESSLQPQLDTMPSDQQIKKFELPQISQSEEKPYLNSV
DKPASDGYNWRKYGQKMVKASECPRSYYKCTHVKCPVRKKVERSVDGHVTEITYKGHHNHELPQPNKRRRDSG
AQDGSDCSKANPEIETHTEIETSGLNGAHLAHSEQVSTERASEPPVLKDYDEIVDTATATGKEQDDESNVKRMKTT
VETPILFSSHKAESESKIVVQTRSEVDILDDGFKWRKYGQKVVKGNHHPR

CaWRKY25

>Capana05g002502 [mRNA] locus=Chr05:216311579-216315172
MSSLPPCRTDFLTIPPGISPAALLDSTFILPSSMTPTDNVAQILHCIESTNNQIPQQQQEFTKKQNQFEQRGIFSSSISPN
NSSDDGYTWRKYGQKHVKGSNFPRSYYKCTQQTCPVRKKVECAPNGQVIEIVYNGPHNHPKTQHLRRKAMDAD
SYVVGQENGSSSSSLIWRNDQQLEYNKDVNSCCNELERKPSASVLSDVSSDPMLSNNLKSMNVFESDATHELSSTL
NSFDDEDEDLATQEGNFLGDGINEFEFEPKRRKKESYSVEPSLLSRTVREPKVVLQVESETDILEDGYRWRKYGQK
VVKGNPNPRSYYKCTSAGCLVRKHVERASDDLKSVITTYEGKHNHEVPSANKTNGVAGHFRSASMLNNGQQQPA
CTASRKSLKDSNIRVQFQDLPIPFERKFFMGSEYLRPNFGASYLSDLSFGGGSLQLPDFAIPLPLPSRMSFPARQNEPR
LGDFQMNNHLLLPNGASTFLAAGNTRHINDDNNNSRLLKAKDEVQYWT
>CA05g20090
MEALEQLVKLLPCDQRVTHGFKLWKQPLPELQDKTPTDNVAQILHCIESTNNQIPQQQQEFTKKQNQFEQRGIFSS
SISPNNSSDDGYTWRKYGQKHVKGSNFPRSYYKCTQQTCPVRKKVECAPNGQVIEIVYNGPHNHPKTQHLRRKA
MDADSYVVGQENGSSSSSLIWRNDQQLEYNKDVNSCCNELERKPSASVLSDVSSDPMLSNNLKSMNVFESDATHE
LSSTLNSFDDEDEDLATQEGNFLGDGINEFEFEPKRRKKESYSVEPSLLSRTVREPKVVLQVESETDILEDGYRWRK
YGQKVVKGNPNPRSYYKCTSAGCLVRKHVERASDDLKSVITTYEGKHNHEVPSANKTNGVAGHFRSASMLNNG
QQQPACTASRKSLKDSNIRVQFQDLPIPFERKFFMGSEYLRPNFGASYLSDLSFGGGSLQLPDFAIPLPLPSRMSFPA
RQNEPRLGDFQMNNHLLLPNGASTFLAAGNTRHINDDNNNSRLLKAKDEVQYWT

CaWRKY26

>Capana06g001008 [mRNA] locus=Chr06:17571223-17578663
MENKNKAEYYSPDDEDQENIIHKFGKGRKEREDDKSKPSSPHHKDFMAIDNNIKGVAVNVMVKRERSPPELNSM
ASSSAHKEKDDQLALAKVEMREVMEENQRLRFHLDRIMKEYRNLQNQFHDIVQREVDQKSSSTVNTTQHESDHE
TNELVSLSLGRATSDMKKEELSKILKKDKGRDDEDVNKSLDLGLDCKFEECSPVKNRSPENSLDDHQANKDENGE
TSTTTWPPNKNLKTMRNDGDNGDDVSQQNPTKRARVSVRVRCDAPTMNDGCQWRKYGQKIAKGNPCPRAYYR
CTVAPNCPVRKQVQRCAEDMSILITTYEGTHNHTLPLSATAMASTTSAAANMLLSGSSSSSDPSPQITATTTNTATA
TTSANINGLNFYISDTSKHKSPFYFPNSSISASTLNNSHPTITLDLTSTSSSSSSSLSHLNRMSNNLHPRYNYNNSSTNL
NFSSVLESNSLPISWTNYQNQTCNKNNQNFGSLNFSSRPNQENIFQSYLQKNNNIIPTQSSFPPDTIAAATKAITSDPN
FHSALAAALTSIIGNTGIENKPGHNFNVSEPFPVLSSLPSSSNPNKCSSSFLNKPTSSSANNSSQQPGNNNNLVFFAQS
SSSSLPFSTSNKGKSTSPSDS
>CA06g19170
MVKRERSPPELNSMASSSAHKEKDDQLALAKVEMREVMEENQRLRFHLDRIMKEYRNLQNQFHDIVQREVDQKS
SSTVNTTQHESDHETNELVSLSLGRATSDMKKEELSKILKKDKGRDDEDVNKSLDLGLDCKFEECSPVKNRSPENS
LDDHQANKDENGETSTTTWPPNKNLKTMRNDGDNGDDVSQQNPTKRARVSVRVRCDAPTMNDGCQWRKYGQ
KIAKGNPCPRAYYRCTVAPNCPVRKQVQRCAEDMSILITTYEGTHNHTLPLSATAMASTTSAAANMLLSGSSSSSD
PSPQITATTTNTATATTSANINGLNFYISDTSKHKSPFYFPNSSISASTLNNSHPTITLDLTSTSSSSSSSLSHLNRMSNN
LHPRYNYNNSSTNLNFSSVLESNSLPISWTNYQNQTCNKNNQNFGSLNFSSRPNQENIFQSYLQKNNNIIPTQSSFPP
DTIAAATKAITSDPNFHSALAAALTSIIGNTGIENKPGHNFNVSEPFPVLSSLPSSSNPNKCSSSFLNKPTSSSANNSSQ
QPGNNNNLVFFAQSSSSSLPFSTSNKGKSTSPSDS

CaWRKY27

>Capana06g001110 [mRNA] locus=Chr06:20042156-20043655
MEFTSLVDTSLDLSFRPRPVLDKLPKQEVQSDFTGLRGDNMGVKNETVDLLEELNRVSSENKKLTEMLTVVCENY

NVLRNQMMEYMSTQNGVADDSAGSRKRKAESISNPNNSNSNVNINNNNNNLDVVPGRSSESSSSDEESSCKKLRE
EHIKAKVTVVSMKTDASDTSLIVKDGYQWRKYGQKVTRDNPCPRAYFRCSFAPTCPVKKKVQRSIEDQSIVVATY
EGEHNHPMTSKPEAGGANTTSTSTGSRLNVTTIAGTTASVPCSTTLNPSGPTITLDLTAPKTVEKRDMKMNQSASPT
GGNSIHTSTGVEYQNRPEFQQFLIEQMATSLTKDPSFKAALAAAISGKILQHNNQTGRW
>CA00g87690
MEFTSLVDTSLDLSFRPRPVLDKLPKQEVQSDFTGLRGDNMGVKNETVDLLEELNRVSSENKKLTEMLTVVCENY
NVLRNQMMEYMSTQNGVADDSAGSRKRKAESISNPNNSNSNVNINNNNNNLDVVPGRSSESSSSDEESSCKKLRE
EHIKAKVTVVSMKTDASDTSLIVKDGYQWRKYGQKVTRDNPCPRAYFRCSFAPTCPVKKKVQRSIEDQSIVVATY
EGEHNHPMTSKPEAGGANTTSTSTGSRLNVTTIAGTTASVPCSTTLNPSGPTITLDLTAPKTVEKRDMKMNQSASPT
GGNSIHTSTGVEYQNRPEFQQFLIEQMATSLTKDPSFKAALAAAISGKILQHNNQTGRW

CaWRKY28

>Capana06g001506 [mRNA] locus=Chr06:35621649-35624167
MAASSFSFPTSSSFMTTSFTDLLASSDDYPITKGLGDRIAERTGSGVPKFKSLPPPSLPLSPPPFSPSSYFAIPPGLSPTE
LLDSPVLLSSSNVLPSPTTGSFPAQAFNWKSSSNNQDVKQEEKNCSDFSFQTQVGTAASISQSQTSHVSLGQQAWN
YQEPTKQDGLSSDQNANGRSEFNTMQSFMQNNDHSNSGNGYNQSIREQKRSDDGYNWRKYGQKQVKGSENPRS
YYKCTYPNCPTKKKVERSLDGQITEIVYKGNHNHPKPQATRRSSSSTASSAIQSYNTQTNEIPDHQSYGSNGTGQID
SVATPENSSISFGDDDHEHTSQKSRSRGDDLDEEEPDSKRWKRESESEGLSALGSRTVREPRVVVQTTSDIDILDDG
YRWRKYGQKVVKGNPNPRSYYKCTSPGCPVRKHVERASQDIKSVITTYEGKHNHDVPAARGSGNHSINRPIAPTIT
NNNSAMAIRPSVTSHQSNYQVPMQSIRPQQFEMRAPFTLEMLQKPNNYGFSGYANSEDSYENQLQDNNGFSRAKN
EPRDDMFMESLLC
>CA06g13580
MAASSFSFPTSSSFMTTSFTDLLASSDDYPITKGLGDRIAERTGSGVPKFKSLPPPSLPLSPPPFSPSSYFAIPPGLSPTE
LLDSPVLLSSSNVLPSPTTGSFPAQAFNWKSSSNNQDVKQEEKNCSDFSFQTQVGTAASISQSQTSHVSLVLLPPSQI
SCRFILLYMPEILIKRVSLVTLILCQAWNYQEPTKQDGLSSDQNANGRSEFNTMQSFMQNNDHSNSGNGYNQSIRE

QKRSDDGYNWRKYGQKQVKGSENPRSYYKCTYPNCPTKKKVERSLDGQITEIVYKGNHNHPKPQATRRSSSSTAS
SAIQSYNTQTNEIPDHQSYGSNGTGQIDSVATPENSSISFGDDDHEHTSQKSRSRGDDLDEEEPDSKRWKRESESEG
LSALGSRTVREPRVVVQTTSDIDILDDGYRWRKYGQKVVKGNPNPRSYYKCTSPGCPVRKHVERASQDIKSVITTY
EGKHNHDVPAARGSGNHSINRPIAPTITNNNSAMAIRPSVTSHQSNYQVPMQSIRPQQFEMRAPFTLEMLQKPNNY
GFSGYANSEDSYENQLQDNNGFSRAKNEPRDDMFMESLLC

CaWRKY29

> Capana06g002128 [mRNA] locus= Chr06:92548747..92550798
MGDTDRRTVRVAAPRMGNLELPPEDGYTWRKYGQKEILGSLPLSQIDSHIMLLNICFDLKLNITLGLNILLKFRYKT
IKVLVTIFSHVNLMKKNIFNIKIHICNLFSMIIDYYPPKWTKIRPSVLPYDRVKINHISIFVINLTDYKTFELKSNSSKQS
VTILMGCEVSPLRHECIQYYNELFLFSHSKTRKRIKHKCIRILLHNQLALLDLSRTDFSYLTKYLLAYFDQFEFHIQQ
HCWSQWDILTLISHTFKYYASNNYPLISSSCCFHIKNGSSSLSFGMNTRNFKIQNYSSISFHKDCKLIINLIFVYCKLS
HNHVTLEQRHCIYLLSLIPKKLSTHIAYTNNVIFYGNIHLLLIVLMILGLIIDAPIKSYTIVQPRSKFSVLIMILTYLKHI
DLNTFAICPPQLPPCLHHLWKRLIKPPQLLHRCCHCRHQLQPPAGIGSPWILSHKKLAQATVPLNLTYKGTNIFQNF
NGVQLQDIHFLKILKNNRSKVYLYYFIHVKYGTRHFICFCIIHLSVNSRIDPPICQYINLTANTINPCLYRDFGHASGG
SLTSICNVVTACVDGGAGPSGSRFGREVDYQPVVDMADAMFNSGSSSNTSMDIIFSSIDDKWDTTQKKE

CaWRKY30

>Capana06g003072 [mRNA] locus=Chr06:218689176-218691106
MEEIEEANNAAVENCHRVMSLLSSGTHDRNQYMNLVRETGEAVNKFKKVVTLLNSTLGHARVRKSNKFKTPLPH
NILVENPNCKIDDQAKALRRSSSGVSLNFDSSTCTPTMSSTRSFISSLSVDGSVANGSNFHLVGASQSLDQSSFQHKR
RCSERGDEGSVKCGSSGKCHCSKKRKHRVKRSIKVPAVSNKLADIPSDEYSWRKYGQKPIKGSPHPRGYYKCSSM
RGCPARKHVERCLEDPSMLIVTYEGEHNHPRMPSQSANT
>CA06g01330
MEEIEEANNAAVENCHRVMSLLSSGTHDRNQYMNLVRETGEAVNKFKKVVTLLNSTLGHARVRKSNKFKTPLPH

NILVENPNCKIDDQAKALRSRPIDENRVLEMGGTNVKCNLTLGSPSLELSSNSRNPLNFGQQTHLPSYNYLQQQQQ
QQRLFLLQQQSFISSLSVDGSVANGSNFHLVGASQSLDQSSFQHKRRCSERGDEGSVKCGSSGKCHCSKKRKHRV
KRSIKVPAVSNKLADIPSDEYSWRKYGQKPIKGSPHPRGYYKCSSMRGCPARKHVERCLEDPSMLIVTYEGEHNHP
RMASQSANT

CaWRKY31

>Capana07g000181 [mRNA] locus=Chr07:9032674-9041137
MAGVTESSKFTGTNKSSHKIENGVVDIDNGVNGAENLQSESQLSVYLAISGCGRRLSGFAVDRISVTRPGTMNAQA
RTKHQQRVPDESSTLELSSTSVAQSISSVPSPTLAESRLSAVVNCGTGEVAKQSSDAKVQPLVPVKTSNRDGYNWR
KYGQKQVKSPQGTRSYYRCTHFECCAKKIECSGHTNRVMEIIYRSEHNHDPSPSVTCSRESKSAILSASTNGKSLID
HPNRNSNETVASSFKENLQESLPIAETANLDSGGSDTDTEIDIKEEHCDEPEQKKRSRKSDASCYESVSKPGKKPKL
VVHAACDVGISSDGYRWRKYGQKMVKGNPHPRNYYRCSSAGCPVRKHIERAVDSTIALTITYKGVHDHDMPVPK
RRHGPPSAPLIAAAAAPASITDMKKPEPLQHQKSTTQWSVDKQGELTSEKLDLGGGKAMGSARTLLSIGFEIKPC
>CA07g01910
MNAQARTKHQQRVPDESSTLELSSTSVAQSISSVPSPTLAESRLSAVVNCGTGEVAKQSSDAKVQPLVPVKTSNRD
GYNWRKYGQKQVKSPQGTRSYYRCTHFECCAKKIECSGHTNRVMEIIYRSEHNHDPSPSVTCSRESKSAILSASTN
GKSLIDHPNRNSNETVASSFKENLQESLPIAETANLDSGGSDTDTEINIKEEHCDEPEQKKRSRKSDASCYESVSKPG
KKPKLVVHAACDVGISSDGYRWRKYGQKMVKGNPHPRNYYRCSSAGCPVRKHIERAVDSTIALTITYKGVHDHD
MPVPKRRHGPPSAPLIAAAAAPASITDMKKPEPLQHQKSTTQWSVDKQGELTSEKLDLGGGKAMGSARTLLSIGFE
IKPC

CaWRKY32

>Capana07g000528 [mRNA] locus=Chr07:40750931-40752123
MIEPWSRRCWHERQEYIQARSGEEVPPEDGYSWRKYGQKLIVGAKYPREHYRCDCRRLSYREATKMVQRSEAEP
LSFEVTYGGSNSCGQENKNQNGEHVVLTKETQRDEVGRAAGETPECYTPEMVSTPNTSFNNSSAGVVFSNSNPVF

NITNSDLIPTPTSSPYPDTDISLEDDSLTVLFDDVPENARSTYKCNAN
>CA05g11500
CWHERQEYIQARSGEEVPPEDGYSWRKYGQKLIVGAKYPREHYRCDCRRLSYREATKMVQRSEAEPLSFEVTYG
GSNSCGQENKNQNGEHVVLTKETQRDEVGRAAGETPECYTPEMVSTPNTSFNNSSAGVVFSNSNPVFNITNSDLIP
TPTSSPYPDTDISLEDDSLTVLFDDVPENARSTYKCNAN

CaWRKY33

>Capana07g001256 [mRNA] locus=Chr07:167387734-167411421
MADSGNSKNYGVPIYGAGWVPPSALRSAVEPPPATDDDKDDGVEKSSSSESYVVLAGGGGEGNSGIRNALIVAQF
HFDSNVLSDEPVARLGTGGDLPYRMAVHPGGDGLICSLPKSCRWFDWDIQRAENRALGLKSSERVLEPLEDVGQQ
LALAFNNDGSLLAVGGEEGKLRVYKWPSMENILDQANAHASVKDLDFSPDGKFLASVGSGPCRIWDVSISTSVAS
LMKENDEIFGYCRFSPSNDENQVLYITTMQDQGGSISKWNTTTWKRIKSKRVVRDPICAFNLSPNGKLLAIGTIEGD
VLIVSSNNLQVQNVVKKAHLGLVTTLKFSEDSRALLSASMDSRVRVTVIKEEKKSGRMGISEDDMTDEISSRKLKQ
KQDPDTVINGSESKEKGTCESMSAEVVSNELHKRPNPDALAKVSQSNRDESAHPTTCQGVLNEGQPRRNVDKEID
VSQSNQKDSSLSNVPEENSENVHQEKGPESEGGASESSRVSVLPKEEPYIKSCKSDSPVKEGNISLVVGTASDCSPQI
QSKKMEEVVSQSHQERVTYSTMAENAMYKLRPRRNPDTSVQDLPSDEGVRDLPSDQGVTPFSEPEKPFEDGYNW
RKYGQKLVRGNMFTRSYYKCTHSNCLAKKQVERSHDGHITNIQYITNHEHPKPLNSPQISPEVVVPSEMRRPDML
MGTPQAEGEKSTALGQACESIEPLESLISAAVESAGGSARDTVPKSLKSGDEGDSNGGRNSKRRKKEVPRSDDMTP
PMKSHSEPRHIVQTRSEVDILNDGYRWRKYGQKFVKGNPNPRSYYRCSSAGCPAKKHVERASHDPKLVITTYEGQ
HEHDIPLSRTVAQNSDSNTTRISGESTAESGGNKHVDN
>CA07g10930
MGISEDDMTDEISSRKLKQKQDPDTVINGSESKEKGTCESMSAEVVSNELHKRPNPDALAKVSQSNRDESAHPTTC
QGVLNEGQPRRNVDKEIDVSQSNQKDSSLSNVPEENSENVHQEKGPESEGGASESSRVSVLPKEEPYIKSCKSDSPV
KEGNISLVVGTASDCSPQIQSKKMEEVVSQSHQERVTYSTMAENAMYKLRPRRNPDTSVQDLPSDEGVRDLPSDQ
GVTPFSEPEKPFEDGYNWRKYGQKLVRGNMFTRSYYKCTHSNCLAKKQVERSHDGHITNIQYITNHEHPKPLNSP

QISPEVVVPSEMRRPDMLMGTPQAEGEKSTALGQACESIEPLESLISAAVESAGGSARDTVPKSLKSGDEGDSNGG
RNSKRRYVKVKIFFICRHCSVLQLPCDRKKEVPRSDDMTPPMKSHSEPRHIVQTRSEVDILNDGYRWRKYGQKFV
KGNPNPRSYYRCSSAGCPAKKHVERASHDPKLVITTYEGQHEHDIPLSRTVAQNSDSNTTRISGESTAESGGNKHV
DN

CaWRKY34

>Capana07g001387 [mRNA] locus=Chr07:177671908-177674692
MESSTFKNRSPPSTIQFPVNLNCSTTAIHHDHQEEEEEHNNRPVIDEMDFFADKKNGNNSEEADVTTTTNNTINHSD
RKDSNTPPPELDFNINTGLHLLTANTYSDQSIVDDGLSPNSEDKRTKSELAVLRAELERMNGENRRLRDMLNQVTS
NYSTLQMHMMTMMQQQQQNQENGQRDGKSTREEVKQQHHSHNSHGGGGGQMVPRQFMDLGLAAAGATGS
EAEEASQSSSEGRSGREKSRSPMNNMESRSTCGIGREDSPEKGSPGWGPNKIPRLGNASTNKPADQATEATMRKAR
VSVRARSEAPMITDGCQWRKYGQKMAKGNPCPRAYYRCTMAAGCPVRKQVQRCAEDRTILITTYEGTHNHPLPP
AAMAMASTTSSAARMLLSGSMPSADGLMNSNFFARTLLPCSSSMATISASAPFPTVTLDLTQSPNPLQFPRPPNQFQ
VPFSNPPHSNILANPAALLPQIFGQALYNQSKFSGLQLSQDLEGQQHPSTMSSSIHPSNHNPLADTVNALTNDPNFT
AALAAAITSLIGNPGQSNNGTNNTPATTTTANNNNGSVTSNGNNSNNGNNKVANSGFPAN
>CA07g11490
MAKGSGLSFDPDPIKHFLPIPTVLNSFLEPHHQQQQEFPHNKFLFKIEPLSSSMESSTFKNRSPPSTIQFPVNLNCSTTA
IHHDHQEEEEEHNNRPVIDEMDFFADKKNGNNSEEADVTTTTNNTINHSDRKDSNTPPPELDFNINTGLHLLTANT
YSDQSIVDDGLSPNSEDKRTKSELAVLRAELERMNGENRRLRDMLNQVTSNYSTLQMHMMTMMQQQQQNQE
NGQRDGKSTREEVKQQHHSHNSHGGGGGQMVPRQFMDLGLAAAGATGSEAEEASQSSSEGRSGREKSRSPMNN
MESRSTCGIGREDSPEKGSPGWGPNKIPRLGNASTNKPADQATEATMRKARVSVRARSEAPMITDGCQWRKYGQ
KMAKGNPCPRAYYRCTMAAGCPVRKQVQRCAEDRTILITTYEGTHNHPLPPAAMAMASTTSSAARMLLSGSMPS
ADGLMNSNFFARTLLPCSSSMATISASAPFPTVTLDLTQSPNPLQFPRPPNQFQVPFSNPPHTNILANPAALLPQIFGQ
ALYNQSKFSGLQLSQDLEGQQHPSTMSSSIHPSNHNPLADTVNALTNDPNFTAALAAAITSLIGNPGQSNNTPATTT

TANNNNGSVTSNGNNSNNGNNKVANSGFPAN

CaWRKY35

>Capana07g001809 [mRNA] locus=Chr07:204092824-204096737
MEDRLYKSSFFHKQEDSTGTPPDNAADSCFSGDEAAEVNMPSPRKRRGAKRKVISVPIIEADGSRSKGEVYPPPDS
WSWRKYGQKPIKGSPYPRGYYRCSSSKGCPARKQVERSCLDPTMLLITYCSDHNHQLPAATATKHHHTAAGGAT
SPSTSTGTAVDINPSAASDTAKKSSPEEQETNIFAGFSEFAGELGWFSDMGTSTLMESASTSATSIVGSTWNDSDVA
LMLPIREEDQSLYGDLGELPECSFVFRRYSVETTCCGGTG
>CA07g14560
MEDRLYKSSFFHKQEDSTGTPPDNAADSCFSGDEAAEVNMPSPRKSRRGAKRKVISVPIIEADGSRSKGEVYPPPDS
WSWRKYGQKPIKGSPYPRGYYRCSSSKGCPARKQVERSCLDPTMLLITYCSDHNHQLPAAAATATKHHHTAAAG
ATSPSTSTGTAVDINPSAASDTAKKSSPEEQETNIFAGFSEFAGELGWFSDMGTSTLMESASTSATSIVGSTWNDSD
VALMLAIREEDQSLYGDLGELPECSFVFRRYSVETTCCGGTG

CaWRKY36

>Capana07g001968 [mRNA] locus=Chr07:210180549-210184089
MSDNNPFHHDYAFPFFGENPSIYDHQVENTQNPHQDFDYPSSYMSSLTECLHGGSMDHYNSLSSAFGMNHRTSSS
EVVCPPPIDHHHHQELSRKNSVDHHHQIPLTPNSLISSSSNSEPGCHEEDSSKIKKDDQCEDGGDDDKSKKVNKAK
KKGEKKQKEPRFAFMTKSEIDNLEDGYRWRKYGQKAVKNSPFPRSYYRCTSQKCSVKKRVERSYQDPSIVITTYE
GQHNHHCPATLRGNAAAALLSPASFLSSSQQQLFHNPSEQQLFYNPNLPINNSFYNNYHQHQHQMQPQLGPDNYQ
YGVFQDMVASLIHKREPX
>CA07g15490
MSDNNPFHHDYAFPFFGENPSIYDHQVENTQNPHQDFDYPSSYMSSLTECLHGGSMDHYNSLSSAFGMNHRTSSS
EVVCPPPIDHHHHQELSRKNSVDHHHQIPLTPNSLISSSSNSEPGCHEEDSSKIKKDDQCEDGGDDDKSKKVNKAK
KKGEKKQKEPRFAFMTKSEIDNLEDGYRWRKYGQKAVKNSPFPRSYYRCTSQKCSVKKRVERSYQDPSIVITTYE

GQHNHHCPATLRGNAAAALLSPASFLSSSQQQLFHNPSEQQLFYNPNLPINNSFYNNYHQHQHQMQPQLGPDNYQ
YGVFQDMVASLIHKREP

CaWRKY37

>Capana07g002350 [mRNA] locus=Chr07:219055519-219061043
MANSHAEQLTNGRTSASSAAAPGGSNGGGAVAKYKLMTPAMLPISRSTCITIPPGLSPSSFLESPLLLSNIKAEPSPT
TGSFSKFQTVQGSGGAAAFLLTRGYSSSNSYIERKSSCFEFKNASGSSSTSGSFATEHVISTGFNQQQNDPLKEVQD
QSHRQLLVPSSLAKLEMESSKELSISAPVNVDASSKEESLCQPINVDAMNPGGPSNASMQGSHADHKDVSSVTSER
SSDDGYNWRKYGQKLVKGSEFPRSYYKCTYPNCEVKKIFERSPEGQITEIVYKGSHDHPKPQLCRRFSPGSLVSIQE
DKCEKEACFRGQEVYVEDKFNTNVQTNKIEPGSTPVSPQTDTDALEGAASQMQGTNDDMDEDDQFAKRRKMDG
GMDVTPVIKPIREPRVVVQTVSEVDILDDGYKWRKYGQKVVRGNPNPRSYYKCTNAGCPVRKHVERASHDPKAV
ITTYEGKHNHDVPTARNNNHEMTGSTPVTGGSRIRAEQTNSLSLDLGVGTGYHLDNGNNGQLHTLHNQVQVSRS
GMMLVQPGAVVARYGIVHNGMSRFGAIDNRVQGPSFETLPLQPSTQSLQSYGKILLGP
>CA07g20160
MANSHAEQLTNGRTSASSXXXNGGGAVAKYKLMTPAMLPISRSTCITIPPGLSPSSFLESPLLLSNIKAEPSPTTGSFS
KFQTVQGSGGAAAFLLTRGYSSSNSYIERKSSCFEFKNASGSSSTSGSFATEHVISTGFNQQQNDPLKEVQDQSHRQ
LLVPSSLAKLEMESSKELSISAPVNVDASSKEESLCQPINVDAMNPGGPSNASMQGSHADHKDVSSVTSERSSDDG
YNWRKYGQKLVKGSEFPRSYYKCTYPNCEVKKIFERSPEGQITEIVYKGSHDHPKPQLCRRFSPGSLVSIQEDKCEK
EACFRGQEVNLYRFSVYVEDKFNTNVQTNKIEPGSTPVSPQTDTDALEGAASQMQGTNDDMDEDDQFAKRSRKM
DGGMDVTPVIKPIREPRVVVQTVSEVDILDDGYKWRKYGQKVVRGNPNPRSYYKCTNAGCPVRKHVERASHDPK
AVITTYEGKHNHDVPTARNNNHEMTGSTPVTGGSRIRAEQTNSLSLDLGVGTGYHLDNGNNGQLHTLHNQVQVS
RSGMMLVQPGAVVARYGIVHNGMSRFGAIDNRVQGPSFETLPLQPSTQSLQSYGKILLGP

CaWRKY38

>Capana07g002454 [mRNA] locus=Chr07:220545442-220548961
MGGFDDHVAIMGDWMPPSPSPRTFFSSLLGDDVGSRSTFQCTNETKSGNLASGPQENVGTFDGNDEAQAAVSEQQ
PVSDQKMNPRGGLLERMAARAGFNAPKLNTESLRPADMRQNQGVQNQGVRSPYLTIPPGLSPTTLLESPVFLSNSL
VQPSPTTGKFPFSSGIESRNSTLMMEDPDNRKENAFESINASSFSFKPVPETAPSLFPGTNSRVNPPNFSQQGFPNIEV
SVHSQNSLQSHRMEATQNLVQNGTLNQASDFPRFSAEMDVKGSNVTPESRTFQTVGSTVEHSPPLDEPQDEDIDQR
GGGDPNVVGAPAEDGYNWRKYGQKQVKGSEYPRSYYKCTHPNCPVKKKVERSQEGHITEIIYKGAHNHPKPPPN
RRSALGSTNSLGDLRLDGVEQGASGVNGDLGRANIQKAPGSGGGFDWRSNNLDATSSVNLGSEYCNRSAPFPAQ
NDSQLESGDAVDVSSTFSNDEDEDDRGTHGSISQGYDGEGDESESKRRKLETYSADMTGATRAIREPRVVVQTTSE
VDILDDGYRWRKYGQKVVKGNPNPRSYYKCTSAGCNVRKHVERASHDLKSVITTYEGKHNHDVPAARNSSHVN
SGASNTHPTAVTAPAQNHLHRPEPAQLQNAMARFDRQPSLGSFGLAGRPQLGPTPGFSYGMNPQGLSSLSMAGFH
PNQNKPGEVPIHPYLGQPRPMHDMGFMFPKEEPKVEPMSDPGLNLSNGSNVYQQFMNRLPLGPQM
>CA07g21030
MGGFDDHVAIMGDWMPPSPSPRTFFSSLLGDDVGSRSTFQCTNETKSGNLASGPQENVGTFDGNDEAQAAVSEQQ
PVSDQKMNPRGGLLERMAARAGFNAPKLNTESLRPADMRQNQGVQNQGVRSPYLTIPPGLSPTTLLESPVFLSNSL
VQPSPTTGKFPFSSGIESRNSTLMMEDPDNRKENAFESINASSFSFKPVPETAPSLFPGTNSRSWLQVNPPNFSQQGF
PNIEVSVHSQNSLQSHRMEATQNLVQNGTLNQASDFPRFSAEMDVKGSNVTPESRTFQTVGSTVEHSPPLDEPQDE
DIDQRGGGDPNVVGAPAEDGYNWRKYGQKQVKGSEYPRSYYKCTHPNCPVKKKVERSQEGHITEIIYKGAHNHP
KPPPNRRSALGSTNSLGDLRLDGVEQGASGVNGDLGRANIQKAPGSGGGFDWRSNNLDATSSVNLGSEYCNRSAP
FPAQNDSQLESGDAVDVSSTFSNDEDEDDRGTHGSISQGYDGEGDESESKRRKLETYSADMTGATRAIREPRVVV
QTTSEVDILDDGYRWRKYGQKVVKGNPNPRSYYKCTSAGCNVRKHVERASHDLKSVITTYEGKHNHDVPAARNS
SHVNSGASNTHPTAVTAPAQNHLHRPEPAQLQNAMARFDRQPSLGSFGLAGRPQLGPTPGFSYGMNPQGLSSLSM
AGFHPNQNKPGEVPIHPYLGQPRPMHDMGFMFPKEEPKVEPMSDPGLNLSNGSNVYQQFMNRLPLGPQM

CaWRKY39

>Capana08g000429 [mRNA] locus=Chr08:60198759-60200111
MAANNPSANMLDGSFRSLDSPDSDDFSNHLINFELSDILEIDNWPIQQDPTLIPQYSNYAANQVVNTSSYQEEPSNNI
GSSSSKRKEVKDKVAFRTLSQIEILDDGYKWRKYGKKMVKNSPNPRNYYRCSVEGCPVKKRVERDKEDSRYVITT
YEGVHNHQGLSPF
>CA08g03020
MAANNPSANMLDGSFRSLDSPDSDDFSNHLINFELSDILEIDNWPIQQDPTLIPQYSNYAANQVVNTSSYQEEPSNNI
GSSSSKRKEVKDKVAFRMLSQIEILDDGYKWRKYGKKMVKNSPNPRNYYRCSVEGCPVKKRVERDKEDSRYVIT
TYEGVHNHQGLSPF

CaWRKY40

>Capana08g000683 [mRNA] locus=Chr08:101219429-101222082
MDTNLGDKTFSIDLNTNPSLHNTSRSPHDTLDEKLVRMREENKKLVTMLTTLCENYNSLHSHLIELLQKYFSHNEE
DNFKFFLRKRKAEGECCENNSDIHFEEASPKRPREITTNVSTVCVKTNPSDQTSLVKDGYNWRKYGQKVTRDNPY
PRAYYKCSFAPTCPVKKKVQRSIEDPSILVAVYEGEHNHPHPSQAEITVPLLNQGVTTDPTFLNKLMEEIDTNSLQQ
HLVEQMASSLTSSPSFTAAVAAAISGKIFEYDLPFK
>CA00g00130
MDTNLGDKTFSIDLNTNPSLHNTSRSPHDTLDEKLVRMREENKKLVTMLTTLCENYNSLHSHLIELLQKYFSHNEE
DNFKFFLRKRKAEGECCENNSDIHFEEASPKRPREITTNVSTVCVKTNPSDQTSLVKDGYNWRKYGQKVTRDNPY
PRAYYKCSFAPTCPVKKKVQRSIEDPSILVAVYEGEHNHPHPSQAEITVPLLNQGVTTDPTFLNKLMEEIDTNSLQQ
HLVEQMASSLTSSPSFTAAVAAAISGKIFEYDLPFK

CaWRKY41

>Capana08g001012 [mRNA] locus=Chr08:123573760-123575138
MEEDWDLHAVVRGCTASSTTTTTSTTTTATSCCSFQPRQDGNFFSFQDPFVPRFDNPTSDFEELHNLYKPFFPKSQV
QPQQQVPRSPQNNIIPISPLSVLGGLQDLSAPQPTLKQQQQQQQQQQHIHQFFNSTRLTQPKQSLSVNGSTNSTITA
SSSLGVSHTQSPRPKRRKNQLKKVCQVPAEGLSSDMWSWRKYGQKPIKGSPYPRGYYRCSTSKGCLARKQVERN
RSDPNMFIVTYTAEHNHPMPTHRNSLAGSTRQKPANSEAGTTASDSNKPTSSSPVSSPACHSTATEKQESSREEKED
IFEDEDEEFGSSNMGLDNMEPADDDFFEGLDELAAQATGDCFSDNFQGSMQLPWLSNNATTTAAGGV
>CA08g07730
MEEDWDLHAVVRGCTASSTTTTTSTTTTATSCCSFQPRQDGNFFSFQDPFVPRFDNPTSDFEELHNLYKPFFPKSQV
QPQQQVPRSPQNNIIPISPLSVLGGLQDLSAPQPTLKQQQQQQQQQQHIHQFFNSTRLTQPKQSLSVNGSTNSTITA
SSSLGVSHTQSPRPKRRKNQLKKVCQVPAEGLSSDMWSWRKYGQKPIKGSPYPRGYYRCSTSKGCLARKQVERN
RSDPNMFIVTYTAEHNHPMPTHRNSLAGSTRQKPANSEAGTTASDSNKPTSSSPVSSPACHSTATEKQESSREEKED
IFEDEDEEFGSSNMGLDNMEPADDDFFEGLDELAAQATGDCFSDNFQGSMQLPWLSNNATTTAAGGV

CaWRKY42

>Capana08g001044 [mRNA] locus=Chr08:124129873-124131797
MEKVKGLEKKKLISELTQGKEFVKQLKKQIGPLASPEECDLLLGKILSSLEKSLSILNLKALLLEGGINANNSTSSCS
SISFLGNNNSPMSEVFDSPSHHLDKNMVSKKRKKSQETNQITISGTGLEGSHEDGFSWRKYGQKDILGANHPRAYY
RCTHRHTQGCLATKQVQRSDGNSTIFEVTYKGRHSCKVAQSDIFSLNNQKRQKHNKKQEQEMVIFNSTPNHDAEN
FNITTKEEVFTPFSFPPTPLNLENIEETKFFCDSMVPLLTSQNQEFGMDYMTLHCSNSDLTELISTPTPTSISNSSFVGD
WDLSEDFEPNVIFDIEEFFS
>CA08g08240
MEKVKGLEKKKLISELTQGKEFVKQLKKQIGPLASPEECDLLLGKILSSLEKSLSILNLKALLLEGGINANNSTSSCS
SISFLGNNNSPMSEVFDSPSHHLDKNMVSKKRKKSQETNQITISGTGLEGSHEDGFSWRKYGQKDILGANHPRAYY
RCTHRHTQGCLATKQVQRSDGNSTIFEVTYKGRHSCKVAQSDIFSLNNQKRQKHNKKQEQEMVIFNSTPNHDAEN

FNITTKEEVFTPFSFPPTPLNLENIEETKFFCDSMVPLLTSQNQEFGMDYMTLHCSNSDLTELISTPTPTSISNSSFVGD
WDLSEDFEPNVIFDIEEFFS

CaWRKY43

>Capana08g001961 [mRNA] locus=Chr08:139093292-139095397
MENNNKSESDNEMEIDLRLKLDAREEENEENKIGEPSQLTEKTQAKDQEIPKNDQELSMLEKEMKRMKEENKVLR
MAVEQTMKDYYDLQAKFSTIHQNNHKDHKKFLSLSGNDDSTTSEGLTTRVPKILDIINTTNRTSSPTSHEDDTMDG
DQLGLSLTLVSSNSTTSSKLLEMLEEDQRKEKKEDHPTITHQIQNNKSQNLGGLTSHHVTTASPPNRKSRVSVRAR
CESATMNDGCQWRKYGQKIAKGSPNCPRAYYRCTVAPGCPVRKQVQRCLEDMSILITTYEGTHNHPLPVGATAM
ASTASAAASFMLVDSSISPLLNNPNSSLNQPLNFPNYHHNLAPNYHHNIPNSSSSSSLIPYNLSMIRNNILNSSDPNSQ
GNIVLDLTKNISNNHQFPFASSSSNSHEMGHSNWMPKLPNYEGNSLLAGPKLQGEHHYSHNNNNIPPTLAHDENM
SAIAADPKFRVAVAAAISSLINKDQSHSTGESNGGSINRHTDS
>CA00g60490
MENNNKSESDNEMEIDLRLKLDAREEENEENKIGEPSQLTEKTQAKDQEIPKNDQELSMLEKEMKRMKEENKVLR
MAVEQTMKDYYDLQAKFSAIHQNNHKDHKNFLSLSGNDDSTTSEGLTTRVPKILDIINTTNRTSSPTSHEDDTMDG
DQLGLSLTLVSSNSTTSSKLLEMLEEDQRKEKKEDHPTITHQIQNNKSQNLGGLTSHHVTTASPPNRKSRVSVRAR
CESATMNDGCQWRKYGQKIAKGSPNCPRAYYRCTVAPGCPVRKQVQRCLEDMSILITTYEGTHNHPLPVGATAM
ASTASAAASFMLVDSSISPLLNNPNSSLNQPLNFPNYHHNLAPNYHHNIPNSSSSSSLIPYNLSMIRNNILNSSDPNSQ
GNIVLDLTKNISNNHQFPFASSSSNSHEMGHSNWMPKLPNYEGNSLLAGPKLQGEHHYSHNNNNIPPTLAHDENM
SAIAADPKFRVAVAAAISSLINKDQSHSTGESNGGSINRHTDS

CaWRKY44

>Capana09g000676 [mRNA] locus=Chr09:32179774-32181439

MGDQTSVEVPPTPNSSISSTSNEAGGQEDSSKIKKHMQNKDGQEGRDDKSKKECKATKKGEKKVKEPRFAFMTKS
EIDNLEDGYRWRKYGQKAVKNSPFPRNYYRCTTQKCSVKKRVERSYEDASIVITTYEGQHNHHCPAALRGNASFL
SSPHFMPSFPPQLFSQMLIPPTSNQNLLITSEAYNNINNNNYHQQNQGSEYNLFGGGTNDASWIQKQEPS
>CA09g08120
MSDNPFYHDYMGTSGGINTFPFFGENPSNYHDQPIIPNIQNPNHEHQFVPSSYMTLTECLHGSMDYNTLSSVFGMS
CSSSSEVVCPHIDNQGSTRKSSVSATAEPILDSPMGDQTSVEVPPTPNSSISSTSNEAGGQEDSSKIKKHMQNKDGQE
GRDDKSKKECKATKKGEKKVKEPRFAFMTKSEIDNLEDGYRWRKYGQKAVKNSPFPRNYYRCTTQKCSVKKRV
ERSYEDASIVITTYEGQHNHHCPAALRGNASFLSSPHFMPSFPPQLFSQMLIPPTSNQNLLITSEAYNNINNNNYHQQ
NQGSEYNLFGGGTNDASWIQKQEPS

CaWRKY45

>Capana09g001251 [mRNA] locus=Chr09:129708552-129712643
MASSGGNTNTFMNSFNSNYSFSSSQFMTSSFSDLLSDNNDDNNNNRNWGFSYQRIMNSINKDEVPKFKSFPPSSLP
MISSSSPASPSSYLAFPHSLSPSVLLDSPVLFNNSNTLPSPTTGSFGSLNSKEDNSRTSDFSFHSRPATSSSIFHSSAPRN
SLDDLITRQQQTTEFSTAKIGVKSEVAPIQSFSQENMQNNPAPMHYCQPSQYVREQKAEDGYNWRKYGQKQVKG
SENPRSYYKCTFPNCPTKKKVERNLDGHVTEIVYKGSHNHPKPQSTRRSSAQSIQNLAYSNLDITNQPNAFLENAQ
RDSLAVTDNSSASFGDEDVDQGSPISKSGENDENEPEAKRWKGDNENEVISSASRTVREPRIVVQTTSDIDILDDGY
RWRKYGQKVVKGNPNPRSYYKCTFIGCPVRKHVERASHDLRAVITTYEGKHNHDVPAARGSGSYSMNKPPSGSN
NNMPVVPRPSLLANNSNQGMNVSNTLFNTAQVEPPITLQMLQSSGSSSYSGFGTSSGSYMNQMQPTNNSKLISKEE
PKDDLFFSSFLN
>CA09g11930
MTSSFSDLLSDNNDDNNNNRNWGFSYQRIMNSINKDEVPKFKSFPPSSLPMISSSSPASPSSYLAFPHSLSPSVLLDSP
VLFNNSNTLPSPTTGSFGSLNSKEDNSRTSDFSFHSRPATSSSIFHSSAPRNSLV
>CA09g11940

MKQDDLITRQQQTTEFSTAKIGVKSEVAPIQSFSQENMQNNPAPMHYCQPSQYVREQKAEDGYNWRKYGQKQVK
GSENPRSYYKCTFPNCPTKKKVERNLDGHVTEIVYKGSHNHPKPQSTRRSSAQSIQNLAYSNLDITNQPNAFLENA
QRDSLAVTDNSSASFGDEDVDQGSPISKSGENDENEPEAKRW
>CA09g11950
MFFRVNCPFRKGDNENEVISSASRTVREPRIVVQTTSDIDILDDGYRWRKYGQKVVKGNPNPRSYYKCTFIGCPVR
KHVERASHDLRAVITTYEGKHNHDVPAARGSGSYSMNKPPSGSNNNMPVVPRPSLLANNSNQGMNVSNTLFNTA
QVEPPITLQMLQSSGSSSYSGFGTSSGSYMNQMQPTNNSKLISKEEPKDDLFFSSFLN

CaWRKY46

>Capana09g001790 [mRNA] locus=Chr09:205298779-205300747
MEEEGLIKNSWSYEDELIKELLDDESPFLLAPHEEYYSTSSSETSYSLDVTKSSISSLSKGSFIDDIESDVSMTRNGVQ
SHDVSHDARNIGLERGLNLMMNKQEALENKYTLRIKTCGNAMADDGYKWRKYGQKSIKNSPYPRSYYKCTNPR
CGAKKQVERSSNEPDTFIITYEGLHLHFAYPFITLNPPQFLDQPTKKPKLTNPKAQNNEENASEVDESPKFVNPSPIV
DLEDGLGFGEMGSQGLLEDMVPLMIRNPFIKPTNSYSSSCSYSSPPTSPSFSWSNN
>CA09g05110MNKQEALENKYTLRIKTCGNAMADDGYKWRKYGQKSIKNSPYPRSYYKCTNPRCGAKKQVERSS
NEPDTFIITYEGLHLHFAYPFITLNPPQFLDQPTKKPKLTNPKAQNNEENASEIDESPKFVNPSPIVDLEDGLGFGEM
GSQGLLEDMVPLMIRNPFIKPTNSYSSSCSYSSPPTSPSFSWSNN

CaWRKY47

>Capana10g000205 [mRNA] locus=Chr10:3656310-3659300
MGGFDDHVAIFGDWITPSPSPRAFVSSLLVDDVGGWLPLMEHTNESNCRNFNAEPQQNVTALCSTDGKDGARAG
ASTDQTVKSSAPSEQKPSTRGGGLMERMVARSGFHAPRLNTDGLRPPVLSQNQEARSPYLTIPPGLSPSVLLYSPVL
IYNPLVCPSPTTGLLPLASGDESKSLMLTAGIADKRKETAFGSNTSSSFSSNPVNPSSDLSQQLLPQIEVSAHPNNSLQ

PQSMEVTQSEQIRHGISKFPMLSTEEDFRGSHIKPEVRPFNIVGGSMQHSQTLDEQKDEDTKQRGGGDSKDVNPPA
EDGYNWRKYGQNQANGRTYPRSYYKCAYPKCPVKKRVGGYHDCQVMEIIYKGIHNHPKPLSNPISALGSLNSFG
DVQLDNVDPSGTGFNSELALATSQQGPTAKGLMWSNNKLEATSLAALHSEYCSGSTTLQSKGAQQGSADAVEVS
SVFSNDEDDHGTRGSVLLGYDGAEDEFEPKRRSYYKCTSSGCNVRKHIQRSPYDQKSVITTYDGKHYHEVPPARTS
SQGSSGASKSPSNPITTDAQSHVGRPEPTQVQNTKPAQVQNTKSTQVQNTNERYGRAPQIQNTNERYGRAPQVQN
TNERYGRVPSLGSAGPISGFDCFGTNEQQDLSSLAMAGFNSNQHQFSVPLNPYIGWQRPVNDVGFVLPEGEAMPD
PNLNYSNGSSTYQKIMNGLPPQM

>CA10g00950

MGGFDDHVAIFGDWITPSPSPRAFVSSLLVDDVGGWLPLMEHTNESNCRNFNAEPQQNVTALCSTDGKDGARAG
ASTDQTVKSSAPSEQKPSTRGGGLMERMVARSGFHAPRLNTDGLRPPVLSQNQEARSPYLTIPPGLSPSVLLYSPVL
IYNPLVCPSPTTGLLPLASGDESKSLMLTAGIADKRKETAFGSNTSSSFSSNPVTTTLSQTHTYTYTHIACQFPAGFH
DLSQQLLPQIEVSAHPNNSLQPQSMEVTQSEQIRHGISKFPMLSTEEDFRGSHIKPEVRPFNIVGGSMQHSQTLDEQK
DEDTKQRGGGDSKDVNPPAEDGYNWRKYGQNQANGRTYPRSYYKCAYPKCPVKKRVGGYHDCQVMEIIYKGIH
NHPKPLSNPISALGSLNSFGDVQLDNVDPSGTGFNSELALATSQQGPTAKGLMWSNNKLEATSLAALHSEYCSGST
TLQSKGAQQGSADAVEVSSVFSNDEDDHGTRGSVLLGYDGAEDEFEPKRRKSTVSDTSGTIRAIREPRVVVHTISE
VDIIDDGYRWRKYGQKVVKGNPNPRSYYKCTSSGCNVRKHIQRSPYDQKSVITTYDGKHYHEVPPARTSSQGSSG
ASKSPSNPITTDAQSHVGRPEPTQVQNTKPAQVQNTKSTQVQNTNERYGRAPQVQNTNERYGRAPQVQNTNERY
GRVPSLGSAGPISGFDSFGTNEQQDLSSLNQHQFSVPLNPYIGWQRPVNDAGFVLPEGEAMPDPNLNYSNGSSTYQ
KIMNGLPPQM

CaWRKY48

>Capana10g000754 [mRNA] locus=Chr10:46615318-46617494

MEEDWDLHAVVRSCTPANTASTTTNDCDLVHGILKVDGALMKSLRIEKDYRQFDNDQMTLQRGSTTTSCNPTNN
IHYLCSFQPRPNDNNNNSLFCFKDLLEQRILTTNSATDFEESHELCKPFFTASESLTISSPRRGLPISPISVLGRLQDLPP

SSQQQQQQHLHQLTNTKPIQPKRPLSSLNGSITNCTLHAQSSRTKRRKNQLKKVCQVAADALSSDMWSWRKYGQ
KPIKGSPYPRGYYKCSTSKACLARKQVERNRSDPNMFIVTYTAEHNHPMPTHRNSLAGISRHKTANPNKPTSLSPA
TNSPAPENQESSRDDKEDIFEDDDDEFGKTEPDDDFFDGLDELVIQATGDSLPEKFSGTLQFPWLVNNAATTAAGG
G
>CA10g06160
MEEDWDLHAVVRSCTPANTASTTTNDCDLVHGILKVDGALMKSLRIEKDYRQFDNDQMTLQRGSTTTSCNPTNN
IHYLCSFQPRPNDNNNNSLFCFKDLLEQRILTTNSATDFEESHELCKPFFTASESLTISSPRRGLPISPISVLGRLQDLPP
SSQQQQQQHLHQLTNTKPIQPKRPLSSLNGSITNCTLHAQSSRTKRRKNQLKKVCQVAADALSSDMWSWRKYGQ
KPIKGSPYPRGYYKCSTSKACLARKQVERNRSDPNMFIVTYTAEHNHPMPTHRNSLAGISRHKTANPNKPTSLSPA
TNSPAPENQESSRDDKEDIFEDDDDEFGKTEPDDDFFDGLDELVIQATGDSLPEKFSGTLQFPWLVNNAAXXXWLT

CaWRKY49

>Capana10g001220 [mRNA] locus=Chr10:128628965-128629689
MEFNYWWYNYVCVSNQCSPNIVRRKTSATTIKEASTLVDDGHVWRKYGQKEILNFPHPRNYYRCTHKFDRGCEA
TKQVQRIQENPPKFRTTYQGHHSCTTYPSISQILFDSSTNEDCSVLLSFNTNKINYQQPYIHSFHSTKQETKGEISSNC
FSPNVGQSQSDDHLIRAALSPGSSDHDVNYSSCTTNCSLEMEMKIDMMVDSVDFEDLIPFDF>CA10g06890MKFN
YWWYNYVCVSNQCSPNIVRRKTSATTIKEASTLVDDGHVWRKYGQKEILNFPHPRNYYRCTHKFDQGCEATKQV
QRIQENPPKFRTTYQGHHSCTTYPSISQILFDSSTNEDCSVLLSFNTNKINYHQPYIHSFHSTKQETKGEISSNCFSPNV
GQSQSDDHLIRAALSPGSSDHDVNYSSCTTNCSLEMEMKIDMMVDSVDFEDLIPFDF
>CA10g06890
MKFNYWWYNYVCVSNQCSPNIVRRKTSATTIKEASTLVDDGHVWRKYGQKEILNFPHPRNYYRCTHKFDQGCEA
TKQVQRIQENPPKFRTTYQGHHSCTTYPSISQILFDSSTNEDCSVLLSFNTNKINYHQPYIHSFHSTKQETKGEISSNC
FSPNVGQSQSDDHLIRAALSPGSSDHDVNYSSCTTNCSLEMEMKIDMMVDSVDFEDLIPFDF*

CaWRKY50

>Capana10g001548 [mRNA] locus=Chr10:164830295-164833345
MESFLLQNTISDLEKVMEELNRGKKFTRRLREIIKKPKINVGNEDAYMSSAEDLVGKIMNSFCASLSILSSEESTEEV
SQKSMEDSSGSCKTSSLKDRRGCYKRRRTLETSIKETS
TLVDDGHGWRKYGQKQILNAKFPRNYFRCTHKFDQGCQASKQVQRIQENPPLFRITYYGHHTCKTFPKVSQMIFD
SPNDHEDSNSVLLNFNSGNNHHQFLDMTVETLDFVDLSFEF

CaWRKY51

>Capana10g001791 [mRNA] locus=Chr10:183798726-183801612
MEVNEAVKIPIARPVASRPRCPVYKSFSELLTGTVDISSTNVHSEMAVTAIRPKTIRLKPATNHALVGEPSSQVGVS
KAPVGFGSDNILQSVEKPKVLYKPIGKLAQRKTIPLLEN
KGSSVSDQQRVIADSEAHVQSANEVKQQHDPTTESKQSLSEKSGQDKKKVRSTIVSGSTEEVAQSLINTSNVDRPS
YDGYNWRKYGQKKVKGSEYPRSYYKCTHLKCPVKKKVERS
YDGQITEIVYRGDHNHPKPQPPKRNLSDGHRQTAICNDTSKETNNPAWVRNSEDSAVGSKKLKATCDEQKSKRRK
IKGPSSGAGTSGESTFPYIPNQSTTDSEITEDGFRWRKYGH
KVVKGSSYPRSYYRCTSPKCSVRKFVERTTDDPRAFITTYEGKHNHGVPNRRPNSEASKTSSKSSAMKEKL
>CA10g14950
MEVNEAVKIPIARPVASRPRCPVYKSFSELLTGTVDISSTNVHSEMAVTAIRPKTIRLKPATNHALVGEPSSQVGVS
KAPVGFGSDNILQSVEKPKVLYKPIGKLAQRKTIPLLEN
KGSSVSDQQRVIADSEAHVQSANEVKQQHDPTTESKQSLSEKSGQDKKKVRSTIVSGSTEEVAQSLINTSNVDRPS
YDGYNWRKYGQKKVKGSEYPRSYYKCTHLKCPVKKKVERS
YDGQITEIVYRGEHNHPKPQPPKRNLSDGHRQTAICNDTSKETNNPAWGNQHPQMSEAYVCRIENQNDRGLTIHSS
KVPCFYDPIVAAGMHTAVRNSEDSAVGSKKLKATCDEQKS
KRRKIKGPSSGAGTSGESTFPYIPNQSTTDSEITEDGFRWRKYGHKVVKGSSYPRSYYRCTSPKCSVRKFVERTTDD
PRAFITTYEGKHNHGVPNRRPNSEASKTSSKSSAMKEKL

CaWRKY52

>Capana10g001805 [mRNA] locus=Chr10:185896489-185897886
MEVNEAAKLPIARPVASRPRCPLYKSFSELLTGAVDISSTNVHSEMAITAIRPKTIRLKPATNHALVGEPSSQVGVSE
APVGFGADNILQSVEKPKVLYKPIAKLAPKKTIPLLENKGSSVSDQQRVIADAEAHIQSANEVKQQHDPTTESKQSL
SAKSGQDKKKVRSTIVSGSTEEVAQSLINTSNVDRPSYDGYNWRKYGQKQVKGSEYPRSYYKCTHLKCLVKKKV
ERSYDGQITEIVYRGEHNHPKPQPPKRNLSDGHRRTAICNDTSKETNNPAWSNQHPQMSEAYVCRRENQNDGGLT
IHSSKVPCFYDPIVAAGMHTAVRTLQSEILKILL
>CA10g14770
MEVNEAAKLPIARPVASRPRCPLYKSFSELLTGAVDISSTNVHSEMAITTIRPKTIRLKPATNHALVGEPSSQVGVSE
APVGFGADNILQSVKKPKVLYKPIAKLAPKKTIPLLENKGSSVSDQQRVIADAEAHIQSANEVKQQHDPTTESKQSL
SAKSGQDKKKVRSTIVSGSTEEVAQSLINTSNVDRPSYDGYNWRKYGQKQVKGSEYPRSYYKCTHLKCLVKKKV
ERSYDGQITEIVYRGEHNHPKPQPPKRNLSDGHRRTAICNDTSKETNNPAWSNQHPQMSEAYVCRRENQNDGGLT
IHSSKVPCFYDPIVAAGMHTAVRNSEDSAEGSKKLKATCDEQKSKRRKIKCPSSGAGTSGESTFPYIPNQSTTDSEIT
EDGFRWRKYGQKVVKGSSYPRRYYRCISPKCNVWKFVERTTDDPRAFITTYEGKHNHGVPNRRPNSEASKTSSKS
SAMKEKL

CaWRKY53

>Capana11g001882 [mRNA] locus=Chr11:200570029-200575124
MAENEGLSSSSATSRGQLVRPTITLPPRNSMDSLFSGGISPGPMTLVSSFFSDNDPDSECRSFSQLLAGAMTSPAGIS
GVRPGFSPPSTAAPAMTPTFSVPPGLNPTNLFDGFFSPGQGPYGMSHQQVLAQLTAQASQPQSQMHIQPDYSSSSA
ATALSMSPFQSLTSNTAANQQIPPALDPNIMKESSDVSLSDQRSEPASFVVDKPADDGYNWRKYGQKQVKGSEYP
RSYYKCTQPNCPVKKKVERSLDGQVTEIIYKGQHNHQPPQASKRSKESGNPNGNYNLQGTYEPKEGEPSYSLRMK

DQESSLANDQISGSSDSEEVGNAETRVDGRDIDERESKRRAVEVQTSEAVCSHRTVAEPRIIVQTTSEVDLLDDGYR
WRKYGQKVVKGNPYPRSYYKCTSQGCNVRKHVERAASDPKAVITTYEGKHNHDVPAARNSSHNTANNSTSQLR
PHNPVFDKPTAMRRSDFPSNEQQPIALLRFKEEQIT
>CA11g03750
MAENEGLSSSSATSRGQLVRPTITLPPRNSMDSLFSGGISPGPMTLVSSFFSDNDPDSECRSFSQLLAGAMTSPAGIS
ACDHVKDNLGCYSFYGLRFANPFNVCHLNRYKLRLSCTYHFSDIYNSDLCIFICQGPFGMSHQQVLAQLTAQASQP
QSQMHIQPDYSSSSAATALSMSPFQSLTSNTAANQQIPPALDPNTIKESSDVSLSDQRSEPASFVVDKPADDGYNWR
KYGQKQVKGSEYPRSYYKCTQPNCPVKKKVERSLDGQVTEIIYKGQHNHQPPQASKRSKESGNPNGNYNLQGTY
EPKEGEPSYSLRMKDQESSLANDQISGSSDSEEVGNAETRVDGRDIDERESKRRAVEVQTSEAVCSHRTVAEPRIIV
QTTSEVDLLDDGYRWRKYGQKVVKGNPYPRSYYKCTSQGCNVRKHVERAASDPKAVITTYEGKHNHDVPAARN
SSHNTANNSTSQLRPHNPVFDKPTAMRRSDFPSNEQQPIALLRFKEEQIT

CaWRKY54

>Capana11g001905 [mRNA] locus=Chr11:201981442-201987021
MDEKDKVDDQLPIESSWSQLNPDDDADHAYFFENNINTNNDTSSILSEFGWNFQPVEENSSSRFDKIDEHLAGNSSI
TTMSTSPASATAATELTTAKISTDEPVSSSCSDDPPEKSTASGGSSASKPPSDTVSKVKKKGQKRIRQPRFAFMTKSE
VDHLEDGYRWRKYGQKAVKNSPFPRSYYRCTNTKCTVKKRVERSSEDSSIVITTYEGQHCHHTVGFPRGGLINHE
AAFTSQLSPLPSQYYHPSGVQYPHELVPMTAAAPVESRTMPGETGSSEARRLPETSQPAATDEGLLGDIVPPGMRS
K
>CA10g13480
MTKSEVDHLEDGYRWRKYGQKAVKNSPFPRSYYRCTNTKCTVKKRVERSSEDSSIVITTYEGQHCHHTVGFPRGG
LINHEAAFTSQLSPLPSQYYHPSGVQYPHELVPMTAAAPVESRTMPGETGSSEARRLPETSQPAATDEGLLGDIVPP
GMRSKCaWRKY47
>Capang05g001761

NLSTFVMNNPNMMSNNFDIEKDYSLTFLLENMFGGTHVDHQDYDFITTTTSNSIFDLLMLPPHQPIITTSTVQDSTIS
DQLINAPVTPNVSSISSTSTELPADDYQQEKKVNQQDGEQDQDKYKKQLKPKRKNQKGKREPRFAFMTKSEIDHL
DDGYKWRKYGQKAVKNSPFPRSYYRCTTTSCGVKKRVERSIQDTSIVVTTYEGTHTHSCPVMPRGYAGVHPVTIN
YGVSIGAGDSGGRTYYGDSSLFSTNNLLQERRF>CA05g16170MFGGTHVDHQDYDFITTTTSNSIFDLLMLPPHQPI
ITTSTVQDSTISDQLINAPVTPNVSSISSTSTELPADDYQQEKKVNQQDGEQDQDKYKKQLKPKRKNQKGKREPRF
AFMTKSEIDHLDDGYKWRKYGQKAVKNSPFPRSYYRCTTTSCGVKKRVERSIQDTSIVVTTYEGTHTHSCPVMPR
GYAGVHPVTINYGVSIGAGDSGGRTYYGDSSLFSTNNLLQERRF

CaWRKY55

>Capana12g001134 [mRNA] locus=Chr12:48289811-48291121
MLEEELKRMKEENKKLATMLTTLGENYNSLRTNLIELQQKHSTHEEDNNSKLLSRKRKAEDVCCVNNSDINFEEA
SPKRPREIITTSISTVSVKTTLSDQTSWVKDGYNWRKYGQKVTRDNPSPRAYYKCSFAPTCPVKKKVQRSVKDPSV
LVATYEGEHNHPHPSQAETTAPLVNQGVITNPTFFNKFMEDINTSSLQKDLVAKMVPSLSKNPSFAATVAQPSLEY
FLEYDLQLLKWVLRG
>CA00g00230
MLEEELKRMKEENKKLATMLTTLGENYNSLRTNLIELQQKHSTHEEDNNSKLLSRKRKAEDVCCVNNSDINFEEA
SPKRPREIITTSISTVSVKTTLSDQTSLVKDGYNWRKYGQKVTRDNPSPRAYYKCSFAPTCPVKKKVQRSVKDPSV
LVATYEGEHNHPHPSQAETTAPLVNQGVITNPTFFNKFMEDINTSSLQKDLVAKMVPSLSKNPSFAATVAQPSLEY
FLEYDLQLLKWVLRG

CaWRKY56

>Capana12g001826 [mRNA] locus=Chr12:170920299-70933933
MENFSYNYSNPNPNNGDIYSSNFIDTPENFELSSYYLFPEDGLSDEFLSQNEFVQSASDSRRSYSNIKPAPTTTHHNM
QVKCTKGVMKKVDAKSRVAFRFRSELEVLDDGYKWRKYGKKMVKNSPNPRNYYKCSNGGCNVKKRVERDNE
DSSYVITTYEGIHNHESPHVIHYTQFPPNNIALHNLHL

>CA12g09290
MYEFRNYYKCSNGGCNVKKRVERDNEDSSYVITTYEGIHNHESPHVIHYTQFPPNNIALHNLHL

CaWRKY57

>Capana12g001851 [mRNA] locus=Chr12:173653320-173658337
MFSQSLLEDHQDMSSQLGFFSFPPNYNNVGMISTTTTATLPFIGYNQNTLKTLTMNIPPSFDHSLNIQESTHDPRRKE
DLSPIFGGPHLHSLQKSTPNTWAWGEVNESSNIIKRREFDHDHNLGVSSIKMKKIKSSRRKVREPRFCFKTMSDVDV
LDDGYKWRKYGQKVVKNTQHPRSYYRCTQDNCRVKKRVERLAEDPRMVITTYEGRHVHSPSHDEEDSQASSQL
NNLLW
>CA12g09140
MKKIKSSRRKVREPRFCFKTMSDVDVLDDGYKWRKYGQKVVKNTQHPRSYYRCTQDNCRVKKRVERLAEDPR
MVITTYEGRHVHSPSHDEEDSQASSQLNNLLW

CaWRKY58

> Capana00g000429 [mRNA] locus= Chr00:245677269..245678729
MENYPPLFPSSLSSHEFSLMNKKRSNTHAKEVLLFQGKNNGFLGLMASMETPSGVTNSFEDDVMKSCKKKGEKKI
KKPRY AFQTRSQVDILDDGYRWRKYGQKAVKNNKFPRSVINLIRSMYKLIMTFPNGNYLLVQRLGVFPNKIFYAN
ESTFSLSRFIVSFRDSFFTFFFFFKKSRGPKIK*VCNLMLYKGKNDICLTEVFYFLAITVQLLYIRSSRACLKRSKFLLF
GFEISYYVHLFSFVYIVRPDRTCARFASLLSILLGLCIVNLLQNEGARKFILVLQIRDVTILRNRGVLMWILILKNGW
VYTGSFEVKTNKNQLLEMEQIGTYIYYRLQTMIGVSNPNSGCGHHKKNVISLPAYFPLFNYYYYFGGANTSVLDIG
KCVELVLLQFTSTKIKCIAMFVVIRSYYRCTHQGCNVKKQVQRLSKDEGVVVTTYEGMHSHPIDKSTDNFEQILSQ
MQVYASF

CaWRKY59

>Capana00g001033 [mRNA] locus=Chr00:331116291-331117751
MENYPPLFPSSLSSHEFSLMNKKRSNTHAKEVLLFQGKNNGFLGLMASMETPSGVTNSFEDDVMKSCKKKGEKKI
KKPRYAFQTRSQVDILDDGYRWRKYGQKAVKNNKFPRSYYRCTHQGCNVKKQVQRLSKDEGVVVTTYEGMHS
HPIDKSTDNFEQILSQMQVYASF
>CA11g12710
MNKKRSNTHAKEVLLFQGKNNGFLGLMASMETPSGVTNSFEDDVMKSCKKKGEKKIKKPRYAFQTRSQVDILDD
GYRWRKYGQKAVKNNKFPRSYYRCTHQGCNVKKQVQRLSKDEGVVVTTYEGMHSHPIDKSTDNFEQILSQMQV
YASF

CaWRKY60

>Capana00g003083 [mRNA] locus=Chr00:523068129-523069793
MALDLFAIEQTASAGLKSMDHLIQFVSSNPTAKPDCREITEYTVSNFRNVISMLNRPTCHARVRRVGPVQPVKVAP
PVVSSPVVAPPMVAAPVEKEKEKEKMFRSTPALTFDFTKRKVAVPAAPSPAAGVGVVSKDVAMANSTNSSSSSFV
STITAEGSVSNGRVFPSMDLPPRPPVTAPAAFSGKPPIAGKRCRDHDVSDEFSGRTSSAGKCPCKKSKPKVKKVIRV
PAISSKTSDIPADEFTWRKYGQKPIKGSPYPRGYYRCSSLKGCPARKHVERATDDPRMLIVTYENDHEHHHNIQTA
FSGAAIGSRDGSSGQRMMVFESMGQK
>CA01g29700
MALDLFAIEQTASAGLKSMDHLIQFVSSNPTAKPDCREITEYTVSNFRNVISMLNRPTGHARVRRGXXVKVAPPVV
SSPVVAPPMVAAPVEKEKEKEKMFRSTPALTFDFTKRKVAVPAAPSPAAGVGVVSKDVAMANSTNSSSSSFVSTIT
AEGSVSNGRPPIAGKRCRDHDVSDEFSGRTSSAGKCPCKKSKPKVKKVIRVPAISSKTSDIPADEFTWRKYGQKPIK
GSPYPRGYYRCSSLKGCPARKHVERATDDPRMLIVTYENDHEHHHNIQTAFSGAAIGSRDGSSGQRMMVFESMGQ
K

CaWRKY61

>Capana00g004057 [mRNA] locus=Chr00:607660271-607664416
MGTPKEETTDEVFSENLEQKPEPDPATKSELKEKRSFESTSADVVSGELQKRLSPDADKQASKNNEEESTYPPTGQ
EVSRISQCDKGINVSQSNQEDISLSRVLENPSENVGQLQVLKSEAGASGSSQLSSLPKDSDAKSCGSESGVKRLSGK
ASDSSDQMQSSNTEILLSQSDQQRVNYPIQKREKALDKLQPRRNPDTSVHGLTSDQGVTLLRVPEKPSEDGYNWR
KYGQKLVRGNEYTRSYYKCTYPNCQAKKQVERSHDGHITDIHYIGKHEHPETPSGPQMLPELVLPLQMKQPEIPIIS
TLEAEGEKSTRPQETCEPSKPSEAPLVLDIVSACGGVKGTPLKRHKSETEVDKDDGSDSKRQKKDIVATVDTPPIKS
QSEPRHIVQTVSEVDIINDGQRWRKYGQKIVKGNPNPRSYYRCSVAGCPVKKHVERASHDPKVVITTYEGQHVHN
FPTPRDISQISPVPDVVTTAIRTDSRIESGHKHVVESKSESGERKHVGDSRTELGENKHIEKSKPELGGNKHVGDSKS
ESGESRHIGKSKIESGENKHVGGSRIESGGNKHVEESKLELGGNKHVGESIPESAENKHVGLDMAVHIGAN
>CA09g14010
MGTPKEETTDEVFSENLEQKPEPDPATKSELKEKRSFESTSADVVSGELQKRLSPDADKQASKNNEEESTYPPTGQ
EVSRISQCDKGINVSQSNQEDISLSRVLENPSENVGQLQVLKSEAGASGSSQLSSLPKDSDAKSCGSESGVKRLSGK
ASDSSDQMQSSNTEILLSQSDQQRVNYPIQKREKALDKLQPRRNPDTSVHGLTSDQGVTLLRVPEKPSEDGYNWR
KYGQKLVRGNEYTRSYYKCTYPNCQAKKQVERSHDGHITDIHYIGKHEHPETPSGPQMLPELVLPLQMKQPEIPIIS
TLEAEGEKSTRPQETCEPSKPSEAPLVLDIVSACGGVKGTPLKRHKSETEVDKDDGSDSKRQYVETPSCVCLWCFR
SRLYFKLIVVLLSCNRKKDIVATVDTPPIKSQSEPRHIVQTVSEVDIINDGQRWRKYGQKIVKGNPNPRSYYRCSVA
GCPVKKHVERASHDPKVVITTYEGQHVHNFPTPRDISQISPVPDVVTTAIRTDSRIESGHKHVVESKSESGERKHVG
DSRTELGENKHIEKSKPELGGNKHVGDSKSESGESRHIGKSKIESGENKHVGGSRIESGGNKHVEESKLELGGNKH
VGESIPESAENKHVGLDMAVHIGAN

CaWRKY62

> Capana00g004112 [mRNA] locus= Chr00:612680042..612682368

MFGSSTFQETSNVTSHHYQTINPNFAFHDPLINMNQDHGHNNNKYQDFDTSFLDMLLDGGDQEYYSNYLNNSYN
NNVSFYSENPFTQQEISSSTYSTSGNSSTASSFDATLTNIHMNHEVTKSNYVSRGGVKILNLFCLLEKTHELLNSDIIY
LYIYVAFFNINIRSRPKLRIISIIDCLILMFIIYLLVFVFWLFYRNSSMGIEKEKKGEKHAIAFRTKTELEILDDGYKWR
KYGKKKVKSNTNLRLIKILILYHITIIICFRLIILFQKYSINCTHIQIKANFGCKSLRYVFATRIAKIRIEFFYKYVDYTT
KKVLLLCVTKIWTILVTHLDSLSFSPNPTIVTLPPTLHPKTHTLTAPVVMLPLFMESQLTLFVRKIIYKDIKEEKKSSI
DILICTTFFFIVHSKKKKKNILSNVRHKSFKGEIERRPTCYNMYIFRLRSVILLLVHSLYSCRYPSNLFQSILFTVNFRF
LILLTYFTYNLIFYWSYLKLIFMRKKLTISKNSCKFNEIEGIHTHTHTQNIFFQKIIEIICSSHVNLFTLNLLIFVRIPVLP
EIYMLAKSEKTFDAKINSNFVILNRDGLILDTPNIQVHLTQVNRGFMFKYCLISFFYLFFRKRYYKFRNSSDFFTRGG
NTGITTSVQVEIAKRREKEMEMIQAILRMKDDTTMKAPLSFIAMMKCQHPSID
